# Conformation Analysis and Stereodynamics of Symmetrically *ortho*-Disubstituted Carvacrol Derivatives

**DOI:** 10.3390/molecules29091962

**Published:** 2024-04-25

**Authors:** Miljana R. Đorđević Zlatković, Niko S. Radulović, Miroslav Dangalov, Nikolay G. Vassilev

**Affiliations:** 1 Department of Chemistry, Faculty of Sciences and Mathematics, University of Niš, Višegradska 33, 18000 Niš, Serbia; miljana.djordjevic@pmf.edu.rs; 2 Institute of Organic Chemistry with Centre of Phytochemistry, Bulgarian Academy of Sciences, Acad. G. Bontchev Str. Bl. 9, 1113 Sofia, Bulgaria; miroslav.dangalov@orgchm.bas.bg

**Keywords:** carvacrol, rotational barrier energy, dynamic NMR, HOBS EXSY experiment, DFT calculations

## Abstract

The design and synthesis of analogs of natural products can be a valuable source of medicinal preparations for the pharmaceutical industry. In the present study, the structural elucidation of eleven derivatives of 2,4-dihalogeno substituted synthetic analogues of the natural compound carvacrol was carried out by means of NMR experiments, and of another thirteen by DFT calculations. By selective NOE experiments and the irradiation of CH signals of the isopropyl group, individual conformers were assigned as *syn* and *anti*. By comparing GIAO/B3LYP/6-311++G(d,p)-calculated and experimentally measured vicinal ^3^*J*_CH_ spin-spin constants, this assignment was confirmed. An unusual relationship is reported for proton-carbon vicinal couplings: ^3^*J*_CH_ (180°) < ^3^*J*_CH_ (0°). The conformational mobility of carvacrols was studied by 2D EXSY spectra. The application of homonuclear decoupling technique (HOBS) to these spectra simplifies the spectra, improves resolution without reducing the sensitivity, and allows a systematic examination of the rotational barrier of all compounds via their CH signals of the isopropyl group in a wider temperature interval. The rate constants of the isopropyl rotation between *syn* and *anti* conformers were determined and the corresponding energy barriers (14–17 kcal/mol) were calculated. DFT calculations of the energy barriers in carvacrol derivatives allowed the determination of the steric origin of the restricted isopropyl rotation. The barrier height depends on the size of the 2- and 4-position substituents, and is independent of the derivatization of the OH group.

## 1. Introduction

Atropisomerism is a form of axial chirality whereby enantiomers or diastereomers, known as atropisomers and atropodiastereomers, emerge as a result of constrained or hindered rotation along a σ-bond axis [1,2,3]. Despite the increasing attention paid to atropisomerism in the pharmaceutical sciences over the past decade, the presence of configurationally stable, axially chiral stereochemical elements in commercially available drugs remains rare even a century after the initial mention of axial atropisomerism [4]. The racemization process may last from a few seconds to several months or even decades. LaPlante and co-workers categorized atropisomers into three groups based on the energy required for the chiral axis to undergo racemization through rotation [5,6]. Atropisomers in class 1 have rotation barriers of <84 kJ/mol (20 kcal/mol) and racemize rapidly, typically on a minute or faster timescale at room temperature. Class 2 atropisomers have rotation barriers ranging from 84 to 117 kJ/mol (20–28 kcal/mol) and racemize over the hour-to-month timescale at room temperature. On the other hand, class 3 atropisomers have rotation barriers exceeding 117 kJ/mol (28 kcal/mol) and undergo racemization on a year or longer timescale at room temperature. Class 1 atropisomers have gained prominence in the field of drug discovery. Around 30% of small-molecule drugs approved by the FDA (Food and Drug Administration) since 2011 exhibit at least one chirality interconverting axis [7].

*Ortho*-substituted biaryl systems (C(*sp*^2^)-C(*sp*^2^)) are a class of widely distributed and extensively studied atropoisomeric compounds. On the other hand, nonbiaryl atropisomers with conformationally stable C(*sp*^2^)-C(*sp*^3^) single bonds have been left unexplored due to the rarity of restricted rotation around these bonds. Even though the first report suggesting the possibility of isolating *sp*^2^*-sp*^3^ atropisomers appeared in 1968 [8], only a few such compounds have been reported in the literature: carbinols [9,10,11,12], 9-arylfluorene derivatives and 9-aryl-9,10-dihydroanthracene [13,14], cannabidiols [15,16], 2-arylindoline derivatives [17] and hexahydro-1,2,4,5-tetrazines [18]. The existence of atropisomeric chirality has the potential to have a significant impact on the chemical, spectroscopic, and biological properties of natural products. Examples such as pegaharmols A–B [19], cordypyridones A−B [20,21], linderatin derivatives [22], and stilbene derivatives [23,24] illustrate stable natural atropisomers resulting from restricted rotation about the C(*sp*^2^)-C(*sp*^3^) bond.

Carvacrol, a phenolic monoterpene, is a major component of the essential oils found in plants of the Lamiaceae family. It is classified as “Generally Recognized As Safe” and is permitted for use in food [25,26,27]. Known for its diverse properties, including antimicrobial, anti-inflammatory, antioxidant, antitumor, analgesic, anti-hepatotoxic, and insecticidal effects [27], carvacrol has been extensively studied for its antibacterial activity against a range of Gram-positive and Gram-negative bacteria in food [27,28]. Research on carvacrol derivatives aims to enhance its biological properties, with many derivatives showing improved features compared to the original compound [29,30,31].

Surprisingly, there are no data in the literature on iodinated derivatives of carvacrol, while information on chlorinated and brominated derivatives is very limited [32,33,34,35]. The aim of the present study was to synthesize new 2,4-substituted halogen derivatives of carvacrol as a starting scaffold for new biologically active compounds. One would expect that both halogen atoms (Cl, Br or I) would be positioned *ortho* to the isopropyl group, and *ortho*, i.e., *para* to the phenolic group. Such regiochemistry could potentially cause the rotation around the aromatic core–isopropyl group bond (C(*sp*^2^)-C(*sp*^3^)) to be difficult, and as a result there are diastereoisomers of the atropisomeric type [12]. Majetich and Grove reported on dibromo derivatives of carvacrol, and the excess of signals in NMR spectra were explained by “open-orbital-induced heavy-atom effects” rather than the presence of a mixture of diastereoisomers [35]. This further encouraged us to investigate (both experimentally with dynamical NMR and theoretically with DFT calculations) the conformational exchange due to restricted rotation of the isopropyl group in disubstituted carvacrols.

**Figure 1:**
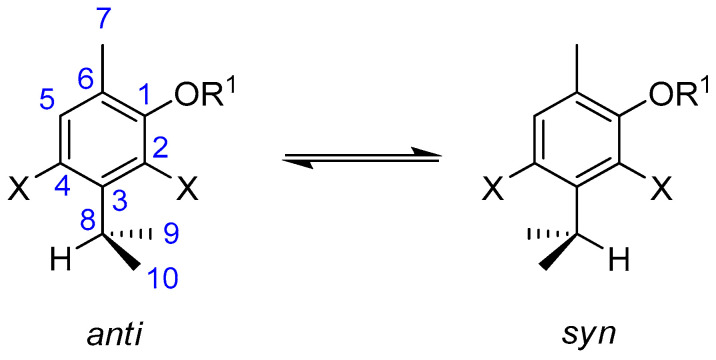
The studied atropisomers of 2,4-disubstituted carvacrols. For variation of substituents refer to Table 1.

Herein, we reveal a new class of rare aryl-C(*sp*^3^) atropisomers obtained by the derivatization of the natural product carvacrol. We present the synthesis, combined dynamic nuclear magnetic resonance (DNMR) spectroscopy, and density functional theory (DFT) study of the restricted rotation of the isopropyl group in 2,4-disubstituted carvacrols (Figure 1). By applying the homonuclear decoupling technique (HOBS experiment) in 1D and 2D NOESY spectra, we determined the rate constants of the isopropyl rotation between *syn* and *anti* conformers.

## 2. Results and Discussion

### 2.1. Synthesis of the Compounds

**Scheme 1:**
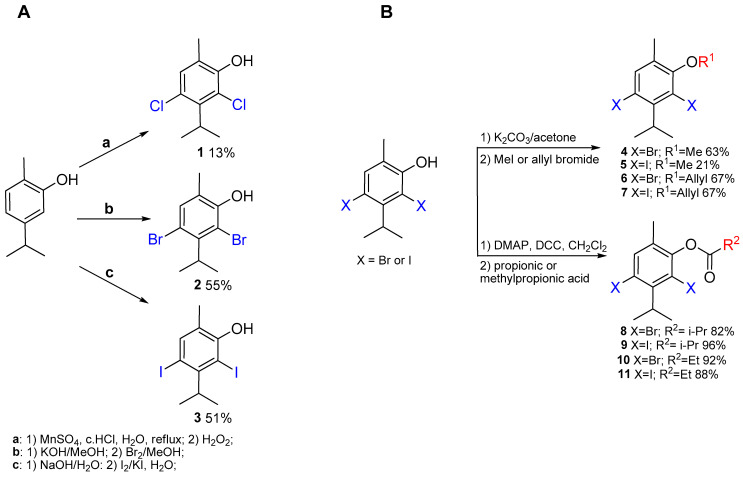
Synthesis of compounds **1**–**11**. (**A**) Halogenation of carvacrol. (**B**) Etherification and esterification of compounds **2** and **3**.

We were interested in synthesizing biologically active derivatives of natural products, and were prompted by the results obtained by studying the iodination reaction of thymol, including the isolation and identification of new, unexpected derivatives of thymol [36]. Here, carvacrol (a regioisomer of thymol) was subjected to iodination. The synthesis of 2,4-diiodo-substituted carvacrol was carried out in a similar manner to the iodation of thymol [36]. After repeated chromatographies on SiO_2_ and Sephadex LH-20 columns, a sample of 2,4-diiodo-3-isopropyl-6-methylphenol (**3**), pure based on TLC and GC-MS analyses, was isolated. Two sets of signals with comparable integrals were observed in the recorded ^1^H and ^13^C NMR spectra in deuterated chloroform at room temperature. A complete assignment of the observed resonances was accomplished by a careful analysis of the 1D and 2D NMR spectra (a series of selective homonuclear ^1^H decoupling, and grHSQC, grHMBC, gr^1^H–^1^H-COSY, ROESY, and NOESY experiments), and this confirmed the presence of two stable atropodiastereomers. Attempts followed to preparatively separate these diastereomers (using chromatography on Sephadex LH-20 and silica gel), but without success. The interconversion of these diastereomers is likely fast at the (high) temperature at which they eluted from the GC column, resulting in only one peak being detected. To confirm the existence of analogous atropisomers, we subsequently synthesized both dichloro and dibromo derivatives of carvacrol, compounds **2** and **3** (see Scheme 1A). Furthermore, additional functionalizations of the halogenated derivatives **2** and **3** were performed; thereby, corresponding ethers and esters were obtained, compounds **4**–**11** (see Scheme 1B). All synthesized compounds represented mixtures of two rotamers, in approximately equal populations. These other halogenated derivatives (compounds **1** and **2**) demonstrated how the size of the *ortho* substituent affects the rotational barrier of the observed atropisomers, while the alkylation or acylation of the phenolic OH group of **2** and **3** revealed how changing the size of the *meta*-supporting group influences the stability of the rotamers.

Spectral data for most of the synthesized halogenated derivatives of carvacrol do not exist in the literature, except for those related to compounds **1**, **2**, and **4**. In the work of Miller and Haggerty (1986), the ^1^H NMR spectra of compounds **1** and **2** are presented, without any mention of the presence of atropisomers, and they provide data only for one compound [33]. The ^1^H and ^13^C NMR, IR, and HRMS spectra for compound **4** are listed in the work of Majetich and Grove [35], but the existence of atropisomers was not recognized in their study. An apparently unexpected larger number of signals was explained as being the consequence of the “open-orbital-induced heavy-atom effects”. In the proton NMR spectrum, the signals were described as doublets originating from a single compound split by exceptionally high-value coupling constants. For example, in the ^1^H NMR spectrum, the H-8 signal is described as a doublet of septets with an additional coupling constant *J* = 46.8 Hz, corresponding to a difference of 0.117 ppm (at 400 MHz), or the difference in the shift of the two atropisomers that we observed. By comparing our spectral data with those in the literature, all signals expected for both atropisomers of 1,3-dibromo-2-isopropyl-4-methoxy-5-methylbenzene (**4**) were accounted for, indicating an error in the existing literature. These authors also provided NMR data for 3,5-dibromo-4-isopropyl-2-methoxybenzyl acetate, from which it can also be concluded that atropisomers are present. However, their explanation of the doubling in number of signals in the NMR spectra remained the same as for compound **4**.

### 2.2. Conformational Analysis and Molecular Geometry

**Figure 2:**
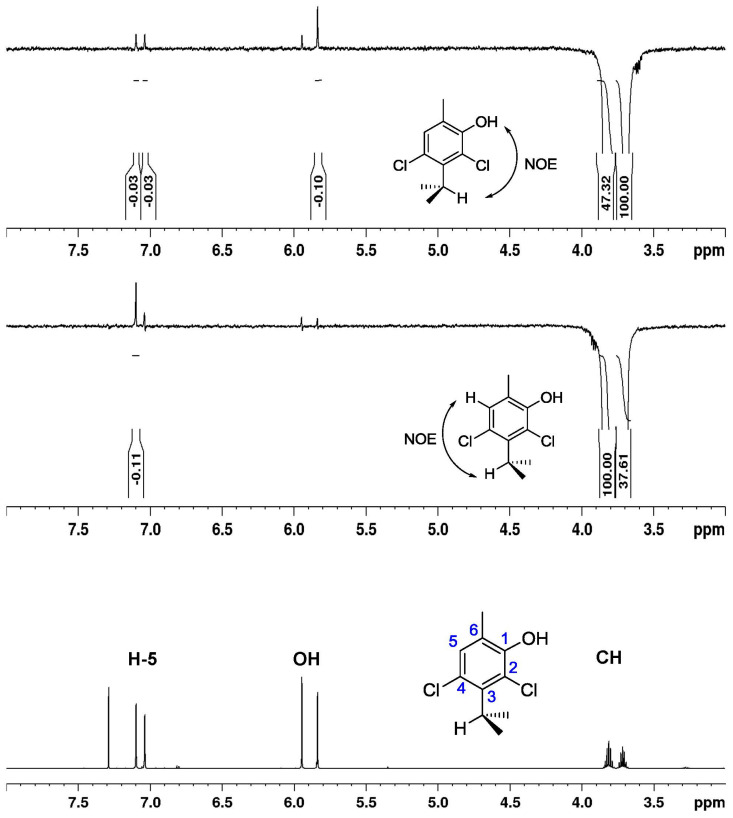
The NOE measurements and assignment of syn and anti atropisomers of **1**. The bottom ^1^H spectrum represents a regular proton spectrum at 238 K. The middle spectrum is a 1D NOE spectrum obtained at 238 K by selective excitation of the signal at 3.78 ppm (0.11% NOE effect on H-5 signal indicate proximity of H-5 and CH protons). The upper spectrum is a 1D NOE spectrum at 238 K obtained by selective excitation of the signal at 3.69 ppm (0.10% NOE effect on OH signal indicates the proximity of OH and CH protons).

**Figure 3:**
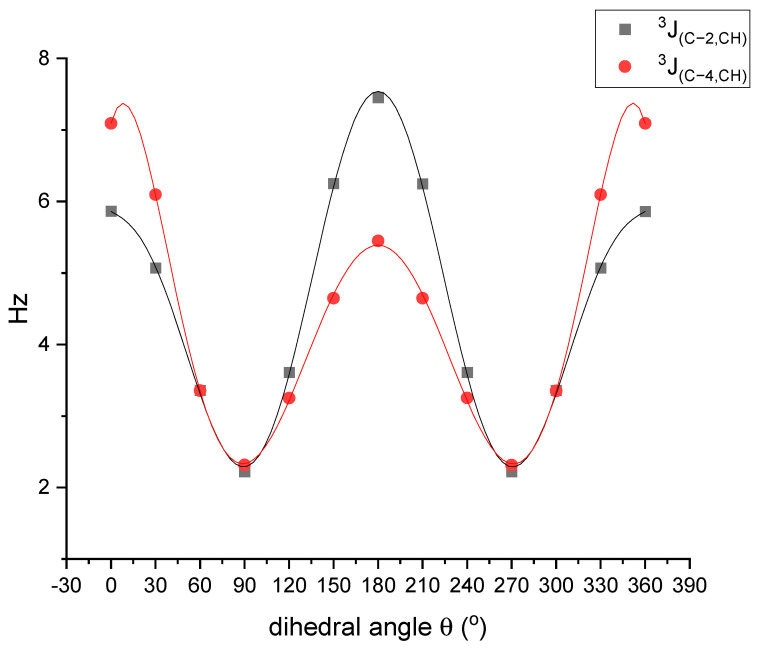
The relationship of vicinal coupling constants of carvacrol and the dihedral angle, based on GIAO/B3LYP/6-311++G**-J data.

The NMR spectra of the obtained and purified 2,4-disubstituted carvacrols **1**–**11** display signals indicating a mixture of two atropisomers, with their exchange occurring slowly on the NMR time scale. Our first task was to assign signals for the two isomers. Consequently, we measured the NOE under conditions of slow exchange, and the isomer is referred to as *anti* when the proximity of the methine proton of *i*-Pr (H-8) and H-5 was observed. In Figure 2, this approach is depicted for compound **1**. Although the NOE experiments reveal the *syn* and *anti* conformers, the observed NOEs were only in the order of 0.1%. Furthermore, in the case of compound **5**, the H-5 signals for the two atropisomers overlap. Consequently, we decided to employ a second approach: measuring the vicinal coupling constants between the CH proton from the *i*-Pr group and the carbon atoms C-2 and C-4. The usual relationship of the vicinal coupling constants should be ^3^*J*_CH_ (180°) > ^3^*J*_CH_ (0°). However, recently, an unusual relationship for proton vicinal couplings was reported, and we decided to calculate the vicinal couplings of the two rotamers of carvacrol (Figure 3) using GIAO/B3LYP/aug-cc-pVTZ-J [37,38] and GIAO/B3LYP/6-311++G**-J [39] methods.

Lately, it has been shown on test compounds that these methods are very accurate in calculating coupling constants [39]. The results of the calculations show that, in the case of carvacrol and its derivatives, there is an unusual relationship of proton–carbon vicinal couplings: ^3^*J*_CH_ (180°) < ^3^*J*_CH_ (0°) (refer to the Supplementary Materials). The calculation of vicinal couplings for all compounds confirms the assignment from the selective NOE experiments with the exception of **1,** in which the two C-4 signals of two conformers overlap in the ^13^C spectrum, and therefore the accurate determination of the coupling constants is rather difficult. Consequently, in the case of **1,** our assignment relies on NOE measurements alone.

### 2.3. Dynamic NMR Spectroscopy

Variable temperature ^1^H NMR spectra of compounds **1**–**11** were measured in CDCl_3_ in order to determine the effects of substituents on the rotational barrier. As the temperature decreases, the separation of signals for the two rotamers increases, allowing 2D EXSY spectra to be acquired in slow exchange over temperature intervals in order to obtain the rate constants using the volume integrals. The kinetic parameters for bond rotation were then extracted by Eyring analysis. In the case of compound **1**, the two isomers show well separated singlet signals for H-5 protons. The same signal of H-5 was used for dynamic NMR studies of compounds **4**, **8** and **10**. In most other studied compounds, with the exception of **3**, the signal for the CH protons from the isopropyl group was the only choice, since at a lower measured temperature the separation of other signals does not allow proper integration (Table 1). Thus, 2D EXSY spectra in the relevant region were measured in such a way as to extract the thermodynamic parameters of the exchange process. As a way to acquire more accurate data, by comparing the signals from the same group for all compounds, and to exclude the possibility of additional interactions (e.g., the phenolic proton of compound 3 also exchanges with the water present in the solvent), we chose to focus our efforts on “pure shift” techniques.

**Figure 4:**
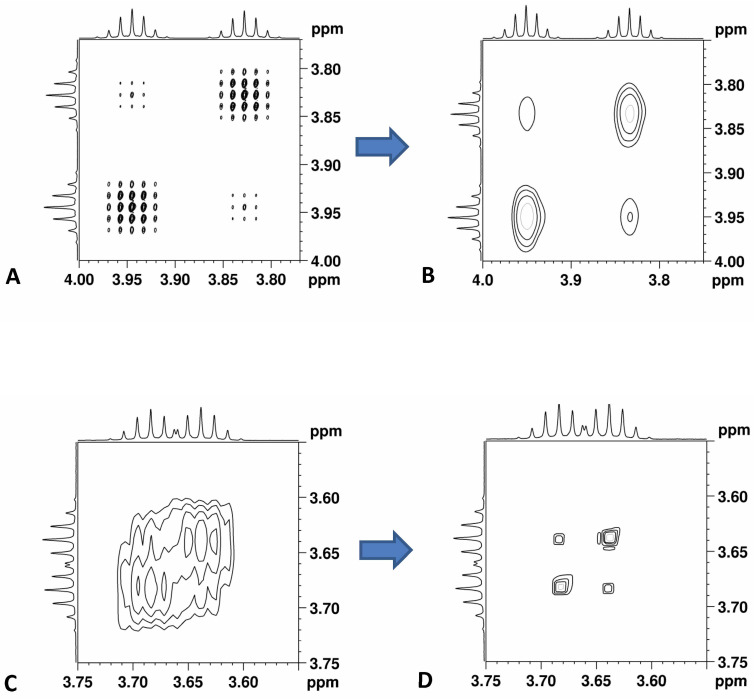
Comparison of the standard 2D ^1^H–^1^H EXSY and 2D ^1^H–^1^H HOBS EXSY experiments in the region of the methine proton of the isopropyl group. (**A**) ^1^H EXSY spectrum of compound **6** in CDCl_3_ at 253 K; (**B**) ^1^H HOBS EXSY spectrum of compound **6** in CDCl_3_ at 253 K; (**C**) ^1^H EXSY spectrum of compound **3** in CDCl_3_ at 253 K; (**D**) ^1^H HOBS EXSY spectrum of compound **3** in CDCl_3_ at 253 K.

The broadband ^1^H homodecoupled NMR [40,41], also known as a “pure shift” technique, relies on improving the resolution via refocusing the scalar coupling. This approach produces a spectrum displaying chemical shift resonances as collapsed singlets, avoiding the complications of overlapping multiplets. We implemented a homodecoupled band selective (HOBS) NMR [42,43,44,45] in 1D and 2D EXSY pulse programs to achieve homonuclear proton decoupling over a selected region of the whole spectrum, while the resonances outside this region are completely removed. Furthermore, HOBS, compared to other homonuclear decoupling approaches, is a quantitative method as it retains full sensitivity of the desired signals. Hence, we expect to obtain the same results for the rate constants as when using standard EXSY pulse programs (Figure 4; refer to Supplementary Materials for pulse program and more details). The rate constants of the isopropyl rotation between *syn* and *anti* conformers were determined and the corresponding energy barriers (14–17 kcal/mol) were calculated using HOBS EXSY spectra. The experimental activation parameters for both directions of the studied exchanging process are summarized in Table 2 and Tables S58–S61. In the case of rate constants, the HOBS EXSY results show a statistical deviation in some cases of up to 10% compared to the EXSY rate constants, which is absolutely within the range of the maximum relative error of the applied method. Accordingly, the comparison between the results derived from the HOBS EXSY and conventional EXSY spectra clearly demonstrates that the calculated energy barriers occasionally differ from each other. For example, the values for the *anti* to *syn* exchange process only in two cases exceed the limits of the statistical error (see Supplementary Materials, Tables S58–S61). By applying the HOBS EXSY experiment to determine the rate constants and activation parameters, we were able to study all compounds using the same signals for all of them (the methine proton of the isopropyl group), thus obtaining comparable accurate results for all compounds. This makes the application of HOBS EXSY quite valuable in cases of overlapping exchange signals to simplify proton spectra. As a result of the simplification of the spectrum, the temperature range of investigation is increased and reliable results can be obtained in an unambiguous way.

**Figure 5:**
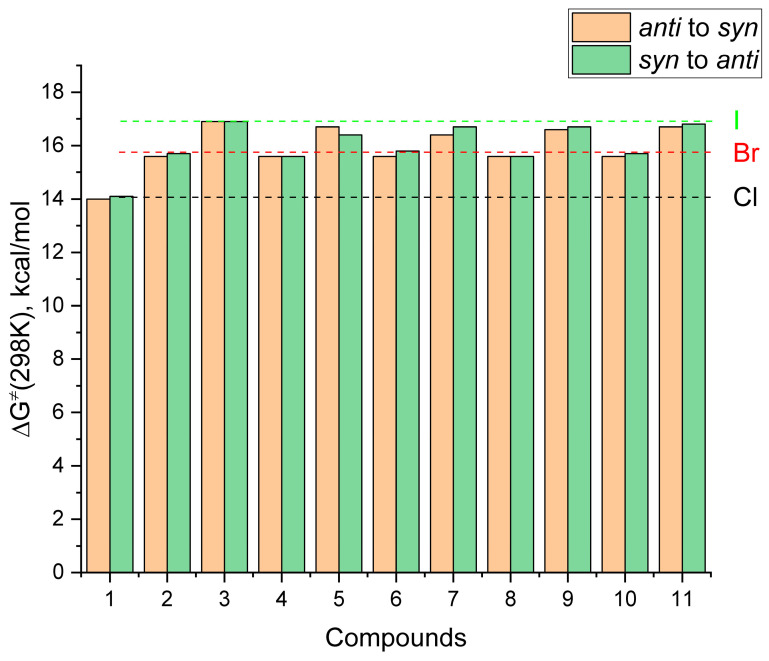
Experimental rotational barriers of the studied compounds in CDCl_3_.

The results indicate that the energy barrier is strongly dependent on the size of the substituents at positions 2 and 4 and is almost independent of the derivatization of the OH group. The dichloro derivative **1** has an energy barrier of 14.0–14.1 kcal/mol, the dibromo derivatives **2**, **4**, **6**, **8** and **10** have energy barriers of 15.6–15.8 kcal/mol, and the diiodo derivatives **3**, **5**, **7**, **9** and **11** have energy barriers of 16.4–16.9 kcal/mol. The dependence of the rotational barrier of the isopropyl group on the substituents at positions 2 and 4 can be clearly seen in Figure 5.

### 2.4. DFT Studies

**Scheme 2:**
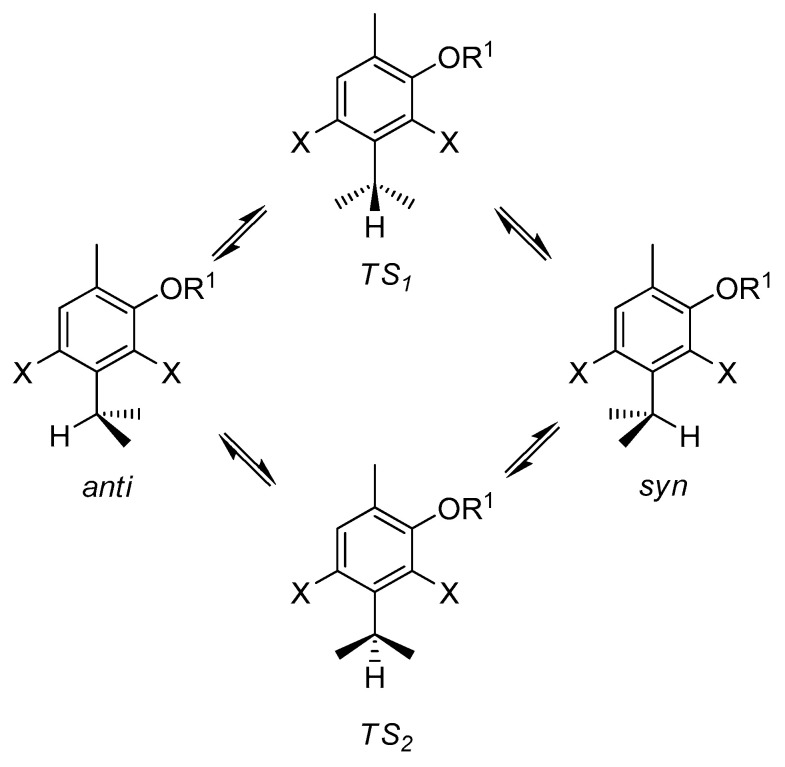
Ground state (*anti* and *syn*) and transition state (*TS*_1_ and *TS*_2_) structures of the studied carvacrols.

Further insights into the studied rotational isomerism come from DFT calculations, which were performed using SMD(CDCl_3_)/B3LYP/6-311G(d,p) theory. In addition to the two ground states, two possible transition states were also located (Scheme 2). The two ground states (GSs) have almost the same energy, and the populations of the two rotamers are around 50 to 50. Therefore, the calculated GS stabilities and their respective populations cannot be used to assign the exchange signals in the NMR spectra to a particular rotamer, as we have applied this approach before [46,47,48]. Calculating chemical shifts is also not applicable because the difference in chemical shifts of rotamers is too small. We trusted the calculated coupling constants and the measured NOE effect (see the discussion about the conformational analysis and molecular geometry above).

The localization of the two GSs and the two TSs enabled the calculation of the rotational energy barriers of the isopropyl group. The calculated thermodynamic parameters (Table 3 and Table S62) effectively reproduce the experimental values.

In addition to the DFT calculations of **1**–**11**, the same calculations were performed for carvacrol and 2,4-fluoro substituted carvacrol. The obtained results are in agreement with the expected energy barriers and with the dependence of this barrier on the steric hindrance of substituents. Steric hindrance is a consequence of steric effects. The steric properties of substituents have been evaluated in the literature via numerous methods: relative rates of chemical reactions [49], A-values (derived from equilibrium measurements of monosubstituted cyclohexanes) and others [50].

**Figure 6:**
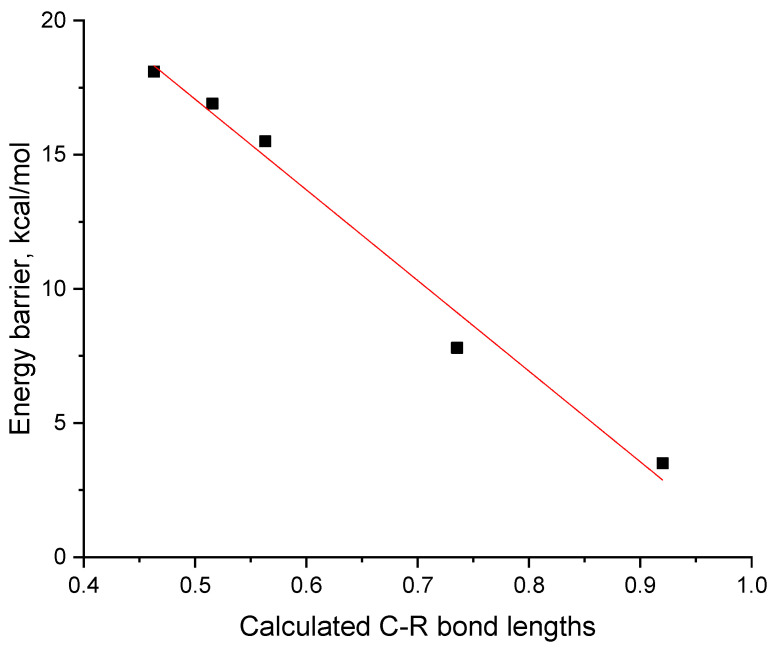
Dependence of the energy barriers of compounds **1**–**11** measured (or calculated) from the calculated bond lengths of substituents at positions 2 and 4 of the studied carvacrols.

**Figure 7:**
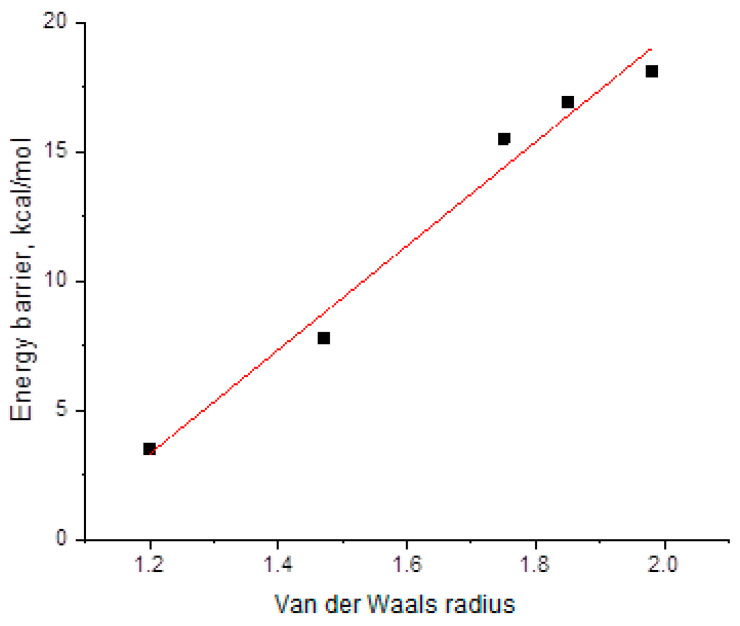
Dependence of the energy barriers of compounds **1**–**11** measured (or calculated) from the van der Waals radii of substituents at positions 2 and 4 of studied carvacrols.

The van der Waals repulsion is related to steric hindrance. It is realized when two substituents in a molecule approach each other within a distance less than the sum of their van der Waals radii. The steric hindrance of substituents can be illustrated by the dependence of the energy barriers of compounds **1**–**11** measured (or calculated) from C-R bond lengths (Figure 6) or from van der Waals radii (Figure 7) of the substituents at position 2 and 4 of the studied carvacrols.

During the rotation around the *sp*^3^*-sp*^2^ bond of the isopropyl group, there is a change in the length of this bond. The *i*-Pr-C bond is elongated by 0.028 Å in the di-Cl compound, by 0.026 Å in di-Br compounds, by 0.022 Å in diiodo compounds, by 0.020 Å in the difluoro compound and by only 0.011 Å in carvacrol. This extension of the *i*-Pr-C bond lengths does not correlate with the steric properties of substituents, but results from non-bonding interactions with the substituents at positions 2 and 4.

## 3. Materials and Methods

### 3.1. General Information

All chemicals were commercially available and used as received (Merck, Darmstadt, Germany; Fluka, Deutschland, Germany), except for the solvents, which were purified via distillation. All reactions were carried out in oven-dried glassware under an inert atmosphere of nitrogen. Column chromatography was performed using silica gel 60 with a particle size distribution of 0.02–0.045 mm (Carl Roth, Karlsruhe, Germany). Also, preparative separation was carried out on a Sephadex LH-20 (Merck, Darmstadt, Germany) column with a length of 50 cm (diameter 1.5 cm) using a mixture of chloroform and methanol in a ratio of 1:1 (*v*/*v*) as the eluent. Thin-layer chromatography (TLC) was conducted on aluminum plates precoated with silica gel and fluorescence indicator F_254_, with a layer thickness of 0.2 mm (Merck, Darmstadt, Germany). Visualization was achieved with UV light (254 nm) and by spraying the plates with a mixture of nitric and sulfuric acids (1:1), followed by brief heating at 110 °C. IR measurements (ATR-attenuated total reflectance) were performed using an FT-IR instrument, model 6700 (Thermo Nicolet, Waltham, MA, USA). UV spectra (in acetonitrile) were obtained using a UV-1800 spectrophotometer (Shimadzu, Tokyo, Japan). Microanalyses of carbon and hydrogen were carried out with a Carlo Erba Elemental Analyzer model 1106 (Carlo Erba Strumentazione, Invorio Italy). High-resolution mass spectrometry (HRMS) analyses were conducted using a JEOL MStation JMS-700 mass spectrometer (Akishima, Japan) with an ionization energy of 70 eV, an ionization trap current of 300 μA, and a source temperature of 230 °C. The error for each elemental composition data is given in units of atomic mass units (amu).

### 3.2. GC and GC-MS Analyses

GC-MS analyses were performed on a Hewlett-Packard 6890N gas chromatograph equipped with a fused silica capillary column DB-5MS (5% phenylmethylsiloxane, 30 m × 0.25 mm, film thickness 0.25 µm, Agilent Technologies, Palo Alto, CA, USA) and coupled with a 5975B mass selective detector from the same company. The injector and interface were operated at 250 °C and 320 °C, respectively. The oven temperature was raised from 70 °C to 310 °C at a heating rate of 5 °C min^−1^ and then isothermally held for 30 min. As a carrier gas, He at 1.0 mL min^−1^ was used. Samples (1 μL of the corresponding solutions in Et_2_O, 1 mg per 1 mL) were injected in split mode (the flow was 1.5 mL min^−1^ for the first 0.5 min and then set to 1.0 mL min^−1^ throughout the rest of the analysis: split ratio, 40:1). MS conditions included an ionization voltage of 70 eV, an acquisition mass range 35–650 amu, and a scan time 0.32 s. AMDIS software (version 2.73) was used for chromatogram deconvolution, and mass spectral libraries (Wiley 7, NIST 14, MassFinder 2.3, and Adams library [51]) were searched with NIST MS Search software (version 3.0).

### 3.3. Initial NMR Measurements at Room Temperature

Routine ^1^H and ^13^C NMR spectra were recorded on a Bruker Avance III spectrometer (Bruker, Ettlingen, Germany) operating at 400 and 100.6 MHz, respectively. Two-dimensional experiments (NOESY and gradient ^1^H–^1^H COSY, HSQC, HMBC), as well as DEPT-90, DEPT-135, and selective ^1^H homonuclear decoupling measurements, were run on same instrument with the built-in Bruker pulse sequences. All NMR spectra were measured at 25 °C in CDCl_3_ with (CH_3_)_4_Si as internal standard. Chemical shifts were reported as (*δ*) in parts per million (ppm) with respect to (CH_3_)_4_Si, and coupling constants *J* values are given in Hertz. The following abbreviations were used to designate multiplicities: s, singlet; d, doublet; dd, doublet of doublets; ddd, doublet of doublets of doublets; dt, doublet of triplets; dq, doublet of quartets; sept, septet.

### 3.4. General Procedure for Preparation

#### 3.4.1. Chlorination of Carvacrol

Carvacrol (5 g, 33 mmol), manganese sulphate (4.98 g, 33 mmol), and conc. hydrochloric acid (6.69 mL, 80 mmol) were added to water in a three-neck flask equipped with a reflux condenser(Merck, Darmstadt, Germany). The mixture was heated and stirred in an oil bath. Then, H_2_O_2_ (10.47 mL, 92 mmol of a 30% aqueous solution) was added dropwise during the reaction. After the reaction was complete, the mixture was allowed to stir for 1.5 h at room temperature, resulting in the formation of a distinct organic phase separated from the aqueous solution at the bottom. After that, the reaction mixture was extracted three times with diethyl ether. The combined organic extracts were dried over anhydrous MgSO_4_ and concentrated in vacuo. The crude chlorination products were purified via gradient flash dry column chromatography (eluent: *n*-hexane–diethyl ether (*v*/*v*)) to give the desired compound **1**.

#### 3.4.2. Bromination of Carvacrol

Bromine solution (0.639 g, 4.0 mmol) in methanol (5 mL) was slowly added drop by drop to a stirred solution of carvacrol (0.50 g, 3.3 mmol) in methanol (10 mL) with added KOH (0.559 g, 9.9 mmol). The mixture was stirred for additional 30 min, then concentrated in vacuo. The resulting reaction mixture was poured into water, neutralized with aqueous HCl (1:1, *v*/*v*), and extracted with diethyl ether. The organic layer was washed with an aqueous Na_2_S_2_O_3_ solution (10%) and dried over anhydrous MgSO_4_, and the solvent was removed using a rotary evaporator. The resulting residue was purified via gradient dry flash column chromatography (*n*-hexane–diethyl ether mixtures of increasing polarity (*v*/*v*)). The first two fractions of the mixture, which contained 2,4-dibromo-3-isopropyl-6-methylphenol and 2-bromo-3-isopropyl-6-methylphenol, were combined and further subjected to chromatography on Sephadex LH-20 (Merck, Darmstadt, Germany) using a mixture of MeOH and CHCl_3_ at a 1:1 (*v*/*v*) ratio.

#### 3.4.3. Iodination of Carvacrol

Five grams (33 mmol) of carvacrol was dissolved in a solution of 1.2 g (30 mmol) of sodium hydroxide in 20 mL of water. While stirring on a magnetic stirrer, a solution containing 6.0 g of iodine (23.6 mmol) and 9.0 g (54 mmol) of potassium iodide dissolved in 10 mL of water was added dropwise. The decolorization of the reaction mixture was considered to be the end of the reaction. The reaction mixture was neutralized with hydrochloric acid (1:1, v/v) and extracted with diethyl ether, and the combined organic layers were washed with aqueous sodium thiosulfate solution (10%). The organic layer was dried and concentrated in vacuo.

The obtained mixture (6.67 g) was initially fractionated via gradient dry-flash column chromatography (*n*-hexane–diethyl ether mixtures of increasing polarity (*v*/*v*)) on silica gel. The fractions were pooled based on thin-layer chromatography (1% diethyl ether in *n*-hexane (*v*/*v*)). Based on GC–MS analysis, fraction I (pure *n*-hexane was used as the eluent) contained a mixture of 3-isopropyl-2,4-diiodo-6-methylphenol and 3-isopropyl-2-iodo-6-methylphenol (*o*-iodocarvacrol), so it was further separated on Sephadex LH-20. After several re-chromatographies on Sephadex LH-20 with a mixture of MeOH and CHCl_3_ at a 1:1 (*v*/*v*) ratio, pure compounds were obtained, which were further used for the synthesis of diiodocarvacrol derivatives.

#### 3.4.4. Etherification of Compounds **2** and **3**

Either phenol **2** or **3** (0.174 mmol) was added to a suspension of anhydrous potassium carbonate (48 mg, 0.350 mmol) in acetone (5 mL). Methyl iodide (25 mg, 0.178 mmol) or allyl bromide (22 mg, 0.178 mmol) was then added, and the mixture was stirred for 24 h at room temperature. After that, acetone was evaporated under reduced pressure, and water was added. The resulting reaction mixture was extracted three times with diethyl ether. The combined extracts were dried and evaporated. The crude methylation product was purified via gradient silica gel column chromatography (eluent: *n*-hexane–diethyl ether (*v*/*v*)) to give the desired compounds **4**, **5**, **6**, and **7**.

#### 3.4.5. Esterification of Compounds **2** and **3**

A solution containing phenols **2** or **3** (0.075 mmol), 4-(dimethylamino)pyridine (DMAP, 1.2 mg, 0.01 mmol), *N*,*N*′-dicyclohexylcarbodiimide (DCC, 16.9 mg, 0.082 mmol), and either propanoic acid (6 mg, 0.082 mmol) or 2-methylpropanoic acid (7.2 mg, 0.082 mmol) in dry CH_2_Cl_2_ (10 mL) was stirred overnight at room temperature. For the workup, silica gel (5 g) was added to the resulting suspension. Dichloromethane was then removed under vacuum, and the resulting residue was subjected to purification using gradient silica gel column chromatography (eluent: *n*-hexane–diethyl ether (*v*/*v*)), yielding the desired esters **8**, **9**, **10**, and **11**.

### 3.5. Dynamic NMR Studies

^1^H spectra (0.1 M in 0.6 mL CDCl_3_) were recorded on a Bruker II+ 600 instrument (BBO probe) at 600.13 MHz in steps of 5 K (for T ranges refer to Table 1). Temperature calibration was carried out with a B-VT 3000 unit (it was checked and calibrated with methanol and ethylene glycol reference samples). ^1^H NMR spectra were acquired using a spectral width of 10 kHz, an acquisition time of 1.7 s, and 32 scans, zerofilled to 64 k datapoints (0.15 Hz per point) and processed without apodization. Peaks were fitted to a Lorentzian lineshape. ^1^H EXSY spectra were recorded on a BBO probe in steps of 5 K (for T ranges refer to Table 1). The spectra were acquired using a spectral width of 1.2 kHz, 2048 × 256 complex time domain data points, mixing times in the range of 0.03 to 0.3 s, and 2 scans in about 45 min. Linear prediction (32 coefficients and 256 points) in F1 was applied. The spectra were zerofilled to 4096 × 4096 data points and processed with a shifted square sine bell apodization in both dimensions. The populations were obtained via the integration of 1D 1H signals, and the exchange rates were calculated using the program EXSYCalc (MestreLab Research S.L., Santiago de Compostela, Spain)) from diagonal- and crosspeak integrals.

**Errors analysis**: Usually, the presented errors in the activation parameters are the statistical errors based on scattering of the data points around the Eyring straight line only. The errors in this analysis are due to inaccuracies in both the calculated rate constants, ***k***, and the measured temperatures, ***T*,** and are computed according to the error propagation equations of Binsch [52] and Heinzer and Oth [53], in which errors due to both the calculated rate constants and the measured temperature are taken into account. The absolute temperature errors were assumed to be ***σT*** = ±0.5 K, and the maximum relative error in rate constants was taken to be ***σk/k*** = ±10%.

### 3.6. HOmodecoupled Band-Selective NMR Experiments (HOBS)

^1^H HOBS EXSY spectra were recorded using hobs_noesy pulse program (refer to Section III. Pulse program codes for the HOBS-EXSY experiments) on a BBO probe in steps of 5 K typically between 233 and 313 K. The spectra were acquired using a spectral width of 2.4 kHz, 1024 × 128 complex time domain data points, mixing times in the range of 0.03 to 1.0 s, and 8 scans in about 45 min. Linear prediction (32 coefficients and 256 points) in F1 was applied. The spectra were zerofilled to 4096 × 4096 data points and processed with a shifted square sine bell apodization in both dimensions. The populations were obtained via the integration of 1D ^1^H signals, and the exchange rates were calculated using the program EXSYCalc (MestreLab Research S.L.) from diagonal- and crosspeak integrals.

### 3.7. NOE Experiments

Selective NOE ^1^H spectra were recorded on a Bruker NEO 600 instrument (prodigy BBO probe) at 600.18 MHz at the lower measured temperature (0.1 M in 0.6 mL CDCl_3_). ^1^H NMR spectra were acquired using the *selnogp* pulse program, an 80 ms selective Gaussian pulse, a mixing time of 1 s, a spectral width of 10 kHz, an acquisition time of 4.6 s, and 256 scans, zerofilled to 64 k datapoints (0.15 Hz per point) and processed with EM (LB=1) apodization.

### 3.8. DFT Calculations

All calculations were performed by means of quantum chemical calculations at the density functional theory (DFT) level using the Gaussian09 program package [54]. The geometries of all compounds were fully optimized, and the corresponding transition states were localized using B3LYP [55] functional with a 6-311++G(d,p) basis set [56]. Solvent effect was included implicitly in the optimizations via the SMD [57] model with the built-in parameters for the solvent (CDCl_3_). The nature of all critical points was confirmed by means of the vibrational analysis. The ∆*H*, ∆*S*, and ∆*G* values were calculated at T = 298.15 K at the same level of theory including zero-point energy in the particular solvent environment (represented by relative permittivity) and vibrational, rotational, and translational thermal energy corrections.

The NMR coupling constants of two rotamers of the studied compounds were calculated using the GIAO approximation [58,59] at the GIAO/B3LYP/6-311++G**-J computational level. The test calculations of the coupling constants [60,61,62,63] of two rotamers of carvacrol were performed using the GIAO/B3LYP/aug-cc-pVTZ-J [37,38] and GIAO/B3LYP/6-311++G**-J [39] methods. Recently, a 6-311++G**-J basis was introduced and shown on test compounds to be very accurate for calculating coupling constants [39].

## 4. Conclusions

The eleven (eight new) derivatives of 2,4-dihalogeno-substituted synthetic analogues of the natural compound carvacrol were synthesized and studied by dynamic NMR spectroscopy and DFT calculations. By selective NOE experiments and irradiating the CH signals of the isopropyl group, the individual conformers were assigned as *syn* and *anti*. By comparing GIAO/B3LYP/6-311++G(d,p)-calculated and experimentally measured vicinal ^3^*J*_CH_ spin-spin constants, this assignment was confirmed.

The conformational mobility of carvacrols was studied by 1D and 2D NOESY and ROESY spectra. The application of the homonuclear decoupling technique (HOBS) to these spectra simplifies the spectra, improves resolution without reducing the sensitivity, and allows a systematic examination of the rotational barrier of all compounds by their CH signals of the isopropyl group in a wider temperature interval. The rate constants of the isopropyl rotation between *syn* and *anti* conformers were determined, and the corresponding energy barriers (14–17 kcal/mol) were calculated. The DFT calculations of the energy barriers in carvacrol derivatives allowed the determination of the steric origin of the restricted isopropyl rotation. The barrier values mainly depend on the size of the 2- and 4-position substituents, and are almost independent of the derivatization of the OH group.

## Figures and Tables

**Table 1:** The studied 2,4-disubstituted carvacrols and NMR experimental condition.

Compound ^a^	X	R^1^	H ^b^	T Range, K	H ^c^	T Range, K
**1**	Cl	H	H-5	233–258	H-8	233–268
**2**	Br	H	H-8	253–273	H-8	253–283
**3**	I	H	OH	253–293	H-8	263–293
**4**	Br	CH_3_	H-5	253–278	H-8	253–273
**5**	I	CH_3_	H-8	253–273	H-8	263–293
**6**	Br	Allyl	H-8	243–283	H-8	253–283
**7**	I	Allyl	H-8	253–273	H-8	263–293
**8**	Br	C(=O)*i*-Pr	H-5	253–273	H-8	263–288
**9**	I	C(=O)*i*-Pr	H-8	263–283	H-8	263–293
**10**	Br	C(=O)Et	H-5	253–293	H-8	263–293
**11**	I	C(=O)Et	H-8	273–313	H-8	273–313
**12**	F	H				
**13**	H	H				

^a^ Compounds **12** and **13** were studied only by DFT calculations. ^b^ Protons used in 2D EXSY experiments. ^c^ Protons used in 2D HOBS-NOESY.

**Table 2:** Experimental HOBS EXSY activation parameters for the *anti* to *syn* exchange process of the studied compounds in CDCl_3_.

Compound	Process	X	R^1^	∆H^≠^(298 K), kcal/mol	∆S^≠^(298 K), e.u.	∆G^≠^(298 K), kcal/mol	T Range, K
**1**	*anti* to *syn*	Cl	H	15.4 ± 1.0	4.2 ± 3.6	14.1 ± 0.1	233–268
	*syn* to *anti*			15.5 ± 0.7	5.0 ± 2.7	14.0 ± 0.1	
**2**	*anti* to *syn*	Br	H	16.2 ± 1.1	1.9 ± 3.2	15.6 ± 0.1	253–283
	*syn* to *anti*			17.5 ± 0.8	7.4 ± 3.0	15.3 ± 0.1	
**3**	*anti* to *syn*	I	H	12.3 ± 1.2	−15.2 ± 4.4	16.9 ± 0.1	263–293
	*syn* to *anti*			13.0 ± 1.0	−13.0 ± 3.5	16.9 ± 0.1	
**4**	*anti* to *syn*	Br	CH_3_	18.7 ± 1.2	10.9 ± 4.4	15.5 ± 0.1	253–273
	*syn* to *anti*			16.8 ± 1.6	3.9 ± 12.6	15.6 ± 0.1	
**5**	*anti* to *syn*	I	CH_3_	16.7 ± 0.9	0.0 ± 2.7	16.7 ± 0.1	263–293
	*syn* to *anti*			16.6 ± 0.9	0.0 ± 1.2	16.6 ± 0.1	
**6**	*anti* to *syn*	Br	Allyl	15.2 ± 0.8	−1.9 ± 2.4	15.8 ± 0.1	253–283
	*syn* to *anti*			16.3 ± 0.8	1.9 ± 2.5	15.7 ± 0.1	
**7**	*anti* to *syn*	I	Allyl	16.4 ± 0.9	−0.1 ± 1.4	16.4 ± 0.1	263–293
	*syn* to *anti*			16.3 ± 0.8	−1.1 ± 3.9	16.7 ± 0.1	
**8**	*anti* to *syn*	Br	C(=O)*i*-Pr	17.4 ± 1.1	7.2 ± 4.5	15.3 ± 0.1	263–288
	*syn* to *anti*			17.5 ± 1.4	7.6 ± 6.4	15.2 ± 0.1	
**9**	*anti* to *syn*	I	C(=O)*i*-Pr	18.1 ± 1.2	5.3 ± 3.7	16.6 ± 0.1	263–293
	*syn* to *anti*			17.0 ± 0.8	0.6 ± 3.2	16.8 ± 0.1	
**10**	*anti* to *syn*	Br	C(=O)Et	14.6 ± 0.9	−3.4 ± 2.9	15.6 ± 0.1	263–293
	*syn* to *anti*			14.8 ± 1.0	−2.9 ± 2.8	15.6 ± 0.1	
**11**	*anti* to *syn*	I	C(=O)Et	15.9 ± 0.8	−2.8 ± 3.1	16.7 ± 0.1	273–313
	*syn* to *anti*			15.4 ± 0.8	−4.8 ± 2.4	16.8 ± 0.1	

**Table 3:** SMD(CDCl_3_)/B3LYP/6-311G(d,p) calculated activation parameters for the *anti* to *syn* exchange processes of the studied compounds (ZPE, thermal and entropy corrections are calculated using SMD(CDCl_3_)/B3LYP/6-311G(d,p) theory for 298 K).

Compound	Barrier	ΔH^≠^(298 K)	ΔS^≠^(298 K)	ΔG^≠^(298 K)	ΔG^≠^(298 K)^eff^
**1**	TS1—*anti*-GS	14.4	−4.9	15.9	15.5
	TS2—*anti*-GS	14.4	−4.9	15.9	
**2**	TS1—*anti*-GS	16.0	−4.8	17.4	17.0
	TS2—*anti*-GS	16.0	−4.8	17.4	
**3**	TS1—*anti*-GS	17.1	−4.9	18.6	18.1
	TS2—*anti*-GS	17.1	−4.9	18.6	
**4**	TS1—*anti*-GS	15.6	−4.3	16.9	16.7
	TS2—*anti*-GS	16.2	−4.5	17.5	
**5**	TS1—*anti*-GS	16.3	−3.5	17.3	17.3
	TS2—*anti*-GS	17.1	−4.5	18.4	
**6**	TS1—*anti*-GS	16.1	−3.8	17.2	16.0
	TS2—*anti*-GS	15.5	−2.2	16.1	
**7**	TS1—*anti*-GS	17.2	−4.7	18.6	17.6
	TS2—*anti*-GS	16.4	−4.4	17.7	
**8**	TS1—*anti*-GS	15.5	−4.2	16.7	16.7
	TS2—*anti*-GS	16.3	−5.7	18.0	
**9**	TS1—*anti*-GS	16.0	−2.8	16.9	17.0
	TS2—*anti*-GS	17.4	−2.5	18.2	
**10**	TS1—*anti*-GS	15.6	−4.2	16.9	16.7
	TS2—*anti*-GS	16.2	−4.3	17.4	
**11**	TS1—*anti*-GS	16.5	−3.3	17.5	17.4
	TS2—*anti*-GS	17.3	−4.5	18.7	
**12**	TS1—*anti*-GS	6.2	−5.8	8.0	7.6
	TS2—*anti*-GS	6.2	−5.8	8.0	
**13**	TS1—*anti*-GS	1.9	−7.1	4.0	3.6
	TS2—*anti*-GS	1.9	−7.1	4.0	

## Data Availability

The data are available within the article and Supplementary Materials.

## Supplementary Materials

The following supporting information can be downloaded at: https://www.mdpi.com/article/10.3390/molecules29091962/s1, Synthesis and characterization data and NMR for compounds **1**–**11.** Variable-temperature NMR spectra of compounds **1**–**11** with assignment of *anti* and *syn* rotamers using ^3^*J*_(C-2,CH)_ and ^3^*J*_(C-4,CH)_ coupling constants and NOE effects, analysis of ^1^H EXSY and HOBS EXSY spectra, pulse program codes for the HOBS-EXSY experiments, DFT optimized structures of the two ground state conformations of **1**–**13**, optimized TS1 and TS2 transition state structures for compounds **1**–**13**, and theoretical predictions of coupling constants in carvacrol. References [37,38,39,51,52,53,54,55,56,57,58,59,60,61,62,63] are cited in the Supplementary Materials.
