# Supplementary material for: Conformation Analysis and Stereodynamics of Symmetrically ortho-Disubstituted Carvacrol Derivatives
Source: Molecules. 2024 Apr 25;29(9):1962. doi: 10.3390/molecules29091962 (PMC11085911; doi:10.3390/molecules29091962)
Supplement: Supplementary file 1 [file molecules-29-01962-s001.zip › molecules-2953228-supplementary.pdf]

Supporting Information

Conformation Analysis and Stereodynamics of Symmetrically *ortho*-Disubstituted Carvacrol Derivatives

Miljana R. Đorđević Zlatković [1], Niko S. Radulović [1,*], Miroslav Dangalov [2] and Nikolay G. Vassilev [2,*]

[1] Department of Chemistry, Faculty of Sciences and Mathematics, University of Niš, Višegradska 33, 18000, Niš, Serbia; nikoradulovic@yahoo.com (N.S.R.); miljanadjordjevic11@gmail.com (M.R.Đ.Z.)

[2] Institute of Organic Chemistry with Centre of Phytochemistry, Bulgarian Academy of Sciences, Acad. G. Bontchev str. Bl. 9, 1113, Sofia, Bulgaria; miroslav.dangalov@orgchm.bas.bg (M.D.); nikolay.vassilev@orgchm.bas.bg (N.G.V.)

* Correspondence: nikoradulovic@yahoo.com; nikolay.vassilev@orgchm.bas.bg

Table of contents:

Experimental Procedures 2

- General procedure for preparation and spectral data for compounds **1-11** 3
- Dynamic NMR studies 19
- NOE Experiments 19
- NMR Structure elucidation 20
- DFT calculations 20
- ^{1}H and ^{13}C NMR spectra of compounds **1-11** 21

Dynamic NMR studies for compounds **1-11** 32

Pulse program codes for the HOBS-EXSY experiments 87

DFT calculations 100

Experimental Procedures

General Information

All chemicals were commercially available and used as received (Merck, Darmstadt, Germany; Fluka, Deutschland, Germany), except for the solvents, which were purified via distillation. All reactions were carried out in oven-dried glassware under an inert atmosphere of ni-trogen. Column chromatography was performed using silica gel 60 with a particle size distribution of 0.02–0.045 mm (Carl Roth, Karlsruhe, Germany). Also, preparative separation was carried out on a Sephadex LH-20 (Merck, Darmstadt, Germany) col-umn with a length of 50 cm (diameter 1.5 cm) using a mixture of chloroform and methanol in a ratio of 1:1 (v/v) as the eluent. Thin-layer chromatography (TLC) was conducted on aluminum plates precoated with silica gel and fluorescence indicator F254, with a layer thickness of 0.2 mm (Merck, Darmstadt, Germany). Visualization was achieved with UV light (254 nm) and by spraying the plates with a mixture of nitric and sulfuric acids (1:1), followed by brief heating at 110 ◎C. IR measurements (ATR-attenuated total reflectance) were performed using an FT-IR instrument, model 6700 (Thermo Nicolet, Waltham, MA, USA). UV spectra (in acetonitrile) were obtained us-ing a UV-1800 spectrophotometer (Shimadzu, Tokyo, Japan). Microanalyses of carbon and hydrogen were carried out with a Carlo Erba Elemental Analyzer model 1106 (Carlo Erba Strumentazione, Invorio Italy). High-resolution mass spectrometry (HRMS) analyses were conducted using a JEOL MStation JMS-700 mass spectrometer (Akishima, Japan) with an ionization energy of 70 eV, an ionization trap current of 300 μA, and a source temperature of 230 ◎C. The error for each elemental composition data is given in units of atomic mass units (amu).

GC and GC-MS analyses

GC-MS analyses were performed on a Hewlett-Packard 6890N gas chromato-graph equipped with a fused silica capillary column DB-5MS (5% phenylmethylsilox-ane, 30 m ◎ 0.25 mm, film thickness 0.25 μm, Agilent Technologies, Palo Alto, CA, USA) and coupled with a 5975B mass selective detector from the same company. The injector and interface were operated at 250 ◎C and 320 ◎C, respectively. The oven tem-perature was raised from 70 ◎C to 310 ◎C at a heating rate of 5 ◎C min-1 and then iso-thermally held for 30 min. As a carrier gas, He at 1.0 mL min-1 was used. Samples (1 μL of the corresponding solutions in Et2O, 1 mg per 1 mL) were injected in split mode (the flow was 1.5 mL min-1 for the first 0.5 min and then set to 1.0 mL min-1 throughout the rest of the analysis: split ratio, 40:1). MS conditions included an ionization voltage of 70 eV, an acquisition mass range 35–650 amu, and a scan time 0.32 s. AMDIS software (version 2.73) was used for chromatogram deconvolution, and mass spec-tral libraries (Wiley 7, NIST 14, MassFinder 2.3, and Adams library [1]) were searched with NIST MS Search software (version 3.0)..

Initial NMR measurements at room temperature

Routine ^{1}H and ^{13}C NMR spectra were recorded on a Bruker Avance III spectrometer (Bruker, Ettlingen, Germany) operating at 400 and 100.6 MHz, respectively. Two-dimensional experiments (NOESY and gradient 1H–1H COSY, HSQC, HMBC), as well as DEPT-90, DEPT-135, and selective 1H homonuclear decoupling measurements, were run on same instrument with the built-in Bruker pulse sequences. All NMR spec-tra were measured at 25 ◎C in CDCl3 with (CH3)4Si as internal standard. Chemical shifts were reported as (δ) in parts per million (ppm) with respect to (CH3)4Si, and cou-pling constants J values are given in Hertz. The following abbreviations were used to designate multiplicities: s, singlet; d,

doublet; dd, doublet of doublets; ddd, doublet of doublets of doublets; dt, doublet of triplets; dq, doublet of quartets; sept, septet.

General procedure for preparation

Chlorination of carvacrol

Carvacrol (5 g, 33 mmol), manganese sulphate (4.98 g, 33 mmol), and conc. hy-drochloric acid (6.69 mL, 80 mmol) were added to water in a three-neck flask equipped with a reflux condenser (Merck, Darmstadt, Germany). The mixture was heated and stirred in an oil bath. Then, H2O2 (10.47 mL, 92 mmol of a 30% aqueous solution) was added dropwise during the reac-tion. After the reaction was complete, the mixture was allowed to stir for 1.5 hours at room temperature, resulting in the formation of a distinct organic phase separated from the aqueous solution at the bottom. After that, the reaction mixture was extract-ed three times with diethyl ether. The combined organic extracts were dried over an-hydrous MgSO4 and concentrated in vacuo. The crude chlorination products were pu-rified via gradient flash dry column chromatography (eluent: n-hexane–diethyl ether (v/v)) to give the desired compound 1.

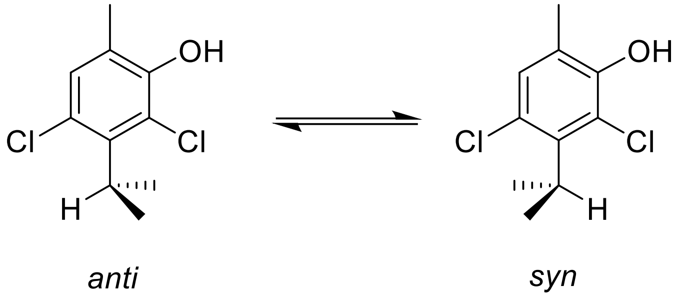

2,4-Dichloro-3-isopropyl-6-methylphenol (3,5-dichlorocarvacrol) (1): colorless oil, yield 13%; ^{1}H NMR (400 MHz, 298 K, $CDCl_3$, ppm): mixture of *syn* and *anti* rotamers around *i*-Pr-Ar bond: δ = 1.39 (d, *J*=7.2 Hz, 6H; CH_3), 2.21 (d, *J*=0.8 Hz, 3H; CH_3), 3.77 (bs, 1H; CH), 5.78 (bs, 1H; OH), 7.04 (bs, 1H; H-5); ^{13}C NMR (100.6 MHz, 298 K, $CDCl_3$, ppm): mixture of *syn* and *anti* rotamers around *i*-Pr-Ar bond: δ = 16.06 (CH_3), 19.69 (CH_3), 31.77 (CH), 124.06 (C-2), 124.83 (C-6), 124.06 (C-4), 130.23/131.48 (C-5), 139.28 (C-3), 149.16 (C-1); FTIR (neat) ν_{max}/cm^{-1} 3419, 2960, 2926, 2871, 1600, 1455, 1396, 1222, 1139, 859, 775, 652; UV/Vis (CH_3CN, nm): λ_{max}(log ε)=290.0 (3.47), 283.0 (3.45); EI-MS: *m/z* (%): 218 (33.8) [*M*]$^+$, 220 (21.7) [*M*+2]$^+$, 207 (10.8), 205 (64.7), 203 (100), 168 (19.6), 103 (12.7), 77 (15.0).

^{1}H NMR (600 MHz, 233 K, $CDCl_3$): Mixture of *syn* and *anti* rotamers around *i*-Pr-Ar bond: 0.54 : 0.46. *Anti* major rotamer: δ = 1.375 (d, *J*=7.2 Hz, 6H, CH_3), 2.201 (d, *J*=0.6 Hz, 3H, CH_3), 3.686 (septet, *J*=7.2 Hz, 1H, CH), 5.811 (s, 1H, OH), 7.011 (s, 1H, H-5); *Syn* minor rotamer: δ = 1.358 (d, *J*=7.3 Hz, 6H, CH_3), 2.205 (d, *J*=0.7 Hz, 3H, CH_3), 3.782 (sept, *J*=7.3 Hz, 1H, CH), 5.921 (s, 1H, OH), 7.072 (s, 1H, H-5).

^{13}C NMR (150.9 MHz, 233 K, $CDCl_3$): Mixture of *syn* and *anti* rotamers around *i*-Pr-Ar bond: 0.54 : 0.46. *Anti* major rotamer: δ = 16.30 (CH_3), 19.45 (CH_3), 31.77 (CH), 120.48 (C-2), 123.79 (C-6), 124.41 (C-4), 131.33 (C-5), 138.75 (C-3), 148.10 (C-1); *Syn* minor rotamer: δ = 16.38 (CH_3), 19.45 (CH_3), 31.30 (CH), 120.06 (C-2), 123.83 (C-6), 124.46 (C-4), 129.89 (C-5), 138.84 (C-3), 148.96 (C-1).

Selective NOE: The irradiation of multiplet at 3.811 ppm of major isomer at 238 K produce 0.11% NOE of signal at 7.099 ppm, while the irradiation of multiplet at 3.715 ppm produce 0.03% NOE of signal at 7.037 ppm and 0.10% NOE of signal at 5.836 ppm (OH). This result confirms that major rotamer is *anti* (CH is closer to H-5) and minor rotamer is *syn* (CH is closer to OH).

Table S1. GIAO/B3LYP/6-311++G(d,p) calculations of coupling constants of the two rotamers of **1**

Rotamers	$^3J_{(C\text{-}2,CH)}$	$^3J_{(C\text{-}4,CH)}$	$^2J_{(C\text{-}4,H\text{-}5)}$	$^4J_{(C\text{-}2,H\text{-}5)}$	$^2J_{(C\text{-}2,OH)}$
Anti	6.8	7.2	-3.6	-1.6	
Syn	7.3	6.4	-3.6	-1.5	

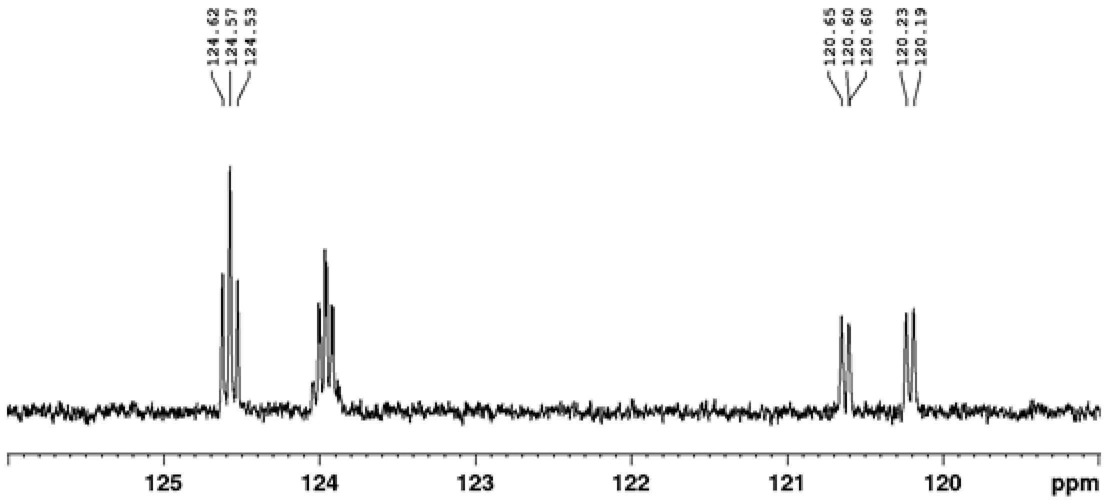

Figure S1. ^{13}C NMR spectrum of **1** at 233 K with selective irradiation of H-5 and OH signals

Bromination of carvacrol

Bromine solution (0.639 g, 4.0 mmol) in methanol (5 mL) was slowly added drop by drop to a stirred solution of carvacrol (0.50 g, 3.3 mmol) in methanol (10 mL) with added KOH (0.559 g, 9.9 mmol). The mixture was stirred for additional 30 minutes, then concentrated in vacuo. The resulting reaction mixture was poured into water, neutralized with aqueous HCl (1:1, v/v), and extracted with diethyl ether. The organic layer was washed with an aqueous Na2S2O3 solution (10%) and dried over anhydrous MgSO4, and the solvent was removed using a rotary evaporator. The resulting residue was purified via gradient dry flash column chromatography (n-hexane–diethyl ether mixtures of increasing polarity (v/v)). The first two fractions of the mixture, which contained 2,4-dibromo-3-isopropyl-6-methylphenol and 2-bromo-3-isopropyl-6-methylphenol, were combined and further subjected to chro-matography on Sephadex LH-20 (Merck, Darmstadt, Germany) using a mixture of MeOH and CHCl3 at a 1:1 (v/v) ra-tio.

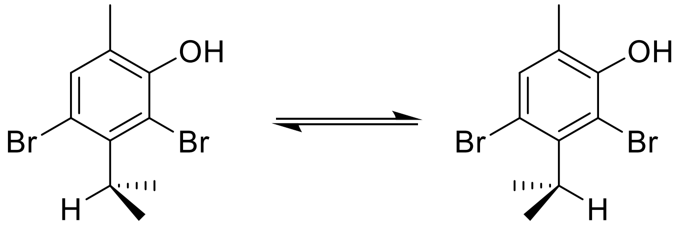

anti *syn*

2,4-dibromo-3-isopropyl-6-methylphenol (3,5-dibromocarvacrol) (2): colorless oil, yield 55%; ^{1}H NMR (400 MHz, 298 K, $CDCl_3$, ppm): mixture of *syn* and *anti* rotamers around *i*-Pr-Ar bond; rotamer 1: δ = 1.41 (m, 6H; CH_3), 2.23 (d, *J*=0.6 Hz, 3H; CH_3), 3.84 (septet, *J*=7.3 Hz, 1H; CH), 5.96 (s, 1H; OH), 7.31 (s, 1H; H-5); rotamer 2: δ = 1.41 (m, 6H; CH_3), 2.23 (s, 3H; CH_3), 3.75 (septet, *J*=7.3 Hz, 1H; CH), 5.79 (s, 1H; OH), 7.27 (s, 1H; H-5); ^{13}C NMR (100.6 MHz, 298 K, $CDCl_3$, ppm): mixture of *syn* and *anti* rotamers around *i*-Pr-Ar bond; rotamer 1: δ = 16.25/16.14 (CH_3), 19.51 (CH_3), 35.88 (CH), 111.20 (C-2), 124.52 (C-6), 115.49 (C-4), 134.09 (C-5), 141.59 (C-3), 150.59 (C-1); rotamer 2: δ = 16.25/16.14 (CH_3), 19.51 (CH_3), 36.24 (CH), 114.94 (C-2), 124.52 (C-6), 111.49 (C-4), 135.79 (C-5), 141.50 (C-3), 149.78 (C-1); UV/Vis (CH_3CN, nm): λ_{max}(log ε)=291.0 (3.31), 206.0 (4.73); FTIR (neat) ν_{max}/cm^{-1} 3494, 2959, 2927, 2874, 1598, 1451, 1349, 1220, 1133, 732, 708, 627, 610; EI-MS: *m*/*z* (%): 308 (55.9) [*M*]$^+$, 310 (27.2) [*M*+2]$^+$, 306 (28.8), 293 (100), 291 (53.3), 212 (37.7), 77 (17.2), 51 (11.6).

^{1}H-NMR (600 MHz, T=253 K, $CDCl_3$): Mixture of *anti* and *syn* rotamers around *i*-Pr-Ar bond: 0.53 : 0.47. *Anti* major rotamer: δ = 1.40 (d, *J*=7.3 Hz, 6H, CH_3), 2.25 (d, *J*=0.7 Hz, 3H, CH_3), 3.83 (septet, *J*=7.3 Hz, 1H, CH), 6.04 (d, *J*=0.4 Hz, 1H, OH), 7.33 (s, 1H, H-5); *Syn* minor rotamer: δ = 1.42 (d, *J*=7.2 Hz, 6H, CH_3), 2.24 (d, *J*=0.7 Hz, 3H, CH_3), 3.75 (septet, *J*=7.2 Hz, 1H, CH), 5.87 (d, *J*=0.6 Hz, 1H, OH), 7.29 (s, 1H, H-5).

^{13}C-NMR (150.9 MHz, T=253 K, $CDCl_3$): Mixture of *anti* and *syn* rotamers around *i*-Pr-Ar bond: 0.53 : 0.47. *Anti* major rotamer: δ = 16.67 (CH_3), 19.54 (CH_3), 35.93 (CH), 111.22 (C-2), 115.49 (C-4), 124.57 (C-6), 134.08 (C-5), 141.43 (C-3), 150.50 (C-1); *Syn* minor rotamer: δ = 16.53 (CH_3), 19.54 (CH_3), 36.31 (CH), 111.85 (C-4), 115.00 (C-2), 124.57 (C-6), 135.77 (C-5), 141.31 (C-3), 149.70 (C-1).

Selective NOE: The irradiation of multiplet at 3.828 ppm of major isomer at 243 K produce 0.10% NOE of signal at 7.334 ppm (H-5), while the irradiation of multiplet at 3.740 ppm produce 0.12% NOE of signal at 5.883 ppm (OH). This result confirms that major rotamer is *anti* (CH is closer to H-5) and minor rotamer is *syn* (CH is closer to OH).

^{13}C-NMR (150.9 MHz, T=253 K, $CDCl_3$): δ = 111.23 (d, *J*=7.3 Hz), 111.85 (dd, *J*=3.1, 7.0 Hz), 115.00 (d, *J*=8.1 Hz), 115.49 (dd, *J*=3.4, 7.7 Hz).

^{13}C-NMR (150.9 MHz, T=253 K, $CDCl_3$): δ = 111.23 (d, *J*=7.3 Hz, C-2, *anti*), 111.85 (d, *J*=7.0 Hz, C-4, *syn*), 115.00 (d, *J*=7.8 Hz, C-2, *syn*), 115.49 (d, *J*=7.7 Hz, C-4, *anti*). Major = *anti*; Minor = *syn*

Table S2. GIAO/B3LYP/6-311++G(d,p) calculations of coupling constants of two rotamers of **2**

Rotamer	$^{3}J_{(C\text{-}2,CH)}$	$^{3}J_{(C\text{-}4,CH)}$	$^{2}J_{(C\text{-}4,H\text{-}5)}$
Anti	6.8	8.3	-3.1
Syn	8.4	6.3	-3.1
Anti (exp. 253 K)	7.3	7.7	-3.4
Syn (exp. 253 K)	7.8	7.0	-3.1

Iodination of carvacrol

Five grams (33 mmol) of carvacrol was dissolved in a solution of 1.2 g (30 mmol) of sodium hydroxide in 20 mL of water. While stirring on a magnetic stirrer, a solution containing 6.0 g of iodine (23.6 mmol) and 9.0 g (54 mmol) of potassium iodide dis-solved in 10 mL of water was added dropwise. The decolorization of the reaction mix-ture was considered to be the end of the reaction. The reaction mixture was neutralized with hydrochloric acid (1:1, v/v) and extracted with diethyl ether, and the combined organic layers were washed with aqueous sodium thiosulfate solution (10%). The or-ganic layer was dried and concentrated in vacuo.

The obtained mixture (6.67 g) was initially fractionated via gradient dry-flash column chromatography (n-hexane–diethyl ether mixtures of increasing polarity (v/v)) on sili-ca gel. The fractions were pooled based on thin-layer chromatography (1% diethyl ether in n-hexane (v/v)). Based on GC–MS analysis, fraction I (pure n-hexane was used as the eluent) contained a mixture of 3-isopropyl-2,4-diiodo-6-methylphenol and 3-isopropyl-2-iodo-6-methylphenol (o-iodocarvacrol), so it was further separated on Sephadex LH-20. After several re-chromatographies on Sephadex LH-20 with a mix-ture of MeOH and CHCl3 at a 1:1 (v/v) ratio, pure compounds were obtained, which were further used for the synthesis of diiodocarvacrol derivatives.

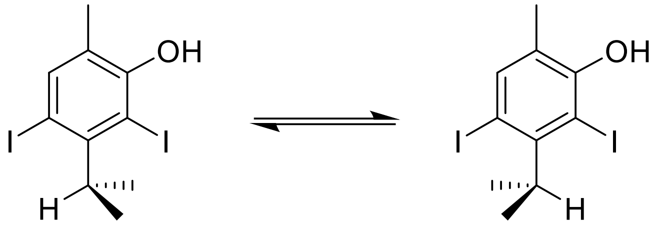

anti *syn*

2,4-diiodo-3-isopropyl-6-methylphenol (3,5-diiodocarvacrol) (3): colorless amorphous solid, yield 51%; ^{1}H NMR (400 MHz, 298 K, $CDCl_3$, ppm): mixture of *syn* and *anti* rotamers around *i*-Pr-Ar bond; rotamer 1: δ = 1.39 (d, *J*=7.3 Hz, 6H; CH_3), 2.222 (bs, 3H; CH_3), 3.66 (septet, *J*=7.3 Hz, 1H; CH), 5.88 (s, 1H; OH), 7.62 (s, 1H; H-5); rotamer 2: δ = 1.40 (d, *J*=7.3 Hz, 6H; CH_3), 2.217 (bs, 3H; CH_3), 3.61 (septet, *J*=7.3 Hz, 1H; CH), 5.63 (s, 1H; OH), 7.63 (s, 1H; H-5); ^{13}C NMR (100.6 MHz, 298 K, $CDCl_3$, ppm): mixture of *syn* and *anti* rotamers around *i*-Pr-Ar bond; rotamer 1: δ = 16.84 (CH_3), 19.88 (CH_3), 43.42 (CH), 86.77 (C-2), 124.53 (C-6), 92.41 (C-4), 142.17 (C-5), 146.40 (C-3), 153.81 (C-1); rotamer 2: δ = 16.54 (CH_3), 19.93 (CH_3), 43.55 (CH), 98.21 (C-2), 124.56 (C-6), 80.57 (C-4), 144.50 (C-5), 146.22 (C-3), 153.11 (C-1); FTIR (neat) ν_{max}/cm^{-1} 3450, 2955, 2922, 2869, 1584, 1434, 1344, 1203, 1120, 874, 645, 610, 602; UV/Vis (CH_3CN, nm): λ_{max}(log ε)=286 (2.51), 215 (3.65); EI-MS: *m*/*z* (%): 402 (100) [*M*]$^+$, 403 (11.0), 387 (40.5), 261 (8.0), 260 (67.0), 247 (9.8), 133 (10.2), 91 (9.5), 77 (13.9); HRMS (ESI): *m*/*z* calcd for ($C_{10}H_{12}I_2O$ + H^+)$^+$: 402.9050; found: 402.9052; elemental analysis calcd (%) for $C_{10}H_{12}I_2O$: C 29.88, H 3.01, O 3.98, I 63.13; found: C 30.00, H 3.07.

^{1}H-NMR (600 MHz, 253 K, $CDCl_3$): Mixture of *syn* and *anti* rotamers around *i*-Pr-Ar bond: 0.52 : 0.48. *Syn* major rotamer: δ = 1.40 (d, *J*=7.3 Hz, 6H, CH_3), 2.23 (s, 3H, CH_3), 3.61 (septet, *J*=7.3 Hz, 1H, CH), 5.72 (s, 1H, OH), 7.64 (s, 1H, H-5); *Anti* minor rotamer: δ = 1.40 (d, *J*=7.3 Hz, 6H, CH_3), 2.24 (s, 3H, CH_3), 3.65 (septet, *J*=7.3 Hz, 1H, CH), 5.98 (s, 1H, OH), 7.65 (s, 1H, H-5).

^{13}C-NMR (150.9 MHz, 253 K, $CDCl_3$): Mixture of *syn* and *anti* rotamers around *i*-Pr-Ar bond: 0.52 : 0.48. *Syn* major rotamer: 16.80 (CH_3), 19.78 (CH_3), 43.53 (CH), 80.60 (C-4), 98.34 (C-2), 124.49 (C-6), 144.30 (C-5), 145.83 (C-3), 152.88 (C-1); *Anti* minor rotamer: δ = 17.10 (CH_3), 19.72 (CH_3), 43.38 (CH), 86.82 (C-2), 92.49 (C-4), 124.44 (C-6), 141.97 (C-5), 146.01 (C-3), 153.55 (C-1).

Selective NOE: The irradiation of multiplet at 3.604 ppm of major rotamer at 243 K produce 0.18% NOE of signal at 5.738 ppm (OH), while the irradiation of multiplet at 3.642 ppm of minor rotamer produce 0.10% NOE of signal at 7.334 ppm (H-5). This result confirms that major rotamer is *syn* (CH is closer to OH) and minor rotamer is *anti* (CH is closer to H-5).

^{13}C-NMR (150.9 MHz, T=253 K, $CDCl_3$): δ = 80.61 (dd, *J*=1.7, 6.6 Hz), 86.82 (d, *J*=7.2 Hz), 92.50 (dd, *J*=1.9, 8.9 Hz), 98.34 (d, *J*=8.9 Hz).

^{13}C-NMR (150.9 MHz, T=253 K, $CDCl_3$): δ = 80.61 (d, *J*=6.6 Hz, C-4, *syn*), 86.82 (d, *J*=7.2 Hz, C-2, *anti*), 92.50 (d, *J*=8.9 Hz, C-4, *anti*), 98.34 (d, *J*=8.9 Hz, C-2, *syn*). Major = *syn*; Minor = *anti*

Table S3. GIAO/B3LYP/6-311++G(d,p) calculations of coupling constants of two rotamers of **3**

Rotamer	$^3J_{(C-2,CH)}$	$^3J_{(C-4,CH)}$	$^2J_{(C-4,H-5)}$
Anti	6.5	10.0	-1.8
Syn	10.3	5.8	-1.7
Anti (exp. 253 K)	6.6	8.9	-1.9
Syn (exp. 253 K)	8.9	7.2	-1.7

Etherification of compounds 2 **and** 3

Either phenol 2 or 3 (0.174 mmol) was added to a suspension of anhydrous potas-sium carbonate (48 mg, 0.350 mmol) in acetone (5 mL). Methyl iodide (25 mg, 0.178 mmol) or allyl bromide (22 mg, 0.178 mmol) was then added, and the mixture was stirred for 24 hours at room temperature. After that, acetone was evaporated under reduced pressure, and water was added. The resulting reaction mixture was extracted three times with diethyl ether. The combined extracts were dried and evaporated. The crude methylation product was purified via gradient silica gel column chromatog-raphy (eluent: n-hexane–diethyl ether (v/v)) to give the desired compounds **4**, **5**, **6**, and **7**.

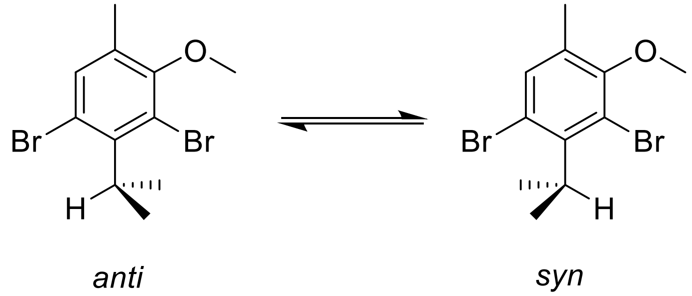

1,3-dibromo-2-isopropyl-4-methoxy-5-methylbenzene (methyl ether of 3,5-dibromocarvacrol) (4): colorless oil, yield 63%; ^{1}H NMR (400 MHz, 298 K, $CDCl_3$, ppm): mixture of *syn* and *anti* rotamers around *i*-Pr-Ar bond; rotamer 1: δ = 1.42 (m, 6H; CH_3), 2.26 (d, *J*=0.6 Hz; 3H; CH_3), 3.77 (s, 3H; CH_3), 3.95 (septet, *J*=7.3 Hz, 1H; CH), 7.33 (s, 1H; H-5); rotamer 2: δ = 1.42 (m, 6H; CH_3), 2.26 (d, *J*=0.6 Hz; 3H; CH_3), 3.77 (s, 3H; CH_3), 3.84/3.95 (septet, *J*=7.3 Hz, 1H; CH), 7.37 (s, 1H; H-5); ^{13}C NMR (100.6 MHz, 298 K, $CDCl_3$, ppm): mixture of *syn* and *anti* rotamers around *i*-Pr-Ar bond; rotamer 1; δ = 16.03 (CH_3), 19.49 (CH_3), 35.57 (CH), 59.95 (OCH_3), 119.16 (C-2), 120.70 (C-4),131.44 (C-6), 136.09 (C-5), 143.24 (C-3), 154.86 (C-1); rotamer 2: δ = 16.03 (CH_3), 19.49 (CH_3), 36.30 (CH), 59.95 (OCH_3), 116.91 (C-4), 122.59 (C-2), 131.44 (C-6), 134.22 (C-5), 143.24 (C-3), 155.71 (C-1); FTIR (neat) ν_{max}/cm^{-1} 2959, 2930, 1453, 1324, 1270, 1147, 940, 870, 732, 706, 652; UV/Vis (CH_3CN, nm): λ_{max}(log ε)=208.0 (4.63); EI-MS: *m*/*z* (%): 322 (82.6) [*M*]$^+$, 320 (41.7), 309 (48.5), 307 (100), 305 (50.0), 115.1 (26.9), 103.1 (17.7), 77 (17.9), 51 (9.9).

^{1}H-NMR (600 MHz, T=243 K, $CDCl_3$): Mixture of *syn* and *anti* rotamers around *i*-Pr-Ar bond: 0.53 : 0.47. *Syn* major rotamer: δ = 1.38 (d, *J*=7.3 Hz, 6H, CH_3), 2.26 (d, *J*=0.6 Hz, 3H, CH_3), 3.75 (s, 3H, OCH_3), 3.91 (septet, *J*=7.3 Hz, 1H, CH), 7.34 (d, *J*=0.6 Hz, 1H, H-5); *Anti* minor rotamer: δ = 1.40 (d, *J*=7.3 Hz, 6H, CH_3), 2.26 (d, *J*=0.6 Hz, 3H, CH_3), 3.75 (s, 3H, OCH_3), 3.80 (septet, *J*=7.3 Hz, 1H, CH), 7.39 (d, *J*=0.6 Hz, 1H, H-5).

^{13}C-NMR (150.9 MHz, T=243 K, $CDCl_3$): Mixture of *syn* and *anti* rotamers around *i*-Pr-Ar bond: 0.53 : 0.47. *Syn* major rotamer: δ = 16.23 (CH_3), 19.37 (CH_3), 35.59 (CH), 60.02 (OCH_3), 116.98 (C-4), 122.68 (C-2), 131.74 (C-6), 136.12 (C-5), 142.89 (C-3), 154.53 (C-1); *Anti* minor rotamer: δ = 16.34 (CH_3), 19.44 (CH_3), 36.32 (CH), 60.16 (OCH_3), 119.19 (C-2), 120.76 (C-4), 131.71 (C-6), 134.25 (C-5), 142.88 (C-3), 155.41 (C-1).

Selective NOE: The irradiation of multiplet at 3.916 ppm of major isomer at 243 K produce 0.04% NOE of signal at 7.342 ppm (H-5), while the irradiation of multiplet at 3.616 ppm produce 0.12% NOE of signal at 7.390 (H-5) ppm. This result confirms that minor rotamer is *anti* (CH is closer to H-5).

^{13}C-NMR (150.9 MHz, T=243 K, $CDCl_3$): δ = 116.98 (dd, *J*=3.1, 7.0 Hz), 119.19 (d, *J*=6.4 Hz), 120.76 (dd, *J*=3.1, 7.6 Hz), 122.68 (d, *J*=8.8 Hz).

^{13}C-NMR (150.9 MHz, T=243 K, $CDCl_3$): δ = 116.98 (d, *J*=7.1 Hz, C-4, *syn*), 119.19 (d, *J*=7.2 Hz, C-2, *anti*), 120.76 (d, *J*=7.7 Hz, C-4, *anti*), 122.68 (d, *J*=7.4 Hz, C-2, *syn*). Major = *syn*; Minor = *anti*

Table S4. GIAO/B3LYP/6-311++G(d,p) calculations of coupling constants of two rotamers of **4**

Rotamer	$^3J_{(C-2,CH)}$	$^3J_{(C-4,CH)}$	$^2J_{(C-4,H-5)}$
Anti	6.7	8.4	-3.0
Syn	8.0	6.4	-3.0
Anti (exp. 243 K)	7.2	7.7	-3.1
Syn (exp. 243 K)	8.8	7.1	-3.1

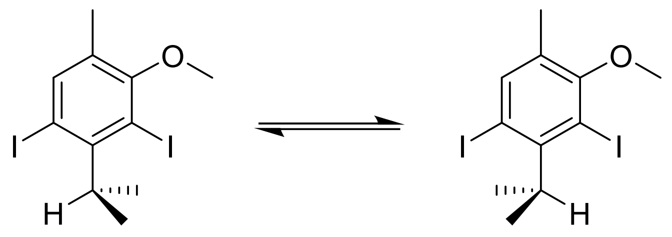

anti *syn*

1,3-diiodo-2-isopropyl-4-methoxy-5-methylbenzene (methyl ether of 3,5-diiodocarvacrol) (5): colorless oil, yield 21%; ^{1}H NMR (400 MHz, 298 K, $CDCl_3$, ppm): mixture of *syn* and *anti* rotamers around *i*-Pr-Ar bond; rotamer 1: δ = 1.43 (d, *J*=7.3 Hz, 6H; CH_3), 2.26 (s, 3H; CH_3), 3.63 (septet, *J*=7.3 Hz, 1H; CH), 3.74 (s, 3H; CH_3), 7.72 (s, 1H; H-5); rotamer 2: δ = 1.39 (d, *J*=7.3 Hz, 6H; CH_3), 2.26 (s, 3H; CH_3), 3.85 (septet, *J*=7.3 Hz, 1H; CH), 3.74 (s, 3H; CH_3), 7.72 (s, 1H; H-5); ^{13}C NMR (100.6 MHz, 298 K, $CDCl_3$, ppm): mixture of *syn* and *anti* rotamers around *i*-Pr-Ar bond; rotamer 1: δ = 16.59 (CH_3), 19.94 (CH_3), 43.91 (CH), 60.20 (OCH_3), 93.23 (C-2), 98.89 (C-4), 131.10 (C-6), 142.61 (C-5), 148.05 (C-3), 159.17 (C-1); rotamer 2: δ = 16.34 (CH_3), 19.85 (CH_3), 42.79 (CH), 59.99 (OCH_3), 86.34 (C-4), 104.62 (C-2), 130.91 (C-6), 145.17 (C-5), 147.96 (C-3), 158.33 (C-1); FTIR (neat) ν_{max}/cm^{-1} 2954, 2926, 2869, 1446, 1412, 1264, 1132, 1016, 870, 712, 679, 635, 600; UV/Vis (CH_3CN, nm): λ_{max}(log ε)=216.5 (4.38); EI-MS: *m*/*z* (%): 416 (100) [*M*]$^+$, 401 (34.9), 274 (45.7), 147 (15.6), 127 (11.7), 115 (18.9), 77 (12.9); HRMS (ESI): *m*/*z* calcd for ($C_{11}H_{14}I_2O$ + H^+)$^+$: 416.9207; found: 416.9206; elemental analysis calcd (%) for $C_{11}H_{14}I_2O$: C 31.76, H 3.39, O 3.85, I 61.01; found: C 31.70, H 3.31%.

^{1}H-NMR (600 MHz, T=253 K, $CDCl_3$): Mixture of *anti* and *syn* rotamers around *i*-Pr-Ar bond: 0.60 : 0.40. *Anti* major rotamer: δ = 1.39 (d, *J*=7.3 Hz, 6H, CH_3), 2.29 (s, 3H, CH_3), 3.76 (s, 3H, OCH_3), 3.83 (septet, *J*=7.3 Hz, 1H, CH), 7.75 (s, 1H, H-5); *Syn* minor rotamer: δ = 1.43 (d, *J*=7.3 Hz, 6H, CH_3), 2.29 (s, 3H, CH_3), 3.62 (septet, *J*=7.3 Hz, 1H, CH), 3.76 (s, 3H, OCH_3), 7.75 (s, 1H, H-5).

^{13}C-NMR (150.9 MHz, T=253 K, $CDCl_3$): Mixture of *anti* and *syn* rotamers around *i*-Pr-Ar bond: 0.60 : 0.40. *Anti* major rotamer: δ = 16.45 (CH_3), 19.77 (CH_3), 42.74 (CH), 60.04 (OCH_3), 86.57 (C-2), 104.86 (C-4), 131.20 (C-6), 145.04 (C-5), 147.54 (C-3), 157.88 (C-1); *Syn* minor rotamer: δ = 16.71 (CH_3), 19.68 (CH_3), 43.85 (CH), 60.24 (OCH_3), 93.37 (C-4), 98.87 (C-2), 131.00 (C-6), 142.49 (C-5), 147.63 (C-3), 158.71 (C-1).

Selective NOE: The H-5 signals of both rotamers are overlapping, which prevent their assignment by NOE.

^{13}C-NMR (150.9 MHz, T=243 K, $CDCl_3$): δ = 86.63 (dd, *J*=1.8, 6.8 Hz, C-2, *anti*), 93.42 (dd, *J*=2.0, 6.9 Hz, C-4, *syn*), 98.94 (dd, *J*=2.1, 8.9 Hz, C-2, *syn*), 104.93 (dd, *J*=2.0, 8.4 Hz, C-4, *anti*).

^{13}C-NMR (150.9 MHz, T=243 K, $CDCl_3$): δ = 86.63 (d, *J*=6.9 Hz, C-2, *anti*), 93.42 (d, *J*=7.0 Hz, C-4, *syn*), 98.94 (d, *J*=8.8 Hz, C-2, *syn*), 104.93 (d, *J*=8.4 Hz, C-4, *anti*). Major = *anti*; Minor = *syn*

Table S5. GIAO/B3LYP/6-311++G(d,p) calculations of coupling constants of two rotamers of **5**.

Rotamer	$^3J_{(C-2,CH)}$	$^3J_{(C-4,CH)}$	$^2J_{(C-4,H-5)}$	$^4J_{(C-2,H-5)}$
Anti	6.3	10.1	-3.0	-1.8
Syn	9.6	6.0	-3.0	-1.7
Anti (exp. 243 K)	6.9	8.4	-2.0	-1.8
Syn (exp. 243 K)	8.8	7.0	-2.1	-2.0

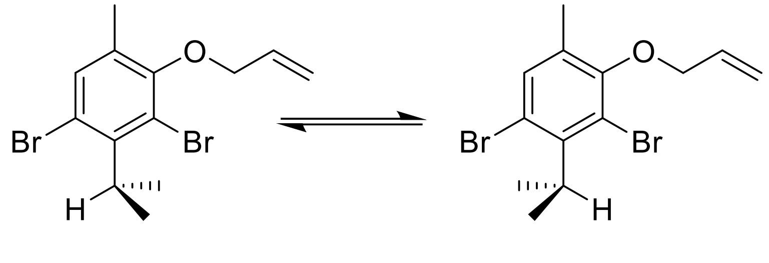

2-(allyloxy)-3,5-dibromo-4-isopropyl-1-methylbenzene (allyl ether of 3,5-dibromocarvacrol) (6): colorless oil, yield 67%; ^{1}H NMR (400 MHz, 298 K, $CDCl_3$, ppm): mixture of *syn* and *anti* rotamers around *i*-Pr-Ar bond: rotamer 1; δ = 1.42 (d, *J*=7.3 Hz, 6H; CH_3), 2.25 (s, 3H; CH_3), 3.83 (septet, *J*=7.3 Hz, 1H; CH), 4.38 (dt, *J*=1.4, 5.6 Hz, 2H; CH_2), 5.29 (ddd *J*=1.3, 2.7, 10.4 Hz, 1H; =CH), 5.45 (dq, *J*=1.6, 17.2 Hz, 1H; =CH), 6.13 (ddt, *J*=5.6, 10.5, 17.1 Hz, 1H; =CH), 7.38 (s, 1H; H-5); rotamer 2: 1.41 (d, *J*=7.3 Hz, 6H; CH_3), 2.25 (s, 3H; CH_3), 3.95 (septet, *J*=7.3 Hz, 1H; CH), 4.38 (dt, *J*=1.4, 5.6 Hz, 2H; CH_2), 5.29 (ddd, *J*=1.3, 2.7, 10.4 Hz, 1H; =CH), 5.45 (dq, *J*=1.6, 17.2 Hz, 1H; =CH), 6.13 (ddt, *J*=5.6, 10.5, 17.1 Hz, 1H; =CH), 7.33 (s, 1H; H-5); ^{13}C NMR (100.6 MHz, 298 K, $CDCl_3$, ppm): mixture of *syn* and *anti* rotamers around *i*-Pr-Ar bond; rotamer 1: δ = 16.33 (CH_3), 19.46 (CH_3), 36.28 (CH), 73.29 (CH_2), 120.74 (C-4), 117.90 (=CH_2), 119.34 (C-2), 136.03 (C-6), 133.30 (=CH), 134.15 (C-5), 143.18 (C-3), 154.64 (C-1); rotamer 2: δ = 16.26 (CH_3), 19.46 (CH_3), 35.59 (CH), 73.29 (CH_2), 117.90 (=CH_2), 122.81 (C-2), 116.93 (C-4), 131.67 (C-6), 133.30 (=CH), 136.03 (C-5), 143.18 (C-3), 153.76 (C-1); FTIR (neat) ν_{max}/cm^{-1} 2960, 2928, 2873, 1706, 1647, 1584, 1444, 1323, 1330, 1267, 1150, 1010, 989, 921, 732, 710, 689; UV/Vis (CH_3CN, nm): λ_{max}(log ε)=216.5 (4.38); EI-MS: *m*/*z* (%): 348 (42.4) [*M*]$^+$, 307 (79.4), 267 (28.2), 147 (51.9), 119 (56.0), 77 (52.0), 41 (100); HRMS (ESI): *m*/*z* calcd for ($C_{13}H_{16}Br_2O$ + H^+)$^+$: 346.9641; found: 346.9645; elemental analysis calcd (%) for $C_{13}H_{16}Br_2O$: C 44.86, H 4.36, O 4.60, Br 45.91; found: C 44.70, H 4.41.

^{1}H-NMR (600 MHz, T=243 K, $CDCl_3$): Mixture of *syn* and *anti* rotamers around *i*-Pr-Ar bond: 0.53 : 0.47. *Syn* major rotamer: δ = 1.41 (d, *J*=7.3 Hz, 6H, CH_3), 2.27 (d, *J*=0.5 Hz, 3H, CH_3), 3.94 (septet, *J*=7.3 Hz, 1H, CH), 4.38 (d, *J*=5.7 Hz, 2H, CH_2), 5.34 (qd, *J*=1.3, 10.4 Hz, 1H, =CH), 5.50 (md, *J*=1.6, 17.2 Hz, 1H, =CH), 6.16 (tdd, *J*=5.5, 11.0, 17.0 Hz, 1H, =CH), 7.36 (d, *J*=0.3 Hz, 1H, H-5); *Anti* minor rotamer: 1.43 (d, *J*=7.3 Hz, 6H, CH_3), 2.28 (d, *J*=0.4 Hz, 3H, CH_3), 3.82 (septet, *J*=7.3 Hz, 1H, CH), 4.38 (d, *J*=5.7 Hz, 2H, CH_2), 5.34 (qd, *J*=1.3, 10.4 Hz, 1H, =CH), 5.50 (md, *J*=1.6, 17.2 Hz, 1H, =CH), 6.16 (tdd, *J*=5.5, 11.0, 17.0 Hz, 1H, =CH), 7.41 (d, *J*=0.6 Hz, 1H, H-5).

^{13}C-NMR (150.9 MHz, T=243 K, $CDCl_3$): Mixture of *syn* and *anti* rotamers around *i*-Pr-Ar bond: 0.53 : 0.47. *Syn* major rotamer: δ = 16.48 (CH_3), 19.44 (CH_3), 35.66 (CH), 73.23 (CH_2), 117.10 (C-4), 118.37 (=CH_2), 122.92 (C-2), 131.91 (C-6), 133.20 (=CH), 136.09 (C-5), 142.93 (C-3), 153.55 (C-1); *Anti* minor rotamer: δ = 16.58 (CH_3), 19.50 (CH_3), 36.35 (CH), 73.31 (CH_2), 118.27 (=CH_2), 119.41 (C-2), 120.90 (C-4), 131.88 (C-6), 133.22 (=CH), 134.22 (C-5), 142.96 (C-3), 154.43 (C-1).

Selective NOE: The irradiation of multiplet at 3.935 ppm of major rotamer at 243 K produce 0.02% NOE of signal at 7.404 ppm, while the irradiation of multiplet at 3.818 ppm produce 0.12% NOE of signal at 7.358 ppm. This result confirms that minor rotamer is *anti* (CH is closer to H-5).

^{13}C-NMR (150.9 MHz, T=243 K, $CDCl_3$): δ = 117.10 (dd, *J*=3.1, 7.1 Hz), 119.43 (d, *J*=5.3 Hz), 120.90 (dd, *J*=3.1, 7.8 Hz), 122.92 (d, *J*=7.5 Hz).

^{13}C-NMR (150.9 MHz, T=243 K, $CDCl_3$): δ = 117.10 (d, *J*=7.1 Hz, C-4, *syn*), 119.42 (d, *J*=7.3 Hz, C-2, *anti*), 120.91 (d, *J*=7.7 Hz, C-4, *anti*), 122.92 (d, *J*=7.5 Hz, C-2, *syn*). Major = *syn*; Minor = *anti*

Table S6. GIAO/B3LYP/6-311++G(d,p) calculations of coupling constants of two rotamers of **6**.

Rotamer	$^3J_{(C\text{-}2,CH)}$	$^3J_{(C\text{-}4,CH)}$	$^2J_{(C\text{-}4,H\text{-}5)}$
Anti	6.7	8.4	-3.0
Syn	8.0	6.4	-3.0
Anti (exp. 243 K)	7.3	7.7	-3.1
Syn (exp. 243 K)	7.5	7.1	-3.1

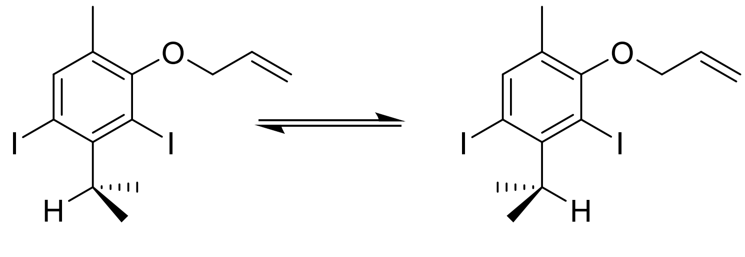

2-(allyloxy)-3,5-diiodo-4-isopropyl-1-methylbenzene (allyl ether of 3,5-diiodocarvacrol) (7): colorless oil, yield 67%; ^{1}H NMR (400 MHz, 298 K, $CDCl_3$, ppm): mixture of *syn* and *anti* rotamers around *i*-Pr-Ar bond: rotamer 1: δ = 1.43 (d, *J*=7.3 Hz, 6H; CH_3), 2.24 (s, 3H; CH_3), 3.64 (septet, *J*=7.3 Hz, 1H; CH), 4.35 (dt, *J*=1.5, 5.6 Hz, 2H; CH_2), 5.29 (dq *J*=1.4, 10.4 Hz, 1H; =CH), 5.47 (dq, *J*=1.6, 17.2 Hz, 1H; =CH), 6.15 (ddt, *J*=5.6, 10.5, 17.2 Hz, 1H; =CH), 7.72 (bs, 1H; H-5); rotamer 2: 1.39 (d, *J*=7.3 Hz, 6H; CH_3), 2.24 (s, 3H; CH_3), 3.85 (septet, *J*=7.3 Hz, 1H; CH), 4.35 (dt, *J*=1.5, 5.6 Hz, 2H; CH_2), 5.29 (dq *J*=1.6, 10.4 Hz, 1H; =CH), 5.47 (dq, *J*=1.6, 17.2 Hz, 1H; =CH), 6.15 (ddt, *J*=5.6, 10.5, 17.2 Hz, 1H; =CH), 7.72 (bs, 1H; H-5); ^{13}C NMR (100.6 MHz, 298 K, $CDCl_3$, ppm): mixture of *syn* and *anti* rotamers around *i*-Pr-Ar bond; rotamer 1; δ = 16.50 (CH_3),

19.82 (CH_3), 43.78 (CH), 73.22 (CH_2), 98.50 (C-4), 117.94 (=CH_2), 93.38 (C-2), 130.98 (C-6), 133.21 (=CH), 142.42 (C-5), 147.93 (C-3), 157.08 (C-1); rotamer 2: δ = 16.27 (CH_3), 19.72 (CH_3), 42.70 (CH), 73.12 (CH_2), 117.94 (=CH_2), 104.81 (C-2), 86.23 (C-4), 131.15 (C-6), 133.21 (=CH), 144.98 (C-5), 147.84 (C-3), 157.90 (C-1); FTIR (neat) ν_{max}/cm^{-1} 2956, 2923, 2869, 1647, 1434, 1416, 1263, 1136, 987, 920, 870, 684; UV/Vis (CH_3CN, nm): λ_{max}(log ε)=216.50 (3.72); EI-MS: *m/z* (%): 442 (67.0) $[M]^+$, 401 (100), 274 (58.3), 188 (46.9), 147 (59.2), 91 (53.8), 41 (64.1);); HRMS (ESI): *m/z* calcd for $(C_{13}H_{16}I_2O + H^+)^+$: 442.9363; found: 442.9361; elemental analysis calcd (%) for $C_{13}H_{16}I_2O$: C 35.32, H 3.65, O 3.62, I 57.41; found: C 35.28, H 3.60%.

^{1}H-NMR (600 MHz, T=243 K, $CDCl_3$): Mixture of *syn* and *anti* rotamers around *i*-Pr-Ar bond: 0.53 : 0.47. *Syn* major rotamer: δ =1.39 (d, *J*=7.3 Hz, 6H, CH_3), 2.27 (s, 3H, CH_3), 3.83 (septet, *J*=7.3 Hz, 1H, CH), 4.35 (td, *J*=1.3, 5.5 Hz, 2H, CH_2), 5.35 (qd, *J*=1.3, 10.4 Hz, 1H, =CH), 5.53 (md , *J*=1.5, 17.3 Hz, 1H, =CH), 6.18 (tdd, *J*=5.5, 10.4, 17.3 Hz, 1H, =CH), 7.75 (s, 1H, H-5); *Anti* minor rotamer: δ = 1.43 (d, *J*=7.3 Hz, 6H, CH_3), 2.27 (s, 3H, CH_3), 3.62 (septet, *J*=7.3 Hz, 1H, CH), 4.34 (td, *J*=1.3, 5.5 Hz, 2H, CH_2), 5.35 (qd, *J*=1.3, 10.4 Hz, 1H, =CH), 5.53 (md, *J*=1.5, 17.3 Hz, 1H, =CH), 6.18 (tdd, *J*=5.5, 10.4, 17.3 Hz, 1H, =CH), 7.75 (s, 1H, H-5).

^{13}C-NMR (150.9 MHz, T=243 K, $CDCl_3$): Mixture of *syn* and *anti* rotamers around *i*-Pr-Ar bond: 0.53 : 0.47. *Syn* major rotamer: δ = 16.54 (CH_3), 19.69 (CH_3), 42.83 (CH), 73.08 (CH_2), 86.71 (C-4), 105.23 (C-2), 118.57 (=CH), 131.45 (C-6), 133.06 (=CH_2), 145.00 (C-5), 147.50 (C-3), 156.79 (C-1); *Anti* minor rotamer: δ = 16.78 (CH_3), 19.78 (CH_3), 43.89 (CH), 73.17 (CH_2), 93.67 (C-2), 99.06 (C-4), 118.48 (=CH), 131.24 (C-6), 133.02 (=CH_2), 142.44 (C-5), 147.61 (C-3), 157.60 (C-1).

Selective NOE: The irradiation of multiplet at 3.827 ppm of major rotamer at 243 K produces 0.04% NOE of signal at 7.753 ppm, while the irradiation of multiplet at 3.619 ppm produces 0.14% NOE of signal at 7.751 ppm. This result confirms that the minor rotamer is *anti* (CH is closer to H-5).

^{13}C-NMR (150.9 MHz, T=243 K, $CDCl_3$): δ = 86.71 (dd, *J*=1.3, 6.7 Hz, C-4), 93.67 (d, *J*=7.0 Hz, C-2), 99.06 (dd, *J*=1.6, 8.9 Hz, C-4), 105.23 (d, *J*=8.2 Hz, C-2).

^{13}C-NMR (150.9 MHz, T=243 K, $CDCl_3$): δ = 86.71 (d, *J*=6.8 Hz, C-4, *syn*), 93.67 (d, *J*=7.1 Hz, C-2, *anti*), 99.06 (d, *J*=8.8 Hz, C-4, *anti*), 105.23 (d, *J*=8.4 Hz, C-2, *syn*). Major = *syn*; Minor = *anti*

Table S7. GIAO/B3LYP/6-311++G(d,p) calculations of coupling constants of two rotamers of **7**.

Rotamer	$^3J_{(C-2,CH)}$	$^3J_{(C-4,CH)}$	$^2J_{(C-4,H-5)}$
Anti	6.3	10.1	-1.8
Syn	9.6	6.0	-1.7
Anti (exp. 243 K)	7.1	8.8	-1.6
Syn (exp. 243 K)	8.4	6.8	-1.3

Esterification of compounds 2 **and** 3

A solution containing phenols **2** or **3** (0.075 mmol), 4-(dimethylamino)pyridine (DMAP, 1.2 mg, 0.01 mmol), N,N′-dicyclohexylcarbodiimide (DCC, 16.9 mg, 0.082 mmol), and either propanoic acid (6 mg,

0.082 mmol) or 2-methylpropanoic acid (7.2 mg, 0.082 mmol) in dry CH2Cl2 (10 mL) was stirred overnight at room temperature. For the workup, silica gel (5 g) was added to the resulting suspension. Dichloromethane was then removed under vacuum, and the resulting residue was subjected to purification using gradient silica gel column chromatography (eluent: n-hexane–diethyl ether (v/v)), yielding the desired esters **8**, **9**, **10**, and **11**.

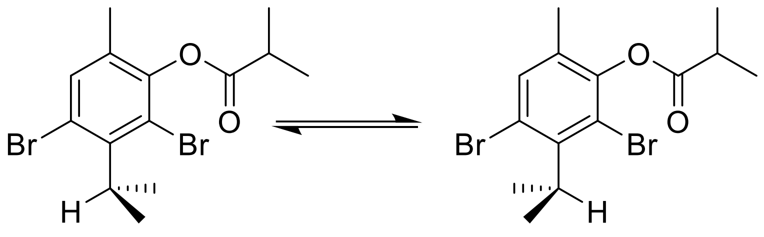

anti *syn*

2,4-dibromo-3-isopropyl-6-methylphenyl isobutyrate (3,5-dibromocarvacrol isobutyrate) (8): colorless oil, yield 82%; ^{1}H NMR (400 MHz, 298 K, $CDCl_3$, ppm): mixture of *syn* and *anti* rotamers around *i*-Pr-Ar bond; rotamer 1: δ = 1.38 (d, *J*=7.0 Hz, 6H; CH_3), δ = 1.42 (d, *J*=7.2 Hz, 6H; CH_3), 2.11 (s, 3H; CH_3), 2.90 (septet, *J*=7.0 Hz, 1H; CH), 3.87 (septet, *J*=7.2 Hz, 1H; CH), 7.43 (s, 1H; H-5); rotamer 2: δ = 1.38 (d, *J*=7.0 Hz, 6H; CH_3), δ = 1.42 (d, *J*=7.2 Hz, 6H; CH_3), 2.12 (s, 3H; CH_3), 2.90 (septet, *J*=7.0 Hz, 1H; CH), 3.91 (septet, *J*=7.2 Hz, 1H; CH), 7.43 (s, 1H; H-5); ^{13}C NMR (100.6 MHz, 298 K, $CDCl_3$, ppm): mixture of *syn* and *anti* rotamers around *i*-Pr-Ar bond; rotamer 1: δ = 16.32 (CH_3), 19.00 (CH_3), 19.41 (CH_3), 34.24 (CH), 36.25 (CH), 117.87 (C-2), 122.76 (C-4), 130.94 (C-6), 134.00 (C-5), 143.30 (C-3), 147.27 (C-1), 173.79 (C).; rotamer 2: δ = 16.26 (CH_3), 19.00 (CH_3), 19.41 (CH_3), 34.24 (CH), 35.69 (CH), 121.38 (C-2), 118.96 (C-4), 130.94 (C-6), 135.84 (C-5), 143.30 (C-3), 146.37 (C-1), 173.79 (C); FTIR (neat) ν_{max}/cm^{-1} 2962, 2930, 2873, 1755, 1440, 1231, 1118, 1039, 910, 707; UV/Vis (CH_3CN, nm): λ_{max}(log ε)=206.5 (4.78); EI-MS: *m/z* (%): 378 (2.4) [*M*]$^+$, 308 (45.6), 292 (20.9), 103 (9.1), 71 (67.9), 43 (100); HRMS (ESI): *m/z* calcd for ($C_{14}H_{18}Br_2O_2$ + H^+)$^+$: 376.9746; found: 376.9748; elemental analysis calcd (%) for $C_{14}H_{18}Br_2O_2$: C 44.47, H 4.80, O 8.46, Br 42.27; found: C 44.38, H 4.89.

^{1}H-NMR (600 MHz, T=238 K, $CDCl_3$): Mixture of *syn* and *anti* rotamers around *i*-Pr-Ar bond: 0.52 : 0.48. *Syn* major rotamer: δ = 1.39 (d, *J*=7.1 Hz, 9H, CH_3), 1.39 (d, *J*=7.2 Hz, 3H, CH_3), 2.13 (d, *J*=0.4 Hz, 3H, CH_3), 2.93 (septet, *J*=7.0 Hz, 1H, CH), 3.90 (septet, *J*=7.2 Hz, 1H, CH), 7.41 (d, *J*=0.4 Hz, 1H, H-5); *Anti* minor rotamer: δ = 1.40 (d, *J*=7.0 Hz, 3H, CH_3), 1.41 (d, *J*=7.2 Hz, 3H, CH_3), 1.42 (d, *J*=7.5 Hz, 3H, CH_3), 1.42 (d, *J*=7.3 Hz, 3H, CH_3), 2.13 (d, *J*=0.4 Hz, 3H, CH_3), 2.94 (septet, *J*=7.0 Hz, 1H, CH), 3.85 (septet, *J*=7.2 Hz, 1H, CH), 7.46 (d, *J*=0.4 Hz, 1H, H-5).

^{13}C-NMR (150.9 MHz, T=238 K, $CDCl_3$): Mixture of *syn* and *anti* rotamers around *i*-Pr-Ar bond: 0.52 : 0.48. *Syn* major rotamer: δ = 16.61 ($ArCH_3$), 19.16 (CH_3), 19.32 (CH_3), 34.35 (CH), 35.76 (CH), 119.24 (C-4), 121.37 (C-2), 131.06 (C-6), 135.97 (C-5), 143.06 (C-3), 146.15 (C-1), 174.52 (C=O); *Anti* minor rotamer: δ = 16.72 ($ArCH_3$), 19.37 (CH_3), 19.40 (CH_3), 19.48 (CH_3), 19.50 (CH_3), 34.34 (CH), 36.32 (CH), 117.82 (C-2), 123.05 (C-4), 131.04 (C-6), 134.14 (C-5), 143.15 (C-3), 147.05 (C-1), 174.50 (C=O).

Selective NOE: The irradiation of multiplet at 3.901 ppm of major rotamer at 243 K produce 0.05% NOE of signal at 7.411 ppm, while the irradiation of multiplet at 3.854 ppm produce 0.17% NOE of signal at 7.461 ppm. This result confirms that minor rotamer is *anti* (CH is closer to H-5).

^{13}C-NMR (150.9 MHz, T=238 K, $CDCl_3$): δ = 117.83 (d, *J*=6.9 Hz, C-2, *anti*), 119.25 (dd, *J*=3.2, 7.1 Hz, C-4, *syn*), 121.38 (d, *J*=8.6 Hz, C-2, *syn*), 123.05 (dd, *J*=3.2, 7.8 Hz, C-4, *anti*)

^{13}C-NMR (150.9 MHz, T=238 K, $CDCl_3$): δ = 117.83 (d, *J*=7.4 Hz), 119.24 (d, *J*=7.1 Hz), 121.38 (d, *J*=7.6 Hz), 123.05 (d, *J*=7.8 Hz). Major = *syn*; Minor = *anti*

Table S8. GIAO/B3LYP/6-311++G(d,p) calculations of coupling constants of two rotamers of **8**.

Rotamer	$^3J_{(C-2,CH)}$	$^3J_{(C-4,CH)}$	$^2J_{(C-4,H-5)}$
Anti	6.8	8.4	-3.0
Syn	8.2	6.5	-3.0
Anti (exp. 238 K)	7.4	7.8	-3.2
Syn (exp. 238 K)	7.6	7.1	-3.2

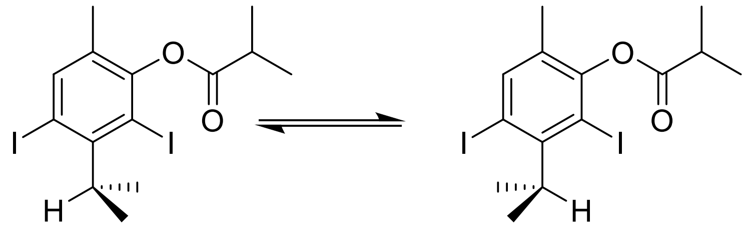

anti *syn*

2,4-diiodo-3-isopropyl-6-methylphenyl isobutyrate (3,5-diiodocarvacrol isobutyrate) (9): colorless oil, yield 96%; ^{1}H NMR (400 MHz, 298 K, $CDCl_3$, ppm): mixture of *syn* and *anti* rotamers around *i*-Pr-Ar bond; rotamer 1: δ = 1.38 (d, *J*=7.0 Hz, 6H; CH_3), δ = 1.42 (d, *J*=7.2 Hz, 6H; CH_3), 2.08 (bs, 3H; CH_3), 2.92 (septet, *J*=6.9 Hz, 1H; CH), 3.67 (septet, *J*=7.2 Hz, 1H; CH), 7.766 (s, 1H; H-5); rotamer 2: δ = 1.38 (d, *J*=7.0 Hz, 6H; CH_3), δ = 1.39 (d, *J*=7.2 Hz, 6H; CH_3), 2.08 (bs, 3H; CH_3), 2.92 (septet, *J*=6.9 Hz, 1H; CH), 3.80 (septet, *J*=7.2 Hz, 1H; CH), 7.772 (s, 1H; H-5); ^{13}C NMR (100.6 MHz, 298 K, $CDCl_3$, ppm): mixture of *syn* and *anti* rotamers around *i*-Pr-Ar bond; rotamer 1: δ = 16.62 (CH_3), 18.98 (CH_3), 19.69 (CH_3), 34.47 (CH), 43.69 (CH), 91.47 (C-2), 100.64 (C-4), 130.52 (C-6), 144.61 (C-5), 147.99 (C-3), 150.89 (C-1), 173.65 (C=O); rotamer 2: δ = 16.38 (CH_3), 18.98 (CH_3), 19.65 (CH_3), 34.47 (CH), 42.73 (CH), 103.04 (C-2), 88.34 (C-4), 130.65 (C-6), 142.06 (C-5), 147.85 (C-3), 149.97 (C-1), 173.62 (C=O); FTIR (neat) ν_{max}/cm^{-1} 2958, 2927, 2870, 1750, 1453, 1351, 1134, 873, 733, 629, 611; UV/Vis (CH_3CN, nm): λ_{max}(log ε)=214.0 (4.26), 196.5 (4.14); EI-MS: *m*/*z* (%): 472 (7.3) [*M*]$^+$, 402 (100), 386 (16.8), 260 (14.1), 71 (39.5), 43 (65.4); HRMS (ESI): *m*/*z* calcd for $(C_{14}H_{18}I_2O_2 + H^+)^+$: 472.9469; found: 472.9472; elemental analysis calcd (%) for $C_{14}H_{18}I_2O_2$: C 35.62, H 3.84, O 6.78, I 53.76; found: C 35.55, H 3.72.

^{1}H-NMR (600 MHz, T=238 K, $CDCl_3$): Mixture of *syn* and *anti* rotamers around *i*-Pr-Ar bond: 0.56 : 0.44. *Syn* major rotamer: δ = 1.39 (d, *J*=7.2 Hz, 6H, CH_3), 1.40 (*J*=7.9 Hz, 3H, CH_3), 1.41 (d, *J*=6.6 Hz, 3H, CH_3), 2.11 (s, 3H, CH_3), 2.95 (septet, *J*=7.2 Hz, 1H, CH), 3.78 (septet, *J*=7.3 Hz, 1H, CH), 7.80 (s, 1H, H-5); *Anti* minor rotamer: δ = 1.38 (d, *J*=7.0 Hz, 3H, CH_3), 1.41 (d, *J*=7.2 Hz, 3H, CH_3), 1.42 (d, *J*=7.0 Hz, 3H, CH_3), 1.42 (d, *J*=7.3 Hz, 3H, CH_3), 2.11 (s, 3H, CH_3), 2.96 (septet, *J*=7.2 Hz, 1H, CH), 3.65 (septet, *J*=7.3 Hz, 1H, CH), 7.79 (s, 1H, H-5).

^{13}C-NMR (150.9 MHz, T=238 K, $CDCl_3$): Mixture of *syn* and *anti* rotamers around *i*-Pr-Ar bond: 0.56 : 0.44. *Syn* major rotamer: δ = 16.75 ($ArCH_3$), 19.03 (CH_3), 19.04 (CH_3), 19.62 (CH_3), 19.69 (CH_3), 34.63 (CH), 42.91 (CH), 88.94 (C-4), 103.33 (C-2), 130.84 (C-6), 144.73 (C-5), 147.66 (C-3), 149.82 (C-1), 174.42 (C=O); *Anti* minor rotamer: δ = 17.00 ($ArCH_3$), 19.45 (CH_3), 19.49 (CH_3), 19.64 (CH_3), 19.75 (CH_3), 34.61 (CH), 43.87 (CH), 91.65 (C-2), 101.32 (C-4), 130.70 (C-6), 142.18 (C-5), 147.81 (C-3), 150.74 (C-1), 174.46 (C=O).

Selective NOE: The irradiation of multiplet at 3.786 ppm of major rotamer at 243 K produce 0.07% NOE of signal at 7.797 ppm, while the irradiation of multiplet at 3.656 ppm produce 0.16% NOE of signal at 7.793 ppm. This result confirms that minor rotamer is *anti* (CH is closer to H-5).

^{13}C-NMR (150.9 MHz, T=238 K, $CDCl_3$): δ = 88.94 (dd, *J*=1.9, 6.7 Hz), 91.65 (d, *J*=8.2 Hz), 101.32 (dd, *J*=1.6, 9.2 Hz), 103.34 (d, *J*=8.4 Hz).

^{13}C-NMR (150.9 MHz, T=238 K, $CDCl_3$): δ = 88.94 (d, *J*=6.8 Hz, C4, *syn*), 91.65 (d, *J*=7.2 Hz, C-2, *anti*), 101.32 (d, *J*=8.9 Hz, C-4, *anti*), 103.33 (d, *J*=8.7 Hz, C-2, *syn*). Major = *syn*; Minor = *anti*

Table S9. GIAO/B3LYP/6-311++G(d,p) calculations of coupling constants of two rotamers of **9**.

Rotamer	$^3J_{(C\text{-}2,CH)}$	$^3J_{(C\text{-}4,CH)}$	$^2J_{(C\text{-}4,H\text{-}5)}$
Anti	6.4	10.1	-1.7
Syn	9.8	6.0	-1.6
Anti (exp. 238 K)	7.2	8.9	-1.6
Syn (exp. 238 K)	8.7	6.8	-1.9

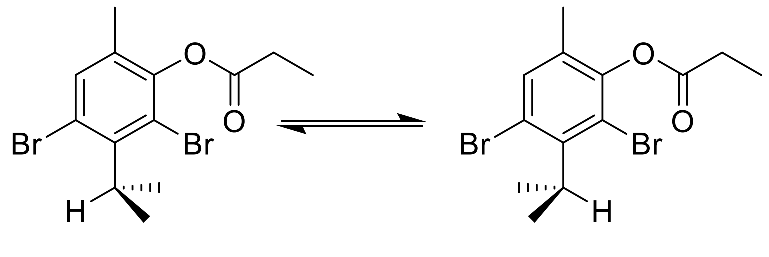

2,4-dibromo-3-isopropyl-6-methylphenyl propionate (3,5-dibromocarvacrol propionate) (10): colorless oil, yield 92%; ^{1}H NMR (400 MHz, 298 K, $CDCl_3$, ppm): mixture of *syn* and *anti* rotamers around *i*-Pr-Ar bond; rotamer 1: δ = 1.32 (t, *J*=7.6 Hz, 3H; CH_3), δ = 1.42 (d, *J*=7.3 Hz, 6H; CH_3), 2.12 (s, 3H; CH_3), 2.67 (q,

J=7.6 Hz, 2H; CH_2), 3.87 (septet, J=7.2 Hz, 1H; CH), 7.44 (s, 1H; H-5); rotamer 2: δ = 1.32 (t, J=7.6 Hz, 3H; CH_3), δ = 1.42 (d, J=7.3 Hz, 6H; CH_3), 2.12 (s, 3H; CH_3), 2.67 (q, J=7.6 Hz, 2H; CH_2), 3.91 (septet, J=7.2 Hz, 1H; CH), 7.39 (s, 1H; H-5); ^{13}C NMR (100.6 MHz, 298 K, $CDCl_3$, ppm): mixture of *syn* and *anti* rotamers around *i*-Pr-Ar bond; rotamer 1: δ = 9.13 (CH_3), 16.34 (CH_3), 19.41 (CH_3), 27.46 (CH_2), 36.23 (CH), 117.85 (C-2), 122.84 (C-4), 130.99 (C-6), 134.00 (C-5), 143.30 (C-3), 147.35 (C-1), 171.37 (C=O); rotamer 2: δ = 9.13 (CH_3), 16.29 (CH_3), 19.41 (CH_3), 27.46 (CH_2), 35.72 (CH), 121.37 (C-2), 119.02 (C-4), 130.99 (C-6), 135.85 (C-5), 143.30 (C-3), 146.44 (C-1), 171.37 (C=O); FTIR (neat) ν_{max}/cm^{-1} 2960, 2928, 2874, 1453, 1394, 1351, 1221, 1134, 1006, 733, 629; UV/Vis (CH_3CN, nm): λ_{max}(log ε)=291.0 (3.1), 206 (4.37); EI-MS: m/z (%): 364 (2.9) $[M]^+$, 308 (100), 292 (55.6), 212 (6.9), 103 (12.6), 77 (16.1), 57 (64.7); HRMS (ESI): m/z calcd for $(C_{13}H_{16}Br_2O_2 + H^+)^+$: 362.9590; found: 362.9588, elemental analysis calcd (%) for $C_{13}H_{16}Br_2O_2$: C 42.89, H 4.43, O 8.79, Br 43.89; found: C 42.81, H 4.52.

^{1}H-NMR (600 MHz, T=238 K, $CDCl_3$): Mixture of *syn* and *anti* rotamers around *i*-Pr-Ar bond: 0.52 : 0.48. *Syn* major rotamer: δ = 1.32 (t, J=7.6 Hz, 3H, CH_3), 1.42 (d, J=7.4 Hz, 6H. CH_3), 2.15 (d, J=0.4 Hz, 3H, CH_3), 2.68-2.79 (m, 2H, CH_2), 3.89 (septet, J=7.3 Hz, 1H, CH), 7.42 (d, J=0.4 Hz, 1H, H-5); *Anti* minor rotamer: δ = 1.32 (t, J=7.6 Hz, 3H, CH_3), 1.40 (d, J=7.1 Hz, 3H, CH_3), 1.41 (d, J=7.5 Hz, 3H, CH_3), 2.15 (d, J=0.4 Hz, 3H. CH_3), 2.68-2.79 (m, 2H, CH_2), 3.85 (septet, J=7.3 Hz, 1H, CH), 7.47 (d, J=0.4 Hz, 1H, H-5).

^{13}C-NMR (150.9 MHz, T=238 K, $CDCl_3$): Mixture of *syn* and *anti* rotamers around *i*-Pr-Ar bond: 0.52 : 0.48. *Syn* major rotamer: δ = 9.38 (CH_3), 16.66 (CH_3), 19.39 (CH_3), 19.48 (CH_3), 27.61 (CH_2), 35.81 (CH), 119.33 (C-4), 121.37 (C-2), 131.12 (C-6), 135.97 (C-5), 143.06 (C-3), 146.22 (C-1), 172.16 (C=O); *Anti* minor rotamer: δ = 9.40 (CH_3), 16.77 (CH_3), 19.38 (CH_3), 19.50 (CH_3), 27.54 (CH_2), 36.31 (CH), 117.81 (C-2), 123.14 (C-4), 131.11 (C-6), 134.13 (C-5), 143.16 (C-3), 147.13 (C-1), 172.16 (C=O).

Selective NOE: The irradiation of multiplet at 3.898 ppm of major rotamer at 243 K produce 0.02% NOE of signal at 7.420 ppm, while the irradiation of multiplet at 3.854 ppm produce 0.15% NOE of signal at 7.471 ppm, This result confirms that minor rotamer is *anti* (CH is closer to H-5).

^{13}C-NMR (150.9 MHz, T=238 K, $CDCl_3$): δ = 117.81 (dd, J=7.4, 1.9 Hz), 119.33 (dd, J=3.2, 7.1 Hz), 121.38 (dd, J=1.8, 7.6 Hz), 123.14 (dd, J=3.2, 7.7 Hz).

^{13}C-NMR (150.9 MHz, T=238 K, $CDCl_3$): δ = 117.81 (d, J=7.4 Hz, C-2, *anti*), 119.33 (d, J=7.2 Hz, C-4, *syn*), 121.38 (d, J=7.6 Hz, C-2, *syn*), 123.14 (d, J=7.8 Hz, C-4, *anti*). Major = *syn*; Minor = *anti*

Table S10. GIAO/B3LYP/6-311++G(d,p) calculations of coupling constants of two rotamers of **10**.

Rotamer	$^3J_{(C-2,CH)}$	$^3J_{(C-4,CH)}$	$^2J_{(C-4,H-5)}$
Anti	6.8	8.4	-3.0
Syn	8.2	6.5	-3.0
Anti (exp. 238 K)	7.4	7.8	-3.2
Syn (exp. 238 K)	7.6	7.2	-3.2

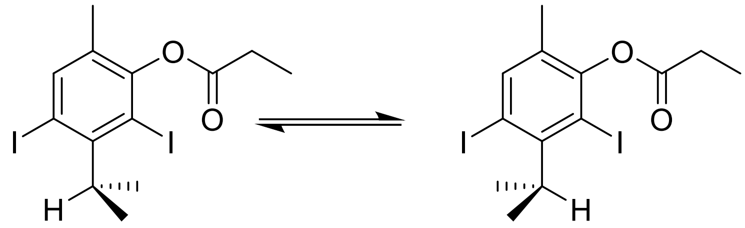

anti *syn*

2,4-diiodo-3-isopropyl-6-methylphenyl propionate (3,5-diiodocarvacrol propionate) (11): colorless oil, yield 88%; ^{1}H NMR (400 MHz, 298 K, $CDCl_3$, ppm): Mixture of *syn* and *anti* rotamers around *i*-Pr-Ar bond: rotamer 1: δ = 1.32 (t, *J*=7.6 Hz, 3H; CH_3), δ = 1.42 (d, *J*=7.3 Hz, 6H; CH_3), 2.10 (s, 3H; CH_3), 2.69 (q, *J*=7.6 Hz, 2H; CH_2), 3.67 (septet, *J*=7.2 Hz, 1H; CH), 7.776 (s, 1H; H-5); rotamer 2: δ = 1.32 (t, *J*=7.6 Hz, 3H; CH_3), δ = 1.40 (d, *J*=7.3 Hz, 6H; CH_3), 2.10 (s, 3H; CH_3), 2.68 (q, *J*=7.6 Hz, 2H; CH_2), 3.79 (septet, *J*=7.2 Hz, 1H; CH), 7.781 (s, 1H; H-5). ^{13}C NMR (100.6 MHz, 298 K, $CDCl_3$, ppm):Mixture of *syn* and *anti* rotamers around *i*-Pr-Ar bond: rotamer 1: δ = 9.22 (CH_3), 16.80 (CH_3), 19.86 (CH_3), 28.07 (CH_2), 43.87 (CH), 91.63 (C-2), 100.89 (C-4), 130.82 (C-6), 142.25 (C-5), 148.21 (C-3), 151.14 (C-1), 171.47 (C=O); rotamer 2: δ = 9.22 (CH_3), 16.56 (CH_3), 19.81 (CH_3), 28.14 (CH_2), 42.96 (CH), 103.21 (C-2), 88.59 (C-4), 130.94 (C-6), 144.81 (C-5), 148.06 (C-3), 150.21 (C-1), 171.43 (C=O); FTIR (neat) ν_{max}/cm^{-1} 2957, 2925, 2871, 1763, 1426, 1334, 1114, 1072, 881, 714, 692; UV/Vis (CH_3CN, nm): λ_{max}(log ε)=214.0 (4.35), 196 (4.25); EI-MS: *m/z* (%): 458 (5.5) $[M]^+$, 402 (100), 386 (24.0), 260 (18.2), 103 (7.7), 51 (30.0); HRMS (ESI): *m/z* calcd for $(C_{13}H_{16}I_2O_2 + H^+)^+$: 458.9312; found: 458.9313; elemental analysis calcd (%) for $C_{13}H_{16}I_2O_2$: C 34.09, H 3.52, O 6.99, I 55.41; found: C 34.21, H 3.59.

^{1}H-NMR (600 MHz, T=238 K, $CDCl_3$): Mixture of *syn* and *anti* rotamers around *i*-Pr-Ar bond: 0.54 : 0.46. *Syn* major rotamer: δ = 1.33 (t, *J*=7.6 Hz, 3H, CH_3), 1.38 (d, *J*=7.3 Hz, 3H, CH_3), 1.40 (d, *J*=7.3 Hz, 3H, CH_3), 2.12 (d, *J*=0.5 Hz, 3H, CH_3), 2.67-2.79 (m, 2H, CH_2), 3.77 (septet, *J*=7.3 Hz, 1H, CH), 7.80 (d, *J*=0.6 Hz, 1H, H-5); *Anti* minor rotamer: δ = 1.33 (t, *J*=7.6 Hz, 3H, CH_3), 1.41 (d, *J*=7.5 Hz, 3H, CH_3), 1.42 (d, *J*=7.5 Hz, 3H, CH_3), 2.13 (d, *J*=0.6 Hz, 3H, CH_3), 2.67-2.79 (m, 2H, CH_2), 3.65 (septet, *J*=7.5 Hz, 1H, CH), 7.80 (d, *J*=0.7 Hz, 1H, H-5).

^{13}C-NMR (150.9 MHz, T=238 K, $CDCl_3$): Mixture of *syn* and *anti* rotamers around *i*-Pr-Ar bond: 0.54 : 0.46. *Syn* major rotamer: δ = 9.32 (CH_3), 16.79 (CH_3), 19.62 (CH_3), 19.70 (CH_3), 28.12 (CH_2), 42.96 (CH), 89.04 (C-4), 103.36 (C-2), 130.96 (C-6), 144.73 (C-5), 147.66 (C-3), 149.87 (C-1), 172.07 (C=O); *Anti* minor rotamer: δ = 9.35 (CH_3), 17.03 (CH_3), 19.63 (CH_3), 19.75 (CH_3), 28.04 (CH_2), 43.85 (CH), 91.66 (C-2), 101.43 (C-4), 130.82 (C-6), 142.17 (C-5), 147.82 (C-3), 150.81 (C-1), 172.12 (C=O).

Selective NOE: The irradiation of multiplet at 3.778 ppm of major rotamer at 243 K produce 0.04% NOE of signal at 7.803 ppm, while the irradiation of multiplet at 3.656 ppm produce 0.20% NOE of signal at 7.800 ppm, This result confirms that minor rotamer is *anti* (CH is closer to H-5).

^{13}C-NMR (150.9 MHz, T=238 K, $CDCl_3$): δ = 89.04 (dd, *J*=1.5, 6.5 Hz), 91.66 (d, *J*=7.4 Hz), 101.43 (dd, *J*=1.6, 8.7 Hz), 103.36 (d, *J*=7.9 Hz).

^{13}C-NMR (150.9 MHz, T=238 K, $CDCl_3$): δ = 89.04 (d, *J*=6.8 Hz, C-4, *syn*), 91.66 (d, *J*=7.2 Hz, C-2, *anti*), 101.43 (d, *J*=8.9 Hz, C-4, *anti*), 103.36 (d, *J*=8.7 Hz, C-2, *syn*). Major = *syn*; Minor = *anti*

Table S11. GIAO/B3LYP/6-311++G(d,p) calculations of coupling constants of two rotamers of **11**.

Rotamer	$^{3}J_{(C\text{-}2,CH)}$	$^{3}J_{(C\text{-}4,CH)}$	$^{2}J_{(C\text{-}4,H\text{-}5)}$
Anti	6.4	10.1	-1.7
Syn	9.9	6.0	-1.6
Anti (exp. 238 K)	7.2	8.9	-1.6
Syn (exp. 238 K)	8.7	6.8	-1.5

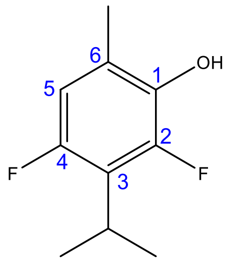

Table S12. GIAO/B3LYP/6-311++G(d,p) calculations of coupling constants of two rotamers of **12**.

Rotamer	$^{3}J_{(C\text{-}2,CH)}$	$^{3}J_{(C\text{-}4,CH)}$	$^{2}J_{(C\text{-}4,H\text{-}5)}$	$^{3}J_{(C\text{-}1,CH)}$	$^{3}J_{(C\text{-}5,CH)}$
Anti	6.4	4.6	-5.2	1.0	-0.2
Syn	4.7	6.2	-5.2	-0.02	0.6

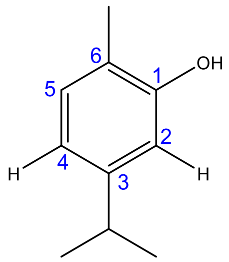

Table S13. GIAO/B3LYP/6-311++G(d,p) calculations of coupling constants of two rotamers of **13**.

Rotamer	$^{3}J_{(C\text{-}2,CH)}$	$^{3}J_{(C\text{-}4,CH)}$	$^{2}J_{(C\text{-}4,H\text{-}5)}$	$^{3}J_{(C\text{-}1,CH)}$	$^{3}J_{(C\text{-}5,CH)}$
Anti	4.9	6.2	1.4	0.9	0.2
Syn	6.4	4.6	1.3	0.4	0.5

Dynamic NMR studies

1H spectra (0.1 M in 0.6 ml CDCl3) were recorded on a Bruker II+ 600 instrument (BBO probe) at 600.13 MHz in steps of 5 K (for T ranges refer to Table 1). Temperature calibration was carried out with a B-VT 3000 unit (it was checked and calibrated with methanol and ethylene glycol reference samples). 1H NMR spectra were acquired using a spectral width of 10 kHz, an acquisition time of 1.7 s, and 32 scans, zerofilled to 64k datapoints (0.15 Hz per point) and processed without apodization. Peaks were fitted to a Lorentzian lineshape. 1H EXSY spectra were recorded on a BBO probe in steps of 5 K (for T ranges refer to Table 1). The spectra were acquired using a spectral width of 1.2 kHz, 2048 x 256 complex time domain data points, mixing times in the range of 0.03 to 0.3 s, and 2 scans in about 45 min. Linear prediction (32 coefficients and 256 points) in F1 was applied. The spectra were zerofilled to 4096 × 4096 data points and processed with a shifted square sine bell apodization in both dimensions. The populations were obtained via the integration of 1D 1H signals, and the exchange rates were calculated using the program EXSYCalc (MestreLab Research S.L., Santiago de Compostela, Spain) from diagonal- and crosspeak integrals.

Errors analysis: Usually, the presented errors in the activation parameters are the sta-tistical errors based on scattering of the data points around the Eyring straight line only. The errors in this analysis are due to inaccuracies in both the calculated rate con-stants, k, and the measured temperatures, T, and are computed according to the error propagation equations of Binsch [2] and Heinzer and Oth [3], in which errors due to both the calculated rate constants and the measured temperature are taken into ac-count. The absolute temperature errors were assumed to be σT = ±0.5 K, and the max-imum relative error in rate constants was taken to be σk/k = ±10%.

Errors analysis

The total errors E_{tot}, quoted in activation parameters tables are calculated according to the expression $E_{tot} = \sqrt{E_S^2 + E_{kT}^2}$, where E_S is the statistical error based on scattering of the data in the Eyring plot while E_{kT} is computed using error propagation equations as derived by Binsch [2] and Heinzer and Oth [2], in which errors due to both the calculated rate constants and the measured temperature are taken into account. The absolute temperature errors were assumed to be σT = ±0.5 K and the maximum relative error in rate constants was taken to be $\sigma k/k$ = ±10%.

HOmodecoupled Band-Selective NMR experiments (HOBS):

1H HOBS EXSY spectra were recorded using hobs_noesy pulse program (refer to Section III. Pulse program codes for the HOBS-EXSY experiments) on a BBO probe in steps of 5 K typically between 233 and 313 K. The spectra were acquired using a spec-tral width of 2.4 kHz, 1024 × 128 complex time domain data points, mixing times in the range of 0.03 to 1.0 s, and 8 scans in about 45 min. Linear prediction (32 coefficients and 256 points) in F1 was applied. The spectra were zerofilled to 4096 × 4096 data points and processed with a shifted square sine bell apodization in both dimensions. The populations were obtained via the integration of 1D 1H signals, and the exchange rates were calculated using the program EXSYCalc (MestreLab Research S.L.) from di-agonal- and crosspeak integrals.

NOE Experiments

Selective NOE 1H spectra were recorded on a Bruker NEO 600 instrument (prod-igy BBO probe) at 600.18 MHz at the lower measured temperature (0.1 M in 0.6 mL CDCl3). 1H NMR spectra were acquired using the selnogp pulse program, an 80 ms se-lective Gaussian pulse, a mixing time of 1 s, a spectral width of 10 kHz, an acquisition time of 4.6 s, and 256 scans, zerofilled to 64k datapoints (0.15 Hz per point) and pro-cessed with EM (LB=1) apodization.

NMR Structure elucidation

For structure elucidation of the studied compounds, the following NMR experiments were performed at low temperature: ^{1}H, ^{13}C, DEPT145, COSY, HSQC, HMBC and NOESY.

For assignment of conformers as *syn* and *anti*, the following methodology was used:

Compounds **1-3**:

1. Distinguishing the C-2 and C-4 signals of two conformers from the HMBC spectra.
2. Recording of ^{13}C spectra with two selective CW irradiations:
   A. CW irradiation at H-5 and at OH.
   B. CW irradiation at H-5 and at CH_3.
   C. CW irradiation at OH and at CH_3.
3. From the 2B ^{13}C spectrum, the C-2 and C-4 protons are distinguished and $^{3}J_{(C\text{-}2,OH)}$ is determined.
4. $^{3}J_{(C\text{-}2,CH)}$ and $^{3}J_{(C\text{-}4,CH)}$ are determined from the 2A ^{13}C spectrum.
5. $^{4}J_{(C\text{-}2,H\text{-}5)}$ and $^{2}J_{(C\text{-}4,H\text{-}5)}$ are determined from the 2C ^{13}C spectrum.
6. If $^{3}J_{(C\text{-}2,CH)} < {}^{3}J_{(C\text{-}4,CH)}$ the rotamer is *anti*.
7. If $^{3}J_{(C\text{-}2,CH)} > {}^{3}J_{(C\text{-}4,CH)}$ the rotamer is *syn*.

Compounds **4-11**:

1. Distinguishing the C-2 and C-4 signals of two conformers from the HMBC spectra.
2. Recording zggd30 ^{13}C spectrum and $^{3}J_{(C\text{-}2,CH)}$ and $^{3}J_{(C\text{-}4,CH)}$ are determined.
3. Recording ^{13}C spectrum with two selective CW irradiation at H-5 and at CH_3 and $^{4}J_{(C\text{-}2,H\text{-}5)}$ and $^{2}J_{(C\text{-}4,H\text{-}5)}$ are determined.
4. If $^{3}J_{(C\text{-}2,CH)} < {}^{3}J_{(C\text{-}4,CH)}$ the rotamer is *anti*.
5. If $^{3}J_{(C\text{-}2,CH)} > {}^{3}J_{(C\text{-}4,CH)}$ the rotamer is *syn*.

DFT calculations

All calculations were performed by means of quantum chemical calculations at the density functional theory (DFT) level using the Gaussian09 program package [4]. The geometries of all compounds were fully optimized, and the corresponding transi-tion states were localized using B3LYP [5] functional with a 6-311++G(d,p) basis set [6]. Solvent effect was included implicitly in the optimizations via the SMD [7] model with the built-in parameters for the solvent (CDCl3). The nature of all critical points was confirmed by means of the vibrational analysis. The ΔH, ΔS, and ΔG values were calculated at T = 298.15 K at the same level of theory including zero-point energy in the particular solvent environment (represented by relative permittivity) and vibra-tional, rotational, and translational thermal energy corrections.

The NMR coupling constants of two rotamers of the studied compounds were calculated using the GIAO approximation [8,9] at the GIAO/B3LYP/6-311++G**-J computational level. The test calculations of the coupling constants [10–13] of two ro-tamers of carvacrol were performed using the GIAO/B3LYP/aug-cc-pVTZ-J [14,15] and GIAO/B3LYP/6-311++G**-J [16] methods. Recently, a 6-311++G**-J basis was introduced and shown on test compounds to be very accurate for calculating coupling con-stants [16].

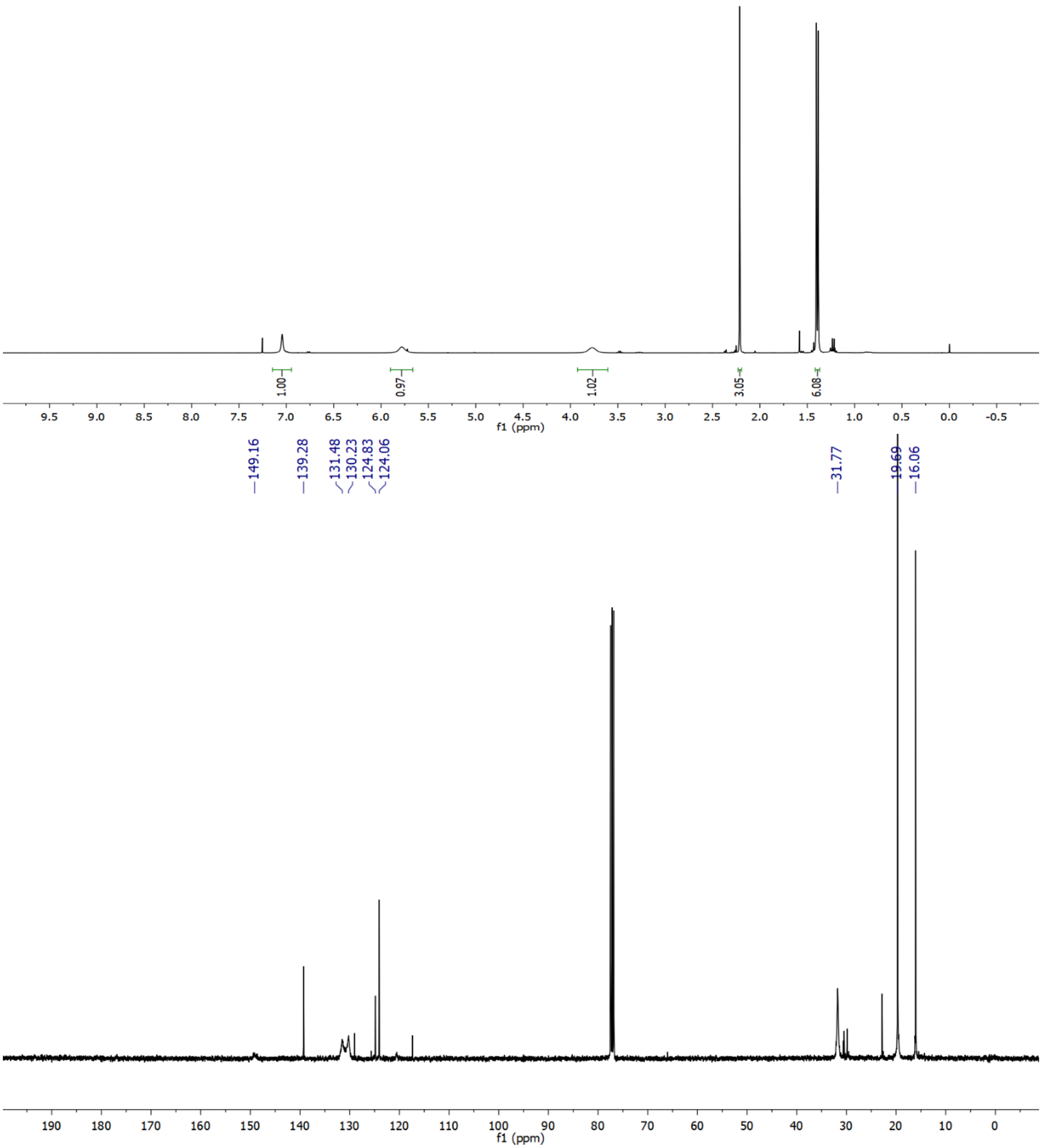

Figure S2. ^{1}H (400 MHz, 298 K, $CDCl_3$) and ^{13}C NMR (100.6 MHz, 298 K, $CDCl_3$) spectra of compound **1**

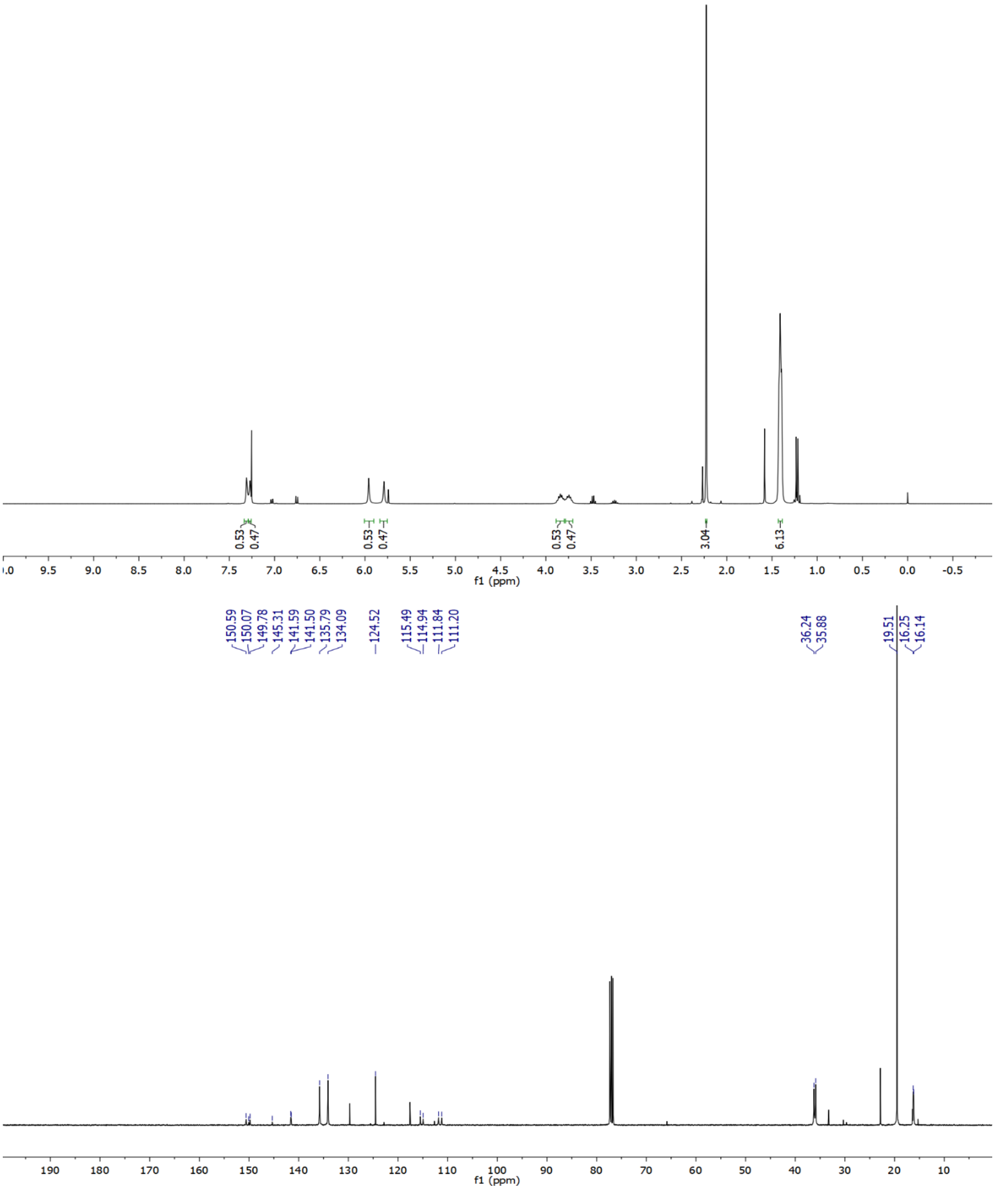

Figure S3. ^{1}H (400 MHz, 298 K, $CDCl_3$) and ^{13}C NMR (100.6 MHz, 298 K, $CDCl_3$) spectra of compound **2**

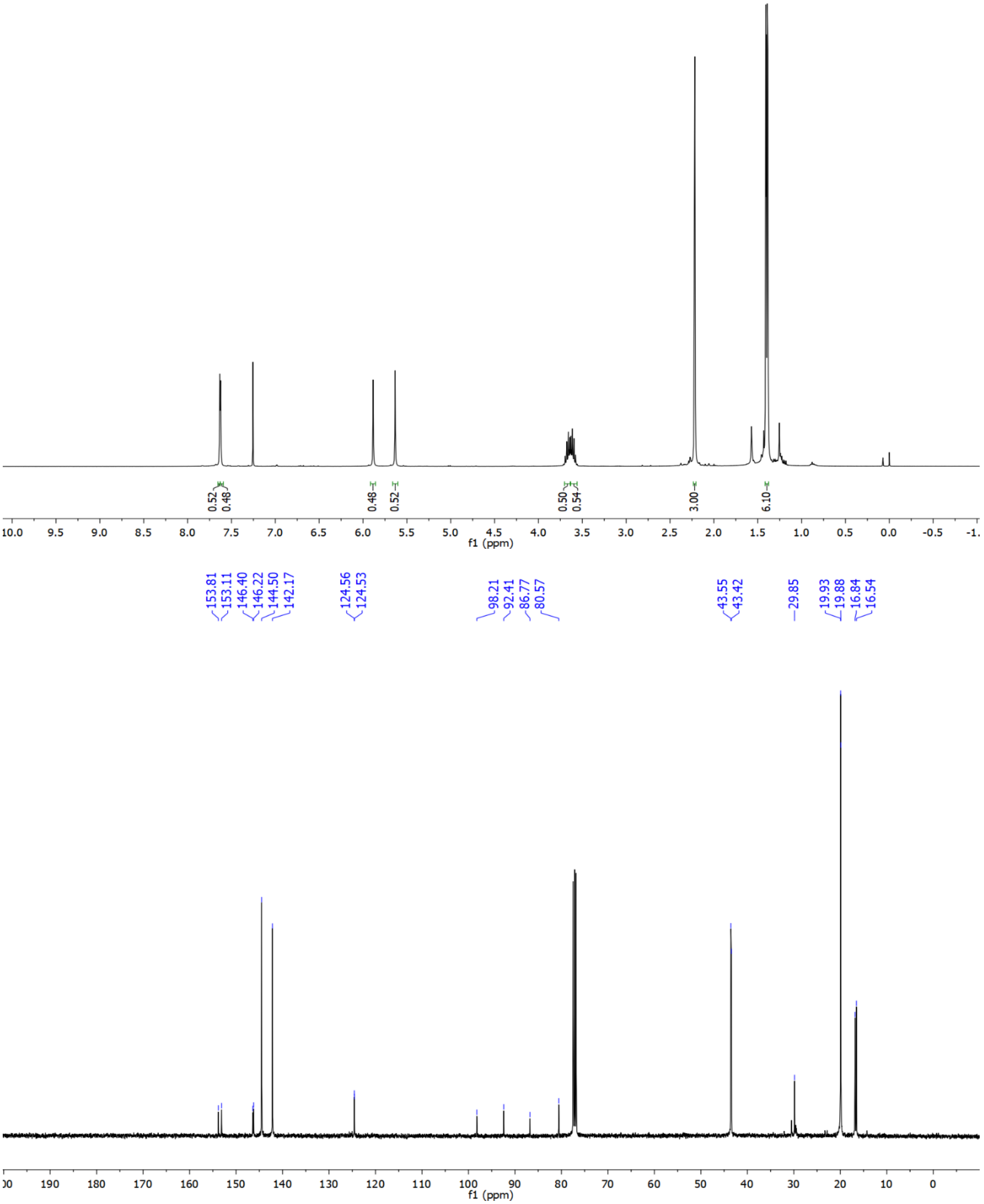

Figure S4. ^{1}H (400 MHz, 298 K, $CDCl_3$) and ^{13}C NMR (100.6 MHz, 298 K, $CDCl_3$) spectra of compound **3**

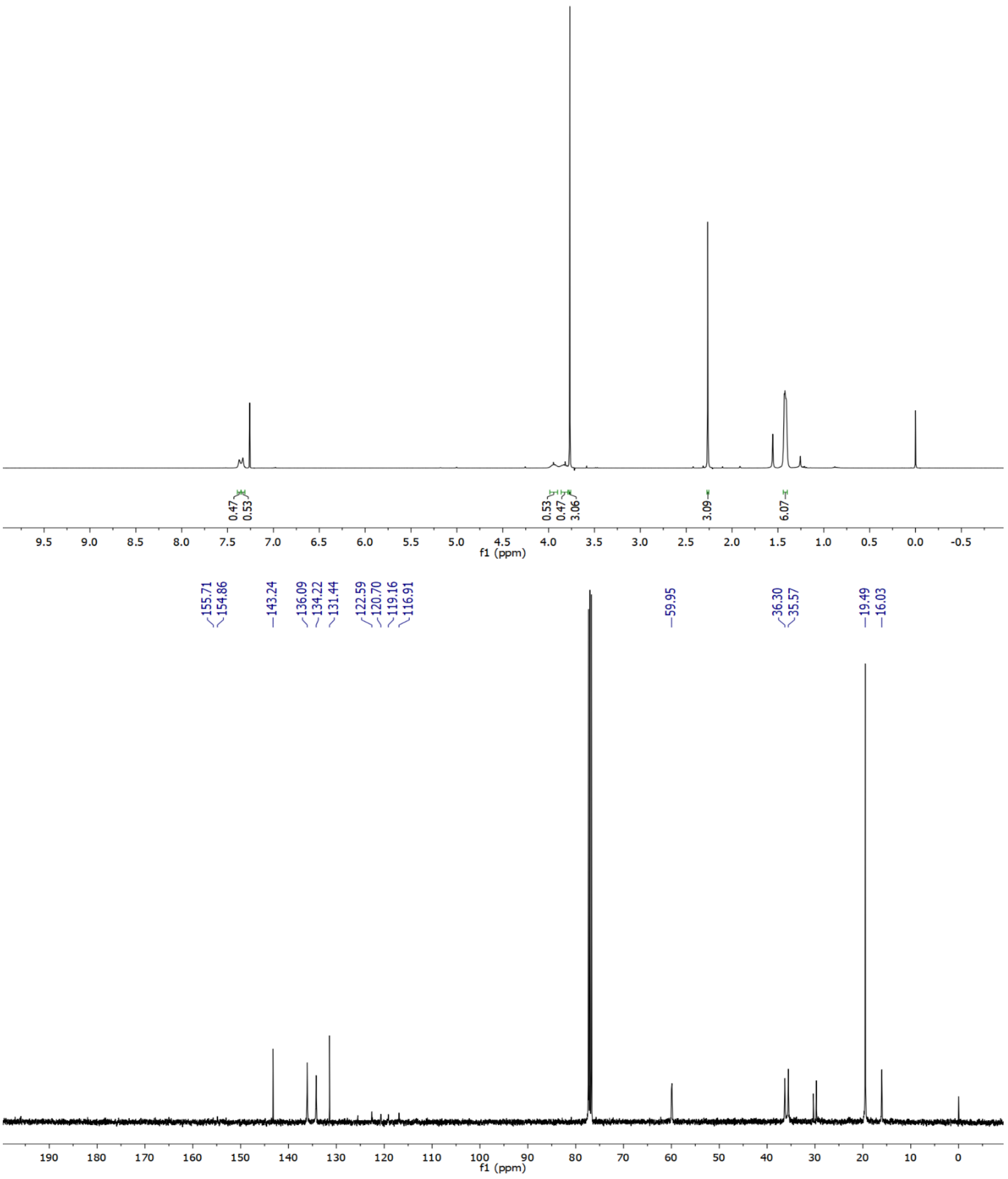

Figure S5. ^{1}H (400 MHz, 298 K, $CDCl_3$) and ^{13}C NMR (100.6 MHz, 298 K, $CDCl_3$) spectra of compound **4**

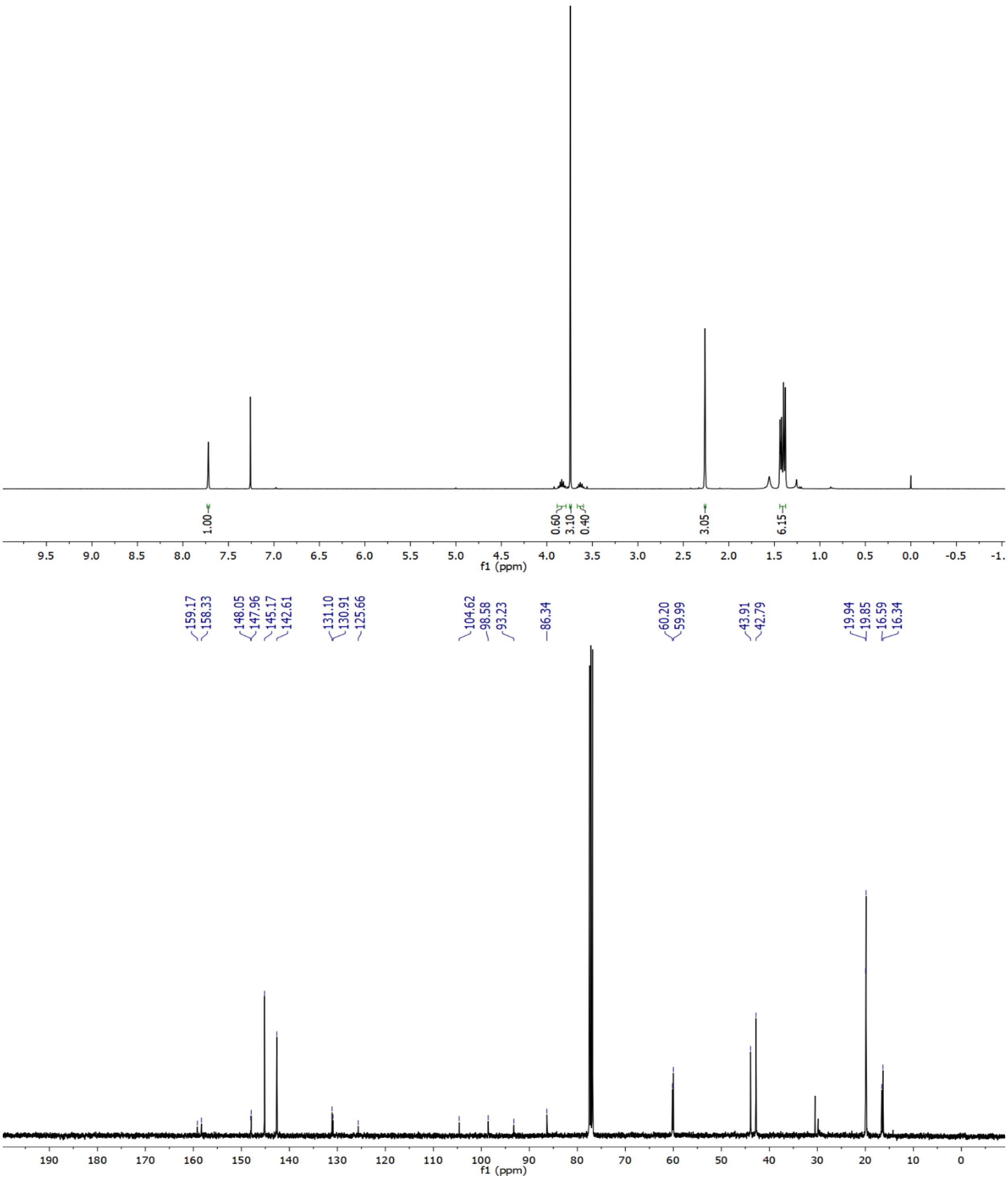

Figure S6. ^{1}H (400 MHz, 298 K, $CDCl_3$) and ^{13}C NMR (100.6 MHz, 298 K, $CDCl_3$) spectra of compound **5**

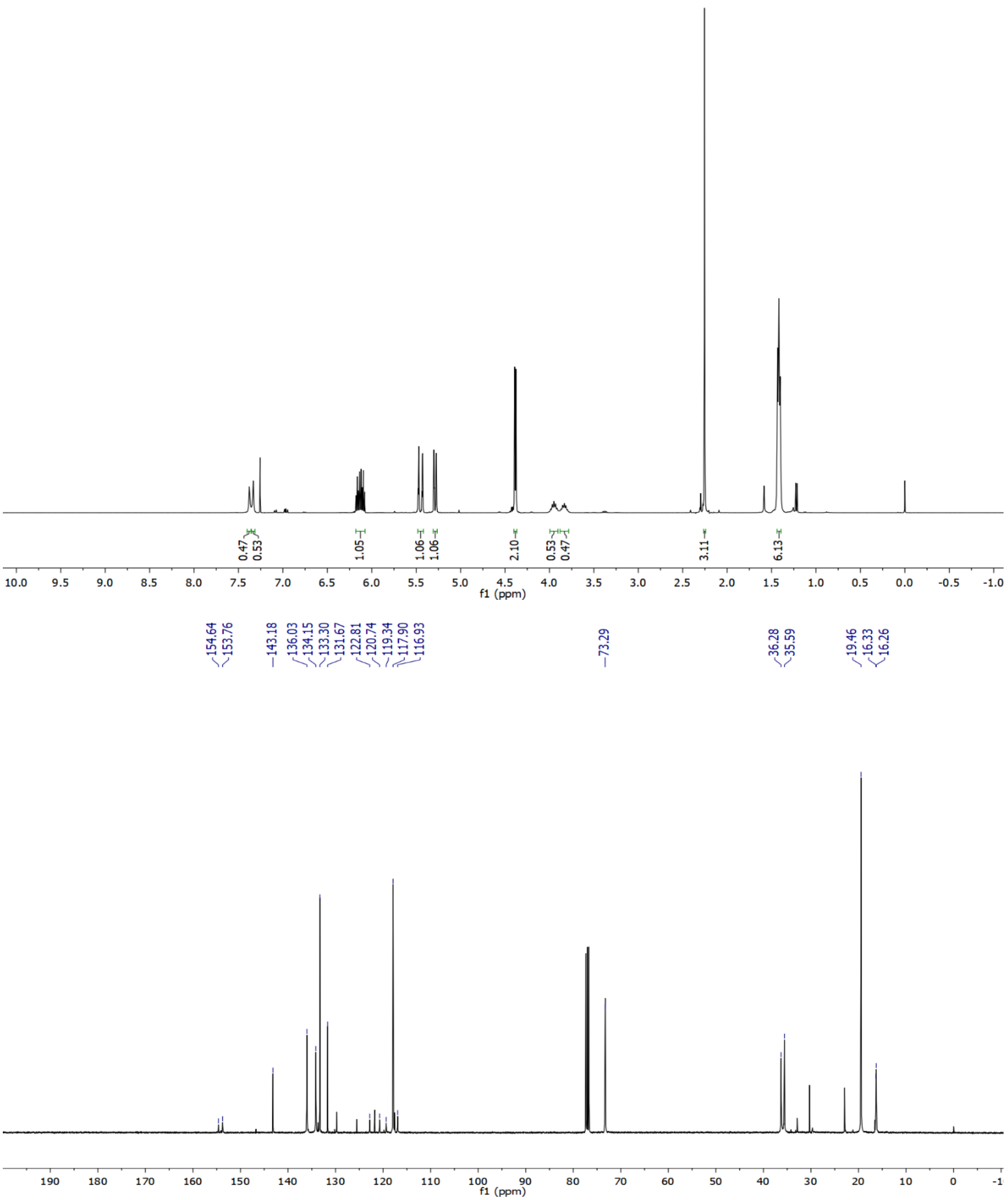

Figure S7. ^{1}H (400 MHz, 298 K, $CDCl_3$) and ^{13}C NMR (100.6 MHz, 298 K, $CDCl_3$) spectra of compound **6**

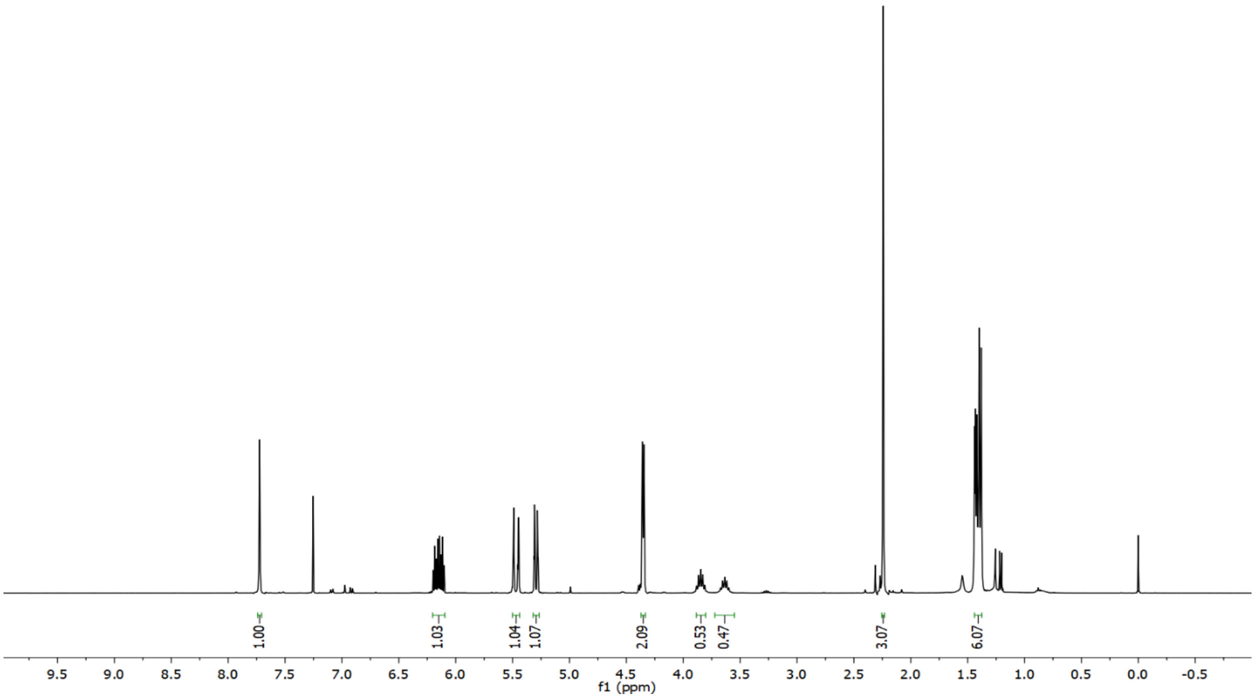

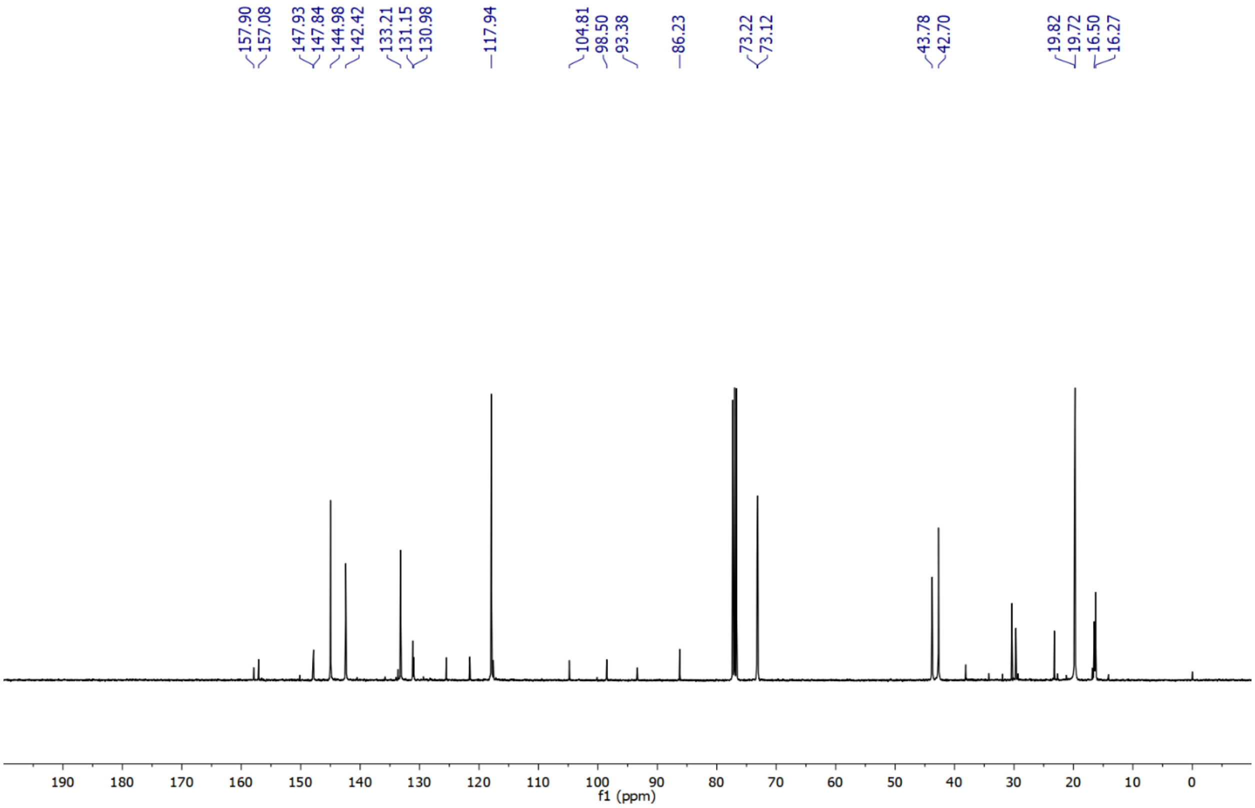

Figure S8. ^{1}H (400 MHz, 298 K, $CDCl_3$) and ^{13}C NMR (100.6 MHz, 298 K, $CDCl_3$) spectra of compound **7**

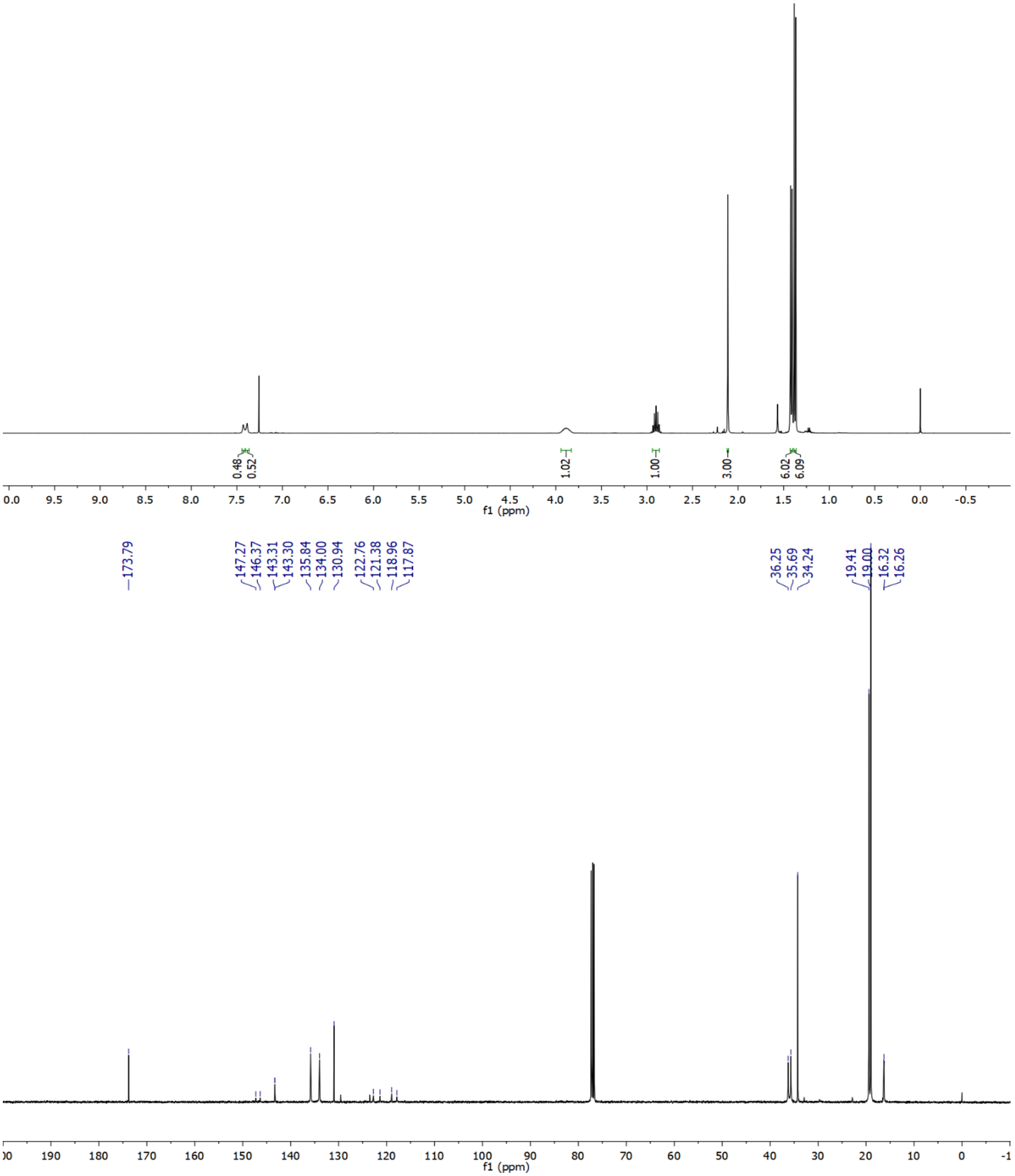

Figure S9. ^{1}H (400 MHz, 298 K, $CDCl_3$) and ^{13}C NMR (100.6 MHz, 298 K, $CDCl_3$) spectra of compound **8**

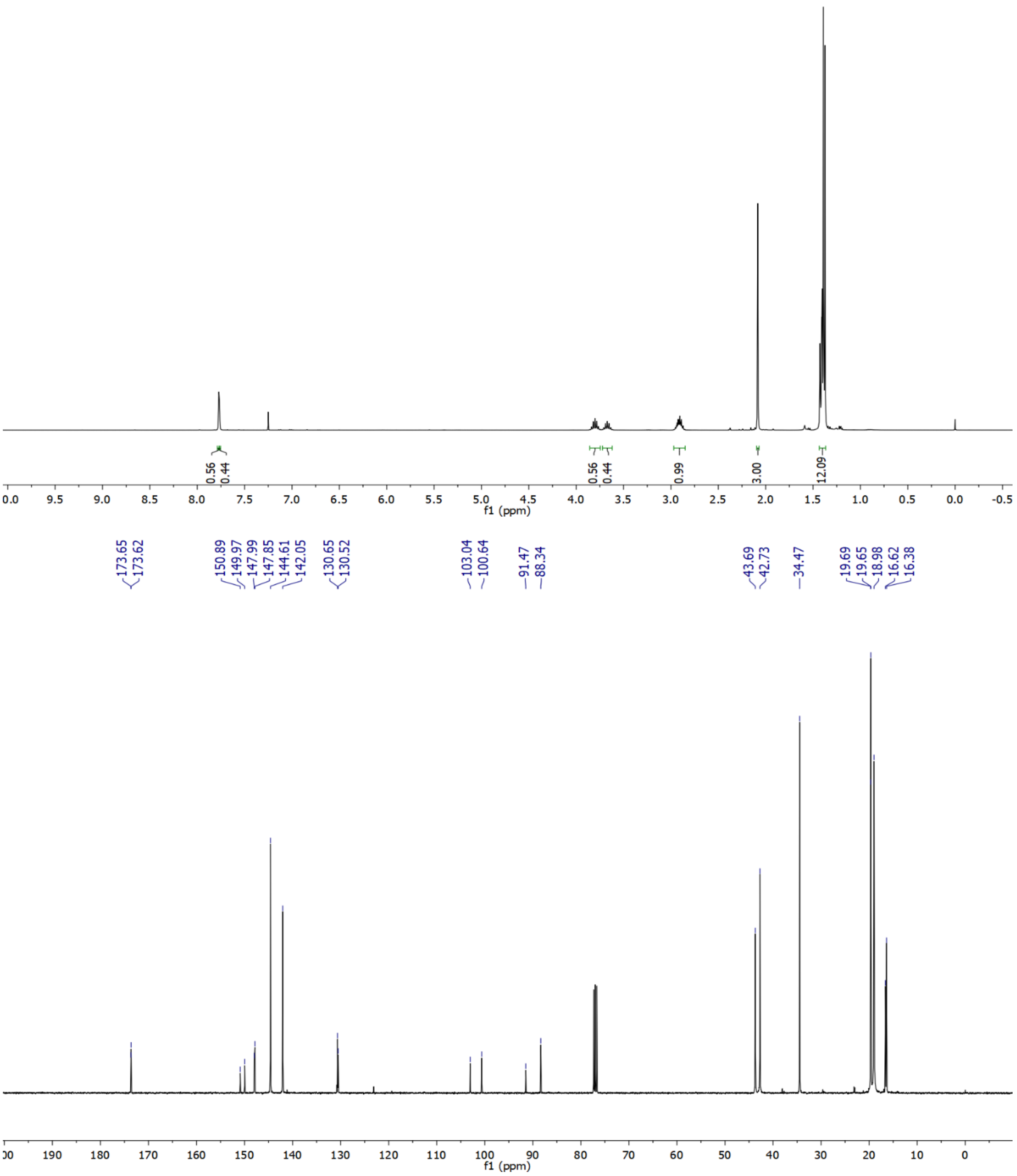

Figure S10. ^{1}H (400 MHz, 298 K, $CDCl_3$) and ^{13}C NMR (100.6 MHz, 298 K, $CDCl_3$) spectra of compound **9**

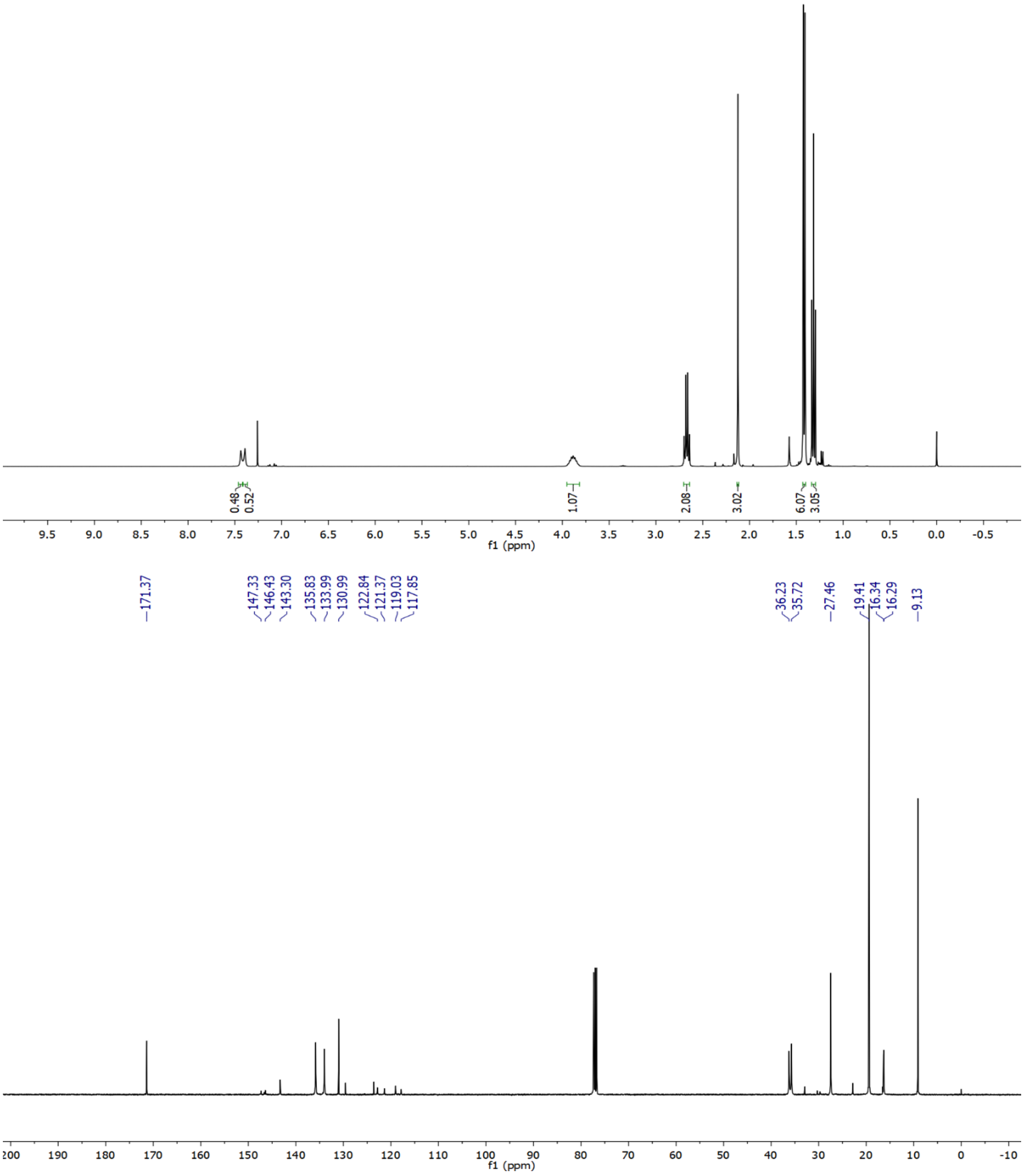

Figure S11. ^{1}H (400 MHz, 298 K, $CDCl_3$) and ^{13}C NMR (100.6 MHz, 298 K, $CDCl_3$) spectra of compound **10**

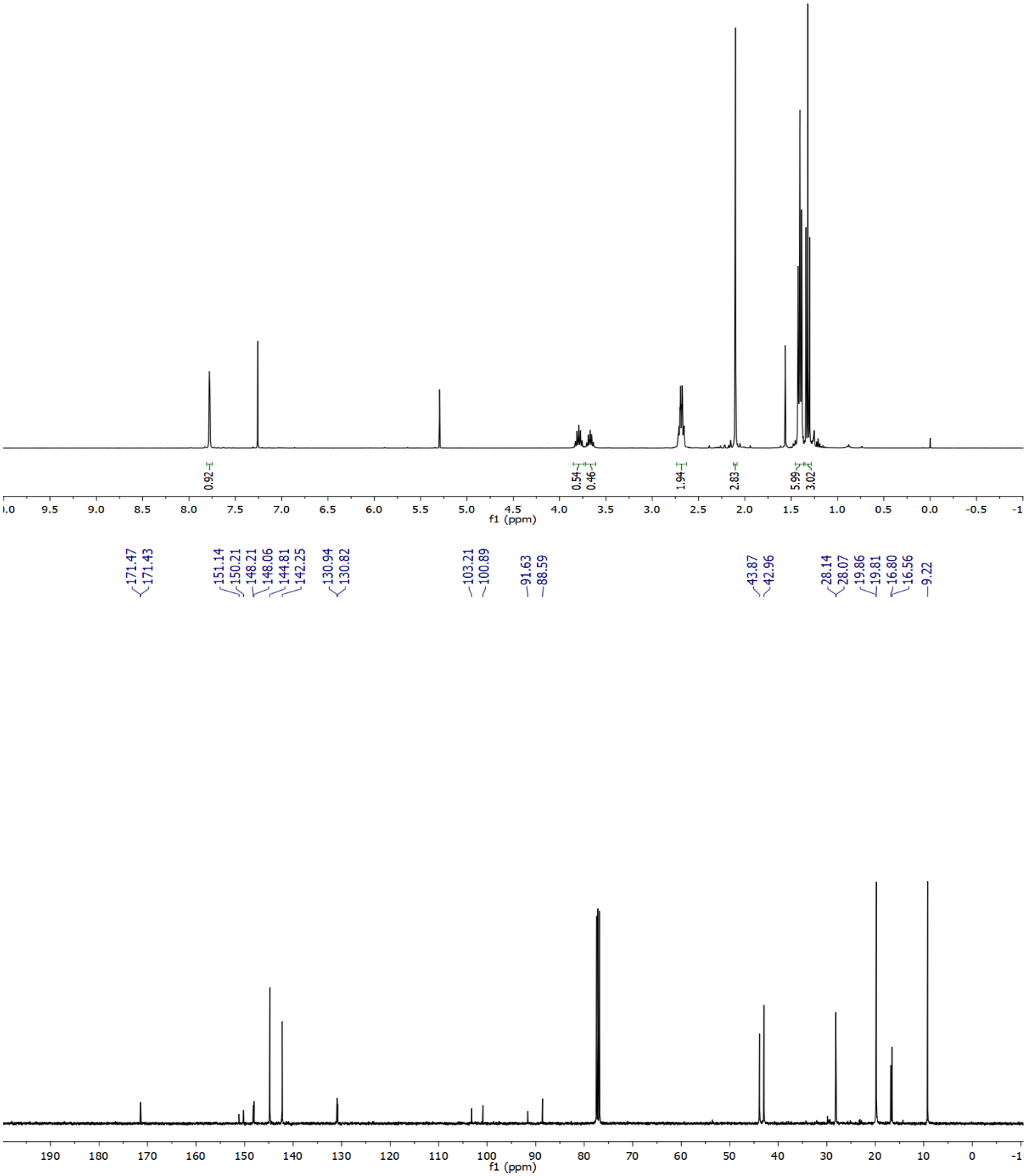

Figure S12. ^{1}H (400 MHz, 298 K, $CDCl_3$) and ^{13}C NMR (100.6 MHz, 298 K, $CDCl_3$) spectra of compound **11**

Dynamic NMR studies

Compound **1** in $CDCl_3$

^1H spectra were recorded on a Bruker II+ 600 instrument (BBO probe) at 600.13 MHz in steps of 5 K between 233 and 293 K (15 mg of **1** in 0.6 ml $CDCl_3$). Temperature calibration was done with B-VT 3000 unit (it was checked and calibrated with methanol and ethylene glycol reference samples). ^1H NMR spectra were acquired using a spectral width of 10 kHz, an acquisition time of 1.7 s and 32 scans, zerofilled to 64k data points (0.15 Hz per point) and processed without apodization. Peaks were fitted to a Lorentzian lineshape.

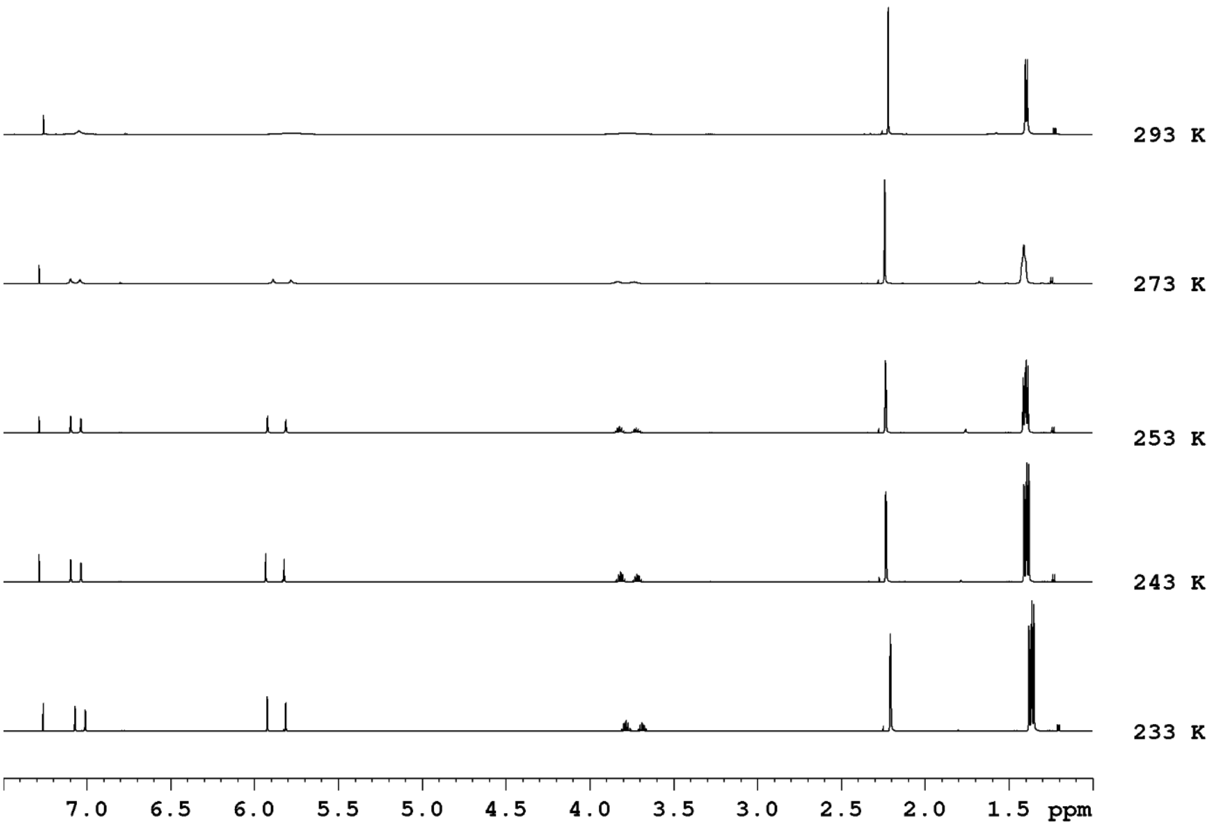

Figure S13. Stacked plots of ^1H NMR spectra of compound **1** in $CDCl_3$ at different temperatures.

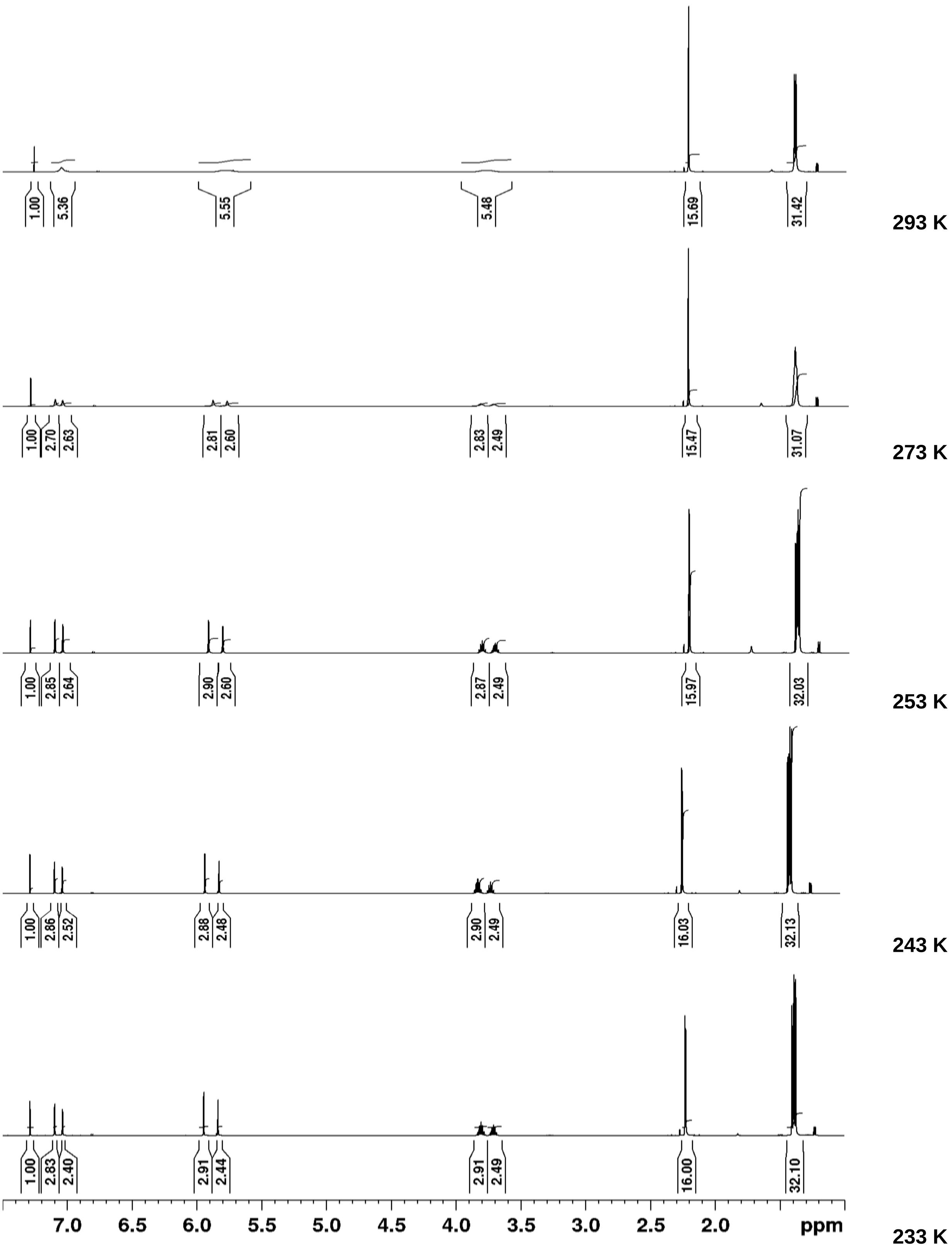

Figure S13A. Stacked plots of ^{1}H NMR spectra of compound **1** in $CDCl_3$ at different temperatures.

^{1}H EXSY spectra were recorded on a BBO probe in steps of 5 K between 233 and 258 K. The spectra were acquired using a spectral width of 1.2 kHz, 2048 x 256 complex time domain data points, mixing times in the range of 0.03 to 0.3 s and 2 scans in about 45 min. Linear prediction (32 coefficients and 256 points) in F1 was applied. The spectra were zerofilled to 4096 x 4096 data points and processed with a shifted square sine bell apodization in both dimensions. The populations were obtained by integration of 1D ^{1}H signals and the exchange rates were calculated by program EXSYCalc (MestreLab Research S.L.) from diagonal- and crosspeak integrals.

In the ^{1}H EXSY spectra of **1** the intensity of following peaks were calculated by volume integration:

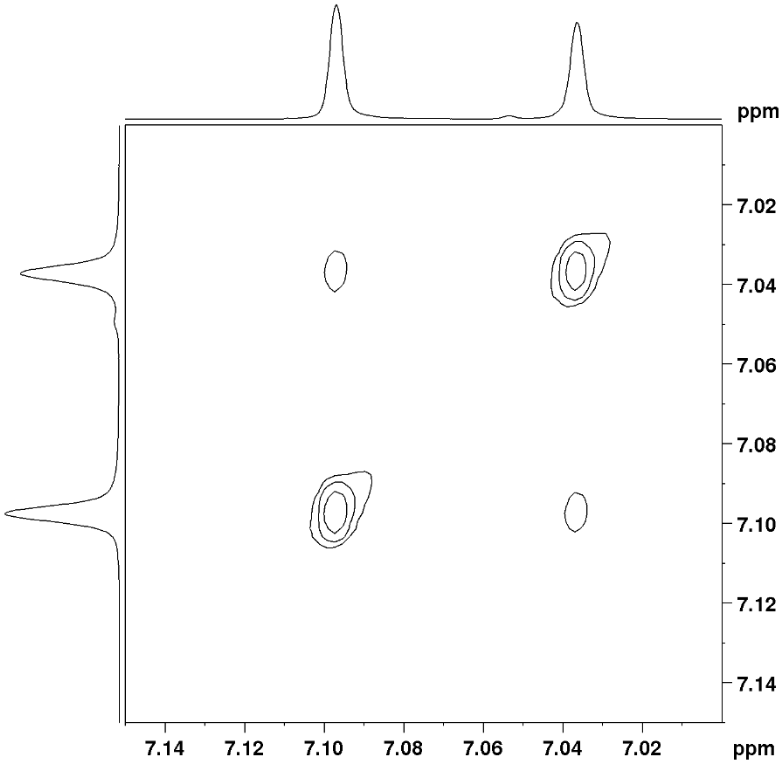

Figure S14. NOESY spectrum of compound **1** in $CDCl_3$ at 233 K using mixing time of 0.3 s.

Errors analysis

The total errors ***E_{tot}***, quoted in activation parameters tables are calculated according to the expression $E_{tot} = \sqrt{E_S^2 + E_{kT}^2}$, where ***$E_S$*** is the statistical error based on scattering of the data in the Eyring plot while ***E_{kT}*** is computed using error propagation equations as derived by Binsch [G. Binsch, in ***Dynamic NMR Spectroscopy,*** edited by **L.** M. Jackman and F. A. Cotton, **p.** 45. Academic Press, New York (1975)] and Heinzer and Oth [J. Heinzer and J. F. M. Oth, ***Helv. Chim. Acta*** 64, 258 (1981)], in which errors due to both the calculated rate constants and the measured temperature are taken into account. The absolute temperature errors were assumed to be σT = ±0.5 K and the maximum relative error in rate constants was taken to be $\sigma k/k$ = ±10%.

Table S14. Rate constants of compound **1** calculated from EXSY spectra in $CDCl_3$

T, K	*syn* to *anti*, s^{-1}	*anti* to *syn*, s^{-1}
233	0.216	0.172
238	0.383	0.371
243	0.782	0.725
248	1.467	1.375
253	2.638	2.795
258	5.036	4.902

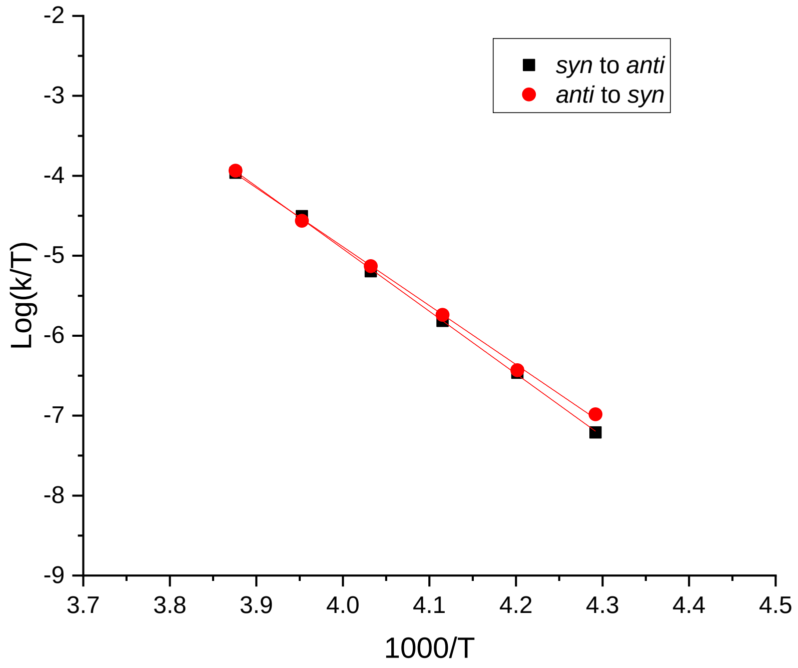

Figure S15. Eyring plot of rate constants of restricted rotation of compound **1** in $CDCl_3$

Table S15. Experimental Activation Parameters for the Exchange Processes of compound **1** calculated from ^1H EXSY spectra (signal of H-5) in $CDCl_3$

Exchange	ΔH≠(298 K)	ΔS≠(298 K)	ΔG≠(298 K)	R^2
syn to *anti*	14.6 ± 0.8	1.6 ± 3.9	14.1 ± 0.1	0.9994
anti to *syn*	15.5 ± 0.8	5.1 ± 3.4	14.0 ± 0.1	0.9998

ΔG≠ and ΔH≠ in kcal mol^{-1} and ΔS≠ in cal mol^{-1} K^{-1}

HOmodecoupled Band-Selective NMR experiments (HOBS):

^{1}H HOBS EXSY spectra were recorded using *hobs_noesy* pulse program (refer to III. Pulse program codes for the HOBS-EXSY experiments) on a BBO probe in steps of 5 K between 233 and 268 K. The spectra were acquired using a spectral width of 2.4 kHz, 1024 x 128 complex time domain data points, mixing times in the range of 0.03 to 1.0 s and 8 scans in about 45 min. Linear prediction (32 coefficients and 256 points) in F1 was applied. The spectra were zerofilled to 4096 x 4096 data points and processed with a shifted square sine bell apodization in both dimensions. The populations were obtained by integration of 1D ^{1}H signals and the exchange rates were calculated by program EXSYCalc (MestreLab Research S.L.) from diagonal- and crosspeak integrals.

In the ^{1}H EXSY spectra of **1** the intensity of following peaks were calculated by volume integration:

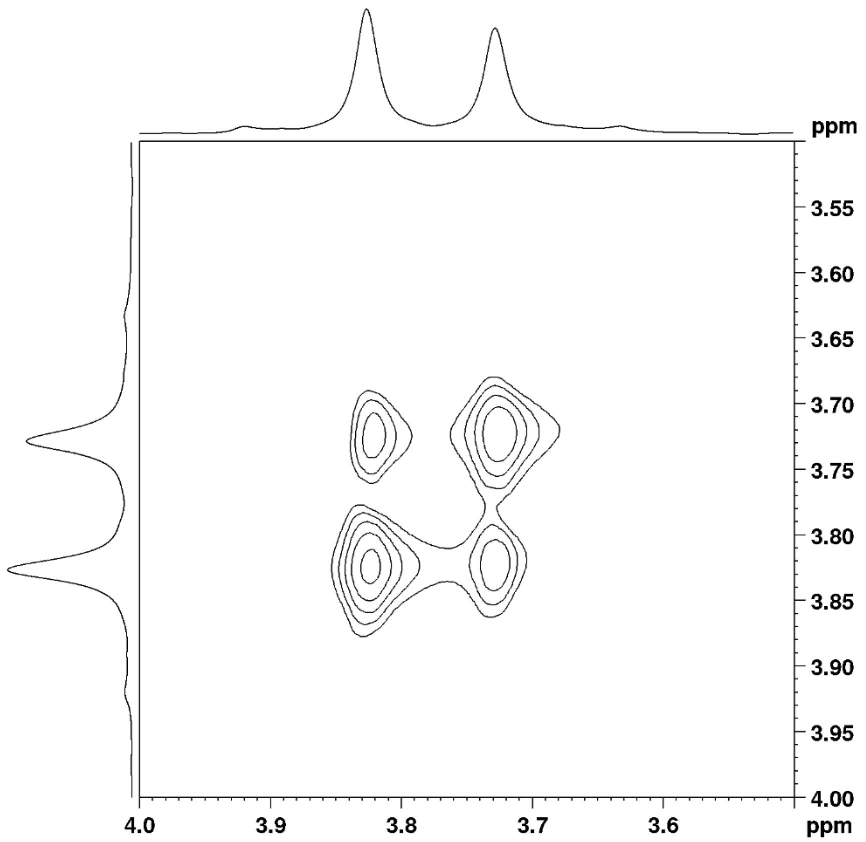

Figure S16. ^{1}H HOBS EXSY spectrum of compound **1** in $CDCl_3$ at 263 K using mixing time of 0.05 s.

Table S16. Rate constants of compound **1** calculated from ^{1}H HOBS EXSY spectra in $CDCl_3$

T, K	*syn* to *anti*, s^{-1}	*anti* to *syn*, s^{-1}
233	0.181	0.172
253	2.431	1.759
263	9.477	7.662
268	16.149	16.01

Table S17. Experimental Activation Parameters for the Exchange Processes of compound **1** calculated from ^{1}H HOBS EXSY spectra (signal of CH from *i*-Pr) in $CDCl_3$

Exchange	ΔH≠(298 K)	ΔS≠(298 K)	ΔG≠(298 K)	R^2
syn to *anti*	15.5 ± 0.7	5.0 ± 2.7	14.0 ± 0.1	0.9994
anti to *syn*	15.4 ± 1.0	4.2 ± 3.6	14.1 ± 0.1	0.9949

ΔG≠ and ΔH≠ in kcal mol^{-1} and ΔS≠ in cal mol^{-1} K^{-1}

Compound **2** in $CDCl_3$

^1H spectra were recorded on a Bruker II+ 600 instrument (BBO probe) at 600.13 MHz in steps of 5 K between 253 and 293 K (15 mg of **2** in 0.6 ml $CDCl_3$). Temperature calibration was done with B-VT 3000 unit (it was checked and calibrated with methanol and ethylene glycol reference samples). ^1H NMR spectra were acquired using a spectral width of 10 kHz, an acquisition time of 1.7 s and 32 scans, zerofilled to 64k datapoints (0.15 Hz per point) and processed without apodization. Peaks were fitted to a Lorentzian lineshape.

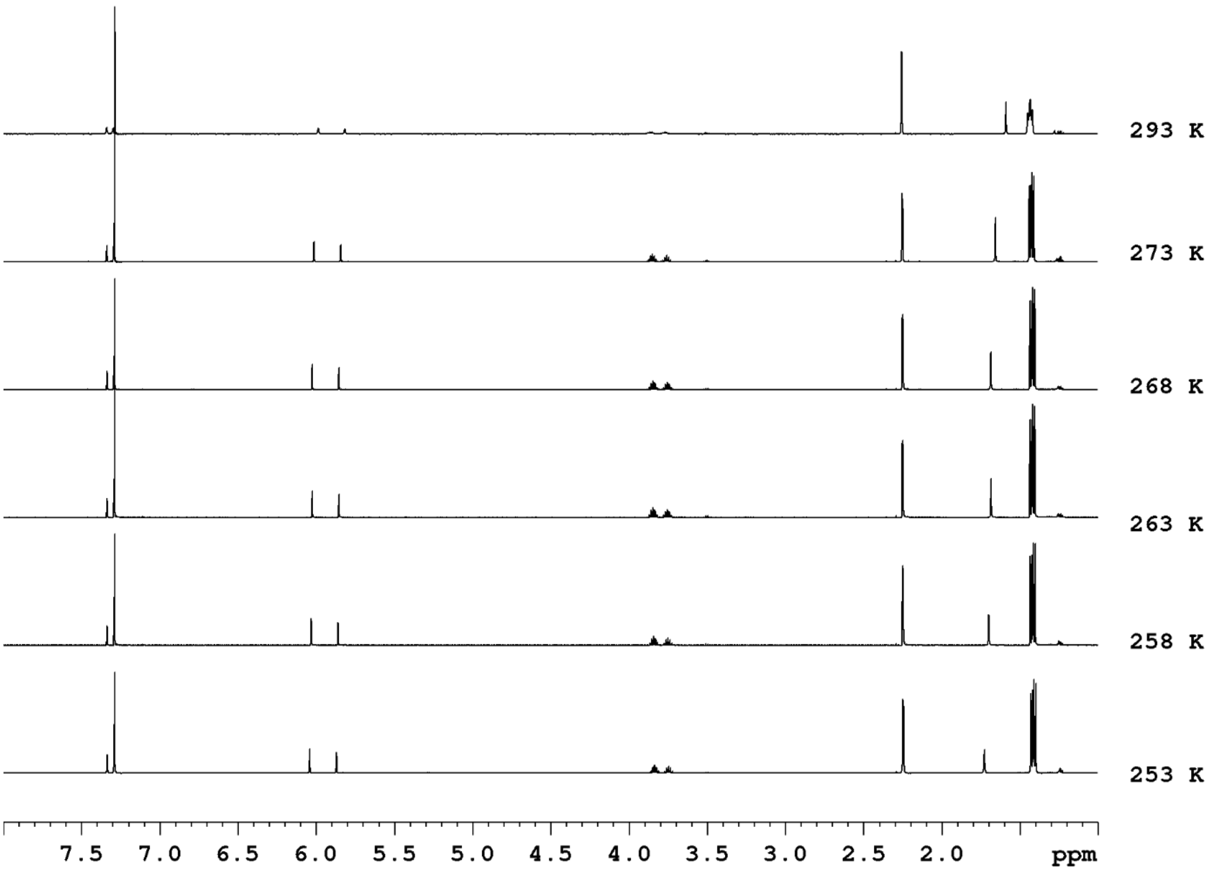

Figure S17. Stacked plots of ^1H NMR spectra of compound **2** in $CDCl_3$ at different temperatures.

^1H EXSY spectra were recorded on a BBO probe in steps of 5 K between 253 and 273 K. The spectra were acquired using a spectral width of 1.2 kHz, 2048 x 256 complex time domain data points, mixing times in the range of 0.05 to 1.0 s and 2 scans in about 45 min. Linear prediction (32 coefficients and 256 points) in F1 was applied. The spectra were zerofilled to 4096 x 4096 data points and processed with a shifted square sine bell apodization in both dimensions. The populations were obtained by integration of 1D ^1H signals and the exchange rates were calculated by program EXSYCalc (MestreLab Research S.L.) from diagonal- and crosspeak integrals.

In the ^1H EXSY spectra of **2** the intensity of following peaks were calculated by volume integration:

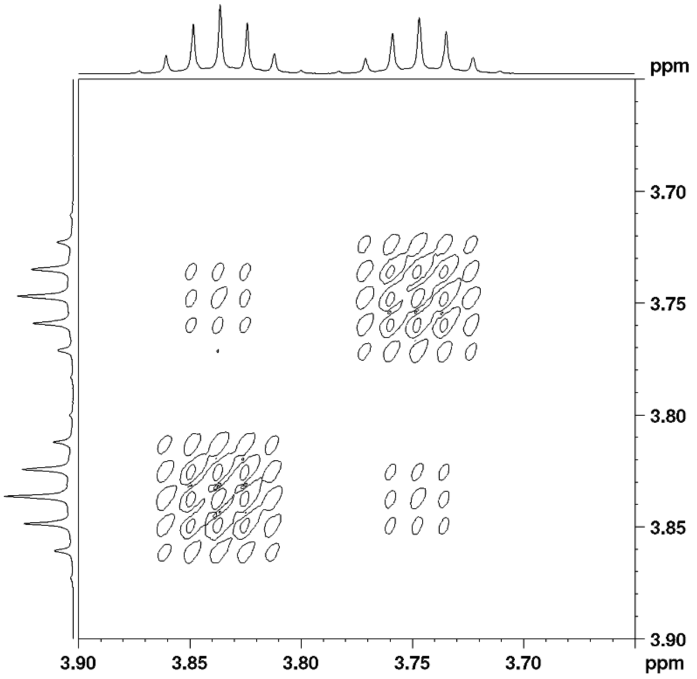

Figure S18. NOESY spectrum of compound **2** in $CDCl_3$ at 253 K using mixing time of 1.0 s.

Errors analysis

The total errors $\underline{E_{tot}}$, quoted in activation parameters tables are calculated according to the expression $E_{tot} = \sqrt{E_S^2 + E_{kT}^2}$, where E_S is the statistical error based on scattering of the data in the Eyring plot while E_{kT} is computed using error propagation equations as derived by Binsch [G. Binsch, in ***Dynamic NMR Spectroscopy,*** edited by **L.** M. Jackman and F. A. Cotton, **p.** 45. Academic Press, New York (1975)] and Heinzer and Oth [J. Heinzer and J. F. M. Oth, ***Helv. Chim.Acta*** 64, 258 (1981)], in which errors due to both the calculated rate constants and the measured temperature are taken into account. The absolute temperature errors were assumed to be σT = ±0.5 K and the maximum relative error in rate constants was taken to be $\sigma k/k$ = ±10%.

Table S18. Rate constants of compound **2** calculated from EXSY spectra in $CDCl_3$

T, K	*syn* to *anti*,s^{-1}	*anti* to *syn*,s^{-1}
253	0.171	0.140
258	0.311	0.284
263	0.585	0.556
268	0.874	0.849
273	1.726	1.595

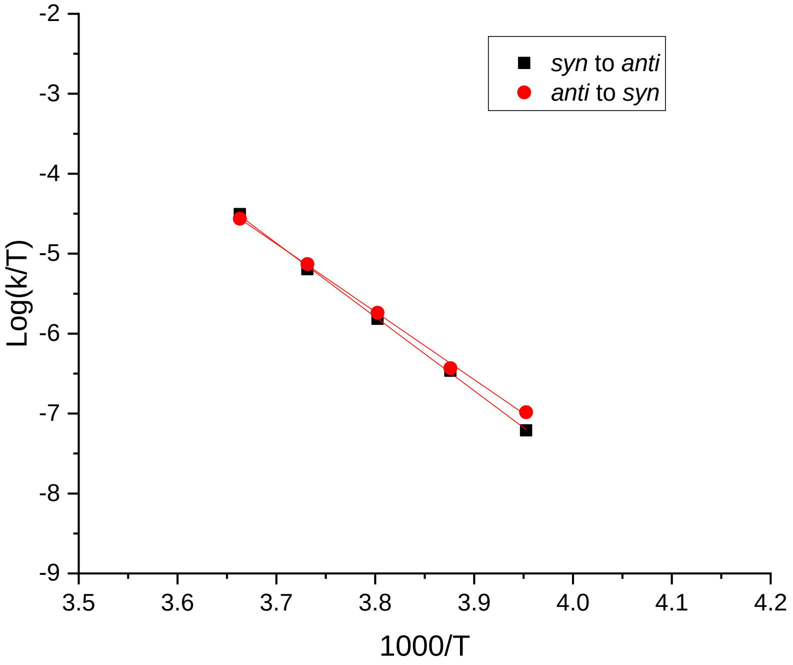

Figure S19. Eyring plot of rate constants of restricted rotation of compound **2** in $CDCl_3$

Table S19. Experimental Activation Parameters for the Exchange Processes of compound **2** calculated from ^{1}H EXSY spectra (signal of CH from *i*-Pr) in $CDCl_3$

Exchange	ΔH≠(298 K)	ΔS≠(298 K)	ΔG≠(298 K)	R^2
syn to *anti*	15.0 ± 1.2	-2.4 ± 9.7	15.7 ± 0.1	0.9975
anti to *syn*	15.8 ± 1.2	0.7 ± 2.5	15.6 ± 0.1	0.9975

ΔG≠ and ΔH≠ in kcal mol^{-1} and ΔS≠ in cal mol^{-1} K^{-1}

HOmodecoupled Band-Selective NMR experiments (HOBS):

^{1}H HOBS EXSY spectra were recorded using *hobs_noesy* pulse program (refer to III. Pulse program codes for the HOBS-EXSY experiments) on a BBO probe in steps of 5 K between 253 and 283 K. The spectra were acquired using a spectral width of 2.4 kHz, 1024 x 128 complex time domain data points, mixing times in the range of 0.03 to 1.5 s and 8 scans in about 45 min. Linear prediction (32 coefficients and 256 points) in F1 was applied. The spectra were zerofilled to 4096 x 4096 data points and processed with a shifted square sine bell apodization in both dimensions. The populations were obtained by integration of 1D ^{1}H signals and the exchange rates were calculated by program EXSYCalc (MestreLab Research S.L.) from diagonal- and crosspeak integrals.

In the ^{1}H EXSY spectra of **2** the intensity of following peaks were calculated by volume integration:

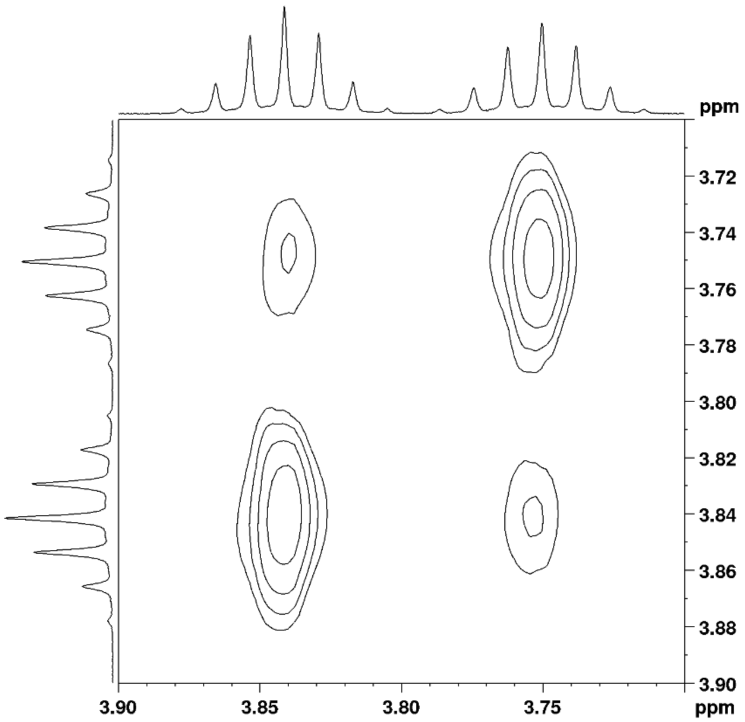

Figure S20. ^{1}H HOBS EXSY spectrum of compound **2** in $CDCl_3$ at 268 K using mixing time of 0.3 s.

Table S20. Rate constants of compound **2** calculated from ^{1}H HOBS EXSY spectra (signal of CH from *i*-Pr) in $CDCl_3$

T, K	*syn* to *anti*, s^{-1}	*anti* to *syn*, s^{-1}
253	0.156	0.150
263	0.629	0.509
268	1.125	0.979
273	2.193	1.343
278	4.019	2.445
283	7.008	6.084

Table S21. Experimental Activation Parameters for the Exchange Processes of compound **2** calculated from ^{1}H HOBS EXSY spectra (signal of CH from *i*-Pr) in $CDCl_3$

Exchange	$\Delta H^{\neq}$(298 K)	$\Delta S^{\neq}$(298 K)	$\Delta G^{\neq}$(298 K)	R^2
syn to *anti*	17.5 ± 0.8	7.4 ± 3.0	15.3 ± 0.1	0.9999
anti to *syn*	16.2 ± 1.1	1.9 ± 3.2	15.6 ± 0.1	0.9923

$\Delta G^{\neq}$ and $\Delta H^{\neq}$ in kcal mol^{-1} and $\Delta S^{\neq}$ in cal mol^{-1} K^{-1}

Compound **3** in $CDCl_3$

^1H spectra were recorded on a Bruker II+ 600 instrument (BBO probe) at 600.13 MHz in steps of 5 K between 253 and 293 K (15 mg of **3** in 0.6 ml $CDCl_3$). Temperature calibration was done with B-VT 3000 unit (it was checked and calibrated with methanol and ethylene glycol reference samples). ^1H NMR spectra were acquired using a spectral width of 10 kHz, an acquisition time of 1.7 s and 32 scans, zerofilled to 64k datapoints (0.15 Hz per point) and processed without apodization. Peaks were fitted to a Lorentzian lineshape.

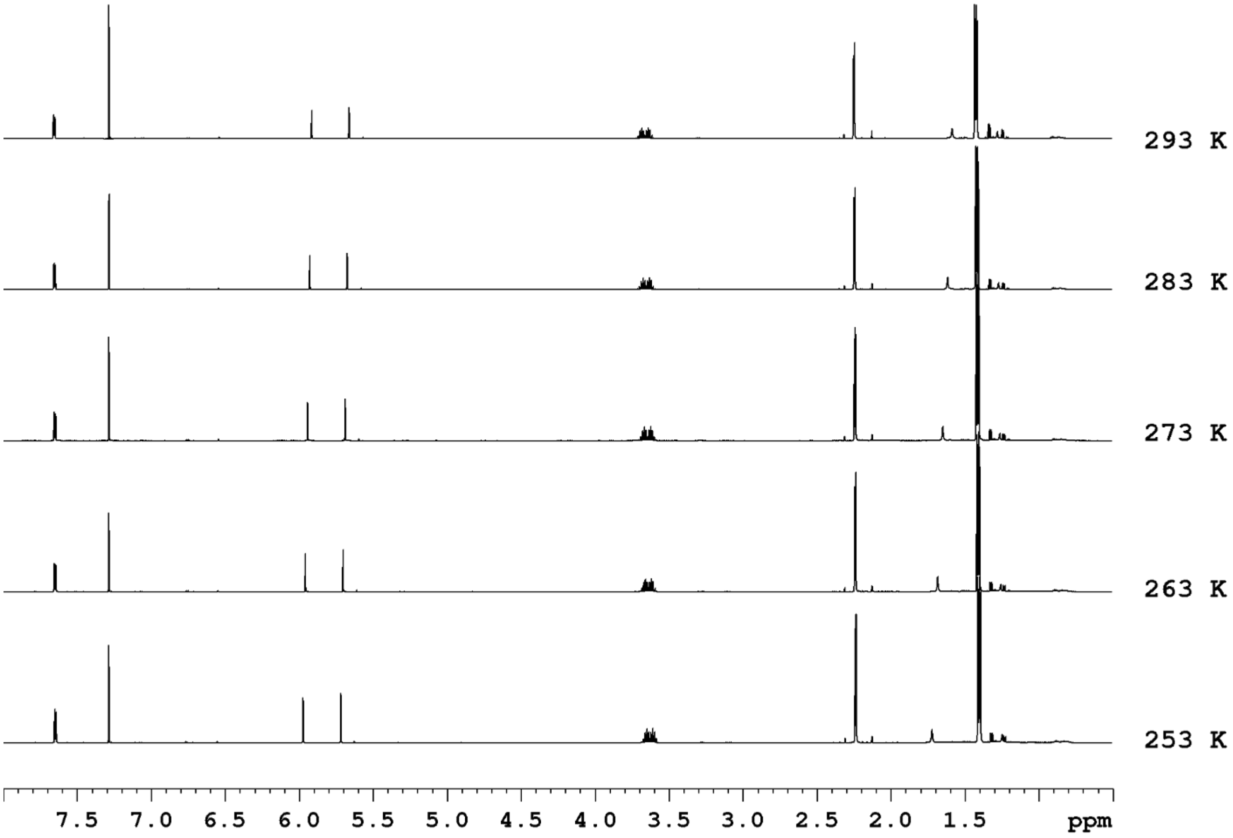

Figure S21. Stacked plots of ^1H NMR spectra of compound **3** in $CDCl_3$ at different temperatures.

^1H EXSY spectra were recorded on a BBO probe in steps of 5 K between 253 and 293 K. The spectra were acquired using a spectral width of 1.2 kHz, 2048 x 256 complex time domain data points, mixing times in the range of 0.05 to 1.0 s and 2 scans in about 45 min. Linear prediction (32 coefficients and 256 points) in F1 was applied. The spectra were zerofilled to 4096 x 4096 data points and processed with a shifted square sine bell apodization in both dimensions. The populations were obtained by integration of 1D ^1H signals and the exchange rates were calculated by program EXSYCalc (MestreLab Research S.L.) from diagonal- and crosspeak integrals.

In the ^1H EXSY spectra of **3** the intensity of following peaks were calculated by volume integration:

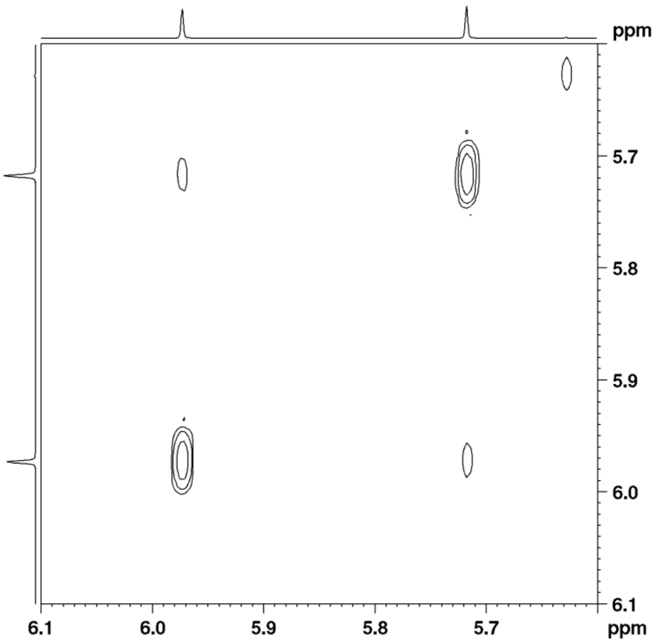

Figure S22. NOESY spectrum of compound **3** in $CDCl_3$ at 253 K using mixing time of 1.5 s.

Errors analysis

The total errors $\underline{\boldsymbol{E_{tot}}}$, quoted in activation parameters tables are calculated according to the expression $E_{tot} = \sqrt{E_S^2 + E_{kT}^2}$, where $\boldsymbol{E_S}$ is the statistical error based on scattering of the data in the Eyring plot while $\boldsymbol{E_{kT}}$ is computed using error propagation equations as derived by Binsch [G. Binsch, in ***Dynamic NMR Spectroscopy,*** edited by **L.** M. Jackman and F. A. Cotton, **p.** 45. Academic Press, New York (1975)] and Heinzer and Oth [J. Heinzer and J. F. M. Oth, ***Helv. Chim.Acta*** 64, 258 (1981)], in which errors due to both the calculated rate constants and the measured temperature are taken into account. The absolute temperature errors were assumed to be $\boldsymbol{\sigma T}$ = ±0.5 K and the maximum relative error in rate constants was taken to be $\boldsymbol{\sigma k/k}$ = ±10%.

Table S22. Rate constants of compound **3** calculated from EXSY spectra in $CDCl_3$

T, K	*syn* to *anti*, s^{-1}	*anti* to *syn*, s^{-1}
253	0.027	0.031
263	0.076	0.081
273	0.215	0.235
283	0.591	0.643
293	1.609	1.756

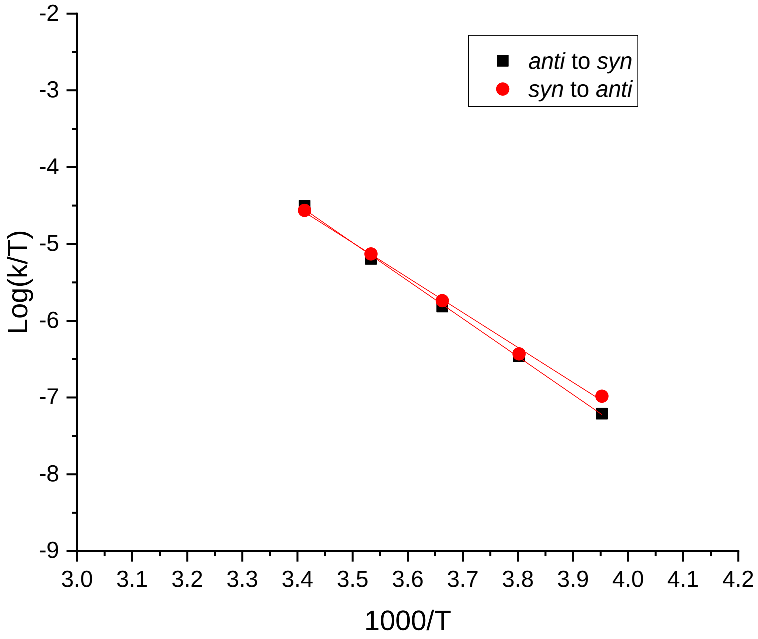

Figure S23. Eyring plot of rate constants of restricted rotation of compound **3** in $CDCl_3$

Table S23. Experimental Activation Parameters for the Exchange Processes of compound **3** calculated from ^1H EXSY spectra (signal of OH) in $CDCl_3$

Exchange	ΔH≠(298 K)	ΔS≠(298 K)	ΔG≠(298 K)	R^2
syn to *anti*	14.5 ± 0.6	-8.1 ± 2.4	16.9 ± 0.1	0.9993
anti to *syn*	14.4 ± 0.7	-8.4 ± 2.4	16.9 ± 0.1	0.9988

ΔG≠ and ΔH≠ in kcal mol^{-1} and ΔS≠ in cal mol^{-1} K^{-1}

HOmodecoupled Band-Selective NMR experiments (HOBS):

^1H HOBS EXSY spectra were recorded using *hobs_noesy* pulse program (refer to III. Pulse program codes for the HOBS-EXSY experiments) on a BBO probe in steps of 5 K between 263 and 293 K. The spectra were acquired using a spectral width of 2.4 kHz, 1024 x 128 complex time domain data points, mixing times in the range of 0.03 to 1.5 s and 8 scans in about 45 min. Linear prediction (32 coefficients and 256 points) in F1 was applied. The spectra were zerofilled to 4096 x 4096 data points and processed with a shifted square sine bell apodization in both dimensions. The populations were obtained by integration of 1D ^1H signals and the exchange rates were calculated by program EXSYCalc (MestreLab Research S.L.) from diagonal- and crosspeak integrals.

In the ^1H EXSY spectra of **3** the intensity of following peaks were calculated by volume integration:

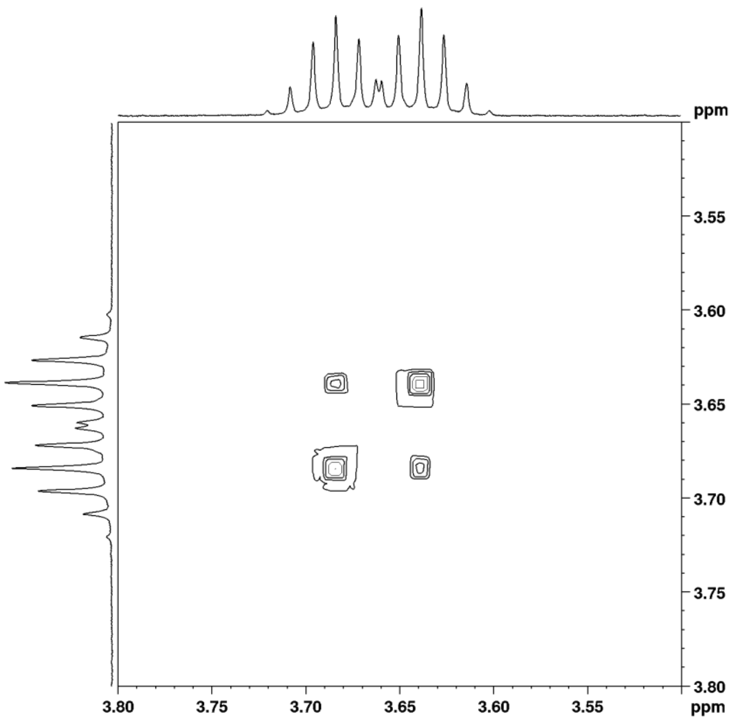

Figure S24. ^1H HOBS EXSY spectrum of compound **3** in $CDCl_3$ at 293 K using mixing time of 0.2 s.

Table S24. Rate constants of compound **3** calculated from ^1H HOBS EXSY spectra in $CDCl_3$

T, K	*syn* to *anti*,s^{-1}	*anti* to *syn*,s^{-1}
263	0.142	0.176
268	0.194	0.211
273	0.295	0.316
278	0.524	0.428
283	0.701	0.815
293	1.982	2.075

Table S25. Experimental Activation Parameters for the Exchange Processes of compound **3** calculated from ^1H HOBS EXSY spectra (signal of CH from *i*-Pr) in $CDCl_3$

Exchange	ΔH≠(298 K)	ΔS≠(298 K)	ΔG≠(298 K)	R^2
syn to *anti*	13.0 ± 1.0	-13.0 ± 3.5	16.9 ± 0.1	0.9940
anti to *syn*	12.3 ± 1.2	-15.2 ± 4.4	16.9 ± 0.1	0.9825

ΔG≠ and ΔH≠ in kcal mol^{-1} and ΔS≠ in cal mol^{-1} K^{-1}

Compound **4** in $CDCl_3$

^{1}H spectra were recorded on a Bruker II+ 600 instrument (BBO probe) at 600.13 MHz in steps of 5 K between 253 and 293 K (15 mg of **4** in 0.6 ml $CDCl_3$). Temperature calibration was done with B-VT 3000 unit (it was checked and calibrated with methanol and ethylene glycol reference samples). ^{1}H NMR spectra were acquired using a spectral width of 10 kHz, an acquisition time of 1.7 s and 32 scans, zerofilled to 64k datapoints (0.15 Hz per point) and processed without apodization. Peaks were fitted to a Lorentzian lineshape.

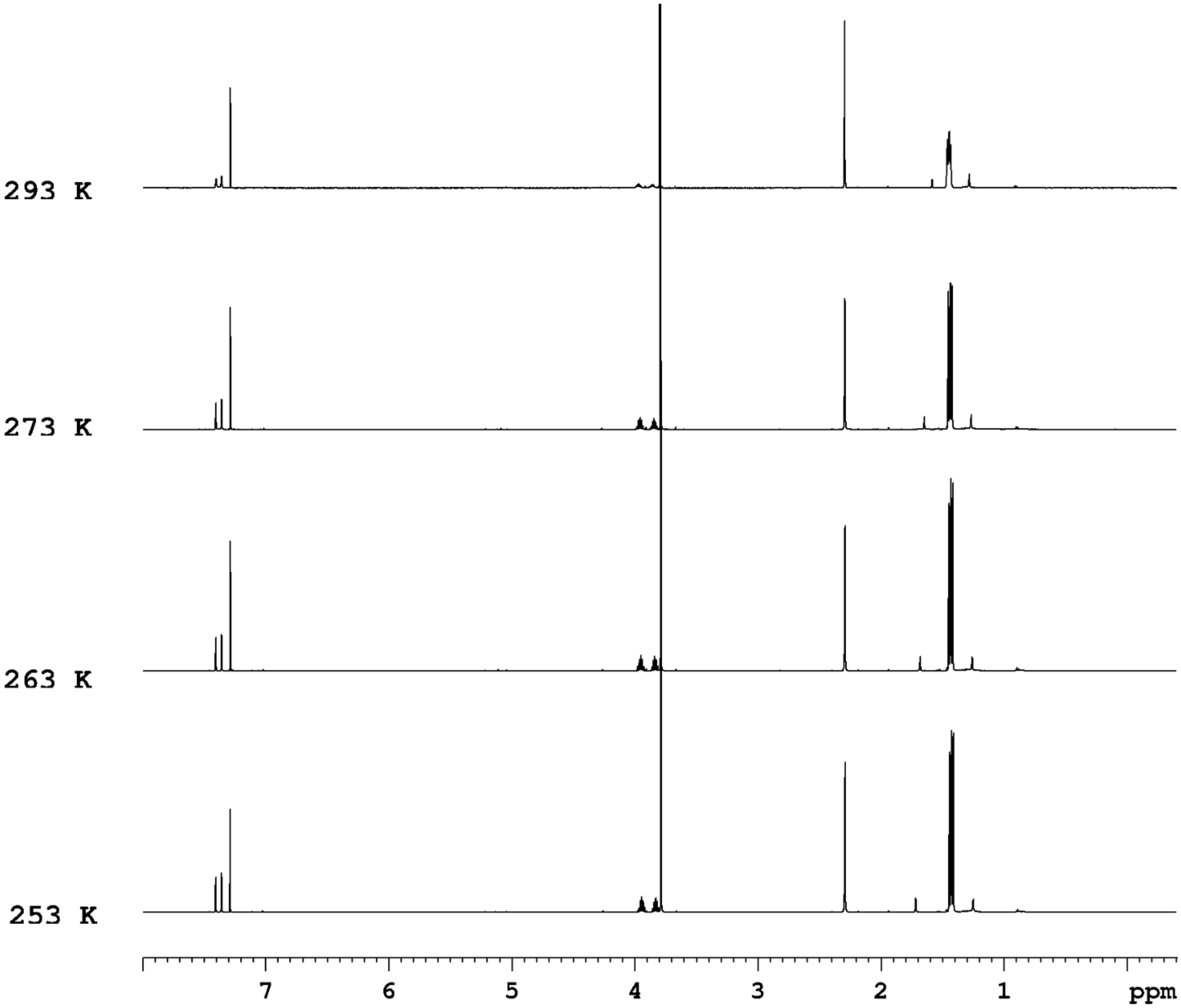

Figure S25. Stacked plots of ^{1}H NMR spectra of compound **4** in $CDCl_3$ at different temperatures.

^{1}H EXSY spectra were recorded on a BBO probe in steps of 5 K between 253 and 273 K. The spectra were acquired using a spectral width of 1.2 kHz, 2048 x 256 complex time domain data points, mixing times in the range of 0.05 to 1.0 s and 2 scans in about 45 min. Linear prediction (32 coefficients and 256 points) in F1 was applied. The spectra were zerofilled to 4096 x 4096 data points and processed with a shifted square sine bell apodization in both dimensions. The populations were obtained by integration of 1D ^{1}H

signals and the exchange rates were calculated by program EXSYCalc (MestreLab Research S.L.) from diagonal- and crosspeak integrals.

In the ^{1}H EXSY spectra of **4** the intensity of following peaks were calculated by volume integration:

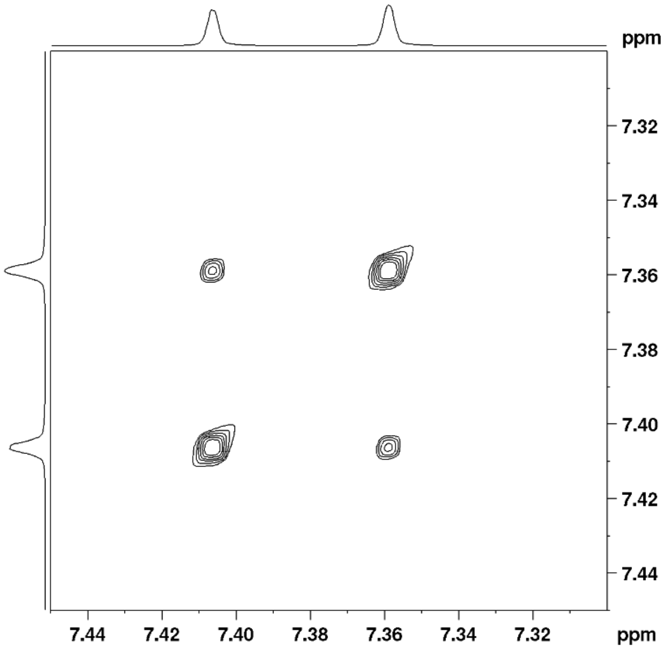

Figure S26. NOESY spectrum of compound **4** in $CDCl_3$ at 253 K using mixing time of 1.0 s.

Errors analysis

The total errors ***E_{tot}***, quoted in activation parameters tables are calculated according to the expression $E_{tot} = \sqrt{E_S^2 + E_{kT}^2}$, where ***$E_S$*** is the statistical error based on scattering of the data in the Eyring plot while ***E_{kT}*** is computed using error propagation equations as derived by Binsch [G. Binsch, in ***Dynamic NMR Spectroscopy,*** edited by **L.** M. Jackman and F. A. Cotton, **p.** 45. Academic Press, New York (1975)] and Heinzer and Oth [J. Heinzer and J. F. M. Oth, ***Helv. Chim.Acta*** 64, 258 (1981)], in which errors due to both the calculated rate constants and the measured temperature are taken into account. The absolute temperature errors were assumed to be ***σT*** = ±0.5 K and the maximum relative error in rate constants was taken to be ***σk/k*** = ±10%.

Table S26. Rate constants of compound **4** calculated from EXSY spectra in $CDCl_3$

T, K	*syn* to *anti*, s^{-1}	*anti* to *syn*, s^{-1}
253	0.137	0.156
258	0.251	0.291
263	0.442	0.561
268	0.812	1.022
273	1.558	1.664
278	2.806	3.148

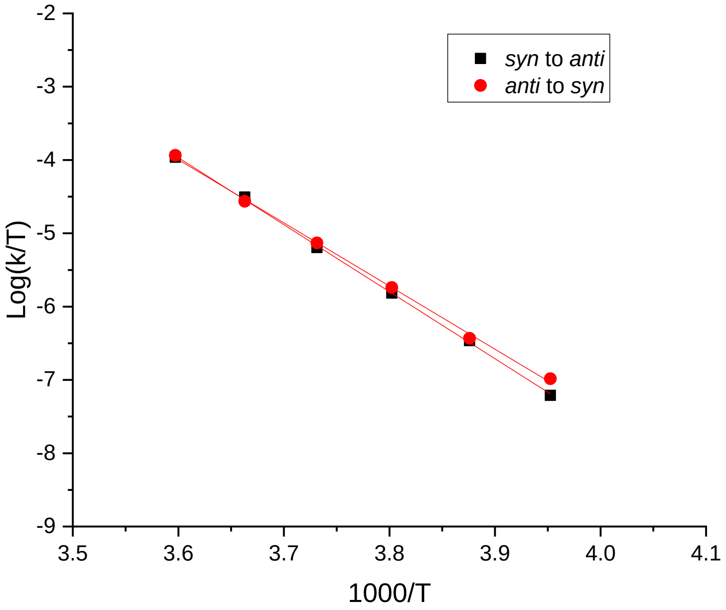

Figure S27. Eyring plot of rate constants of restricted rotation of compound **4** in $CDCl_3$

Table S27. Experimental Activation Parameters for the Exchange Processes of compound **4** calculated from ^1H EXSY spectra (signal of H-5) in $CDCl_3$

Exchange	ΔH≠(298 K)	ΔS≠(298 K)	ΔG≠(298 K)	R^2
syn to *anti*	16.4 ± 1.0	2.5 ± 3.7	15.6 ± 0.1	0.9991
anti to *syn*	16.1 ± 1.0	1.9 ± 2.8	15.6 ± 0.1	0.9995

HOmodecoupled Band-Selective NMR experiments (HOBS):

^{1}H HOBS EXSY spectra were recorded using *hobs_noesy* pulse program (refer to III. Pulse program codes for the HOBS-EXSY experiments) on a BBO probe in steps of 5 K between 263 and 293 K. The spectra were acquired using a spectral width of 2.4 kHz, 1024 x 128 complex time domain data points, mixing times in the range of 0.03 to 1.5 s and 8 scans in about 45 min. Linear prediction (32 coefficients and 256 points) in F1 was applied. The spectra were zerofilled to 4096 x 4096 data points and processed with a shifted square sine bell apodization in both dimensions. The populations were obtained by integration of 1D ^{1}H signals and the exchange rates were calculated by program EXSYCalc (MestreLab Research S.L.) from diagonal- and crosspeak integrals.

In the ^{1}H EXSY spectra of **4** the intensity of following peaks were calculated by volume integration:

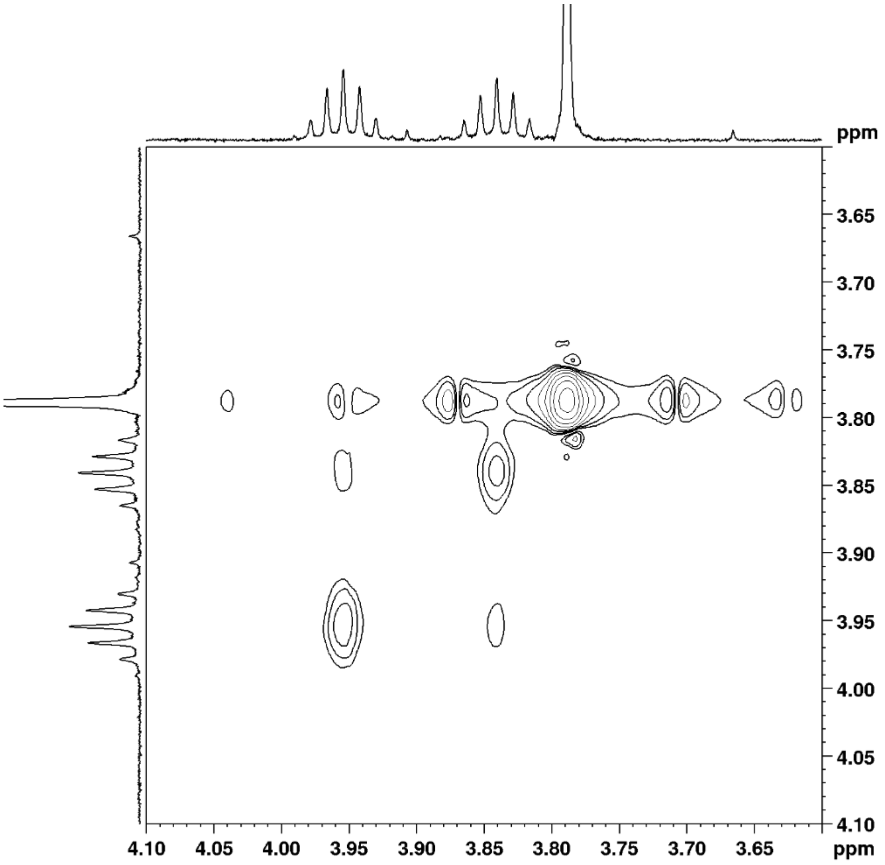

Figure S28. ^{1}H HOBS EXSY spectrum of compound **4** in $CDCl_3$ at 273 K using mixing time of 0.25 s.

Table S28. Rate constants of compound **4** calculated from ^{1}H HOBS EXSY spectra in $CDCl_3$

T, K	*syn* to *anti*, s^{-1}	*anti* to *syn*, s^{-1}
253	0.107	0.086
263	0.462	0.373
268	0.928	0.723
273	1.226	1.428

Table S29. Experimental Activation Parameters for the Exchange Processes of compound **4** calculated from ^{1}H HOBS EXSY spectra (signal of CH from *i*-Pr) in $CDCl_3$

Exchange	ΔH≠(298 K)	ΔS≠(298 K)	ΔG≠(298 K)	R^2
anti to *syn*	18.7 ± 1.2	10.9 ± 4.4	15.5 ± 0.1	0.9999
syn to *anti*	16.8 ± 1.6	3.9 ± 12.6	15.6 ± 0.1	0.9907

ΔG≠ and ΔH≠ in kcal mol^{-1} and ΔS≠ in cal mol^{-1} K^{-1}

Compound **5** in $CDCl_3$

^1H spectra were recorded on a Bruker II+ 600 instrument (BBO probe) at 600.13 MHz in steps of 5 K between 253 and 293 K (15 mg of **5** in 0.6 ml $CDCl_3$). Temperature calibration was done with B-VT 3000 unit (it was checked and calibrated with methanol and ethylene glycol reference samples). ^1H NMR spectra were acquired using a spectral width of 10 kHz, an acquisition time of 1.7 s and 32 scans, zerofilled to 64k datapoints (0.15 Hz per point) and processed without apodization. Peaks were fitted to a Lorentzian lineshape.

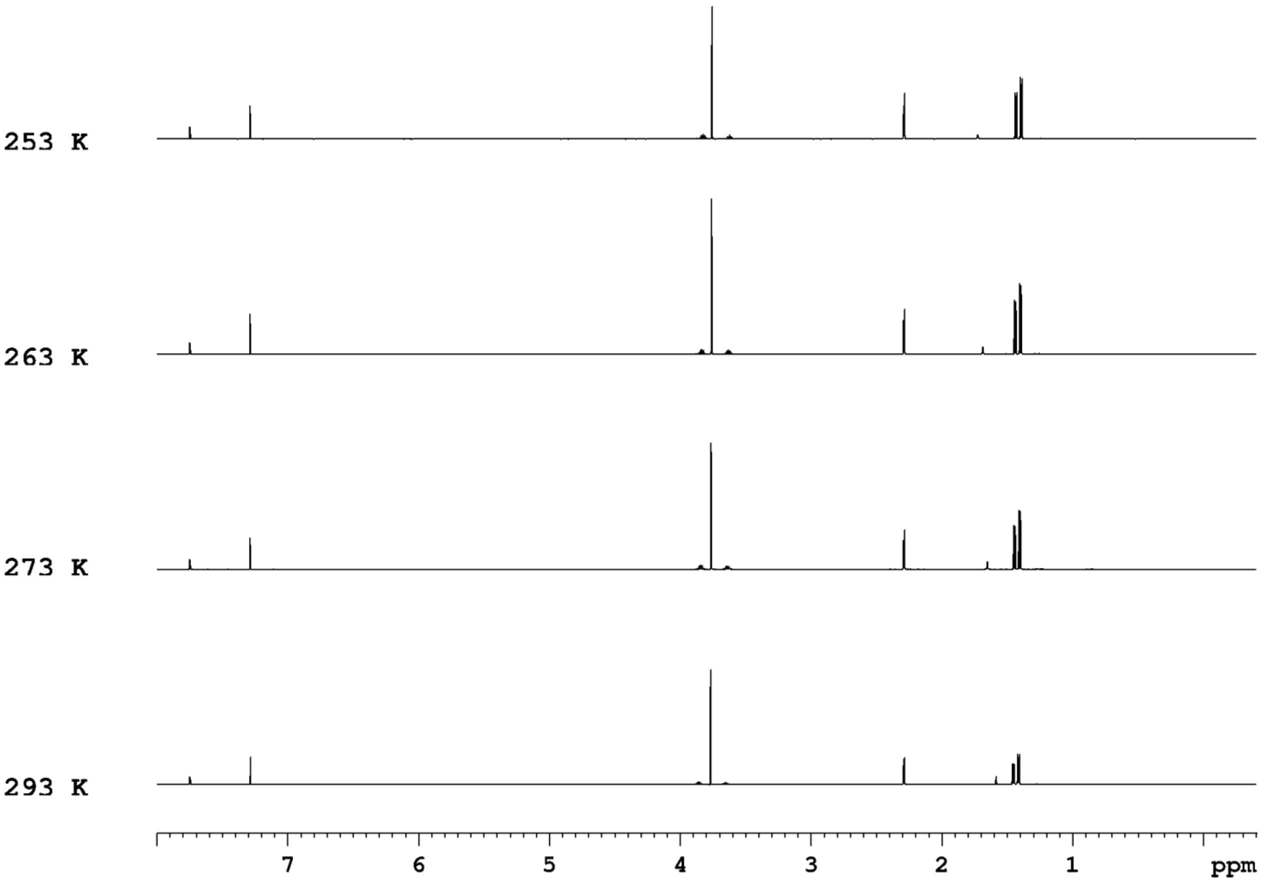

Figure S29. Stacked plots of ^1H NMR spectra of compound **5** in $CDCl_3$ at different temperatures.

^1H EXSY spectra were recorded on a BBO probe in steps of 5 K between 253 and 273 K. The spectra were acquired using a spectral width of 1.2 kHz, 2048 x 256 complex time domain data points, mixing times in the range of 0.5 to 1.5 s and 2 scans in about 45 min. Linear prediction (32 coefficients and 256 points) in F1 was applied. The spectra were zerofilled to 4096 x 4096 data points and processed with a shifted square sine bell apodization in both dimensions. The populations were obtained by integration of 1D ^1H signals and the exchange rates were calculated by program EXSYCalc (MestreLab Research S.L.) from diagonal- and crosspeak integrals.

In the ^1H EXSY spectra of **5** the intensity of following peaks were calculated by volume integration:

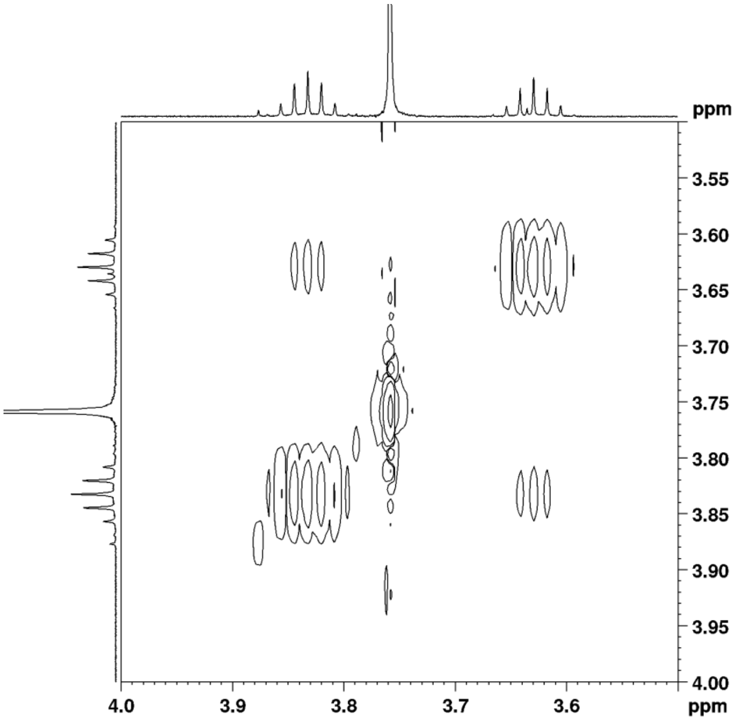

Figure S30. NOESY spectrum of compound **5** in $CDCl_3$ at 263 K using mixing time of 1.0 s.

Errors analysis

The total errors $\underline{\boldsymbol{E_{tot}}}$, quoted in activation parameters tables are calculated according to the expression $E_{tot} = \sqrt{E_S^2 + E_{kT}^2}$, where $\boldsymbol{E_S}$ is the statistical error based on scattering of the data in the Eyring plot while $\boldsymbol{E_{kT}}$ is computed using error propagation equations as derived by Binsch [G. Binsch, in ***Dynamic NMR Spectroscopy,*** edited by **L.** M. Jackman and F. A. Cotton, **p.** 45. Academic Press, New York (1975)] and Heinzer and Oth [J. Heinzer and J. F. M. Oth, ***Helv. Chim.Acta*** 64, 258 (1981)], in which errors due to both the calculated rate constants and the measured temperature are taken into account. The absolute temperature errors were assumed to be $\boldsymbol{\sigma T}$ = ±0.5 K and the maximum relative error in rate constants was taken to be $\boldsymbol{\sigma k/k}$ = ±10%.

Table S30. Rate constants of compound **5** calculated from EXSY spectra in $CDCl_3$

T, K	*syn* to *anti*, s^{-1}	*anti* to *syn*, s^{-1}
253	0.027	0.023
258	0.054	0.046
263	0.100	0.084
268	0.190	0.145
273	0.352	0.265

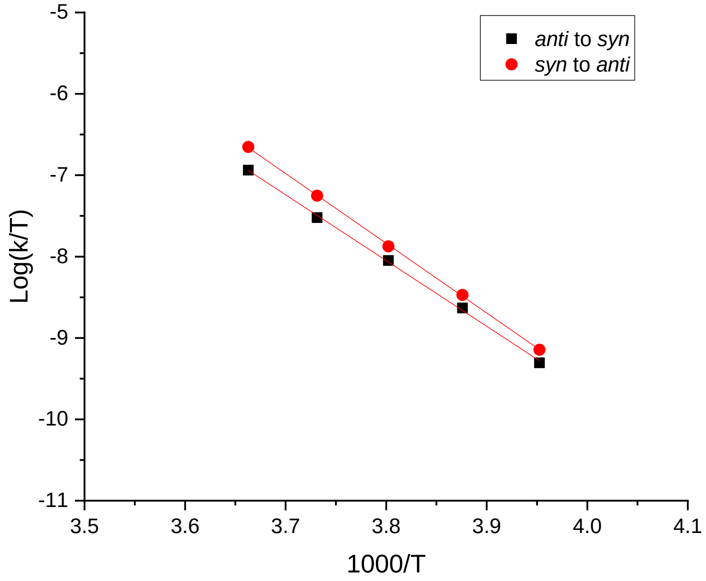

Figure S31. Eyring plot of rate constants of restricted rotation of compound **5** in $CDCl_3$

Table S31. Experimental Activation Parameters for the Exchange Processes of compound **5** calculated from ^{1}H EXSY spectra (signal of CH from *i*-Pr) in $CDCl_3$

Exchange	ΔH≠(298 K)	ΔS≠(298 K)	ΔG≠(298 K)	R^2
syn to *anti*	17.0 ± 1.2	1.9 ± 3.9	16.4 ± 0.1	0.9999
anti to *syn*	16.0 ± 1.1	-2.2 ± 6.5	16.7 ± 0.1	0.9996

ΔG≠ and ΔH≠ in kcal mol^{-1} and ΔS≠ in cal mol^{-1} K^{-1}

HOmodecoupled Band-Selective NMR experiments (HOBS):

^{1}H HOBS EXSY spectra were recorded using *hobs_noesy* pulse program (refer to III. Pulse program codes for the HOBS-EXSY experiments) on a BBO probe in steps of 5 K between 263 and 293 K. The spectra were acquired using a spectral width of 2.4 kHz, 1024 x 128 complex time domain data points, mixing times in the range of 0.03 to 1.5 s and 8 scans in about 45 min. Linear prediction (32 coefficients and 256 points) in F1 was applied. The spectra were zerofilled to 4096 x 4096 data points and processed with a shifted square sine bell apodization in both dimensions. The populations were obtained by integration of 1D ^{1}H signals and the exchange rates were calculated by program EXSYCalc (MestreLab Research S.L.) from diagonal- and crosspeak integrals.

In the ^{1}H EXSY spectra of **5** the intensity of following peaks were calculated by volume integration:

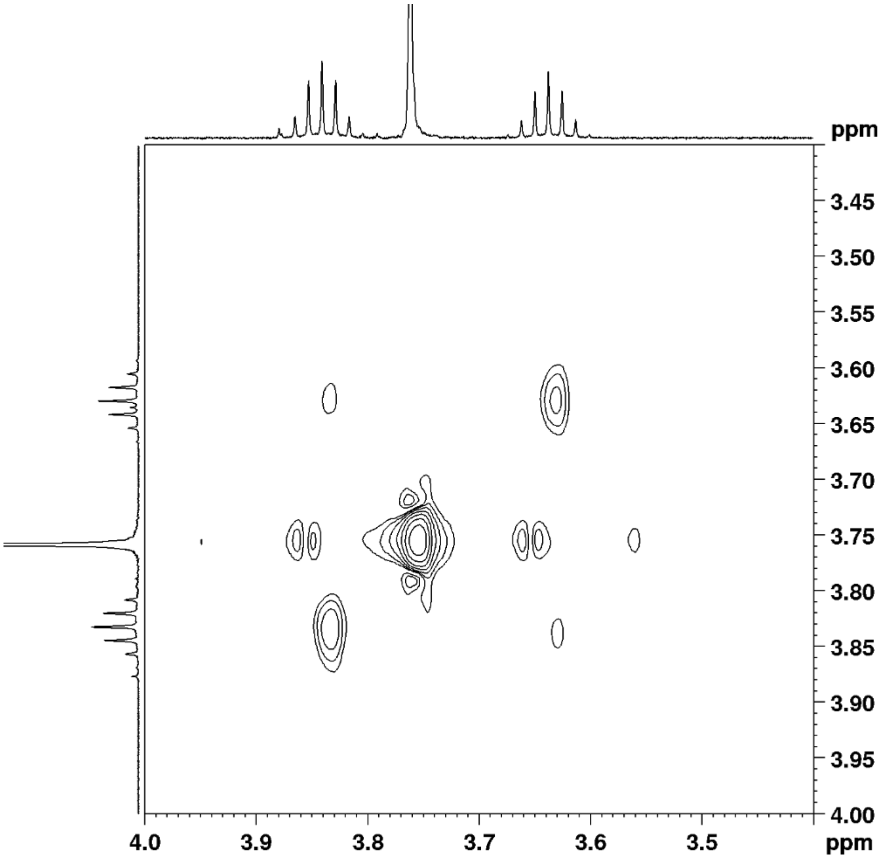

Figure S32. ^{1}H HOBS EXSY spectrum of compound **5** in $CDCl_3$ at 273 K using mixing time of 1.0 s.

Table S32. Rate constants of compound **5** calculated from ^{1}H HOBS EXSY spectra in $CDCl_3$

T, K	*syn* to *anti*, s^{-1}	*anti* to *syn*, s^{-1}
263	0.086	0.073
268	0.176	0.142
273	0.307	0.293
278	0.561	0.436
283	0.994	0.770
288	1.572	1.443
293	2.466	2.136

Table S33. Experimental Activation Parameters for the Exchange Processes of compound **5** calculated from ^{1}H HOBS EXSY spectra (signal of CH from *i*-Pr) in $CDCl_3$

Exchange	ΔH≠(298 K)	ΔS≠(298 K)	ΔG≠(298 K)	R^2
syn to *anti*	16.6 ± 0.9	0.0 ± 1.2	16.6 ± 0.1	0.9990
anti to *syn*	16.7 ± 0.9	0.0 ± 2.7	16.7 ± 0.1	0.9982

ΔG≠ and ΔH≠ in kcal mol^{-1} and ΔS≠ in cal mol^{-1} K^{-1}

Compound **6** in $CDCl_3$

^1H spectra were recorded on a Bruker II+ 600 instrument (BBO probe) at 600.13 MHz in steps of 5 K between 253 and 293 K (15 mg of **6** in 0.6 ml $CDCl_3$). Temperature calibration was done with B-VT 3000 unit (it was checked and calibrated with methanol and ethylene glycol reference samples). ^1H NMR spectra were acquired using a spectral width of 10 kHz, an acquisition time of 1.7 s and 32 scans, zerofilled to 64k datapoints (0.15 Hz per point) and processed without apodization. Peaks were fitted to a Lorentzian lineshape.

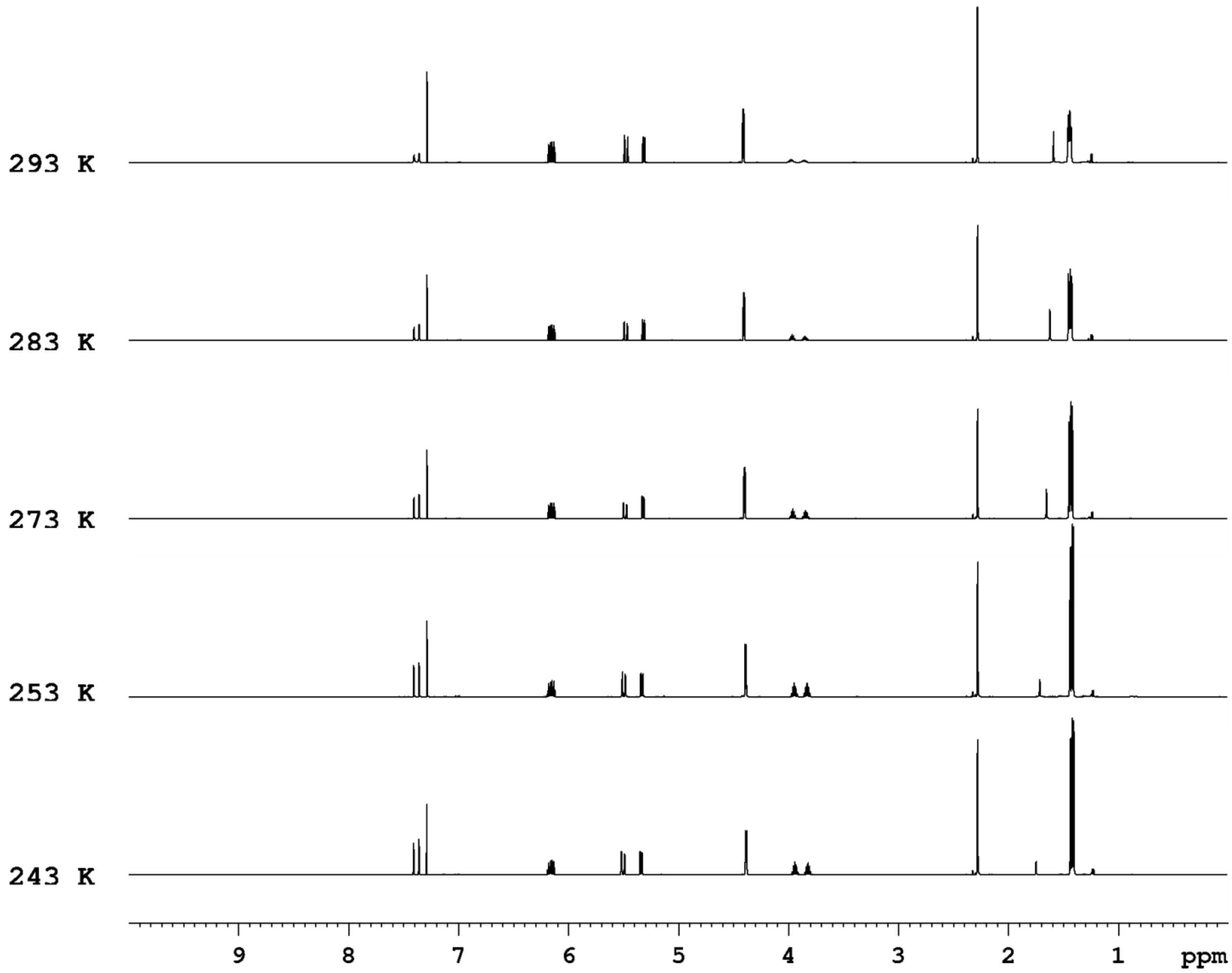

Figure S33. Stacked plots of ^1H NMR spectra of compound **6** in $CDCl_3$ at different temperatures.

^1H EXSY spectra were recorded on a BBO probe in steps of 10 K between 243 and 283 K. The spectra were acquired using a spectral width of 1.2 kHz, 2048 x 256 complex time domain data points, mixing times in the range of 0.05 to 1.5 s and 2 scans in about 45 min. Linear prediction (32 coefficients and 256 points) in F1 was applied. The spectra were zerofilled to 4096 x 4096 data points and processed with a shifted square sine bell apodization in both dimensions. The populations were obtained by integration of 1D ^1H

signals and the exchange rates were calculated by program EXSYCalc (MestreLab Research S.L.) from diagonal- and crosspeak integrals.

In the ^{1}H EXSY spectra of **6** the intensity of following peaks were calculated by volume integration:

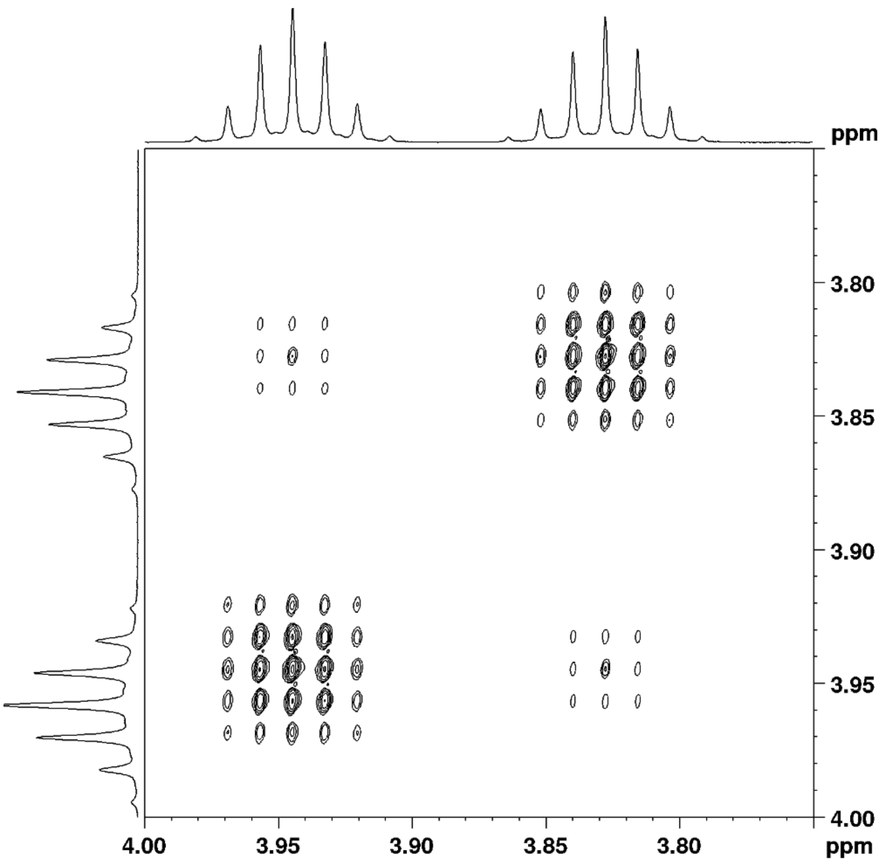

Figure S34. NOESY spectrum of compound **6** in $CDCl_3$ at 253 K using mixing time of 1.0 s.

Errors analysis

The total errors E_{tot}, quoted in activation parameters tables are calculated according to the expression $E_{tot} = \sqrt{E_S^2 + E_{kT}^2}$, where E_S is the statistical error based on scattering of the data in the Eyring plot while E_{kT} is computed using error propagation equations as derived by Binsch [G. Binsch, in ***Dynamic NMR Spectroscopy,*** edited by **L.** M. Jackman and F. A. Cotton, **p.** 45. Academic Press, New York (1975)] and Heinzer and Oth [J. Heinzer and J. F. M. Oth, ***Helv. Chim.Acta*** 64, 258 (1981)], in which errors due to both the calculated rate constants and the measured temperature are taken into account. The absolute temperature errors were assumed to be σT = ±0.5 K and the maximum relative error in rate constants was taken to be $\sigma k/k$ = ±10%.

Table S34. Rate constants of compound **6** calculated from EXSY spectra in $CDCl_3$

T, K	*syn* to *anti*, s^{-1}	*anti* to *syn*, s^{-1}
243	0.044	0.049
253	0.151	0.163
263	0.479	0.566
273	1.513	1.737
283	4.362	5.049

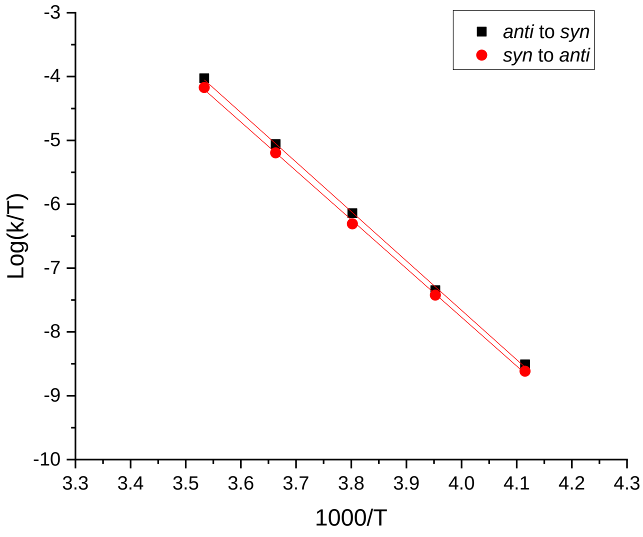

Figure S35. Eyring plot of rate constants of restricted rotation of compound **6** in $CDCl_3$

Table S35. Experimental Activation Parameters for the Exchange Processes of compound **6** calculated from ^1H EXSY spectra (signal of CH from *i*-Pr) in $CDCl_3$

Exchange	$\Delta H^{\neq}$(298 K)	$\Delta S^{\neq}$(298 K)	$\Delta G^{\neq}$(298 K)	R^2
syn to *anti*	15.2 ± 0.6	-1.9 ± 2.2	15.8 ± 0.1	0.9998
anti to *syn*	15.4 ± 0.6	-0.9 ± 1.8	15.6 ± 0.1	0.9998

$\Delta G^{\neq}$ and $\Delta H^{\neq}$ in kcal mol^{-1} and $\Delta S^{\neq}$ in cal mol^{-1} K^{-1}

HOmodecoupled Band-Selective NMR experiments (HOBS):

^1H HOBS EXSY spectra were recorded using *hobs_noesy* pulse program (refer to III. Pulse program codes for the HOBS-EXSY experiments) on a BBO probe in steps of 10 K between 253 and 283 K. The spectra were acquired using a spectral width of 2.4 kHz, 1024 x 128 complex time domain data points, mixing

times in the range of 0.03 to 1.5 s and 8 scans in about 45 min. Linear prediction (32 coefficients and 256 points) in F1 was applied. The spectra were zerofilled to 4096 x 4096 data points and processed with a shifted square sine bell apodization in both dimensions. The populations were obtained by integration of 1D ^{1}H signals and the exchange rates were calculated by program EXSYCalc (MestreLab Research S.L.) from diagonal- and crosspeak integrals.

In the ^{1}H EXSY spectra of **6** the intensity of following peaks were calculated by volume integration:

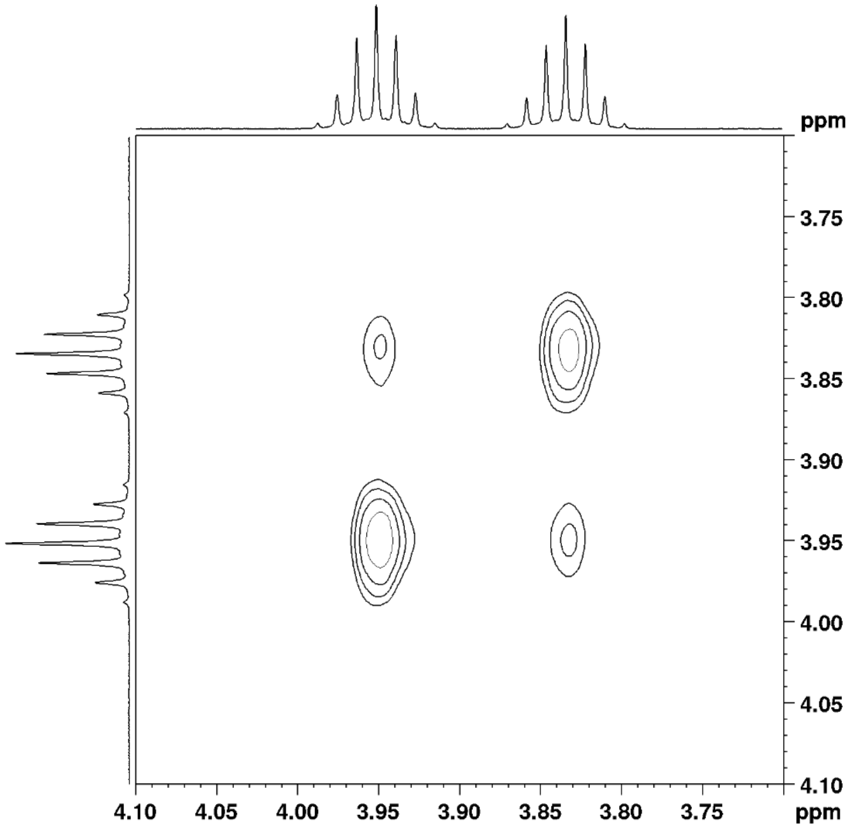

Figure S36. ^{1}H HOBS EXSY spectrum of compound **6** in $CDCl_3$ at 263 K using mixing time of 0.5 s.

Table S36. Rate constants of compound **6** calculated from ^{1}H HOBS EXSY spectra in $CDCl_3$

T, K	*syn* to *anti*, s^{-1}	*anti* to *syn*, s^{-1}
253	0.108	0.149
263	0.431	0.479
273	1.269	1.537
283	3.878	4.031

Table S37. Experimental Activation Parameters for the Exchange Processes of compound **6** calculated from ^{1}H HOBS EXSY spectra (signal of CH from *i*-Pr) in $CDCl_3$

Exchange	$\Delta H^{\neq}$(298K)	$\Delta S^{\neq}$(298K)	$\Delta G^{\neq}$(298K)	R^2
syn to *anti*	16.3 ± 0.8	1.9 ± 2.5	15.7 ± 0.1	0.9994
anti to *syn*	15.2 ± 0.8	-1.9 ± 2.4	15.8 ± 0.1	0.9998

$\Delta G^{\neq}$ and $\Delta H^{\neq}$ in kcal mol^{-1} and $\Delta S^{\neq}$ in cal mol^{-1} K^{-1}

Compound **7** in $CDCl_3$

^1H spectra were recorded on a Bruker II+ 600 instrument (BBO probe) at 600.13 MHz in steps of 5 K between 253 and 293 K (15 mg of **7** in 0.6 ml $CDCl_3$). Temperature calibration was done with B-VT 3000 unit (it was checked and calibrated with methanol and ethylene glycol reference samples). ^1H NMR spectra were acquired using a spectral width of 10 kHz, an acquisition time of 1.7 s and 32 scans, zerofilled to 64k datapoints (0.15 Hz per point) and processed without apodization. Peaks were fitted to a Lorentzian lineshape.

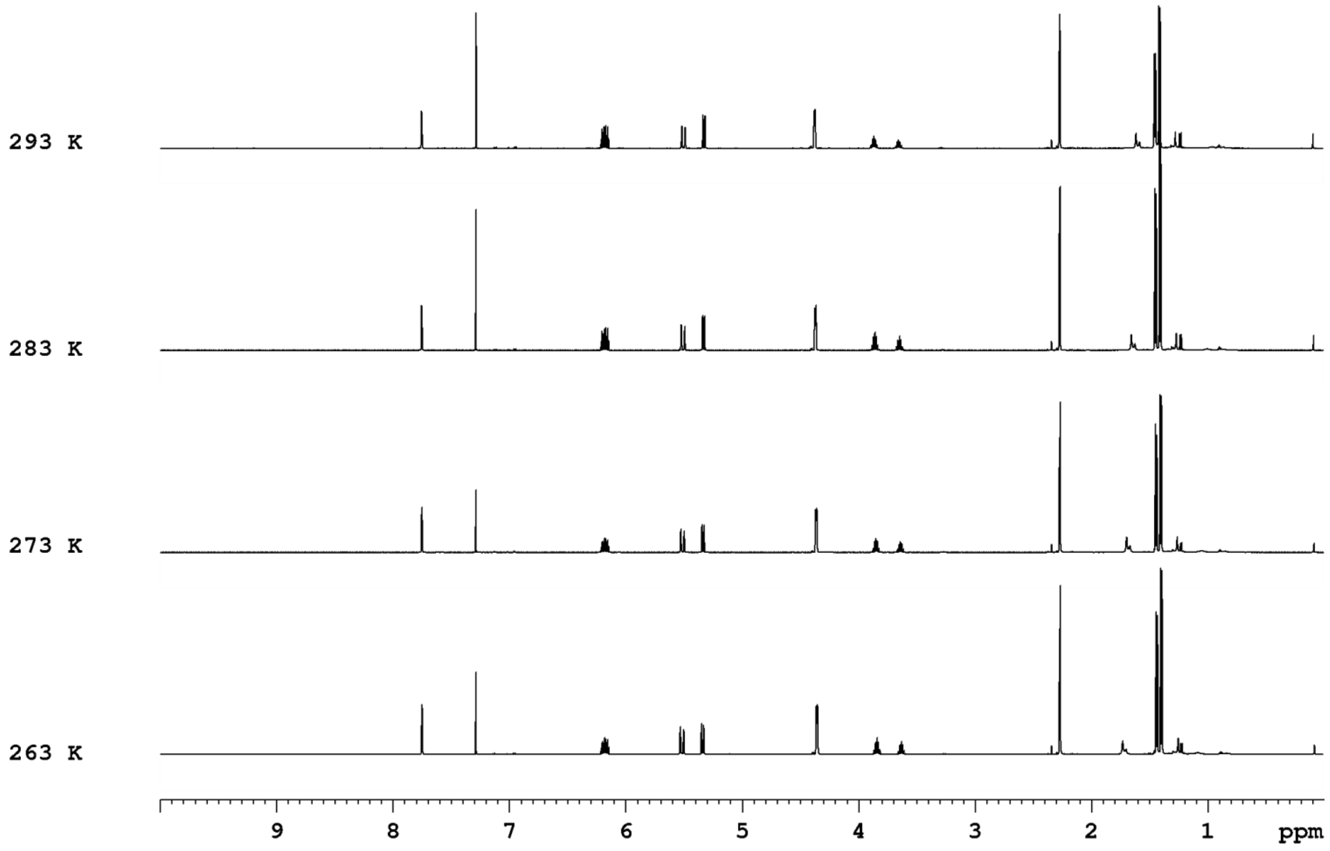

Figure S37. Stacked plots of ^1H NMR spectra of compound **7** in $CDCl_3$ at different temperatures.

^1H EXSY spectra were recorded on a BBO probe in steps of 5 K between 253 and 273 K. The spectra were acquired using a spectral width of 1.2 kHz, 2048 x 256 complex time domain data points, mixing times in the range of 0.05 to 1.0 s and 2 scans in about 45 min. Linear prediction (32 coefficients and 256 points) in F1 was applied. The spectra were zerofilled to 4096 x 4096 data points and processed with a shifted square sine bell apodization in both dimensions. The populations were obtained by integration of 1D ^1H signals and the exchange rates were calculated by program EXSYCalc (MestreLab Research S.L.) from diagonal- and crosspeak integrals.

In the ^{1}H EXSY spectra of **7** the intensity of following peaks were calculated by volume integration:

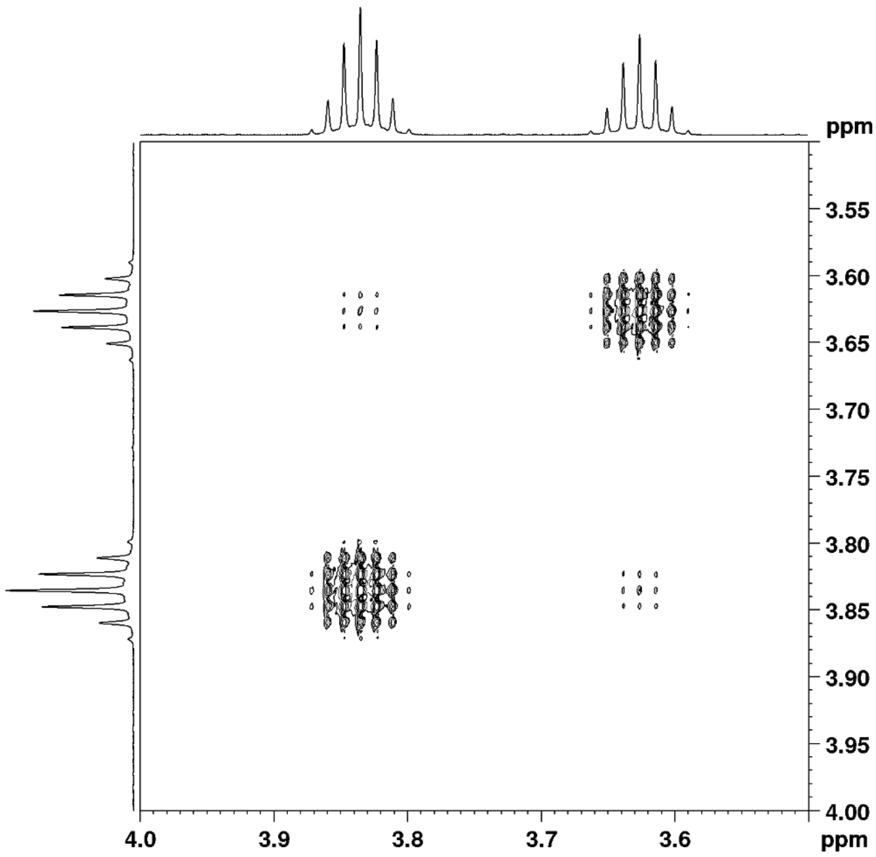

Figure S38. NOESY spectrum of compound **7** in $CDCl_3$ at 253K using mixing time of 1.0 s.

Errors analysis

The total errors ***E_{tot}***, quoted in activation parameters tables are calculated according to the expression $E_{tot} = \sqrt{E_S^2 + E_{kT}^2}$, where ***E_S*** is the statistical error based on scattering of the data in the Eyring plot while ***E_{kT}*** is computed using error propagation equations as derived by Binsch [G. Binsch, in ***Dynamic NMR Spectroscopy,*** edited by **L.** M. Jackman and F. A. Cotton, **p.** 45. Academic Press, New York (1975)] and Heinzer and Oth [J. Heinzer and J. F. M. Oth, ***Helv. Chim.Acta*** 64, 258 (1981)], in which errors due to both the calculated rate constants and the measured temperature are taken into account. The absolute temperature errors were assumed to be ***σT*** = ±0.5 K and the maximum relative error in rate constants was taken to be ***$\sigma k/k$*** = ±10%.

Table S38. Rate constants of compound **7** calculated from EXSY spectra in $CDCl_3$

T, K	*syn* to *anti*, s^{-1}	*anti* to *syn*, s^{-1}
253	0.024	0.027
258	0.038	0.062
263	0.078	0.112
268	0.152	0.200
273	0.268	0.347

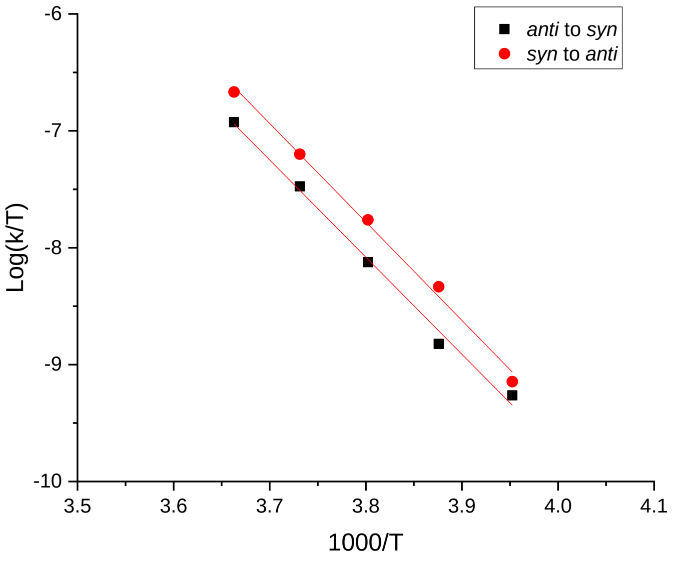

Figure S39. Eyring plot of rate constants of restricted rotation of compound **7** in $CDCl_3$

Table S39. Experimental Activation Parameters for the Exchange Processes of compound **7** calculated from ^1H EXSY spectra (signal of CH from *i*-Pr) in $CDCl_3$

Exchange	ΔH≠(298K)	ΔS≠(298K)	ΔG≠(298K)	R^2
syn to *anti*	16.5 ± 1.3	-0.5 ± 2.4	16.7 ± 0.1	0.9970
anti to *syn*	16.7 ± 1.2	0.9 ± 3.0	16.4 ± 0.1	0.9977

ΔG≠ and ΔH≠ in kcal mol^{-1} and ΔS≠ in cal mol^{-1} K^{-1}

HOmodecoupled Band-Selective NMR experiments (HOBS):

^1H HOBS EXSY spectra were recorded using *hobs_noesy* pulse program (refer to III. Pulse program codes for the HOBS-EXSY experiments) on a BBO probe in steps of 5 K between 263 and 293 K. The spectra were acquired using a spectral width of 2.4 kHz, 1024 x 128 complex time domain data points, mixing times in the range of 0.03 to 1.5 s and 8 scans in about 45 min. Linear prediction (32 coefficients and 256

points) in F1 was applied. The spectra were zerofilled to 4096 x 4096 data points and processed with a shifted square sine bell apodization in both dimensions. The populations were obtained by integration of 1D ^{1}H signals and the exchange rates were calculated by program EXSYCalc (MestreLab Research S.L.) from diagonal- and crosspeak integrals.

In the ^{1}H EXSY spectra of **7** the intensity of following peaks were calculated by volume integration:

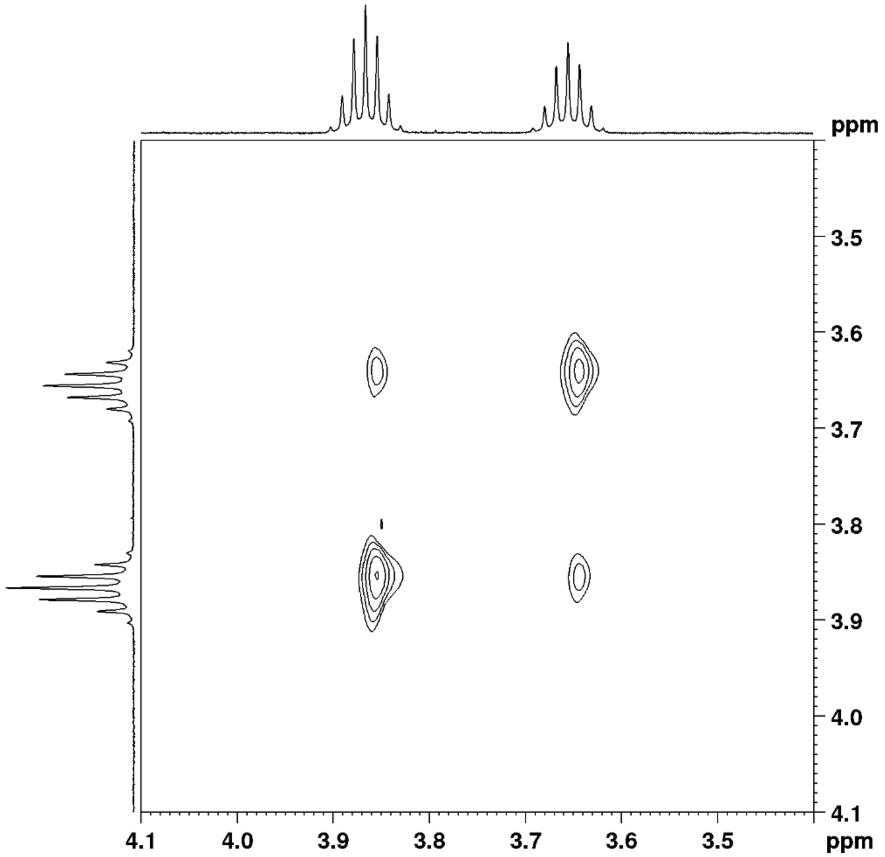

Figure S40. ^{1}H HOBS EXSY spectrum of compound **7** in $CDCl_3$ at 273 K using mixing time of 0.25 s.

Table S40. Rate constants of compound **7** calculated from ^{1}H HOBS EXSY spectra in $CDCl_3$

T, K	*syn* to *anti*,s^{-1}	*anti* to *syn*,s^{-1}
263	0.081	0.110
268	0.153	0.234
273	0.275	0.345
278	0.468	0.720
283	0.808	1.112
288	1.365	1.886
293	2.250	3.271

Table S41. Experimental Activation Parameters for the Exchange Processes of compound **7** calculated from ^{1}H HOBS EXSY spectra (signal of CH from *i*-Pr) in $CDCl_3$

Exchange	$\Delta H^{\neq}$(298 K)	$\Delta S^{\neq}$(298 K)	$\Delta G^{\neq}$(298 K)	R^2
anti to *syn*	16.4 ± 0.9	-0.1 ± 1.4	16.4 ± 0.1	0.9982
syn to *anti*	16.3 ± 0.8	-1.1 ± 3.9	16.7 ± 0.1	0.9999

$\Delta G^{\neq}$ and $\Delta H^{\neq}$ in kcal mol^{-1} and $\Delta S^{\neq}$ in cal mol^{-1} K^{-1}

Compound **8** in $CDCl_3$

^{1}H spectra were recorded on a Bruker II+ 600 instrument (BBO probe) at 600.13 MHz in steps of 5 K between 253 and 293 K (15 mg of **8** in 0.6 ml $CDCl_3$). Temperature calibration was done with B-VT 3000 unit (it was checked and calibrated with methanol and ethylene glycol reference samples). ^{1}H NMR spectra were acquired using a spectral width of 10 kHz, an acquisition time of 1.7 s and 32 scans, zerofilled to 64k datapoints (0.15 Hz per point) and processed without apodization. Peaks were fitted to a Lorentzian lineshape.

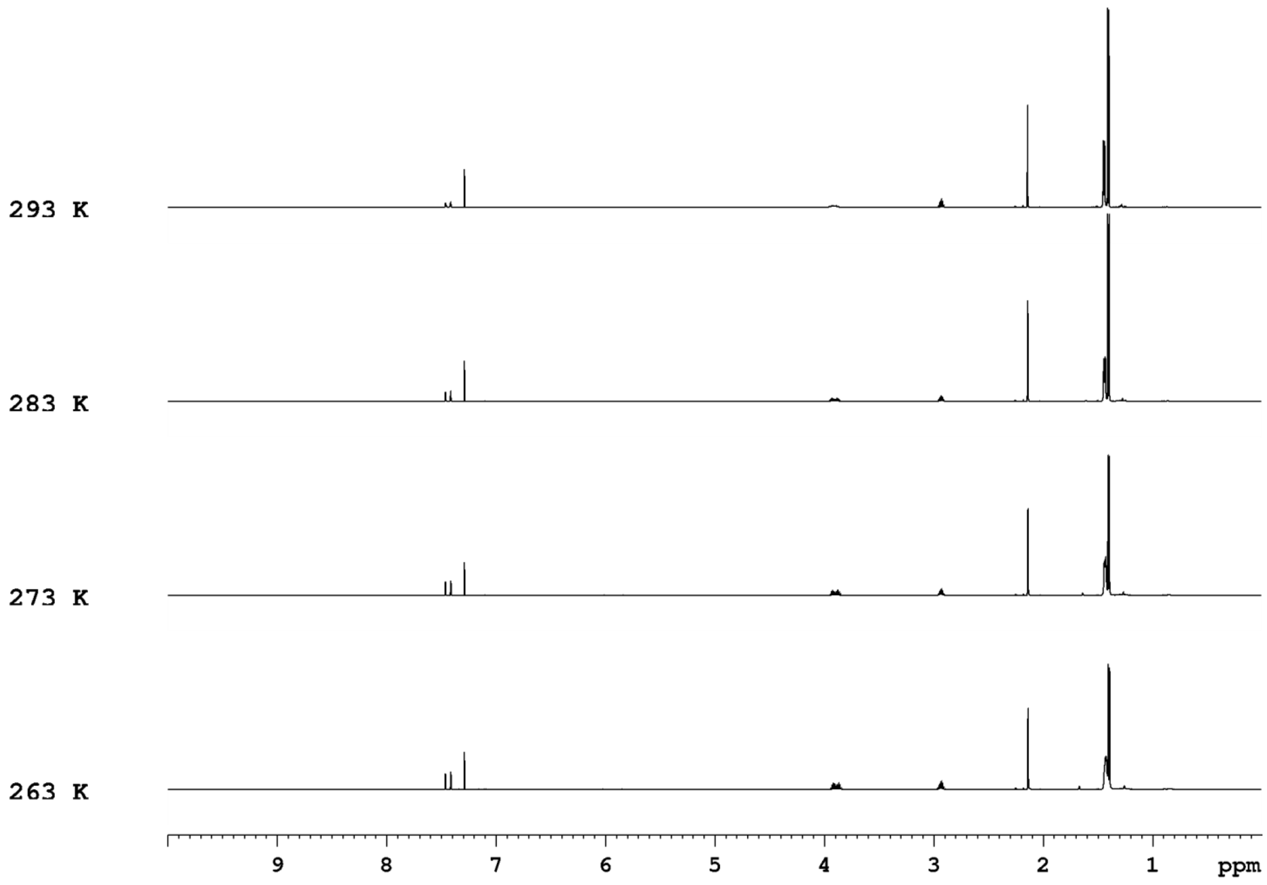

Figure S41. Stacked plots of ^{1}H NMR spectra of compound **8** in $CDCl_3$ at different temperatures.

^{1}H EXSY spectra were recorded on a BBO probe in steps of 5 K between 253 and 273 K. The spectra were acquired using a spectral width of 1.2 kHz, 2048 x 256 complex time domain data points, mixing times in the range of 0.05 to 1.0 s and 2 scans in about 45 min. Linear prediction (32 coefficients and 256 points) in F1 was applied. The spectra were zerofilled to 4096 x 4096 data points and processed with a shifted square sine bell apodization in both dimensions. The populations were obtained by integration of 1D ^{1}H signals and the exchange rates were calculated by program EXSYCalc (MestreLab Research S.L.) from diagonal- and crosspeak integrals.

In the ^{1}H EXSY spectra of **8** the intensity of following peaks were calculated by volume integration:

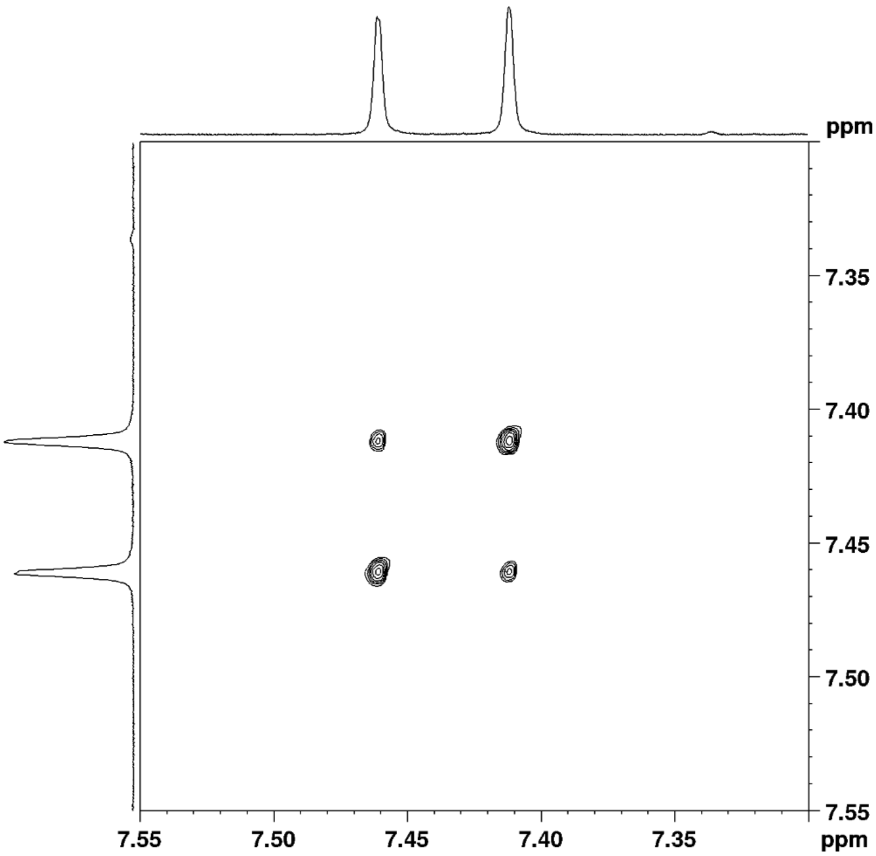

Figure S42. NOESY spectrum of compound **8** in $CDCl_3$ at 263 K using mixing time of 0.6 s.

Errors analysis

The total errors $\underline{\boldsymbol{E_{tot}}}$, quoted in activation parameters tables are calculated according to the expression $E_{tot} = \sqrt{E_S^2 + E_{kT}^2}$, where $\boldsymbol{E_S}$ is the statistical error based on scattering of the data in the Eyring plot while $\boldsymbol{E_{kT}}$ is computed using error propagation equations as derived by Binsch [G. Binsch, in ***Dynamic NMR Spectroscopy,*** edited by **L.** M. Jackman and F. A. Cotton, **p.** 45. Academic Press, New York (1975)] and Heinzer and Oth [J. Heinzer and J. F. M. Oth, ***Helv. Chim.Acta*** 64, 258 (1981)], in which errors due to both the calculated rate constants and the measured temperature are taken into account. The absolute temperature errors were assumed to be $\boldsymbol{\sigma T}$ = ±0.5 K and the maximum relative error in rate constants was taken to be $\boldsymbol{\sigma k/k}$ = ±10%.

Table S42. Rate constants of compound **8** calculated from EXSY spectra in $CDCl_3$

T, K	*syn* to *anti*,s^{-1}	*anti* to *syn*,s^{-1}
253	0.152	0.171
258	0.287	0.316
263	0.520	0.587
268	0.948	1.048
273	1.651	1.783

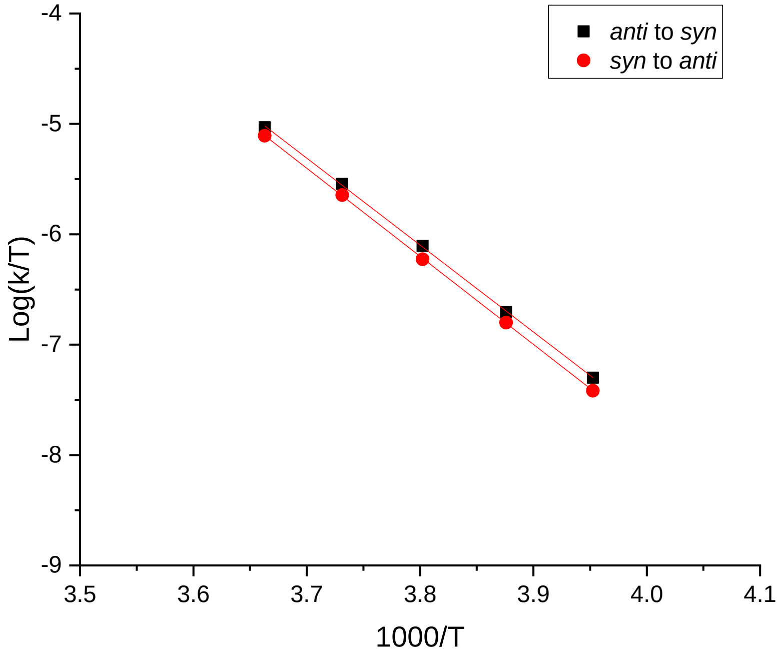

Figure S43. Eyring plot of rate constants of restricted rotation of compound **8** in $CDCl_3$

Table S43. Experimental Activation Parameters for the Exchange Processes of compound **8** calculated from ^1H EXSY spectra (signal of H-5) in $CDCl_3$

Exchange	ΔH≠(298 K)	ΔS≠(298 K)	ΔG≠(298 K)	R^2
syn to *anti*	15.8 ± 1.1	0.7 ± 4.6	15.6 ± 0.1	0.9999
anti to *syn*	15.6 ± 1.1	0.1 ± 2.7	15.6 ± 0.1	0.9995

ΔG≠ and ΔH≠ in kcal mol^{-1} and ΔS≠ in cal mol^{-1} K^{-1}

HOmodecoupled Band-Selective NMR experiments (HOBS):

^{1}H HOBS EXSY spectra were recorded using *hobs_noesy* pulse program (refer to III. Pulse program codes for the HOBS-EXSY experiments) on a BBO probe in steps of 5 K between 263 and 293 K. The spectra were acquired using a spectral width of 2.4 kHz, 1024 x 128 complex time domain data points, mixing times in the range of 0.03 to 1.5 s and 8 scans in about 45 min. Linear prediction (32 coefficients and 256 points) in F1 was applied. The spectra were zerofilled to 4096 x 4096 data points and processed with a shifted square sine bell apodization in both dimensions. The populations were obtained by integration of 1D ^{1}H signals and the exchange rates were calculated by program EXSYCalc (MestreLab Research S.L.) from diagonal- and crosspeak integrals.

In the ^{1}H EXSY spectra of **8** the intensity of following peaks were calculated by volume integration:

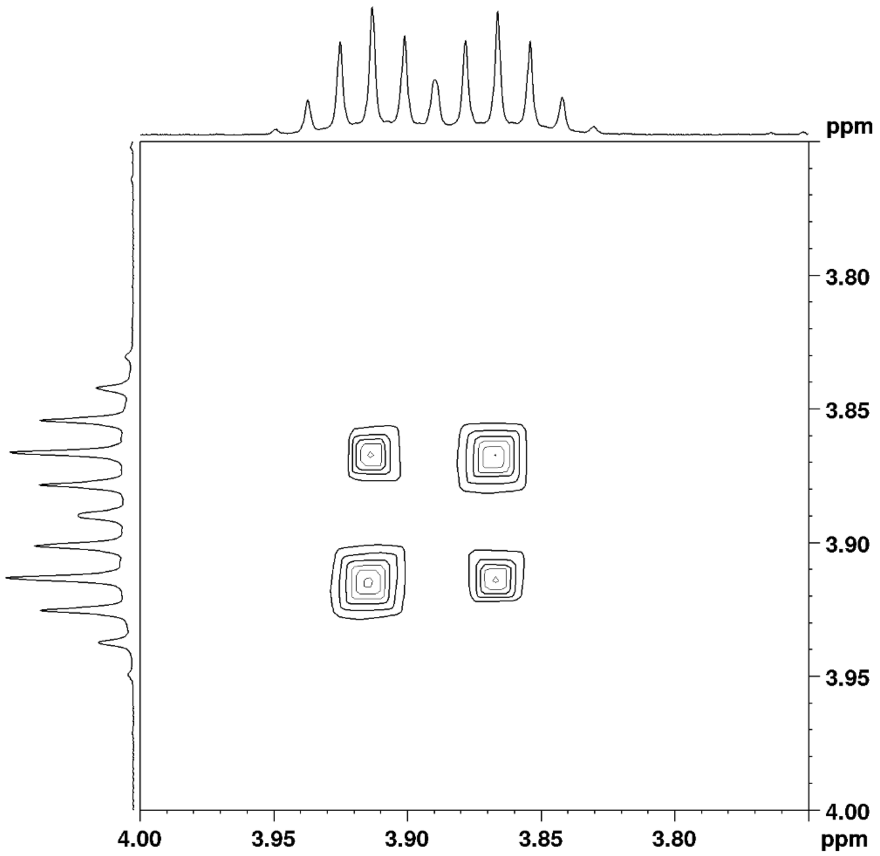

Figure S44. ^{1}H HOBS EXSY spectrum of compound **8** in $CDCl_3$ at 273 K using mixing time of 0.25 s.

Table S44. Rate constants of compound **8** calculated from ^{1}H HOBS EXSY spectra in $CDCl_3$

T, K	*syn* to *anti*, s^{-1}	*anti* to *syn*, s^{-1}
263	0.628	0.678
268	1.401	1.326
273	2.331	2.174
278	5.161	4.817
283	9.293	7.936
288	11.403	12.836

Table S45. Experimental Activation Parameters for the Exchange Processes of compound **8** calculated from ^{1}H HOBS EXSY spectra (signal of CH from *i*-Pr) in $CDCl_3$

Exchange	$\Delta H^{\neq}$(298 K)	$\Delta S^{\neq}$(298 K)	$\Delta G^{\neq}$(298 K)	R^2
anti to *syn*	17.4 ± 1.1	7.2 ± 4.5	15.3 ± 0.1	0.9980
syn to *anti*	17.5 ± 1.4	7.6 ± 6.4	15.2 ± 0.1	0.9917

$\Delta G^{\neq}$ and $\Delta H^{\neq}$ in kcal mol^{-1} and $\Delta S^{\neq}$ in cal mol^{-1} K^{-1}

Compound **9** in $CDCl_3$

^1H spectra were recorded on a Bruker II+ 600 instrument (BBO probe) at 600.13 MHz in steps of 5 K between 253 and 293 K (15 mg of **9** in 0.6 ml $CDCl_3$). Temperature calibration was done with B-VT 3000 unit (it was checked and calibrated with methanol and ethylene glycol reference samples). ^1H NMR spectra were acquired using a spectral width of 10 kHz, an acquisition time of 1.7 s and 32 scans, zerofilled to 64k datapoints (0.15 Hz per point) and processed without apodization. Peaks were fitted to a Lorentzian lineshape.

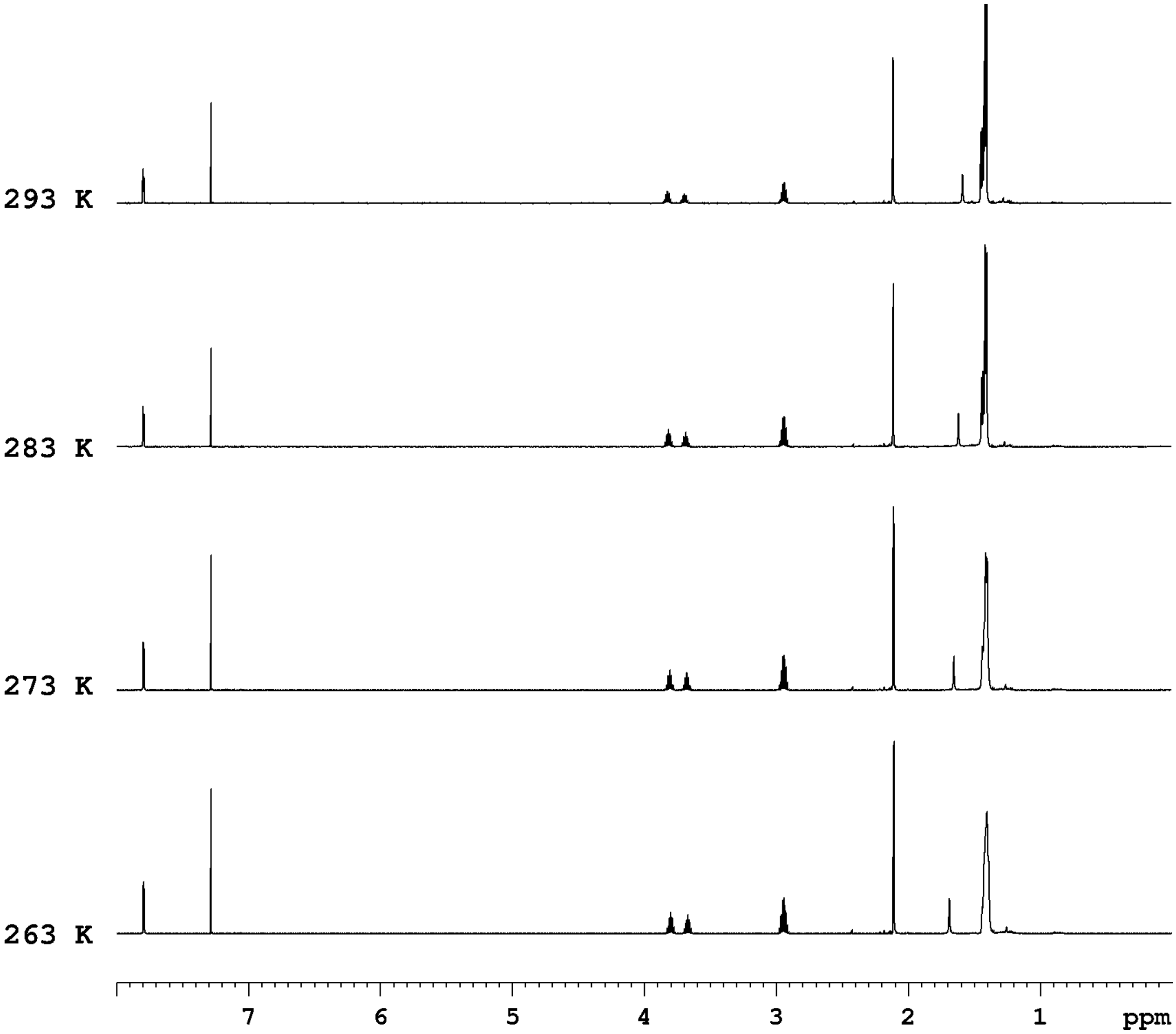

Figure S45. Stacked plots of ^1H NMR spectra of compound **9** in $CDCl_3$ at different temperatures.

^1H EXSY spectra were recorded on a BBO probe in steps of 5 K between 253 and 273 K. The spectra were acquired using a spectral width of 1.2 kHz, 2048 x 256 complex time domain data points, mixing times in the range of 0.05 to 1.0 s and 2 scans in about 45 min. Linear prediction (32 coefficients and 256 points) in F1 was applied. The spectra were zerofilled to 4096 x 4096 data points and processed with a shifted

square sine bell apodization in both dimensions. The populations were obtained by integration of 1D ^{1}H signals and the exchange rates were calculated by program EXSYCalc (MestreLab Research S.L.) from diagonal- and crosspeak integrals.

In the ^{1}H EXSY spectra of **6** the intensity of following peaks were calculated by volume integration:

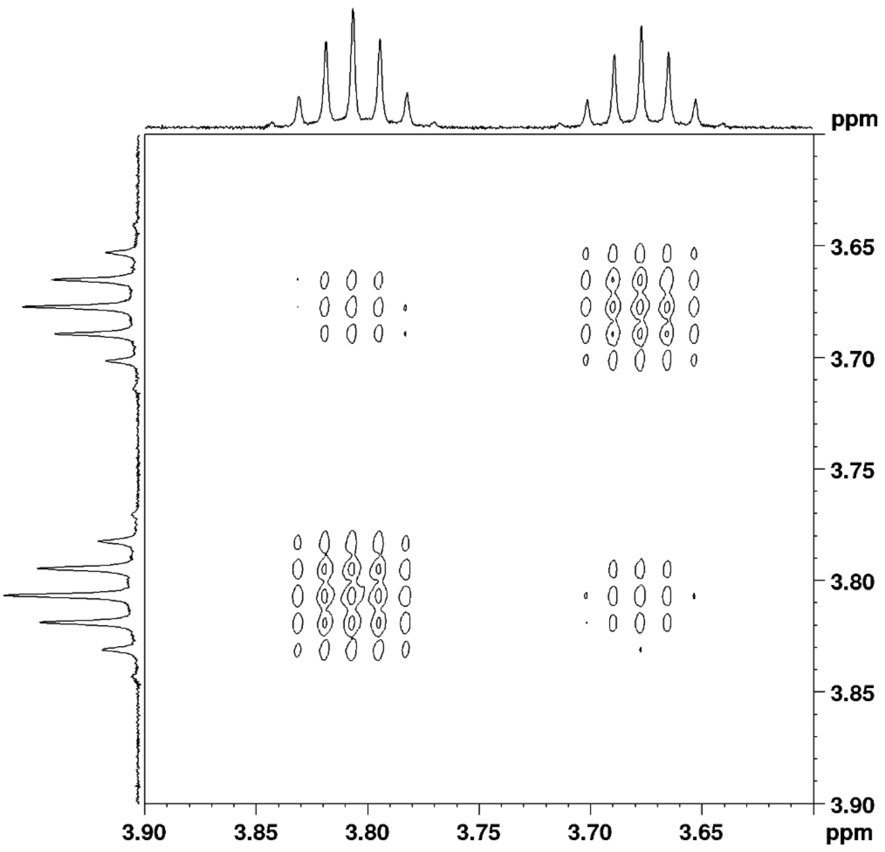

Figure S46. NOESY spectrum of compound **9** in $CDCl_3$ at 253K using mixing time of 1.0 s.

Errors analysis

The total errors ***E*** $_{tot}$, quoted in activation parameters tables are calculated according to the expression $E_{tot} = \sqrt{E_S^2 + E_{kT}^2}$, where E_S is the statistical error based on scattering of the data in the Eyring plot while E_{kT} is computed using error propagation equations as derived by Binsch [G. Binsch, in ***Dynamic NMR Spectroscopy,*** edited by **L.** M. Jackman and F. A. Cotton, **p.** 45. Academic Press, New York (1975)] and Heinzer and Oth [J. Heinzer and J. F. M. Oth, ***Helv. Chim.Acta*** 64, 258 (1981)], in which errors due to both the calculated rate constants and the measured temperature are taken into account. The absolute temperature errors were assumed to be σT = ±0.5 K and the maximum relative error in rate constants was taken to be $\sigma k/k$ = ±10%.

Table S46. Rate constants of compound **9** calculated from EXSY spectra in $CDCl_3$

T, K	*syn* to *anti*, s^{-1}	*anti* to *syn*, s^{-1}
263	0.079	0.100
268	0.145	0.193
273	0.257	0.314
278	0.455	0.573
283	0.769	0.978

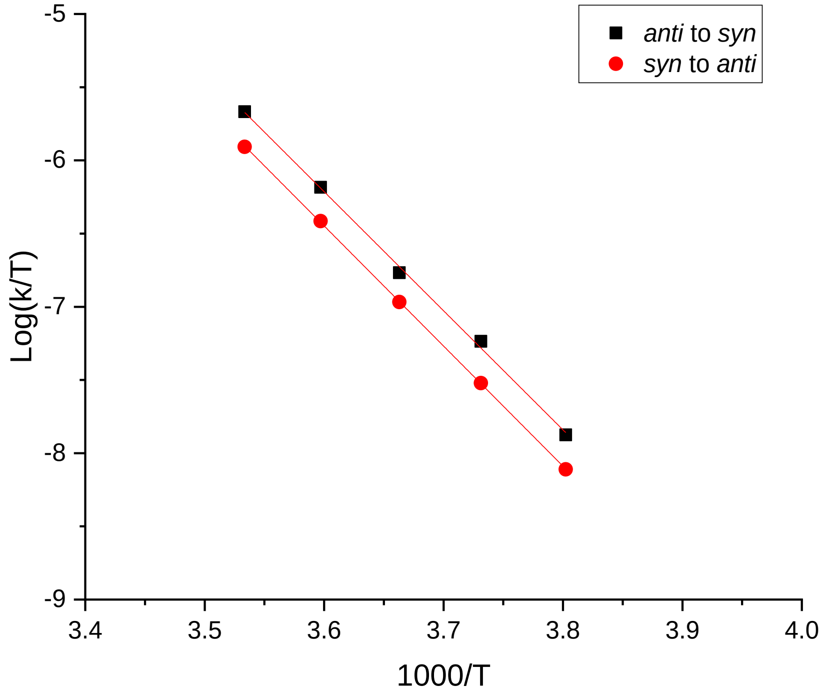

Figure S47. Eyring plot of rate constants of restricted rotation of compound **9** in $CDCl_3$

Table S47. Experimental Activation Parameters for the Exchange Processes of compound **9** calculated from ^1H EXSY spectra (signal of CH from *i*-Pr) in $CDCl_3$

Exchange	ΔH≠(298 K)	ΔS≠(298 K)	ΔG≠(298 K)	R^2
syn to *anti*	16.3 ± 1.2	-1.3 ± 4.1	16.7 ± 0.1	0.9999
anti to *syn*	16.2 ± 1.2	-1.4 ± 7.5	16.6 ± 0.1	0.9993

ΔG≠ and ΔH≠ in kcal mol^{-1} and ΔS≠ in cal mol^{-1} K^{-1}

HOmodecoupled Band-Selective NMR experiments (HOBS):

^{1}H HOBS EXSY spectra were recorded using *hobs_noesy* pulse program (refer to III. Pulse program codes for the HOBS-EXSY experiments) on a BBO probe in steps of 5 K between 263 and 293 K. The spectra were acquired using a spectral width of 2.4 kHz, 1024 x 128 complex time domain data points, mixing times in the range of 0.03 to 1.5 s and 8 scans in about 45 min. Linear prediction (32 coefficients and 256 points) in F1 was applied. The spectra were zerofilled to 4096 x 4096 data points and processed with a shifted square sine bell apodization in both dimensions. The populations were obtained by integration of 1D ^{1}H signals and the exchange rates were calculated by program EXSYCalc (MestreLab Research S.L.) from diagonal- and crosspeak integrals.

In the ^{1}H EXSY spectra of **9** the intensity of following peaks were calculated by volume integration:

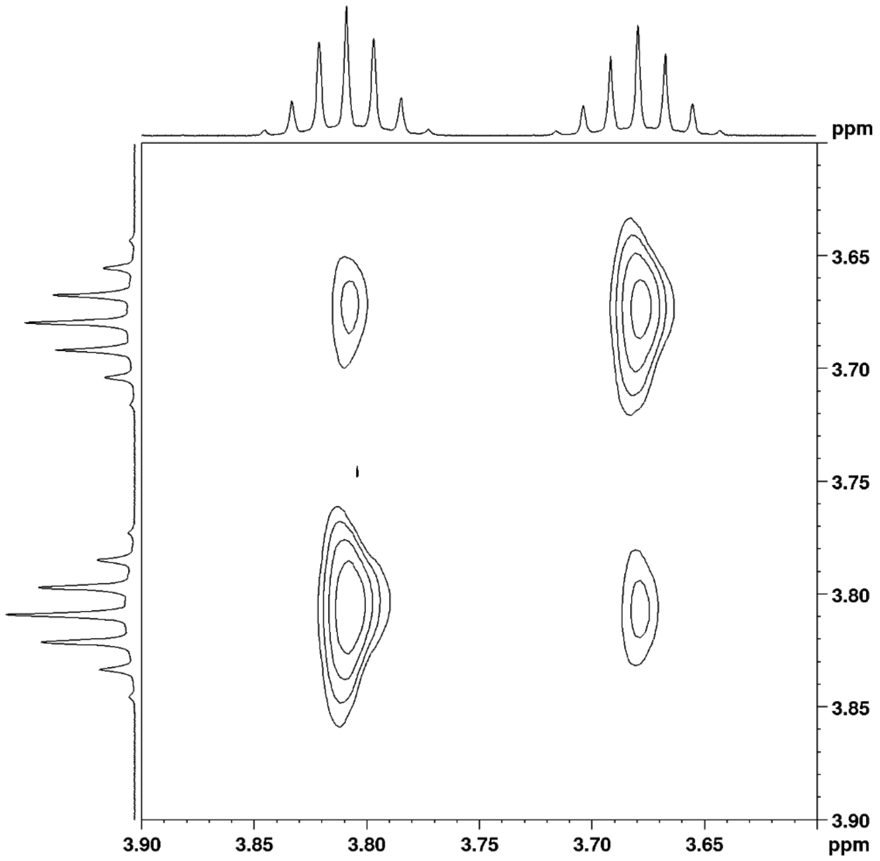

Figure S48. ^{1}H HOBS EXSY spectrum of compound **9** in $CDCl_3$ at 273 K using mixing time of 1.0 s.

Table S48. Rate constants of compound **9** calculated from ^{1}H HOBS EXSY spectra in $CDCl_3$

T, K	*syn* to *anti*, s^{-1}	*anti* to *syn*, s^{-1}
263	0.056	0.050
268	0.109	0.156
273	0.201	0.275
278	0.360	0.511
283	0.647	0.796
288	1.045	1.457
293	1.736	2.313

Table S49. Experimental Activation Parameters for the Exchange Processes of compound **9** calculated from ^{1}H HOBS EXSY spectra (signal of CH from *i*-Pr) in $CDCl_3$

Exchange	ΔH≠(298 K)	ΔS≠(298 K)	ΔG≠(298 K)	R^2
anti to *syn*	18.1 ± 1.2	5.3 ± 3.7	16.6 ± 0.1	0.9922
syn to *anti*	17.0 ± 0.8	0.6 ± 3.2	16.8 ± 0.1	0.9997

ΔG≠ and ΔH≠ in kcal mol^{-1} and ΔS≠ in cal mol^{-1} K^{-1}

Compound **10** in $CDCl_3$

^1H spectra were recorded on a Bruker II+ 600 instrument (BBO probe) at 600.13 MHz in steps of 5 K between 253 and 293 K (15 mg of **10** in 0.6 ml $CDCl_3$). Temperature calibration was done with B-VT 3000 unit (it was checked and calibrated with methanol and ethylene glycol reference samples). ^1H NMR spectra were acquired using a spectral width of 10 kHz, an acquisition time of 1.7 s and 32 scans, zerofilled to 64k datapoints (0.15 Hz per point) and processed without apodization. Peaks were fitted to a Lorentzian lineshape.

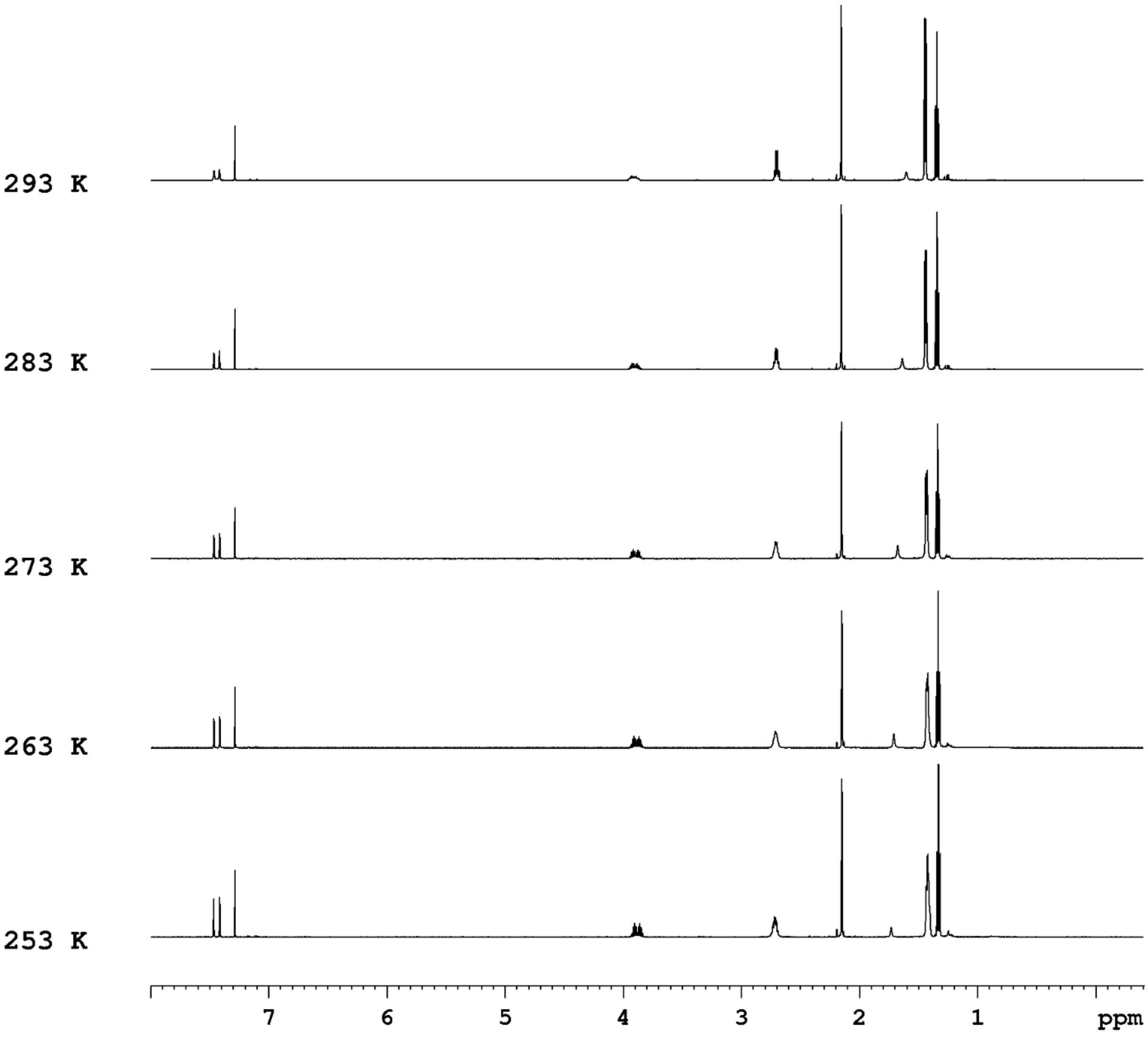

Figure S49. Stacked plots of ^1H NMR spectra of compound **10** in $CDCl_3$ at different temperatures.

^1H EXSY spectra were recorded on a BBO probe in steps of 5 K between 253 and 273 K. The spectra were acquired using a spectral width of 1.2 kHz, 2048 x 256 complex time domain data points, mixing times in the range of 0.05 to 1.0 s and 2 scans in about 45 min. Linear prediction (32 coefficients and 256 points) in F1 was applied. The spectra were zerofilled to 4096 x 4096 data points and processed with a shifted square sine bell apodization in both dimensions. The populations were obtained by integration of 1D ^1H

signals and the exchange rates were calculated by program EXSYCalc (MestreLab Research S.L.) from diagonal- and crosspeak integrals.

In the ^{1}H EXSY spectra of **10** the intensity of following peaks were calculated by volume integration:

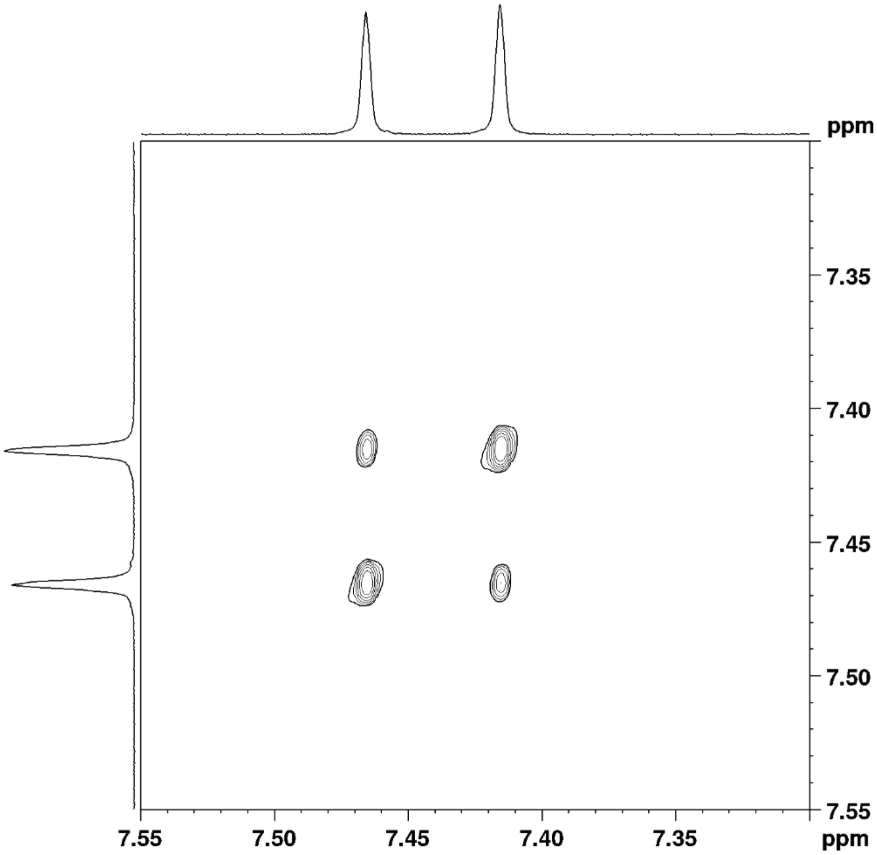

Figure S50. NOESY spectrum of compound **10** in $CDCl_3$ at 253 K using mixing time of 1.0 s.

Errors analysis

The total errors ***<u>Etot</u>***, quoted in activation parameters tables are calculated according to the expression $E_{tot} = \sqrt{E_S^2 + E_{kT}^2}$, where E_S is the statistical error based on scattering of the data in the Eyring plot while E_{kT} is computed using error propagation equations as derived by Binsch [G. Binsch, in ***Dynamic NMR Spectroscopy,*** edited by **L.** M. Jackman and F. A. Cotton, **p.** 45. Academic Press, New York (1975)] and Heinzer and Oth [J. Heinzer and J. F. M. Oth, ***Helv. Chim.Acta*** 64, 258 (1981)], in which errors due to both the calculated rate constants and the measured temperature are taken into account. The absolute temperature errors were assumed to be σT = ±0.5 K and the maximum relative error in rate constants was taken to be $\sigma k/k$ = ±10%.

Table S50. Rate constants of compound **10** calculated from EXSY spectra in $CDCl_3$

T, K	*syn* to *anti*, s^{-1}	*anti* to *syn*, s^{-1}
253	0.149	0.161
263	0.544	0.586
273	1.585	1.705
283	4.665	5.074
293	12.095	12.998

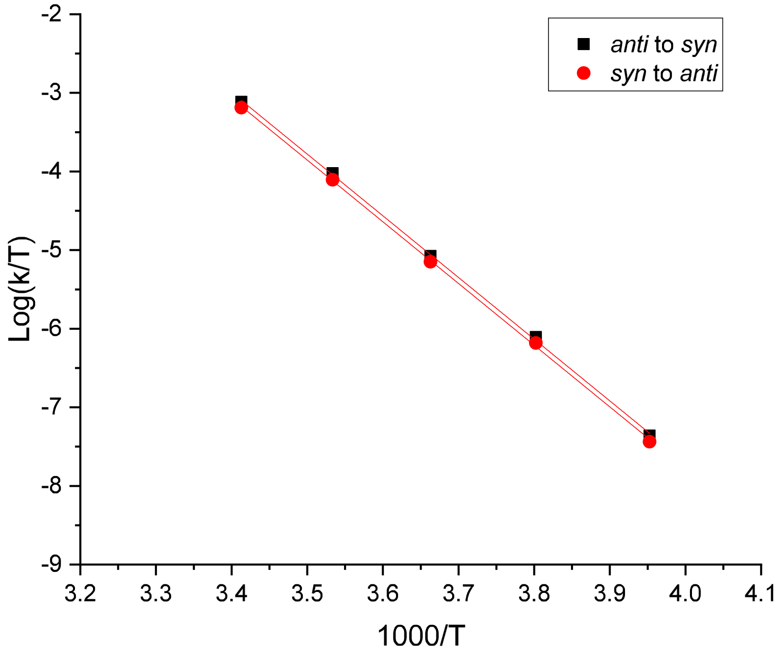

Figure S51. Eyring plot of rate constants of restricted rotation of compound **10** in $CDCl_3$

Table S51. Experimental Activation Parameters for the Exchange Processes of compound **10** calculated from ^1H EXSY spectra (signal of H-5) in $CDCl_3$

Exchange	ΔH≠(298 K)	ΔS≠(298 K)	ΔG≠(298 K)	R^2
syn to *anti*	15.6 ± 0.6	-0.3 ± 4.7	15.7 ± 0.1	0.9998
anti to *syn*	15.6 ± 0.6	-0.1 ± 5.2	15.6 ± 0.1	0.9998

ΔG≠ and ΔH≠ in kcal mol^{-1} and ΔS≠ in cal mol^{-1} K^{-1}

HOmodecoupled Band-Selective NMR experiments (HOBS):

^{1}H HOBS EXSY spectra were recorded using *hobs_noesy* pulse program (refer to III. Pulse program codes for the HOBS-EXSY experiments) on a BBO probe in steps of 5 K between 263 and 293 K. The spectra were acquired using a spectral width of 2.4 kHz, 1024 x 128 complex time domain data points, mixing times in the range of 0.03 to 1.5 s and 8 scans in about 45 min. Linear prediction (32 coefficients and 256 points) in F1 was applied. The spectra were zerofilled to 4096 x 4096 data points and processed with a shifted square sine bell apodization in both dimensions. The populations were obtained by integration of 1D ^{1}H signals and the exchange rates were calculated by program EXSYCalc (MestreLab Research S.L.) from diagonal- and crosspeak integrals.

In the ^{1}H EXSY spectra of **10** the intensity of following peaks were calculated by volume integration:

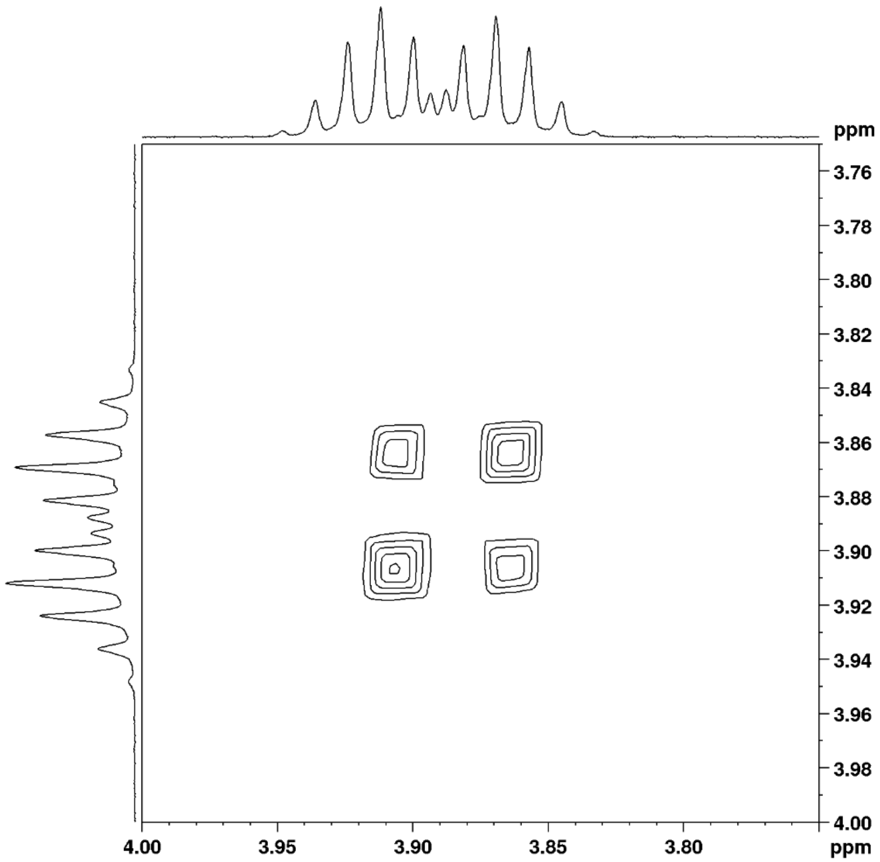

Figure S52. ^{1}H HOBS EXSY spectrum of compound **10** in $CDCl_3$ at 268 K using mixing time of 0.5 s.

Table S52. Rate constants of compound **10** calculated from HOBS EXSY spectra in $CDCl_3$

T, K	*syn* to *anti*, s^{-1}	*anti* to *syn*, s^{-1}
263	0.716	0.785
268	1.280	1.467
273	1.655	2.113
278	3.290	3.473
283	5.215	5.765
288	10.130	11.039
293	13.099	14.697

Table S53. Experimental Activation Parameters for the Exchange Processes of compound **10** calculated from ^1H HOBS EXSY spectra (signal of CH from *i*-Pr) in $CDCl_3$

Exchange	ΔH≠(298 K)	ΔS≠(298 K)	ΔG≠(298 K)	R^2
syn to *anti*	14.8 ± 1.0	-2.9 ± 2.8	15.6 ± 0.1	0.9946
anti to *syn*	14.6 ± 0.9	-3.4 ± 2.9	15.6 ± 0.1	0.9970

ΔG≠ and ΔH≠ in kcal mol^{-1} and ΔS≠ in cal mol^{-1} K^{-1}

Compound **11** in $CDCl_3$

^1H spectra were recorded on a Bruker II+ 600 instrument (BBO probe) at 600.13 MHz in steps of 5 K between 253 and 293 K (15 mg of **11** in 0.6 ml $CDCl_3$). Temperature calibration was done with B-VT 3000 unit (it was checked and calibrated with methanol and ethylene glycol reference samples). ^1H NMR spectra were acquired using a spectral width of 10 kHz, an acquisition time of 1.7 s and 32 scans, zerofilled to 64k datapoints (0.15 Hz per point) and processed without apodization. Peaks were fitted to a Lorentzian lineshape.

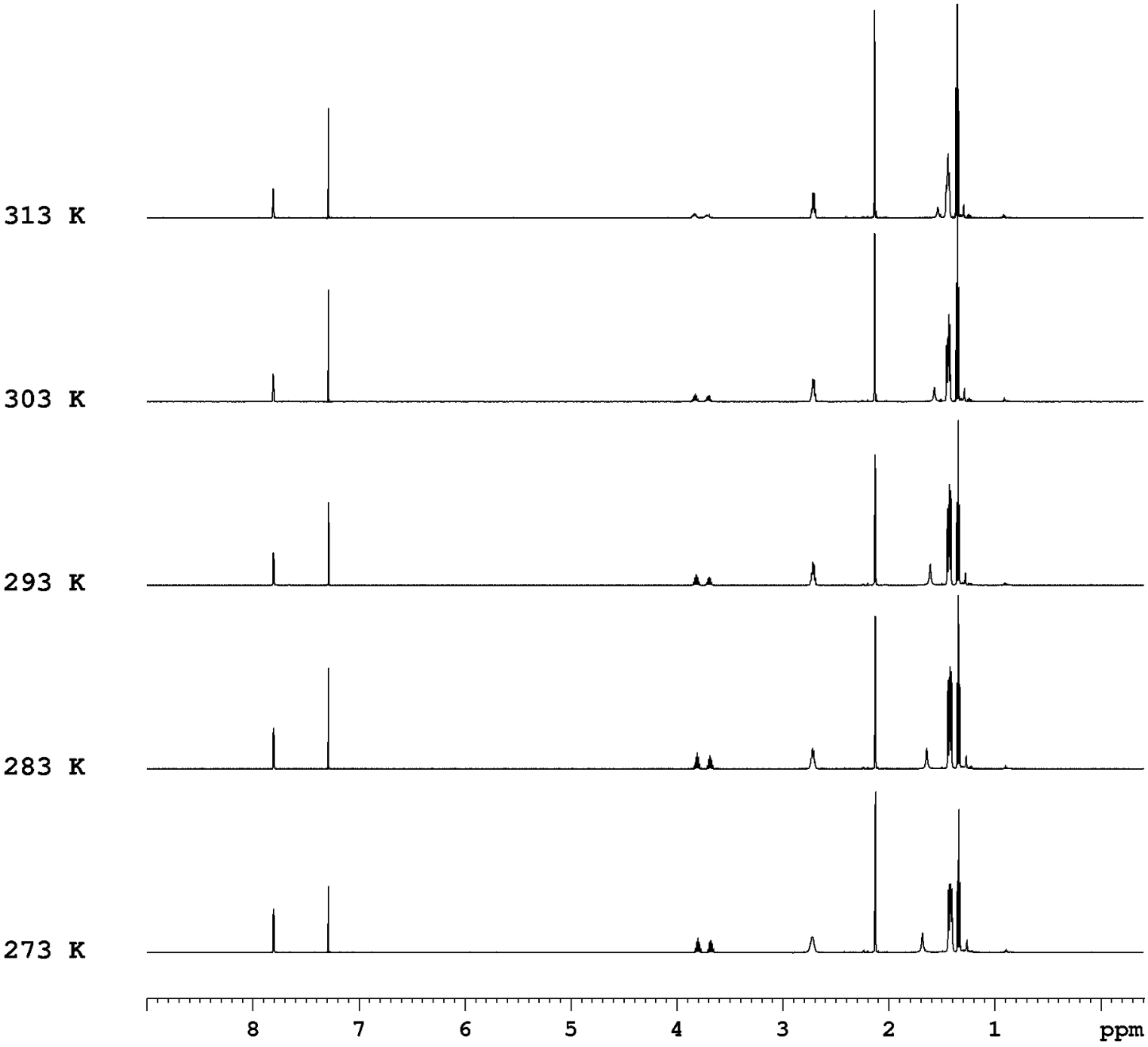

Figure S53. Stacked plots of ^1H NMR spectra of compound **11** in $CDCl_3$ at different temperatures.

^1H EXSY spectra were recorded on a BBO probe in steps of 5 K between 253 and 273 K. The spectra were acquired using a spectral width of 1.2 kHz, 2048 x 256 complex time domain data points, mixing times in the range of 0.05 to 1.0 s and 2 scans in about 45 min. Linear prediction (32 coefficients and 256 points) in

F1 was applied. The spectra were zerofilled to 4096 x 4096 data points and processed with a shifted square sine bell apodization in both dimensions. The populations were obtained by integration of 1D ^{1}H signals and the exchange rates were calculated by program EXSYCalc (MestreLab Research S.L.) from diagonal- and crosspeak integrals.

In the ^{1}H EXSY spectra of **11** the intensity of following peaks were calculated by volume integration:

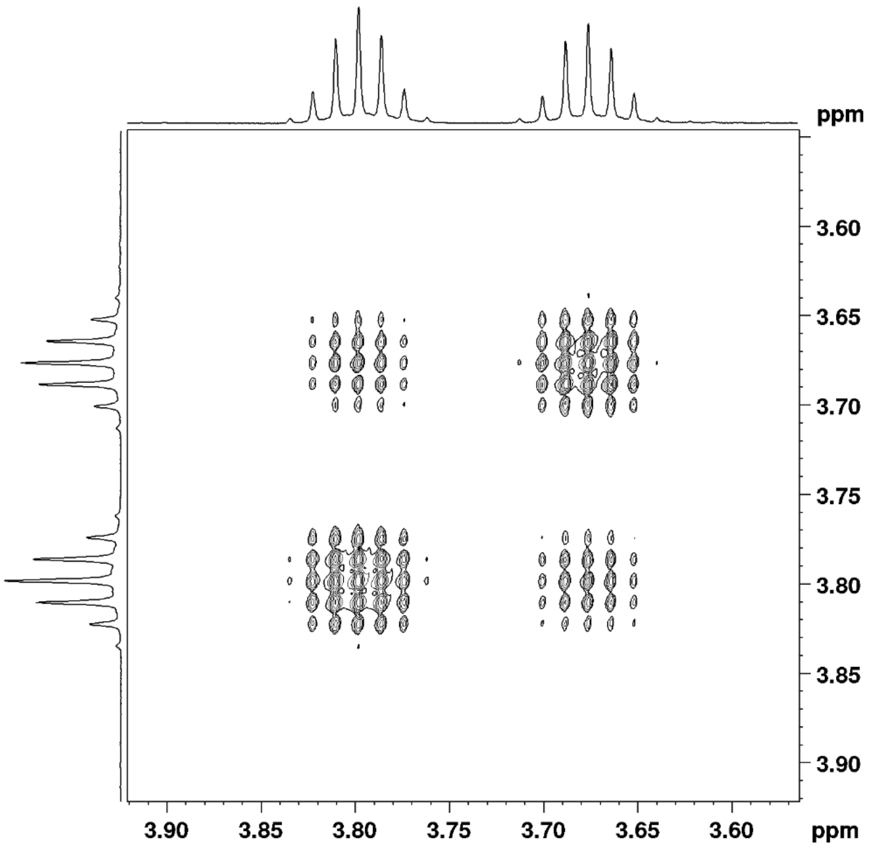

Figure S54. NOESY spectrum of compound **11** in $CDCl_3$ at 273 K using mixing time of 1.25 s.

Errors analysis

The total errors ***E**$_{tot}$*, quoted in activation parameters tables are calculated according to the expression $E_{tot} = \sqrt{E_S^2 + E_{kT}^2}$, where ***E**$_S$* is the statistical error based on scattering of the data in the Eyring plot while ***E**$_{kT}$* is computed using error propagation equations as derived by Binsch [G. Binsch, in ***Dynamic NMR Spectroscopy,*** edited by **L.** M. Jackman and F. A. Cotton, **p.** 45. Academic Press, New York (1975)] and Heinzer and Oth [J. Heinzer and J. F. M. Oth, ***Helv. Chim.Acta*** 64, 258 (1981)], in which errors due to both the calculated rate constants and the measured temperature are taken into account. The absolute temperature errors were assumed to be σT = ±0.5 K and the maximum relative error in rate constants was taken to be $\sigma k/k$ = ±10%.

Table S54. Rate constants of compound **11** calculated from EXSY spectra in $CDCl_3$

T, K	*syn* to *anti*,s^{-1}	*anti* to *syn*,s^{-1}
273	0.255	0.293
283	0.740	0.847
293	1.946	2.230
303	4.996	5.726
313	11.390	13.200

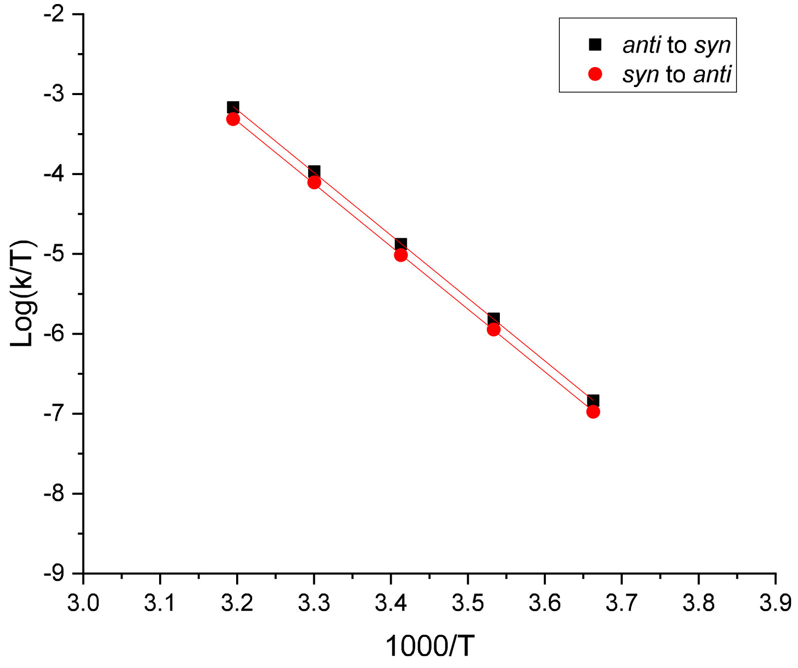

Figure S55. Eyring plot of rate constants of restricted rotation of compound **11** in $CDCl_3$

Table S55. Experimental Activation Parameters for the Exchange Processes of compound **11** calculated from ^1H EXSY spectra (signal of CH from *i*-Pr) in $CDCl_3$

Exchange	ΔH≠(298 K)	ΔS≠(298 K)	ΔG≠(298 K)	R^2
syn to *anti*	15.6 ± 0.7	-4.0 ± 2.2	16.8 ± 0.1	0.9999
anti to *syn*	15.6 ± 0.7	-3.6 ± 2.2	16.7 ± 0.1	0.9999

ΔG≠ and ΔH≠ in kcal mol^{-1} and ΔS≠ in cal mol^{-1} K^{-1}

HOmodecoupled Band-Selective NMR experiments (HOBS):

^{1}H HOBS EXSY spectra were recorded using *hobs_noesy* pulse program (refer to III. Pulse program codes for the HOBS-EXSY experiments) on a BBO probe in steps of 5 K between 263 and 293 K. The spectra were acquired using a spectral width of 2.4 kHz, 1024 x 128 complex time domain data points, mixing times in the range of 0.03 to 1.5 s and 8 scans in about 45 min. Linear prediction (32 coefficients and 256 points) in F1 was applied. The spectra were zerofilled to 4096 x 4096 data points and processed with a shifted square sine bell apodization in both dimensions. The populations were obtained by integration of 1D ^{1}H signals and the exchange rates were calculated by program EXSYCalc (MestreLab Research S.L.) from diagonal- and crosspeak integrals.

In the ^{1}H EXSY spectra of **11** the intensity of following peaks were calculated by volume integration:

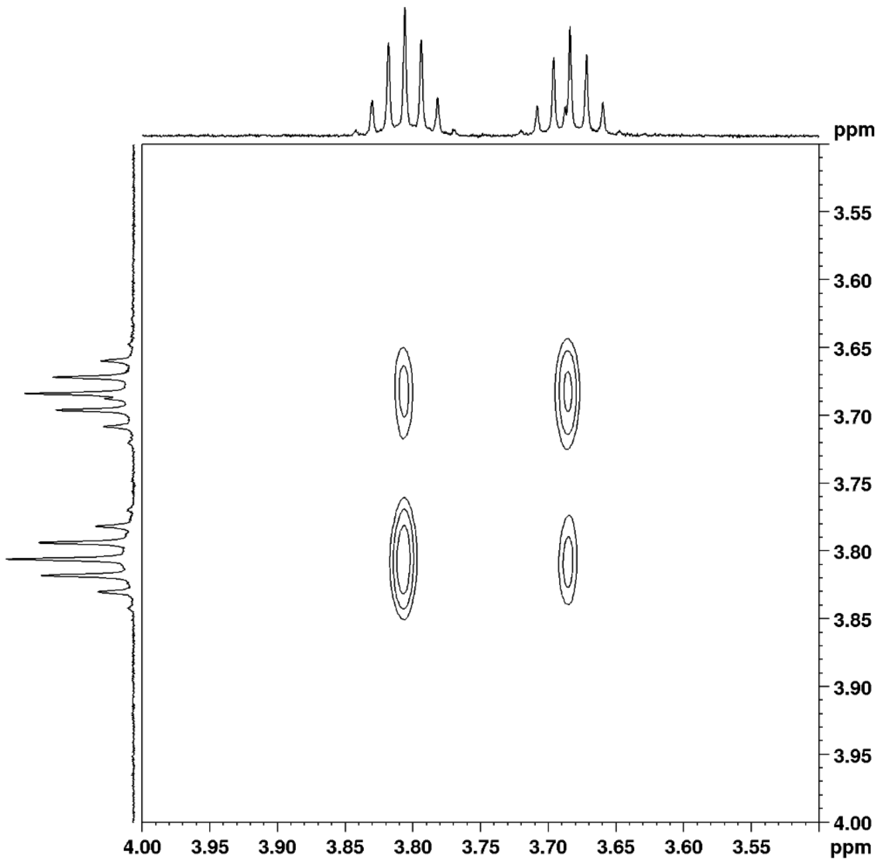

Figure S56. ^{1}H HOBS EXSY spectrum of compound **11** in $CDCl_3$ at 283 K using mixing time of 0.75 s.

Table S56. Rate constants of compound **11** calculated from HOBS EXSY spectra in $CDCl_3$

T, K	*syn* to *anti*, s^{-1}	*anti* to *syn*, s^{-1}
273	0.231	0.247
283	0.744	0.871
293	1.886	2.184
303	4.923	5.405
313	9.711	12.502

Table S57. Experimental Activation Parameters for the Exchange Processes of compound **11** calculated from ^1H HOBS EXSY spectra (signal of CH from *i*-Pr) in $CDCl_3$

Exchange	ΔH≠(298 K)	ΔS≠(298 K)	ΔG≠(298 K)	R^2
syn to *anti*	15.4 ± 0.8	-4.8 ± 2.4	16.8 ± 0.1	0.9986
anti to *syn*	15.9 ± 0.8	-2.8 ± 3.1	16.7 ± 0.1	0.9988

ΔG≠ and ΔH≠ in kcal mol^{-1} and ΔS≠ in cal mol^{-1} K^{-1}

Table S58. Experimental EXSY activation parameters for the exchange processes *anti* to *syn* in $CDCl_3$

Compound	X	R[1]	ΔH≠(298 K), kcal/mol	ΔS≠(298 K), e.u.	ΔG≠(298 K), kcal/mol	T range, K
**1**	Cl	H	15.5 ± 0.8	5.1 ± 3.4	14.0 ± 0.1	233 – 268
**2**	Br	H	15.8 ± 1.2	0.7 ± 2.5	15.6 ± 0.1	253 – 293
**3**	I	H	14.4 ± 0.7	-8.4 ± 2.4	16.9 ± 0.1	253 – 273
**4**	Br	CH_3	16.1 ± 1.0	1.9 ± 2.8	15.6 ± 0.1	253 – 293
**5**	I	CH_3	16.0 ± 1.1	-2.2 ± 6.5	16.7 ± 0.1	253 – 278
**6**	Br	Allyl	15.4 ± 0.6	-0.9 ± 1.8	15.6 ± 0.1	243 – 293
**7**	I	Allyl	16.7 ± 1.2	0.9 ± 3.0	16.4 ± 0.1	253 – 293
**8**	Br	C(=O)*i*-Pr	15.6 ± 1.1	0.1 ± 2.7	15.6 ± 0.1	253 – 293
**9**	I	C(=O)*i*-Pr	16.2 ± 1.2	-1.4 ± 7.5	16.6 ± 0.1	273 – 313
**10**	Br	C(=O)Et	15.6 ± 0.6	-0.1 ± 5.2	15.6 ± 0.1	243 – 293
**11**	I	C(=O)Et	15.6 ± 0.7	-3.6 ± 2.2	16.7 ± 0.1	253 – 293

Table S59. Experimental HOBS EXSY activation parameters for the exchange processes *anti* to *syn* in $CDCl_3$

Compound	X	R[1]	ΔH≠(298 K), kcal/mol	ΔS≠(298 K), e.u.	ΔG≠(298 K), kcal/mol	T range, K
**1**	Cl	H	15.4 ± 1.0	4.2 ± 3.6	14.1 ± 0.1	233 – 268
**2**	Br	H	16.2 ± 1.1	1.9 ± 3.2	15.6 ± 0.1	253 – 283
**3**	I	H	12.3 ± 1.2	-15.2 ± 4.4	16.9 ± 0.1	263 – 293
**4**	Br	CH_3	18.7 ± 1.2	10.9 ± 4.4	15.5 ± 0.1	253 – 273
**5**	I	CH_3	16.7 ± 0.9	0.0 ± 2.7	16.7 ± 0.1	263 – 293
**6**	Br	Allyl	15.2 ± 0.8	-1.9 ± 2.4	15.8 ± 0.1	253 – 283
**7**	I	Allyl	16.4 ± 0.9	-0.1 ± 1.4	16.4 ± 0.1	263 – 293
**8**	Br	C(=O)*i*-Pr	17.4 ± 1.1	7.2 ± 4.5	15.3 ± 0.1	263 – 288
**9**	I	C(=O)*i*-Pr	18.1 ± 1.2	5.3 ± 3.7	16.6 ± 0.1	263 – 293
**10**	Br	C(=O)Et	14.6 ± 0.9	-3.4 ± 2.9	15.6 ± 0.1	263 – 293
**11**	I	C(=O)Et	15.9 ± 0.8	-2.8 ± 3.1	16.7 ± 0.1	273 – 313

Table S60. Experimental EXSY activation parameters for the exchange processes *syn* to *anti* in $CDCl_3$.

Compound	X	R[1]	ΔH≠(298 K), kcal/mol	ΔS≠(298 K), e.u.	ΔG≠(298 K), kcal/mol	T range, K
**1**	Cl	H	14.6 ± 0.8	1.6 ± 3.9	14.1 ± 0.1	233 – 268
**2**	Br	H	15.0 ± 1.2	-2.4 ± 9.7	15.7 ± 0.1	253 – 293
**3**	I	H	14.5 ± 0.6	-8.1 ± 2.4	16.9 ± 0.1	253 – 273
**4**	Br	CH_3	16.4 ± 1.0	2.5 ± 3.7	15.6 ± 0.1	253 – 293
**5**	I	CH_3	17.0 ± 1.2	1.9 ± 3.9	16.4 ± 0.1	253 – 278
**6**	Br	Allyl	15.2 ± 0.6	-1.9 ± 2.2	15.8 ± 0.1	243 – 293
**7**	I	Allyl	16.5 ± 1.3	-0.5 ± 2.4	16.7 ± 0.1	253 – 293
**8**	Br	C(=O)*i*-Pr	15.8 ± 1.1	0.7 ± 4.6	15.6 ± 0.1	253 – 293
**9**	I	C(=O)*i*-Pr	16.3 ± 1.2	-1.3 ± 4.1	16.7 ± 0.1	273 – 313
**10**	Br	C(=O)Et	15.6 ± 0.6	-0.3 ± 4.7	15.7 ± 0.1	243 – 293
**11**	I	C(=O)Et	15.6 ± 0.7	-4.0 ± 2.2	16.8 ± 0.1	253 – 293

Table S61. Experimental HOBS EXSY activation parameters for the exchange processes *syn* to *anti* in $CDCl_3$.

Compound	X	R[1]	ΔH≠(298 K), kcal/mol	ΔS≠(298 K), e.u.	ΔG≠(298 K), kcal/mol	T range, K
**1**	Cl	H	15.5 ± 0.7	5.0 ± 2.7	14.0 ± 0.1	233 – 268
**2**	Br	H	17.5 ± 0.8	7.4 ± 3.0	15.3 ± 0.1	253 – 283
**3**	I	H	13.0 ± 1.0	-13.0 ± 3.5	16.9 ± 0.1	263 – 293
**4**	Br	CH_3	16.8 ± 1.6	3.9 ± 12.6	15.6 ± 0.1	253 – 273
**5**	I	CH_3	16.6 ± 0.9	0.0 ± 1.2	16.6 ± 0.1	263 – 293
**6**	Br	Allyl	16.3 ± 0.8	1.9 ± 2.5	15.7 ± 0.1	253 – 283
**7**	I	Allyl	16.3 ± 0.8	-1.1 ± 3.9	16.7 ± 0.1	263 – 293
**8**	Br	C(=O)*i*-Pr	17.5 ± 1.4	7.6 ± 6.4	15.2 ± 0.1	263 – 288
**9**	I	C(=O)*i*-Pr	17.0 ± 0.8	0.6 ± 3.2	16.8 ± 0.1	263 – 293
**10**	Br	C(=O)Et	14.8 ± 1.0	-2.9 ± 2.8	15.6 ± 0.1	263 – 293
**11**	I	C(=O)Et	15.4 ± 0.8	-4.8 ± 2.4	16.8 ± 0.1	273 – 313

Pulse program codes for the HOBS-EXSY experiments

Pulse Program Code for Bruker: 1D selective HOBS-EXSY

```
;Pulse Program Code for Bruker:

;pp:hobs_selnogp
;1D Experiment
;HOmodecoupled Band-Selective NMR experiment (HOBS)
;    based on the instant Zangger-Sterk experiment
;Method for fast acquisition and full sensitive homodecoupled band-selective spectra

;N.H. Meyer and K. Zangger.
;   Angew. Chem. Int. Ed. (2013) 52, 7143-7146

;L. Casta<F1>ar, P. Nolis, A. Virgili & T. Parella,
;   Chem. Eur. J (2013)19,17283-17286
;   DOI:chem.201303235
;
;incorporated into
;
;1D NOESY using selective refocussing with a shaped pulse
;dipolar coupling may be due to noe or chemical exchange.
;
;H. Kessler, H. Oschkinat, C. Griesinger & W. Bermel,
;   J. Magn. Reson. 70, 106 (1986)
;J. Stonehouse, P. Adell, J. Keeler & A.J. Shaka,
;   J. Am. Chem. Soc 116, 6037 (1994)
;K. Stott, J. Stonehouse, J. Keeler, T.L. Hwang & A.J. Shaka,
```

```
;   J. Am. Chem. Soc 117, 4199-4200 (1995)


;Avance III version (01/07/2013)
;Topspin2.1 & Topspin3.1


#include <Avance.incl>
#include <Grad.incl>
#include <Delay.incl>
#include <De.incl>

dwellmode explicit


"d12=6.5u"
"d2=aq/l0"
"d3=d2/2"
"l1=l0-1"
"p2=2*p1"

"d20=d8*0.5-p16-d16"


#   ifdef CALC_SPOFFS
"spoff2=bf1*(cnst21/1000000)-o1"
#   else
#   endif /*CALC_SPOFFS*/
```

```
"acqt0=-p1*2/3.1416-4u"

1 ze
2 30m
  20u pl1:f1
  d1
  50u UNBLKGRAD
  (p1 ph1):f1
  3u
  p16:gp4
  d16 pl0:f1
  p13:sp3:f1 ph2:r
  3u
  p16:gp4
  d16 pl1:f1
  (p1 ph3):f1
  d20
  p16:gp5
  d16
  3u
  (p2 ph4):f1
  3u
  p16:gp5*-1
  d16
```

```
d20
(p1 ph5):f1

p16:gp1
d16 pl0:f1
(p12:sp2 ph8)
p16:gp1
d16


ACQ_START(ph30,ph31)

 0.05u setrtp1|0
 0.1u setrtp1|5
 d3:r
 0.1u setrtp1^5
 0.05u setrtp1^0

 p16:gp2
 d16 pl1:f1
 p2 ph6
 p16:gp2
 d16

 p16:gp3
 d16 pl0:f1
 (p12:sp2 ph7)
 p16:gp3
```

```
  d16

3  0.05u setrtp1|0
  0.1u setrtp1|5
  d2:r
  0.1u setrtp1^5
  0.05u setrtp1^0

  p16:gp2
  d16 pl1:f1
  p2 ph6
  p16:gp2
  d16

  p16:gp3
  d16 pl0:f1
  (p12:sp2 ph7)
  p16:gp3
  d16

lo to 3 times l1

  0.05u setrtp1|0
  0.1u setrtp1|5
  d3
  30m
  0.1u setrtp1^5
  0.05u setrtp1^0
```

rcyc=2

wr #0

exit

ph0=0

ph1=0 2

ph2=0 0 1 1 2 2 3 3

ph3=0

ph4=0

ph5=0

ph8=0 0 2 2

ph6=0 2

ph7=0 2

ph30=0

ph31=0 2 2 0 1 3 3 1

;pl1 : f1 channel - power level for pulse (default)

;p1 : f1 channel - high power pulse

;p12 : f1 channel - 180 degree band-selective pulse [ms]

;p16: homospoil/gradient pulse [500 us]

;sp2: f1 channel - shaped pulse power level for band-selective excitation

;spnam2: shaped pulse for selective excitation [REBURP]

;d1 : relaxation delay [1s] (only one scan is recorded)

;d16: delay for homospoil/gradient recovery [20us]

;NS: 1 * n

;DS: 0

;l1: number of concatenated loops

;for z-only gradients:

;gpz1: 17%

;gpz2: 41%

;gpz3: 63%

;use gradient files:

;gpnam1: SMSQ10.100 (Topspin3.1)

;   SINE.100 (Topspin2.1)

;gpnam2: SMSQ10.100 (Topspin3.1)

;   SINE.100 (Topspin2.1)

;gpnam3: SMSQ10.100 (Topspin3.1)

;   SINE.100 (Topspin2.1)

;pl0 : 0W

;pl1 : f1 channel - power level for pulse (default)

;sp3: f1 channel - shaped pulse

;p1 : f1 channel - 90 degree high power pulse

;p2 : f1 channel - 180 degree high power pulse

;p13: f1 channel - 180 degree shaped pulse

;p16: homospoil/gradient pulse   [1 msec]

;d1 : relaxation delay; 1-5 * T1

```
;d8 : mixing time
;d16: delay for homospoil/gradient recovery
;d20: d8*0.5 - p16 - d16
;cnst21: chemical shift for selective pulse (offset, in ppm)
;ns: 2 * n, total number of scans: NS * TD0
;ds: 4
```

Pulse Program Code for Bruker: 2D HOBS-EXSY

```
;Pulse Program Code for Bruker:


;hobs_noesy based on hobs and noesyph
;HOmodecoupled Band-Selective NMR experiment (HOBS)
;    based on the instant Zangger-Sterk experiment
;Method for fast acquisition and full sensitive homodecoupled band-selective spectra


;N.H. Meyer and K. Zangger.
;   Angew. Chem. Int. Ed. (2013) 52, 7143-7146
;L. Casta<F1>ar, P. Nolis, A. Virgili & T. Parella,
;   Chem. Eur. J (2013) DOI:chem.201303235


;avance-version (07/0/2013)
;Topspin2.1 & Topspin3.1


#include <Avance.incl>
#include <Delay.incl>
#include <De.incl>
#include <Grad.incl>
```

```
dwellmode explicit

"d12=20u"
"d20=2m"
"d21=3m"
"d2=aq/l0"
"d3=d2/2"
"l2=l0-1"
"p2=2*p1"

"in0=inf1"
"d0=in0/2-p1*4/3.1416"
"l4=p15/(p25*2)"
"acqt0=-p1*2/3.1416"

1 ze
2 2m
  d1
  50u UNBLKGRAD

3 d12 pl1:f1
  p1 ph1
  d0
  p1 ph2
  d8
  p1 ph3

  p16:gp1
```

```
d16
d12 pl0:f1
(p12:sp2 ph3)
p16:gp1
d16
d12 pl1:f1

ACQ_START(ph30,ph31)

 0.05u DWL_CLK_ON
 0.1u REC_UNBLK
 d3:r
 0.05u DWL_CLK_OFF
 0.1u REC_BLK

 p16:gp2
 d16 pl1:f1
 p2 ph6
 p16:gp2
 d16

 p16:gp3
 d16 pl0:f1
 (p12:sp2 ph7)
 p16:gp3
 d16

5  0.05u DWL_CLK_ON
```

```
  0.1u REC_UNBLK
  d2:r
  0.05u DWL_CLK_OFF
  0.1u REC_BLK

  p16:gp2
  d16 pl1:f1
  p2 ph6
  p16:gp2
  d16

  p16:gp3
  d16 pl0:f1
  (p12:sp2 ph7)
  p16:gp3
  d16

lo to 5 times l2

  0.05u DWL_CLK_ON
  0.1u REC_UNBLK
  d3
  30m
  0.05u DWL_CLK_OFF
  0.1u REC_BLK

rcyc=2
2m mc #0 to 2 F1PH(ip1, id0)
```

exit

ph0=0

ph1=0 2

ph2=0 0 0 0 0 0 0 0 2 2 2 2 2 2 2 2

ph3=0 0 2 2 1 1 3 3

ph23=3

ph25=1

ph6=0 2

ph7=0 2

ph30=0

ph31=0 2 2 0 1 3 3 1 2 0 0 2 3 1 1 3

;pl1 : f1 channel - power level for pulse (default)

;p1 : f1 channel - 90 degree high power pulse

;p6 : f1 channel - 90 degree low power pulse

;p12 : f1 channel - 180 degree band-selective pulse [ms]

;p16: homospoil/gradient pulse [1 ms]

;d0 : incremented delay (2D)

;d1 : relaxation delay; 1-5 * T1

;d12: delay for power switching [20 usec]

;d16: delay for homospoil/gradient recovery

;d20: first z-filter delay [2 msec]

;d21: second z-filter delay [3 msec]

;l2: number of concatenated loops

;inf1: 1/SW = 2 * DW

;in0: 1/(1 * SW) = 2 * DW

;nd0: 1

;NS: 8 * n

;DS: 16

;td1: number of experiments

;FnMODE: States-TPPI, TPPI, States or QSEQ

;sp2: f1 channel - shaped pulse power level for band-selective excitation

;spnam2: shaped pulse for selective excitation [REBURP]

;d8 : mixing time

;Processing

;PHC0(F1): 90

;PHC1(F1): -180

;FCOR(F1): 1

DFT calculations

All calculations were performed by means of quantum chemical calculations at the density functional theory (DFT) level using Gaussian09 program package [4].

The geometries of all compounds have been fully optimized and the corresponding transition states were localized using B3LYP [5] functional with 6-311++G(d,p) basis set [6]. Solvent effect was included implicitly to the optimizations via the SMD [7] model with the built in parameters for solvent ($CDCl_3$). The nature of all critical points was confirmed by means of the vibrational analysis.

The ΔH, ΔS and ΔG values were calculated at T = 298.15 K at the same level of theory including zero-point energy in the particular solvent environment (represented by relative permittivity) and vibrational, rotational and translational thermal energy corrections.

XYZ coordinates

1 (*anti*-GS)

25

XYZ -Stationary point- [Standard orientation]

C	-1.20495000	1.73227400	-0.00000400
C	0.15473700	1.43296300	-0.00000400
C	0.65493700	0.12216100	-0.00000100
C	-0.34022100	-0.87300600	0.00000200
C	-1.71349100	-0.60006500	0.00000200
C	-2.16333800	0.72807100	-0.00000100
C	-3.63717300	1.02965400	-0.00000100
C	2.15767500	-0.15743200	-0.00000100
C	2.64996100	-0.85545200	1.28220300
C	2.64996300	-0.85544900	-1.28220500
O	-2.66396500	-1.57121500	0.00000600
H	-1.52194900	2.76762900	-0.00000600
H	-4.12849600	0.60132400	0.87891500
H	-4.12849600	0.60132000	-0.87891500
H	-3.80891900	2.10726500	-0.00000300
H	2.63058100	0.82311200	0.00000100
H	3.74419100	-0.87013100	1.28620000
H	2.32125600	-0.31350600	2.17353200
H	2.30478300	-1.88679000	1.36483100
H	3.74419300	-0.87012600	-1.28620100
H	2.32125700	-0.31350100	-2.17353300
H	2.30478600	-1.88678700	-1.36483700
H	-2.24942000	-2.44704500	0.00000800
Cl	0.07828100	-2.59766400	0.00000800

Cl	1.24697500	2.83499600	-0.00000700

1 (*syn*-GS)

25

XYZ -Stationary point- [Standard orientation]

C	-0.66910500	1.88541600	-0.00000100
C	0.52633800	1.17166200	-0.00000000
C	0.58053000	-0.22928200	-0.00000100
C	-0.68264200	-0.85334100	0.00000000
C	-1.89854000	-0.15970500	-0.00000500
C	-1.89954800	1.24171300	-0.00000600
C	-3.19862700	2.00047400	-0.00000900
C	1.86565700	-1.05723100	-0.00000500
C	2.70062800	-0.87835200	-1.28212600
C	2.70062300	-0.87837000	1.28212000
O	-3.10870200	-0.77845400	-0.00001100
H	-0.63856700	2.96803500	0.00000000
H	-3.80148500	1.75259100	0.87890100
H	-3.80147300	1.75260400	-0.87893100
H	-3.01538700	3.07624300	-0.00000000
H	1.54799500	-2.09844600	-0.00001400
H	3.51848200	-1.60550300	-1.28443700
H	2.09255800	-1.05839600	-2.17331700
H	3.13939600	0.11651000	-1.36492000
H	3.51848300	-1.60551600	1.28442000
H	2.09255200	-1.05843400	2.17330600
H	3.13938400	0.11649400	1.36493400
H	-2.99243900	-1.74006100	-0.00000500

Cl	-0.82652400	-2.62495600	0.00001300
Cl	1.99818500	2.16340400	0.00000700

2 (*anti*-GS)

25

XYZ -Stationary point- [Standard orientation]

C	1.20013300	1.84256500	0.00000000
C	1.33043400	0.45586300	0.00000000
C	0.23514300	-0.42329200	0.00000100
C	-1.01765000	0.22276400	0.00000100
C	-1.17620500	1.61505500	0.00000100
C	-0.04764400	2.44884600	0.00000100
C	-0.20989300	3.94453700	0.00000100
C	0.43799400	-1.93989500	0.00000100
C	-0.05979400	-2.63101900	1.28337700
C	-0.05979800	-2.63101700	-1.28337500
O	-2.38475000	2.23454700	0.00000100
Br	3.16118600	-0.18618700	-0.00000100
Br	-2.68753900	-0.76674500	-0.00000100
H	2.08538200	2.46579500	-0.00000000
H	-0.76766600	4.28143800	0.87899100
H	-0.76766400	4.28143800	-0.87899100
H	0.76436700	4.43606500	0.00000200
H	1.51788300	-2.07622600	-0.00000000
H	0.28297500	-3.67020000	1.28650900
H	0.34676900	-2.14373500	2.17414600
H	-1.14668700	-2.64242400	1.37017800
H	0.28296600	-3.67020000	-1.28650700

H	0.34676700	-2.14373500	-2.17414300
H	-1.14669100	-2.64241600	-1.37017500
H	-3.09436700	1.57398500	0.00000100

2 (*syn*-GS)

25

XYZ -Stationary point- [Standard orientation]

C	0.80138400	1.96244800	0.00000300
C	1.13859500	0.61049300	0.00000200
C	0.18157600	-0.41585200	0.00000100
C	-1.15234400	0.04505800	-0.00000000
C	-1.51678200	1.39704100	0.00000100
C	-0.52075100	2.38400200	0.00000200
C	-0.89476900	3.84115400	0.00000300
C	0.49297000	-1.91393800	-0.00000000
C	1.19826700	-2.39121000	-1.28320000
C	1.19824300	-2.39121900	1.28320900
O	-2.80386000	1.83156800	-0.00000000
Br	3.04710100	0.27249900	-0.00000100
Br	-2.64860600	-1.19485600	-0.00000300
H	1.58655900	2.70781400	0.00000300
H	-1.49475800	4.09533100	0.87901900
H	-1.49475600	4.09533200	-0.87901400
H	-0.00042900	4.46639400	0.00000400
H	-0.47379500	-2.41374000	-0.00001000
H	1.24398300	-3.48460100	-1.28250900
H	0.64352100	-2.08127700	-2.17351900
H	2.21797800	-2.01566400	-1.37068900

H	1.24395700	-3.48461100	1.28251300
H	0.64348200	-2.08129000	2.17352100
H	2.21795300	-2.01567600	1.37071900
H	-3.40844800	1.07406500	-0.00000200

3 (*anti*-GS)

25

XYZ -Stationary point- [Standard orientation]

C	1.25913900	1.90165900	-0.00000400
C	1.31388300	0.50817300	-0.00000300
C	0.16437400	-0.30319200	-0.00000500
C	-1.05859300	0.40719100	-0.00000500
C	-1.12748800	1.81024600	-0.00000800
C	0.04889800	2.57729600	-0.00000800
C	-0.02399800	4.08041700	-0.00001000
C	0.29513700	-1.82961100	-0.00000900
C	-0.21993400	-2.50265700	1.28547000
C	-0.21994600	-2.50265100	-1.28548600
O	-2.28591900	2.51907200	-0.00001200
I	3.32204600	-0.28619800	0.00000400
I	-2.99446300	-0.55747100	0.00000800
H	2.17594500	2.47729700	-0.00000200
H	-0.56007000	4.45138300	0.87880100
H	-0.56006500	4.45138100	-0.87882400
H	0.97841000	4.51195500	-0.00000700
H	1.36844000	-2.01164200	-0.00001500
H	0.07636100	-3.55635200	1.28127400
H	0.22075100	-2.03665100	2.17152400

H	-1.30480600	-2.46580700	1.38754300
H	0.07635500	-3.55634500	-1.28129900
H	0.22072500	-2.03663800	-2.17154200
H	-1.30482000	-2.46580700	-1.38754600
H	-3.05058200	1.91785100	-0.00001200

3 (*syn*-GS)

25

XYZ -Stationary point- [Standard orientation]

C	1.07853000	1.93288600	0.00000100
C	1.19839100	0.54184500	0.00000200
C	0.07964700	-0.31010600	0.00000100
C	-1.16967200	0.35669800	-0.00000000
C	-1.30158800	1.75323000	-0.00000000
C	-0.15625400	2.56377500	-0.00000100
C	-0.28539000	4.06319500	-0.00000500
C	0.14085500	-1.84060200	-0.00000300
C	0.74694500	-2.43357700	-1.28504400
C	0.74692600	-2.43358200	1.28504500
O	-2.49018000	2.41122100	-0.00000100
I	3.25642400	-0.11390500	0.00000700
I	-3.04432100	-0.72343600	-0.00000600
H	1.97062000	2.54608200	0.00000000
H	-0.83526800	4.41341100	0.87882900
H	-0.83526600	4.41340600	-0.87884100
H	0.69982800	4.53276900	-0.00000500
H	-0.89565700	-2.17154200	-0.00001200
H	0.61147800	-3.51973800	-1.27872800

H	0.24111800	-2.03948200	-2.17132200
H	1.81379900	-2.23314500	-1.38642700
H	0.61145000	-3.51974100	1.27872800
H	0.24109200	-2.03948200	2.17131800
H	1.81378000	-2.23315900	1.38644000
H	-3.22731900	1.77734100	-0.00000100

4 (*anti*-GS)

28

XYZ -Stationary point- [Standard orientation]

C	0.96356600	1.95851900	-0.11937400
C	1.37744000	0.63498000	-0.02142100
C	0.49543700	-0.46049500	-0.00822900
C	-0.86625200	-0.10037900	-0.09266100
C	-1.31200100	1.22805400	-0.16109100
C	-0.38708000	2.28113800	-0.19221100
C	-0.83986900	3.71141900	-0.32814800
C	1.02435000	-1.89358500	0.08903400
C	0.59967600	-2.62623300	1.37550600
C	0.78532500	-2.72903900	-1.18270700
O	-2.64694400	1.51256300	-0.28843700
Br	3.30184700	0.40180700	0.08075600
Br	-2.26640600	-1.42956600	-0.15923100
H	1.69870700	2.75265200	-0.14589700
H	-1.32713200	4.06900300	0.58404300
H	-1.56068400	3.81862800	-1.14238500
H	0.01171300	4.36372200	-0.52884700
H	2.10504100	-1.78764000	0.16400600

H	1.15924900	-3.56343100	1.45500300
H	0.82890200	-2.02639400	2.26117600
H	-0.46235100	-2.87064500	1.39580800
H	1.34115800	-3.66859500	-1.10453300
H	1.14592100	-2.20213600	-2.07089500
H	-0.26504800	-2.97580400	-1.33764500
C	-3.35492700	1.74990800	0.94125100
H	-3.31945300	0.87105000	1.59106200
H	-2.94624000	2.61406500	1.47268300
H	-4.38868300	1.95089900	0.66086300

4 (*syn*-GS)

28

XYZ -Stationary point- [Standard orientation]

C	0.67931800	1.98759500	0.12346800
C	1.21694400	0.70807100	0.02980300
C	0.43609900	-0.45975100	0.01949400
C	-0.95451100	-0.22049500	0.09513700
C	-1.52219700	1.05937200	0.16135900
C	-0.69497800	2.18990000	0.19412100
C	-1.27423900	3.57394400	0.32957200
C	0.98758600	-1.88504200	-0.05893200
C	1.69973800	-2.19504300	-1.38902400
C	1.81926100	-2.28911100	1.17280900
O	-2.87797300	1.22258800	0.28262100
Br	3.15306200	0.67793900	-0.07091700
Br	-2.21692300	-1.68564500	0.14725400
H	1.34018600	2.84483700	0.14806100

H	-1.98037000	3.62238500	1.16235500
H	-1.81696800	3.87589100	-0.57122100
H	-0.48204100	4.30441000	0.50286400
H	0.11345200	-2.53228800	-0.04234100
H	1.91757800	-3.26644100	-1.43818600
H	1.06198800	-1.94571700	-2.24214600
H	2.64236800	-1.65914400	-1.50221800
H	2.03783100	-3.36033900	1.12328700
H	1.26429100	-2.10503600	2.09729400
H	2.76981200	-1.75892600	1.23666800
C	-3.59671400	1.39132500	-0.95210000
H	-3.24427500	2.27160600	-1.49707400
H	-3.50019600	0.50599100	-1.58678300
H	-4.64256400	1.52721300	-0.67756000

5 (*anti*-GS)

28

XYZ -Stationary point- [Standard orientation]

C	1.19964200	1.89893300	0.14101500
C	1.38606100	0.52420200	0.02936000
C	0.32298700	-0.40046300	-0.01111100
C	-0.96274900	0.18347300	0.06114200
C	-1.16544500	1.57065400	0.14199600
C	-0.07536800	2.45008000	0.20075300
C	-0.27041200	3.93657700	0.35432300
C	0.60904700	-1.90155200	-0.12071800
C	0.25653100	-2.69302300	1.15221100
C	0.07327800	-2.55566700	-1.40736200

O -2.43460300 2.08215100 0.25424400

I 3.46345300 -0.06661000 -0.04140500

I -2.78759800 -0.96465700 0.11634100

H 2.05695200 2.55786100 0.19142800

H -1.05120900 4.15710700 1.08563900

H -0.56633600 4.40575800 -0.58944500

H 0.65723700 4.41010300 0.68125000

H 1.69246100 -1.96929900 -0.20509200

H 0.63350100 -3.71624200 1.05613000

H 0.72892900 -2.24513000 2.03133800

H -0.81674800 -2.74936200 1.33510200

H 0.49180500 -3.56328800 -1.49576500

H 0.38271400 -1.98926300 -2.29072300

H -1.01252800 -2.64589300 -1.42416400

C -3.03088600 2.54537100 -0.97053400

H -2.45743200 3.36908800 -1.40391700

H -3.11019900 1.73127100 -1.69651200

H -4.02872700 2.89572000 -0.70727800

5 (*syn*-GS)

28

XYZ -Stationary point- [Standard orientation]

C -1.02972000 1.91634100 0.15285600

C -1.27803000 0.54942300 0.04467800

C -0.24851700 -0.41071300 0.00813300

C 1.05823800 0.12968500 0.07066600

C 1.32127600 1.50478100 0.14845000

C 0.26456800 2.42243600 0.20993600

C	0.51185100	3.90159600	0.35857100
C	-0.46194300	-1.92426800	-0.08603700
C	-1.15109500	-2.52831500	1.15112700
C	-1.09698200	-2.37711600	-1.41351900
O	2.61181300	1.96185900	0.25247900
I	-3.38932600	0.09744900	-0.03674000
I	2.81569600	-1.12168800	0.10014300
H	-1.86040000	2.60880000	0.20259800
H	0.79515900	4.36141500	-0.59369300
H	1.31980400	4.09605700	1.06727400
H	-0.39029600	4.40461700	0.71174600
H	0.53811600	-2.35274800	-0.09086000
H	-1.12249600	-3.62035600	1.08149000
H	-0.62763400	-2.23940300	2.06717400
H	-2.19534000	-2.22999200	1.24822700
H	-1.06908500	-3.46991200	-1.47055400
H	-0.53586400	-1.98500900	-2.26688400
H	-2.13662500	-2.06640000	-1.51975000
C	3.21599700	2.40423600	-0.97619700
H	3.24887800	1.59184000	-1.70780600
H	2.67769800	3.25687400	-1.39880500
H	4.23210000	2.70550000	-0.72233000

6 (*anti*-GS)

32

XYZ -Stationary point- [Standard orientation]

C	-0.87695600	-2.06230000	-0.19774800
C	-1.62805100	-0.90541800	-0.02416600

C	-1.07714100	0.38863600	-0.00727900
C	0.32449000	0.41660800	-0.16748500
C	1.10921800	-0.73717800	-0.31509300
C	0.50400700	-2.00137500	-0.34887000
C	1.31883200	-3.24904200	-0.56838100
C	-1.96985500	1.61976400	0.16691500
C	-1.69528000	2.41078200	1.45959900
C	-2.03311100	2.51832000	-1.08265900
O	2.46312300	-0.64446100	-0.51209100
Br	-3.53437100	-1.20960400	0.17951200
Br	1.30447800	2.07998200	-0.23536900
H	-1.36916300	-3.02602400	-0.22232500
H	1.92898700	-3.49238700	0.30680600
H	2.00104900	-3.12812100	-1.41316300
H	0.66600500	-4.10086400	-0.76661500
H	-2.97568200	1.22068700	0.28443500
H	-2.48573400	3.15494600	1.59816800
H	-1.70391200	1.75080600	2.33201000
H	-0.74178400	2.93855200	1.44199500
H	-2.81857700	3.26813000	-0.94604900
H	-2.28240700	1.93389100	-1.97305700
H	-1.09890300	3.04643200	-1.27411000
C	3.27959000	-0.71406300	0.68700600
H	2.93722300	0.05510500	1.38897900
H	3.16415700	-1.69315300	1.16148700
C	4.70032900	-0.46679800	0.29536200
H	4.88717500	0.46033700	-0.24138000
C	5.70457900	-1.28823700	0.59145800

H	5.54058200	-2.22253500	1.12136500
H	6.72834000	-1.05151000	0.32122900

6 (*syn*-GS)

32

XYZ -Stationary point- [Standard orientation]

C	0.77584900	2.00656900	-0.20555200
C	1.54273000	0.85829400	-0.03806000
C	1.00040100	-0.43764400	-0.02103600
C	-0.40399500	-0.47371100	-0.17289600
C	-1.20209600	0.67004000	-0.31638000
C	-0.60580900	1.93769300	-0.35023300
C	-1.42956700	3.18065400	-0.56264000
C	1.80988000	-1.72663000	0.13723500
C	2.76596100	-1.99697500	-1.03960900
C	2.49837700	-1.85486000	1.50906800
O	-2.55594800	0.56486700	-0.50841400
Br	3.44066700	1.20555300	0.15386600
Br	-1.35635800	-2.15698300	-0.22938000
H	1.26030700	2.97434600	-0.23028400
H	-2.04206400	3.41405800	0.31359500
H	-2.11039300	3.05987900	-1.40863300
H	-0.78298100	4.03846500	-0.75531600
H	1.07830000	-2.53099500	0.10590600
H	3.18566800	-3.00262400	-0.93820400
H	2.23347400	-1.95314200	-1.99411700
H	3.59733100	-1.29288200	-1.08224700
H	2.91332200	-2.86259200	1.60938400

H	1.78142500	-1.70807700	2.32217000
H	3.31458900	-1.14483200	1.64397000
C	-3.36807700	0.62272300	0.69418200
H	-3.01354900	-0.14339600	1.39353600
H	-3.26222800	1.60242600	1.16966800
C	-4.78738100	0.35884400	0.30820700
H	-4.96534800	-0.57006000	-0.22851600
C	-5.80021300	1.16794400	0.60901700
H	-5.64531400	2.10376000	1.13896300
H	-6.82211500	0.91914300	0.34262200

7 (*anti*-GS)

32

XYZ -Stationary point- [Standard orientation]

C	1.05993500	1.97827000	-0.26335100
C	1.58457400	0.70514000	-0.06085400
C	0.79024800	-0.45691700	0.01149400
C	-0.59715800	-0.22693800	-0.13622200
C	-1.14272900	1.05626100	-0.30473100
C	-0.30995200	2.18111700	-0.38635000
C	-0.87096600	3.55935300	-0.62461400
C	1.44316000	-1.82456500	0.23659000
C	1.02882800	-2.51369300	1.54978200
C	1.37088100	-2.75799600	-0.98588700
O	-2.49521900	1.22637100	-0.47611000
H	1.72368900	2.83063200	-0.33219200
H	-1.31396800	3.97682900	0.28536300
H	-1.65263400	3.53996600	-1.38708900

H	-0.08230800	4.24068400	-0.94943800
H	2.50224300	-1.60513200	0.36117300
H	1.68415400	-3.37327300	1.72282700
H	1.14259300	-1.83360200	2.39913600
H	0.00168500	-2.87747100	1.54042600
H	1.99822500	-3.63665900	-0.80511500
H	1.75030100	-2.25632500	-1.88086600
H	0.36015500	-3.10845600	-1.19585500
C	-3.24425600	1.54093500	0.72813900
H	-3.05488100	0.75530700	1.46910800
H	-2.90799400	2.49721600	1.13873700
C	-4.69536800	1.58616300	0.37428000
H	-5.09329500	0.69251600	-0.10036900
C	-5.48934500	2.62199200	0.63469900
H	-5.11235100	3.52690400	1.10322700
H	-6.54755300	2.59849900	0.39629200
I	-2.05926800	-1.81068000	-0.18976000
I	3.73844900	0.66967300	0.10307500

7 (*syn*-GS)

32

XYZ -Stationary point- [Standard orientation]

C	0.95301500	1.94904600	-0.26644800
C	1.50479100	0.68416100	-0.07305500
C	0.72350900	-0.48554500	-0.00995800
C	-0.66882700	-0.26752300	-0.14463800
C	-1.23833800	1.00334800	-0.30995400
C	-0.41942800	2.13731400	-0.38973600

C	-0.99615500	3.50970600	-0.62415100
C	1.27576200	-1.90078900	0.18376400
C	2.13650000	-2.39474900	-0.99311900
C	1.94103000	-2.12109700	1.55488400
O	-2.59381700	1.14993300	-0.47974400
H	1.60327600	2.81216500	-0.32947500
H	-1.44793000	3.91765000	0.28583800
H	-1.77469200	3.48356300	-1.38972900
H	-0.21515400	4.20229700	-0.94349700
H	0.40195400	-2.54901000	0.18705500
H	2.36186900	-3.45675200	-0.85325900
H	1.59727100	-2.29050700	-1.93917000
H	3.08450300	-1.86366500	-1.08287100
H	2.16072500	-3.18607100	1.68041500
H	1.26978600	-1.82216000	2.36540700
H	2.87699200	-1.57399000	1.67053900
C	-3.34804300	1.45100000	0.72447900
H	-3.13849700	0.67310300	1.46819400
H	-3.03469000	2.41698900	1.13048700
C	-4.80039000	1.45861000	0.37282100
H	-5.17608600	0.55376500	-0.09874900
C	-5.62013400	2.47457700	0.63147500
H	-5.26557800	3.39019000	1.09675000
H	-6.67769800	2.42347800	0.39450400
I	-2.08331200	-1.89674700	-0.16206500
I	3.65811800	0.73978700	0.09144900

8 (*anti*-GS)

36

XYZ -Stationary point- [Standard orientation]

C	1.17058200	-1.99035700	-0.46091200
C	1.85790900	-0.84045300	-0.08887200
C	1.28034200	0.44251500	-0.06248600
C	-0.08286200	0.46730400	-0.42441700
C	-0.80202400	-0.69039500	-0.74603900
C	-0.17512500	-1.93856700	-0.80727100
C	-0.91262900	-3.16321300	-1.27782000
C	2.10832300	1.66998200	0.32566600
C	2.34044200	2.65035200	-0.83957400
C	1.62266300	2.36907100	1.60919000
O	-2.12177000	-0.61738200	-1.16057000
Br	3.71582400	-1.13430500	0.37887900
Br	-1.07690900	2.11409300	-0.54278300
H	1.68567300	-2.94164200	-0.49127900
H	-1.26294800	-3.03061700	-2.30581900
H	-1.79341300	-3.37389100	-0.66679700
H	-0.25871800	-4.03592100	-1.24721300
H	3.09285000	1.27563500	0.56997200
H	3.08093300	3.39680500	-0.53655800
H	2.73255800	2.12989600	-1.71800700
H	1.43590600	3.18179000	-1.13567500
H	2.36908200	3.10767900	1.91676400
H	1.50895600	1.65202800	2.42745200
H	0.67466800	2.89119900	1.47958200
C	-3.21422800	-0.59495000	-0.31104800

O	-4.29542800	-0.57051900	-0.83478400
C	-2.99360600	-0.62610000	1.19405600
H	-1.95583900	-0.37054700	1.41278300
C	-3.91806300	0.39385900	1.87455300
H	-3.73248000	0.39007600	2.95178400
H	-3.74635600	1.40723000	1.50454800
H	-4.96715000	0.14202000	1.70493700
C	-3.26339100	-2.04994000	1.72112600
H	-4.28855800	-2.35756100	1.49945700
H	-2.58063800	-2.78529400	1.29088100
H	-3.12897200	-2.06290000	2.80574000

8 (*syn*-GS)

36

XYZ -Stationary point- [Standard orientation]

C	1.07998700	-1.94635300	-0.47134900
C	1.78291700	-0.79992900	-0.11553200
C	1.20956400	0.48318600	-0.09037000
C	-0.15929500	0.50923300	-0.43851100
C	-0.89120500	-0.64323800	-0.74539800
C	-0.26993500	-1.89370500	-0.80242300
C	-1.01640800	-3.12084200	-1.25195600
C	1.95358900	1.77177800	0.26701600
C	2.44144000	1.81270500	1.72756900
C	3.05564800	2.14203000	-0.74304300
O	-2.21434700	-0.56438700	-1.14785400
Br	3.64080200	-1.12682700	0.32065200
Br	-1.13170700	2.17262700	-0.55218100

H	1.59023300	-2.90039600	-0.50042600
H	-1.39706700	-2.98997800	-2.26919500
H	-1.87801100	-3.33481400	-0.61495200
H	-0.35884400	-3.99127600	-1.23969300
H	1.21194200	2.56376000	0.18939900
H	2.80740400	2.81878100	1.95375600
H	1.62432600	1.59269000	2.42084800
H	3.25282400	1.11223100	1.92557200
H	3.42843500	3.14524800	-0.51514000
H	2.66286800	2.15666600	-1.76376000
H	3.90433000	1.45820300	-0.71543700
C	-3.29844700	-0.51687100	-0.28858900
O	-4.38404600	-0.48313900	-0.80256800
C	-3.06458500	-0.53532200	1.21466900
H	-2.01847000	-0.30487200	1.42122800
C	-3.95560300	0.51653500	1.89145700
H	-3.76299700	0.51744000	2.96746500
H	-3.75811300	1.52137300	1.51102400
H	-5.01241300	0.29245200	1.73109800
C	-3.36695100	-1.94558300	1.75990800
H	-4.40335900	-2.22610900	1.55544500
H	-2.71142200	-2.70351300	1.32643500
H	-3.21714800	-1.95241100	2.84254700

9 (*anti*-GS)

36

XYZ -Stationary point- [Standard orientation]

C	1.25056300	-1.84100500	-0.63576100

C	1.75914800	-0.62311400	-0.19281700
C	0.97313500	0.53809400	-0.04605000
C	-0.39538900	0.36862100	-0.36222300
C	-0.91786700	-0.87273700	-0.74979500
C	-0.09901900	-1.99094200	-0.93263600
C	-0.64308200	-3.28436500	-1.47889400
C	1.61617600	1.84968900	0.41575100
C	1.67853300	2.92819100	-0.68169500
C	1.08060000	2.37974200	1.75840300
O	-2.25112400	-1.00265900	-1.11539400
I	3.87767000	-0.66812200	0.21727000
I	-1.82269000	1.97855800	-0.35968700
H	1.91066900	-2.68907500	-0.76233900
H	-1.08232800	-3.13086700	-2.46909900
H	-1.42748500	-3.70492000	-0.84569300
H	0.15385500	-4.02436700	-1.56751000
H	2.65443700	1.58967600	0.61491300
H	2.29409400	3.76231700	-0.33049400
H	2.14144900	2.53211900	-1.59013500
H	0.69869500	3.32631100	-0.94580100
H	1.72379700	3.19679700	2.09998300
H	1.10395900	1.59776900	2.52317300
H	0.06295700	2.76455800	1.69547500
C	-3.27332300	-1.35036600	-0.25123800
O	-4.35569900	-1.52034400	-0.74659000
C	-2.98863400	-1.48995600	1.23756700
H	-1.98081800	-1.13007900	1.44875700
C	-3.99947600	-0.64430600	2.03009300

H	-3.77593700	-0.71899600	3.09759500
H	-3.95585000	0.40909800	1.74591100
H	-5.01787500	-1.00381900	1.86582200
C	-3.08155400	-2.97168000	1.64994100
H	-4.07243000	-3.37446200	1.42542500
H	-2.33538200	-3.59028500	1.14728000
H	-2.91405100	-3.05606200	2.72682700

9 (*syn*-GS)

36

XYZ -Stationary point- [Standard orientation]

C	-1.15124100	1.81246600	-0.65170800
C	-1.68505300	0.60390000	-0.20726100
C	-0.90984200	-0.56235400	-0.05663100
C	0.46300300	-0.40377900	-0.36733000
C	1.00803200	0.82548100	-0.75488400
C	0.20106000	1.95003300	-0.94448700
C	0.75853100	3.23715000	-1.49203200
C	-1.45022800	-1.92337300	0.39141500
C	-1.98665400	-1.93514200	1.83453800
C	-2.42033100	-2.56062700	-0.61990100
O	2.34579500	0.93419500	-1.11046400
I	-3.80590200	0.73265000	0.17672600
I	1.84123300	-2.05724100	-0.34141900
H	-1.79975900	2.66864400	-0.78357800
H	1.20790400	3.07532900	-2.47632300
H	1.53825000	3.65671500	-0.85226900
H	-0.03290100	3.98131500	-1.59370500

H	-0.58418800	-2.58175000	0.40877300
H	-2.18703000	-2.96919200	2.13202500
H	-1.24684300	-1.52684600	2.52943200
H	-2.91207000	-1.37176300	1.95438100
H	-2.64465700	-3.58524300	-0.30722500
H	-1.96892400	-2.60729500	-1.61516300
H	-3.36598800	-2.02366300	-0.70065500
C	3.36748400	1.25715600	-0.23605300
O	4.45997200	1.39347700	-0.71944300
C	3.06880500	1.41716700	1.24778800
H	2.04686600	1.09308800	1.44876100
C	4.04026200	0.54546500	2.06104400
H	3.80678500	0.63926600	3.12490800
H	3.96389100	-0.50890100	1.78741500
H	5.07237000	0.86795700	1.90568500
C	3.20609200	2.89859400	1.64866200
H	4.21461800	3.26391700	1.43931700
H	2.49208600	3.53816900	1.12589800
H	3.02143400	2.99937200	2.72132200

10 (*anti*-GS)

33

XYZ -Stationary point- [Standard orientation]

C	0.97379100	-2.01187900	-0.40882600
C	1.70095300	-0.87164800	-0.08403900
C	1.15201400	0.42359300	-0.04482700
C	-0.22471400	0.47420500	-0.34882600
C	-0.97533700	-0.66911800	-0.64272100

C	-0.38245600	-1.93311800	-0.70386300
C	-1.18057500	-3.14844400	-1.09155100
C	2.02040400	1.63707000	0.29553900
C	2.20635100	2.61054300	-0.88365400
C	1.61440000	2.34745900	1.60042800
O	-2.30683700	-0.55518100	-1.01229200
Br	3.57082300	-1.20017400	0.30680900
Br	-1.20264400	2.13415500	-0.40532800
H	1.46530500	-2.97549900	-0.44069000
H	-1.62225700	-3.02465900	-2.08448100
H	-2.00441400	-3.32968000	-0.39481500
H	-0.54487100	-4.03490800	-1.10383900
H	3.00982000	1.22675800	0.48846500
H	2.97415600	3.34531100	-0.62301200
H	2.54264300	2.08120200	-1.77976200
H	1.29618500	3.15611600	-1.13324400
H	2.38754300	3.07500400	1.86547600
H	1.53217700	1.63486300	2.42630500
H	0.66917100	2.88398100	1.51827500
C	-3.35022300	-0.69080700	-0.11707200
O	-4.46219800	-0.63066400	-0.56696300
C	-3.00168800	-0.89796200	1.33793400
H	-2.39465200	-1.80588300	1.41418800
H	-2.34896500	-0.07825400	1.65424800
C	-4.23194800	-0.98946900	2.23588200
H	-3.92067400	-1.14030300	3.27243900
H	-4.82677900	-0.07483200	2.18843900
H	-4.87421800	-1.82437600	1.94777700

10 (*syn*-GS)

33

XYZ -Stationary point- [Standard orientation]

C	0.87601000	-1.95978600	-0.42057100
C	1.62044700	-0.82639400	-0.10823200
C	1.07974700	0.47063000	-0.06983000
C	-0.30185600	0.52715700	-0.35961800
C	-1.06694800	-0.60768200	-0.64407800
C	-0.48271800	-1.87497800	-0.70404800
C	-1.29137100	-3.08772700	-1.07761700
C	1.86838300	1.74405700	0.24332200
C	2.40804700	1.79197800	1.68516600
C	2.94348800	2.07217200	-0.80957800
O	-2.40033300	-0.48306600	-1.00352500
Br	3.48511000	-1.19486400	0.26066400
Br	-1.25413500	2.20562000	-0.40013500
H	1.35982200	-2.92746000	-0.45141000
H	-1.73453900	-2.97012900	-2.07069000
H	-2.11493600	-3.25501700	-0.37706500
H	-0.66292600	-3.97940100	-1.08292500
H	1.14451500	2.55360500	0.17918500
H	2.81461600	2.78863500	1.88185200
H	1.60886800	1.61022800	2.40963000
H	3.20244800	1.06777300	1.86659300
H	3.34534900	3.07054100	-0.61183300
H	2.51683200	2.07856400	-1.81667800
H	3.77779200	1.37051300	-0.79853000

C	-3.43759900	-0.60432400	-0.09923200
O	-4.55260600	-0.53540800	-0.54024800
C	-3.07906900	-0.80784800	1.35374900
H	-2.48479500	-1.72425800	1.42944500
H	-2.41148800	0.00381500	1.65950600
C	-4.30234600	-0.87623900	2.26316700
H	-3.98394100	-1.02457800	3.29790400
H	-4.88476400	0.04631900	2.21474900
H	-4.95873200	-1.70393700	1.98634400

11 (*anti*-GS)

33

XYZ -Stationary point- [Standard orientation]

C	-1.16128900	1.88093900	-0.54151800
C	-1.65223300	0.63269200	-0.16727000
C	-0.84171400	-0.51345500	-0.03546400
C	0.53026800	-0.30033100	-0.30669800
C	1.02890200	0.96397200	-0.64429700
C	0.19201500	2.07351500	-0.79270200
C	0.73444900	3.41053000	-1.22327300
C	-1.46150700	-1.85377500	0.37191600
C	-1.44657900	-2.91139400	-0.74681400
C	-0.96008200	-2.39256300	1.72429400
O	2.37004900	1.12885200	-0.97009800
I	-3.78299100	0.60871700	0.17305200
I	2.00864600	-1.86425600	-0.28304100
H	-1.83789200	2.71911400	-0.64571200
H	1.25899400	3.33079600	-2.17957900

H	1.44999400	3.81064600	-0.49809100
H	-0.07497300	4.13394100	-1.33251800
H	-2.51451300	-1.63037100	0.53474300
H	-2.05330500	-3.76847800	-0.43808000
H	-1.88102300	-2.51022700	-1.66703700
H	-0.44588000	-3.27802500	-0.97550600
H	-1.58269500	-3.24201000	2.02221100
H	-1.04570400	-1.62924700	2.50334600
H	0.07417500	-2.73496400	1.69490500
C	3.31795400	1.53819800	-0.05313600
O	4.43429900	1.70768800	-0.46446100
C	2.87198300	1.72293100	1.37728800
H	2.04503200	2.44003300	1.38958300
H	2.44691800	0.77312000	1.71729600
C	4.00052500	2.17600700	2.29858400
H	3.62175600	2.28761900	3.31755800
H	4.81545500	1.44929700	2.31576600
H	4.41297500	3.13642500	1.98158000

11 (*syn*-GS)

33

XYZ -Stationary point- [Standard orientation]

C	-1.05343100	1.85530800	-0.54864100
C	-1.57399400	0.61570900	-0.17950200
C	-0.77836400	-0.53964200	-0.05185000
C	0.59929500	-0.33973900	-0.31396900
C	1.12433900	0.91225100	-0.64916600
C	0.30283900	2.03196200	-0.79795700

C	0.86195600	3.36323800	-1.22476900
C	-1.30062800	-1.92764000	0.32980500
C	-1.86563400	-2.00491200	1.75995800
C	-2.23594200	-2.54540600	-0.72542800
O	2.46949400	1.05169000	-0.96996600
I	-3.70670700	0.68684600	0.14551200
I	2.02831800	-1.94983300	-0.25624100
H	-1.71505200	2.70551400	-0.65133700
H	1.38676900	3.27907700	-2.18057500
H	1.58123500	3.75307800	-0.49766700
H	0.06170500	4.09681600	-1.33381600
H	-0.42182400	-2.56912900	0.33997300
H	-2.05828200	-3.05242700	2.01197800
H	-1.14513000	-1.61554100	2.48532500
H	-2.80049400	-1.45783400	1.88291400
H	-2.43864700	-3.58763100	-0.45974700
H	-1.76580700	-2.53884600	-1.71306300
H	-3.19389900	-2.03028500	-0.80192900
C	3.42197400	1.43900500	-0.04821600
O	4.54421500	1.58159800	-0.45364700
C	2.97232200	1.63675700	1.37925300
H	2.16730000	2.37854000	1.38530300
H	2.51624300	0.70062000	1.71700300
C	4.10763600	2.05651400	2.30793700
H	3.72555800	2.17996100	3.32429200
H	4.90032000	1.30574000	2.33084800
H	4.55076900	3.00394000	1.99330400

12 (*anti*-GS)

25

XYZ -Stationary point- [Standard orientation]

C	-1.33456500	1.57263300	-0.00000200
C	0.04791900	1.48515100	-0.00000200
C	0.75694700	0.28557300	-0.00000100
C	-0.05919000	-0.84410100	0.00000100
C	-1.45039100	-0.81729600	0.00000100
C	-2.11472900	0.41584800	-0.00000000
C	-3.61849500	0.46477100	0.00000000
C	2.27802900	0.23258400	-0.00000100
C	2.83520100	-0.42768700	1.27456700
C	2.83520100	-0.42769000	-1.27456700
O	-2.18953500	-1.96557300	0.00000300
H	-1.80159700	2.55032700	-0.00000300
H	-4.03097600	-0.04036600	0.87886400
H	-4.03097600	-0.04036700	-0.87886300
H	-3.97151800	1.49719900	-0.00000100
H	2.61636200	1.27053200	-0.00000200
H	3.92709400	-0.35725800	1.28143600
H	2.46138700	0.07052000	2.17346900
H	2.56669100	-1.48518400	1.33403700
H	3.92709300	-0.35726000	-1.28143700
H	2.46138700	0.07051600	-2.17347000
H	2.56669100	-1.48518700	-1.33403500
H	-1.60236100	-2.73432500	0.00000400
F	0.51195500	-2.08965600	0.00000300
F	0.75153900	2.65929200	-0.00000400

12 (*syn*-GS)

25

XYZ -Stationary point- [Standard orientation]

C	-0.96961400	1.64458800	0.00000400
C	0.34843200	1.22242900	0.00000400
C	0.74038300	-0.11578700	0.00000000
C	-0.32815800	-1.00912400	-0.00000300
C	-1.67176100	-0.64273400	-0.00000400
C	-2.01144700	0.71487900	0.00000000
C	-3.45632600	1.13442900	-0.00000000
C	2.18613600	-0.59241500	0.00000000
C	2.93542400	-0.16194600	-1.27444400
C	2.93542200	-0.16195600	1.27444900
O	-2.67097800	-1.57367300	-0.00000700
H	-1.18248900	2.70715800	0.00000700
H	-3.98104100	0.74727000	0.87893300
H	-3.98103900	0.74727600	-0.87893700
H	-3.54192400	2.22218200	0.00000300
H	2.15081200	-1.68379400	-0.00000400
H	3.93788300	-0.60053000	-1.28100800
H	2.41439800	-0.50237500	-2.17372800
H	3.04289500	0.92363600	-1.33293200
H	3.93788000	-0.60053900	1.28101200
H	2.41439300	-0.50239100	2.17372900
H	3.04289100	0.92362600	1.33294500
H	-2.29339700	-2.46410300	-0.00000900
F	-0.07863700	-2.35688600	-0.00000700

F 1.31814900 2.18775100 0.00000800

13 (*anti*-GS)

25

XYZ -Stationary point- [Standard orientation]

C	1.24372900	-1.59305800	-0.00000100
C	-0.15292000	-1.58217700	-0.00000200
C	-0.85635100	-0.37536800	-0.00000100
C	-0.11734500	0.81476300	0.00000000
C	1.27587400	0.79321800	0.00000100
C	1.99136700	-0.41577300	0.00000000
C	3.49717400	-0.41638200	0.00000000
C	-2.37893700	-0.35928800	-0.00000100
C	-2.94966100	0.30068000	1.26766400
C	-2.94966000	0.30068500	-1.26766300
O	2.01589300	1.94974400	0.00000200
H	1.76929300	-2.54313800	-0.00000200
H	3.89829200	0.09976200	0.87833800
H	3.89829200	0.09976300	-0.87833700
H	3.88237600	-1.43810800	-0.00000000
H	-2.70240000	-1.40582200	-0.00000300
H	-4.04224500	0.23305800	1.27639800
H	-2.57363400	-0.18574900	2.17231800
H	-2.68224300	1.36081700	1.32094000
H	-4.04224500	0.23306300	-1.27639800
H	-2.57363400	-0.18574100	-2.17232000
H	-2.68224300	1.36082200	-1.32093600
H	1.42591900	2.71488600	0.00000300

H	-0.62515500	1.77567500	0.00000100
H	-0.69713500	-2.52104400	-0.00000300

13 (*syn*-GS)

25

XYZ -Stationary point- [Standard orientation]

C	0.94046500	-1.55039700	0.00000400
C	-0.42094900	-1.24855000	0.00000400
C	-0.85319100	0.08257500	-0.00000000
C	0.12034100	1.08613600	-0.00000400
C	1.48059700	0.77341700	-0.00000300
C	1.92288800	-0.55701500	0.00000000
C	3.39388200	-0.87949000	0.00000000
C	-2.33201900	0.44787700	-0.00000000
C	-3.04734500	-0.05257700	-1.26740600
C	-3.04734200	-0.05256600	1.26741200
O	2.44361200	1.75197800	-0.00000700
H	1.25483100	-2.58974100	0.00000700
H	3.89616200	-0.46132400	0.87842500
H	3.89616100	-0.46132900	-0.87842700
H	3.55184700	-1.95998600	0.00000300
H	-2.38966800	1.54204300	-0.00000500
H	-4.08995200	0.28117400	-1.27617400
H	-2.56371000	0.32579900	-2.17264400
H	-3.04736500	-1.14571400	-1.31924500
H	-4.08994900	0.28118400	1.27617900
H	-2.56370500	0.32581800	2.17264600
H	-3.04736100	-1.14570300	1.31926100

H	2.02327000	2.62201200	-0.00000900
H	-0.18036900	2.13105300	-0.00000600
H	-1.14303900	-2.05756900	0.00000600

XYZ coordinates of Transition structures

1 (TS1)

25

XYZ -Stationary point- [Standard orientation]

C	-0.68405500	-1.90659800	0.09320000
C	0.53002700	-1.24690000	-0.09030600
C	0.66422900	0.15890300	-0.09503000
C	-0.59519000	0.81437900	-0.10257400
C	-1.82756900	0.16819100	0.07606900
C	-1.87981700	-1.22163200	0.22293100
C	-3.19175900	-1.92322100	0.43996100
C	2.02826400	0.90831300	-0.08269200
C	1.99754700	2.30170700	0.59738100
C	3.17133900	0.13579400	0.62273500
O	-3.01688800	0.82588500	0.10165800
H	-0.69092600	-2.98903400	0.11848000
H	-3.88965800	-1.72017100	-0.37794300
H	-3.67395200	-1.58182400	1.36101200
H	-3.04368100	-3.00225300	0.50784700
H	2.32278100	1.05449600	-1.12992200
H	3.02442400	2.60075100	0.81475900
H	1.56882800	3.08470100	-0.01874900
H	1.45731900	2.27435300	1.54845600
H	4.00409200	0.82382300	0.77985500

H	3.55689800	-0.70115900	0.05241300
H	2.85787900	-0.23107900	1.60508300
H	-2.87685400	1.77379400	-0.04231100
Cl	-0.78523800	2.54118800	-0.48818200
Cl	1.85817100	-2.37219400	-0.46018800

1 (TS2)

25

XYZ -Stationary point- [Standard orientation]

C	0.68405500	-1.90659800	0.09320000
C	-0.53002700	-1.24690000	-0.09030600
C	-0.66422900	0.15890300	-0.09503000
C	0.59519000	0.81437900	-0.10257400
C	1.82756900	0.16819100	0.07606900
C	1.87981700	-1.22163200	0.22293100
C	3.19175900	-1.92322100	0.43996100
C	-2.02826400	0.90831300	-0.08269200
C	-3.17133900	0.13579400	0.62273500
C	-1.99754700	2.30170700	0.59738100
O	3.01688800	0.82588500	0.10165800
H	0.69092600	-2.98903400	0.11848000
H	3.67395200	-1.58182400	1.36101200
H	3.88965800	-1.72017100	-0.37794300
H	3.04368100	-3.00225300	0.50784700
H	-2.32278100	1.05449600	-1.12992200
H	-4.00409200	0.82382300	0.77985500
H	-3.55689800	-0.70115900	0.05241300
H	-2.85787900	-0.23107900	1.60508300

H	-3.02442400	2.60075100	0.81475900
H	-1.56882800	3.08470100	-0.01874900
H	-1.45731900	2.27435300	1.54845600
H	2.87685400	1.77379400	-0.04231100
Cl	0.78523800	2.54118800	-0.48818200
Cl	-1.85817100	-2.37219400	-0.46018800

2 (TS1)

25

XYZ -Stationary point- [Standard orientation]

C	-1.05136500	1.81198800	0.23780500
C	-1.25259600	0.44327800	0.06242100
C	-0.20743900	-0.50679500	0.08736400
C	1.07311700	0.10847800	0.04576800
C	1.29684600	1.48323300	0.21445500
C	0.21394400	2.35626800	0.37525800
C	0.43864100	3.82652700	0.59580400
C	-0.42278600	-2.04365600	0.20405100
C	0.71402000	-2.79402800	0.94426700
C	-1.71367500	-2.43934800	0.96047800
O	2.52990700	2.05420300	0.21991000
Br	-3.09174000	0.05750700	-0.44490300
H	-1.90731200	2.47441600	0.25102400
H	1.01630000	4.26662000	-0.22262000
H	1.00372200	4.00511700	1.51570500
H	-0.51412900	4.35361700	0.66917100
H	-0.49104400	-2.44828400	-0.81397000
H	0.34060000	-3.77382400	1.24696400

H	1.59663100	-2.97234100	0.34050200
H	1.01749800	-2.26548500	1.85289800
H	-1.68860100	-3.51563800	1.14248600
H	-2.62900700	-2.23275400	0.42000600
H	-1.76802300	-1.94045100	1.93335600
H	3.20671800	1.38004700	0.05419100
Br	2.69303000	-0.83580200	-0.48409800

2 (TS2)

25

XYZ -Stationary point- [Standard orientation]

C	1.05137000	1.81198500	0.23780600
C	1.25259800	0.44327500	0.06242200
C	0.20743800	-0.50679600	0.08736100
C	-1.07311600	0.10848100	0.04576400
C	-1.29684200	1.48323800	0.21444900
C	-0.21393700	2.35626900	0.37525400
C	-0.43863100	3.82652900	0.59579800
C	0.42277600	-2.04365900	0.20404000
C	1.71366700	-2.43936500	0.96045700
C	-0.71403300	-2.79402300	0.94426000
O	-2.52990100	2.05421000	0.21989900
H	1.90731900	2.47441000	0.25102900
H	-1.00372900	4.00512100	1.51568700
H	-1.01627200	4.26662600	-0.22263700
H	0.51414100	4.35361400	0.66918300
H	0.49102600	-2.44828100	-0.81398400
H	1.68859100	-3.51565800	1.14244600

H	2.62899800	-2.23276300	0.41998500
H	1.76801800	-1.94048500	1.93334200
H	-0.34060800	-3.77380500	1.24699500
H	-1.59662900	-2.97236700	0.34048000
H	-1.01753900	-2.26545700	1.85286800
H	-3.20671400	1.38005500	0.05418700
Br	3.09174400	0.05750300	-0.44489400
Br	-2.69303300	-0.83579700	-0.48408900

3 (TS1)

25

XYZ -Stationary point- [Standard orientation]

C	-1.17570200	1.81648400	0.37301600
C	-1.25577300	0.42882500	0.23808900
C	-0.12152800	-0.41081400	0.31654400
C	1.10486900	0.30623700	0.22835500
C	1.19652900	1.70055100	0.36927100
C	0.03539400	2.47261400	0.51915100
C	0.12212900	3.96084400	0.71979800
C	-0.19610000	-1.94272000	0.57465500
C	1.00067600	-2.50265100	1.38319500
C	-1.43805100	-2.36868000	1.39107000
O	2.36408400	2.39755600	0.35974400
I	-3.21876700	-0.16788900	-0.46393000
I	2.94618800	-0.57277600	-0.51158200
H	-2.08331600	2.40612200	0.35349800
H	0.66214300	4.44245600	-0.10102100
H	0.66150700	4.20436300	1.64035800

H	-0.87608900	4.39807700	0.78037100
H	-0.23010500	-2.45602400	-0.39485200
H	0.73186600	-3.49128600	1.76122800
H	1.91219200	-2.62765000	0.81001000
H	1.22298600	-1.86941400	2.24752100
H	-1.34852900	-3.43206000	1.62446700
H	-2.38372600	-2.24064100	0.87854200
H	-1.48430900	-1.82248500	2.33872800
H	3.11472100	1.79918900	0.20645400

3 (TS2)

25

XYZ -Stationary point- [Standard orientation]

C	1.17570200	1.81648600	0.37302100
C	1.25577300	0.42882700	0.23809700
C	0.12152700	-0.41081100	0.31655300
C	-1.10486800	0.30623900	0.22836000
C	-1.19653000	1.70055200	0.36927500
C	-0.03539500	2.47261600	0.51915600
C	-0.12212900	3.96084600	0.71980300
C	0.19609700	-1.94271500	0.57467300
C	1.43804700	-2.36867000	1.39109200
C	-1.00068100	-2.50263500	1.38322100
O	-2.36408600	2.39755600	0.35974600
I	3.21876000	-0.16789200	-0.46393700
I	-2.94617800	-0.57278000	-0.51159100
H	2.08331600	2.40612400	0.35350100
H	-0.66149900	4.20436500	1.64036800

H	-0.66215200	4.44245700	-0.10101100
H	0.87608800	4.39807900	0.78036700
H	0.23010000	-2.45602600	-0.39483000
H	1.34853100	-3.43205200	1.62448700
H	2.38372500	-2.24062200	0.87857100
H	1.48429500	-1.82247700	2.33875100
H	-0.73186900	-3.49126000	1.76127700
H	-1.91219400	-2.62764900	0.81003600
H	-1.22299500	-1.86937900	2.24753300
H	-3.11472100	1.79918700	0.20645800

4 (TS1)

28

XYZ -Stationary point- [Standard orientation]

C	-0.92752800	1.89068500	0.10997500
C	-1.34188400	0.56357600	0.03489600
C	-0.45970700	-0.54024800	0.08365500
C	0.89724400	-0.14312300	-0.06521700
C	1.33943300	1.18422200	0.04430000
C	0.41718200	2.22740100	0.17948600
C	0.86948900	3.65907800	0.28511100
C	-0.91227800	-2.00789200	0.32706800
C	0.13786700	-2.88491800	1.05220600
C	-2.18718500	-2.12598400	1.19779900
O	2.66682400	1.50551000	-0.08081800
Br	-3.23792800	0.43087200	-0.38444700
Br	2.27315100	-1.34642400	-0.71058800
H	-1.66811000	2.67989400	0.09547900

H	1.51842000	3.92906500	-0.55240100
H	1.44067300	3.83184900	1.20194100
H	0.01066800	4.33257200	0.29010000
H	-1.12265000	-2.46473300	-0.64845100
H	-0.35065600	-3.80362500	1.38310100
H	0.97774900	-3.17786200	0.43399600
H	0.52604500	-2.38033100	1.94273300
H	-2.31812100	-3.17350900	1.47667100
H	-3.09851900	-1.81555000	0.70148900
H	-2.08572700	-1.54867100	2.12242800
C	3.44130500	1.41172700	1.12696300
H	3.04814500	2.08329300	1.89637800
H	3.45651800	0.38718500	1.50943700
H	4.45456900	1.71352600	0.86239100

4 (TS2)

28

XYZ -Stationary point- [Standard orientation]

C	0.85444000	1.87067100	0.34019100
C	1.31386900	0.58361300	0.08302100
C	0.48744100	-0.56466400	0.11211200
C	-0.89131500	-0.22370700	0.11360300
C	-1.38113800	1.07215300	0.35234100
C	-0.49305200	2.13458700	0.55094400
C	-0.96459400	3.51742200	0.91850100
C	1.02835300	-2.02092000	0.19364500
C	2.39085700	-2.13688000	0.91972800
C	0.09915600	-3.00513100	0.94749600

O	-2.73115900	1.28323300	0.46500900
Br	3.16053000	0.59104600	-0.53230200
Br	-2.27977500	-1.46400800	-0.42244000
H	1.56161200	2.69007300	0.37129600
H	-1.84171400	3.47451700	1.56690200
H	-1.23617800	4.10161900	0.03315400
H	-0.17330200	4.06099800	1.43867800
H	1.15726100	-2.39149900	-0.83142700
H	2.60243200	-3.19483300	1.08748900
H	3.22963100	-1.73227900	0.36673400
H	2.35605900	-1.64797100	1.89862600
H	0.67145200	-3.90342300	1.18732500
H	-0.76569300	-3.32401100	0.37824400
H	-0.24972400	-2.57688400	1.89222800
C	-3.37819100	1.88062700	-0.67198900
H	-2.98386300	2.87864900	-0.87956200
H	-3.26598600	1.25114600	-1.55895200
H	-4.43407900	1.95509200	-0.41241600

5 (TS1)

28

XYZ -Stationary point- [Standard orientation]

C	1.12855300	1.85056700	-0.18851400
C	1.33651200	0.47180000	-0.18622600
C	0.28635600	-0.46740900	-0.31956300
C	-0.99632200	0.11734300	-0.13074600
C	-1.22032500	1.50185200	-0.16739300
C	-0.14715300	2.39602100	-0.25839900

C	-0.36580700	3.88464600	-0.31140600
C	0.50414800	-1.95333200	-0.71732900
C	-0.66897200	-2.56597500	-1.51792600
C	1.73732400	-2.16477000	-1.62691800
O	-2.48442400	2.01851500	-0.01511200
I	3.36508300	-0.00752400	0.41172500
I	-2.69595400	-0.97389900	0.65219200
H	1.97758900	2.51807300	-0.11718400
H	-1.06476200	4.20947600	0.46326800
H	-0.78507000	4.19278500	-1.27449000
H	0.57824300	4.41523600	-0.17385400
H	0.64436200	-2.54278300	0.19770500
H	-0.34681800	-3.52873100	-1.92126500
H	-1.56308400	-2.75262000	-0.93496600
H	-0.93839100	-1.92693600	-2.36503100
H	1.73040500	-3.19693200	-1.98457900
H	2.69219100	-2.00982700	-1.13869700
H	1.69000500	-1.50989100	-2.50295000
C	-3.23923800	2.16063800	-1.23094200
H	-2.74279400	2.84950200	-1.92093000
H	-3.38322300	1.19245000	-1.71940500
H	-4.20752700	2.56924100	-0.94217000

5 (TS2)

28

XYZ -Stationary point- [Standard orientation]

C	1.12700800	1.78181600	0.49104000
C	1.33705500	0.42360400	0.26729200

C	0.29581700	-0.53466700	0.32776800
C	-0.99346000	0.05976700	0.26952500
C	-1.22079300	1.43254300	0.47014600
C	-0.14680800	2.30398300	0.68621700
C	-0.33975000	3.75383600	1.04996100
C	0.54125200	-2.05540400	0.53333500
C	1.83382300	-2.36343500	1.32514600
C	-0.57517200	-2.76739300	1.33480600
O	-2.50933200	1.90283000	0.52633500
I	3.32861000	0.05772100	-0.50962700
I	-2.75229600	-0.96946700	-0.46026600
H	1.97491500	2.45501100	0.51355000
H	-1.23597100	3.89180300	1.65783800
H	-0.43956500	4.38689700	0.16222800
H	0.52049300	4.11847800	1.61521200
H	0.61856300	-2.53221000	-0.45216200
H	1.85990300	-3.43398600	1.54094700
H	2.75347400	-2.12834100	0.80270600
H	1.83735200	-1.83170800	2.28211300
H	-0.20654100	-3.74706800	1.64683600
H	-1.48780300	-2.93903200	0.77653800
H	-0.82728100	-2.20614500	2.23994500
C	-2.95433200	2.69614800	-0.58891900
H	-2.44026500	3.65929600	-0.62827500
H	-2.80504300	2.16017000	-1.52994800
H	-4.02007100	2.86192500	-0.43175300

6 (TS1)

32

XYZ -Stationary point- [Standard orientation]

C	0.82944600	-1.93652200	0.39136700
C	1.59710100	-0.81771500	0.08735500
C	1.09703900	0.50579000	0.10993600
C	-0.32228300	0.53399600	0.15546600
C	-1.12288900	-0.58625800	0.43960700
C	-0.53298200	-1.83826400	0.64421700
C	-1.33413100	-3.04452000	1.05994900
C	1.99892100	1.77283400	0.14350900
C	1.37975500	2.97679300	0.89717600
C	3.36572300	1.54224000	0.83298900
O	-2.47931200	-0.44366400	0.58778400
Br	3.35672100	-1.31448800	-0.58026900
Br	-1.35522700	2.08480600	-0.37447600
H	1.30139500	-2.91031400	0.42501400
H	-1.78247100	-3.54985000	0.19817500
H	-2.14441400	-2.76448100	1.73554200
H	-0.69289100	-3.76887000	1.56608100
H	2.18903800	2.08029800	-0.89283600
H	2.17361800	3.69568700	1.10922700
H	0.61608600	3.50512200	0.33896700
H	0.95309200	2.66794400	1.85641700
H	3.85299500	2.51063000	0.96309600
H	4.05111300	0.91787000	0.27325200
H	3.23351100	1.10281600	1.82695000
C	-3.29309400	-0.85689000	-0.54127900

H	-2.94518300	-0.32423500	-1.43391900
H	-3.18449500	-1.93115800	-0.71329700
C	-4.71356000	-0.50117800	-0.24208300
H	-4.89713200	0.54145000	0.00630200
C	-5.72166300	-1.36847200	-0.29518500
H	-5.56155700	-2.41601100	-0.53530000
H	-6.74481300	-1.05972300	-0.10802700

6 (TS2)

32

XYZ -Stationary point- [Standard orientation]

C	0.94079600	-1.97121400	-0.00013600
C	1.64786300	-0.77224700	-0.03152600
C	1.03578800	0.50079300	-0.09143800
C	-0.36051000	0.43177200	0.16708400
C	-1.09740400	-0.76281800	0.16776800
C	-0.44650500	-1.99427700	0.03636900
C	-1.21539700	-3.28819300	0.04589300
C	1.78777600	1.81327800	-0.45214300
C	2.98736000	1.59663600	-1.40711500
C	0.91096100	2.87379300	-1.16227800
O	-2.45036800	-0.76696700	0.39543700
Br	3.55095600	-1.05798100	0.25938900
Br	-1.37565600	1.94685400	0.82402400
H	1.48337800	-2.90759100	0.01433200
H	-1.87256000	-3.36954500	-0.82502100
H	-1.84980100	-3.36207900	0.93310200
H	-0.53272400	-4.13967900	0.03472900

H	2.16878100	2.25333300	0.47831500
H	3.32727500	2.57244700	-1.75995100
H	3.84137600	1.11163800	-0.95025200
H	2.69011000	1.01403400	-2.28500600
H	1.56795100	3.63980800	-1.57901500
H	0.20778900	3.37965500	-0.51181600
H	0.35321800	2.43159200	-1.99396700
C	-3.27914300	-0.55169100	-0.77616400
H	-3.00012700	0.40388400	-1.23578500
H	-3.10504600	-1.35114400	-1.50331200
C	-4.70719600	-0.52048400	-0.33684400
H	-4.94667400	0.20381900	0.43819100
C	-5.65949500	-1.29177200	-0.85569900
H	-5.44248500	-2.02621300	-1.62647600
H	-6.69235500	-1.21025600	-0.53365400

7 (TS1)

32

XYZ -Stationary point- [Standard orientation]

C	1.00446800	-1.84476000	0.55251000
C	1.55986500	-0.59686900	0.28239300
C	0.80931400	0.60337100	0.32855800
C	-0.59203400	0.36922200	0.30564100
C	-1.16894000	-0.88910300	0.55177900
C	-0.35735400	-2.00659800	0.78164000
C	-0.91705400	-3.34428700	1.19318500
C	1.45070400	2.01014400	0.48630300
C	0.57763500	3.01016400	1.28251500

C	2.79501200	1.98650900	1.25068900
O	-2.53460900	-1.01109400	0.64039900
I	3.55723200	-0.78965800	-0.54055300
I	-2.02554100	1.81052500	-0.43569200
H	1.64508000	-2.71728900	0.58369500
H	-1.17204900	-3.96343900	0.32669300
H	-1.82035800	-3.22586400	1.79413200
H	-0.18007900	-3.89598000	1.78074700
H	1.62966600	2.42580100	-0.51353400
H	1.19922600	3.86187600	1.56746800
H	-0.26510500	3.40810500	0.72972700
H	0.19984000	2.55490800	2.20325000
H	3.11152600	3.01757600	1.42423200
H	3.60686000	1.49512500	0.72777300
H	2.67580300	1.50679200	2.22753600
C	-3.19678200	-1.66284800	-0.47639800
H	-2.93395300	-1.12350500	-1.39378900
H	-2.85696100	-2.69713600	-0.56929400
C	-4.67098500	-1.60182400	-0.23785800
H	-5.08231000	-0.60890700	-0.07321800
C	-5.46757400	-2.66803000	-0.24836900
H	-5.07806400	-3.67022700	-0.40494100
H	-6.53950700	-2.57554600	-0.10822000

7 (TS2)

32

XYZ -Stationary point- [Standard orientation]

C	1.15320100	1.91350000	0.06884700

C	1.60296600	0.59704800	0.16727700
C	0.73094400	-0.50761800	0.31385600
C	-0.62347200	-0.17691700	0.03109500
C	-1.09207100	1.14392900	-0.03023300
C	-0.20115700	2.22098300	0.04679000
C	-0.68655200	3.64463800	-0.01490100
C	1.18505600	-1.90568200	0.81681400
C	2.38395300	-1.84238800	1.79255500
C	0.09530100	-2.67155900	1.60327600
O	-2.41892400	1.42029100	-0.26131800
I	3.71576300	0.45897800	-0.30081800
I	-2.04795900	-1.60051400	-0.76569400
H	1.87204100	2.71902600	-0.00817900
H	-1.28664900	3.90150200	0.86339900
H	-1.31642100	3.80863000	-0.89332600
H	0.15738700	4.33543300	-0.05926400
H	1.47657500	-2.51124700	-0.05074500
H	2.53529000	-2.83661100	2.21917700
H	3.32329700	-1.55112800	1.33760400
H	2.17566400	-1.15521000	2.61895900
H	0.55844500	-3.53778100	2.08114300
H	-0.71988900	-3.04597200	0.99555500
H	-0.32674700	-2.04427200	2.39510300
C	-3.25540500	1.46310000	0.92422600
H	-3.33070600	0.46214500	1.35998900
H	-2.78565500	2.12899200	1.65835600
C	-4.59969400	1.98498800	0.53078400
H	-4.60369400	2.94708000	0.02340400

C	-5.74395900	1.35827800	0.79294400
H	-5.76434500	0.39304700	1.29096500
H	-6.70148900	1.79161100	0.52354900

8 (TS1)

36

XYZ -Stationary point- [Standard orientation]

C	1.21338700	-1.93300000	-0.20984900
C	1.88149700	-0.72722700	-0.01341500
C	1.23939700	0.53231100	0.02551900
C	-0.10472300	0.46077400	-0.43153000
C	-0.79803100	-0.74622700	-0.58452100
C	-0.15362200	-1.97663200	-0.44638500
C	-0.88265500	-3.27667700	-0.64741200
C	1.90351400	1.83526100	0.55342400
C	0.91087900	2.84272900	1.18425900
C	2.96362700	1.58734300	1.65459200
O	-2.10732300	-0.75500700	-1.04120200
Br	3.80958100	-0.96746000	-0.04171700
Br	-1.05101600	1.98687700	-1.14894700
H	1.77299500	-2.85928000	-0.20176200
H	-1.41473900	-3.28638000	-1.60246800
H	-1.62664300	-3.44394000	0.13709300
H	-0.18255400	-4.11318100	-0.63134600
H	2.39980800	2.32856400	-0.29190700
H	1.48457200	3.59219500	1.73268400
H	0.29991800	3.37678300	0.46657600
H	0.24759400	2.34850300	1.90121800

H	3.22261100	2.54795000	2.10391200
H	3.88663600	1.14827900	1.29612700
H	2.56445100	0.94882700	2.44912400
C	-3.22232900	-0.69649500	-0.22700100
O	-4.28991100	-0.74479400	-0.77759400
C	-3.04820600	-0.58261100	1.28002800
H	-1.98514700	-0.56714200	1.52402300
C	-3.69561000	-1.80282900	1.95886100
H	-3.24894500	-2.74195500	1.62311700
H	-3.55899600	-1.73175200	3.04102400
H	-4.76717600	-1.83870700	1.74891700
C	-3.69144100	0.72631500	1.77214900
H	-3.23627200	1.60506200	1.30955200
H	-4.76132200	0.73781700	1.55093300
H	-3.56283000	0.80897000	2.85449200

8 (TS2)

36

XYZ -Stationary point- [Standard orientation]

C	0.93284100	-1.83064400	-0.73609300
C	1.74769700	-0.84414500	-0.18994900
C	1.38316300	0.52145500	-0.11237300
C	-0.00742100	0.71346400	-0.33145700
C	-0.85415500	-0.28823500	-0.82885300
C	-0.36805700	-1.55888700	-1.13840500
C	-1.21944200	-2.59661900	-1.81698800
C	2.39865300	1.67627200	0.12519300
C	3.82400200	1.36509600	-0.39285100

C	2.01669200	3.01487100	-0.55668400
O	-2.16578700	0.02075500	-1.15305100
Br	3.33564400	-1.60497400	0.63314600
Br	-0.94759000	2.31914400	0.18676800
H	1.31490500	-2.83781600	-0.84142700
H	-1.67178800	-2.19480000	-2.72765400
H	-2.03547800	-2.93918000	-1.17483500
H	-0.61711200	-3.46614000	-2.08413900
H	2.46103700	1.85090500	1.20691000
H	4.41164700	2.28411600	-0.35317900
H	4.36015500	0.62443200	0.18756300
H	3.79942000	1.03431500	-1.43607400
H	2.90366200	3.65039000	-0.58518600
H	1.24688300	3.57362000	-0.03787700
H	1.69304200	2.85899900	-1.59018800
C	-3.27684300	-0.32431600	-0.40398900
O	-4.34446800	-0.02917300	-0.87096400
C	-3.10082400	-1.01774000	0.93925200
H	-2.05767600	-1.31275600	1.06337400
C	-3.99220300	-2.26965700	0.99322300
H	-3.76772500	-2.96993800	0.18522700
H	-3.83538500	-2.78689400	1.94327400
H	-5.04702400	-1.99521200	0.92125900
C	-3.47498100	-0.04126300	2.07184900
H	-2.83893100	0.84554200	2.07694700
H	-4.51510500	0.27992400	1.97542800
H	-3.35911400	-0.54602200	3.03443600

9 (TS1)

36

XYZ -Stationary point- [Standard orientation]

C	-1.19522600	1.84513000	-0.36400900
C	-1.72016100	0.61396200	0.02879900
C	-0.91581300	-0.52006600	0.29215400
C	0.41486800	-0.37638600	-0.19138500
C	0.95105600	0.86758800	-0.54232900
C	0.16354800	2.02007200	-0.59176000
C	0.73945700	3.35397600	-0.98085000
C	-1.40233100	-1.75576700	1.09714300
C	-0.28204800	-2.46170700	1.89641500
C	-2.46890300	-1.40570700	2.16175900
O	2.25736200	0.96299300	-1.00824200
I	-3.87505500	0.58370900	-0.18877900
I	1.61782100	-2.04380200	-0.85686300
H	-1.86041000	2.68474200	-0.51772500
H	1.27412100	3.29126800	-1.93277900
H	1.45332400	3.71554000	-0.23434900
H	-0.05205700	4.09888400	-1.07669900
H	-1.83704400	-2.47936700	0.39602100
H	-0.74349600	-3.18574500	2.57158900
H	0.43005700	-3.00849600	1.28970800
H	0.26980900	-1.74340500	2.51125100
H	-2.63494000	-2.28584000	2.78693600
H	-3.43412600	-1.12222000	1.75893500
H	-2.11797100	-0.59839600	2.81257800
C	3.34010800	1.29193800	-0.21805000

O	4.40096200	1.39179200	-0.77627200
C	3.14381600	1.48206200	1.27795600
H	2.07652100	1.50936200	1.50177600
C	3.78406500	2.80634700	1.72191800
H	3.34692000	3.66306000	1.20214100
H	3.62694400	2.94576700	2.79471100
H	4.85927100	2.80277600	1.52948800
C	3.76915400	0.28553400	2.02143800
H	3.31746000	-0.66247000	1.72108800
H	4.84347400	0.23029400	1.82804600
H	3.61944900	0.40898700	3.09719400

9 (TS2)

36

XYZ -Stationary point- [Standard orientation]

C	-1.16731700	-1.60799200	0.97379400
C	-1.71730100	-0.46019600	0.40743900
C	-1.01060600	0.76401800	0.31280400
C	0.39323000	0.59933300	0.47175900
C	0.95872200	-0.57966600	0.98038300
C	0.16539200	-1.66281200	1.36449800
C	0.70956100	-2.83038000	2.14298300
C	-1.70862500	2.14331000	0.15007300
C	-3.13166200	2.18179300	0.75370000
C	-0.96487900	3.30902600	0.84885600
O	2.32145700	-0.65366500	1.24329100
I	-3.59434300	-0.89965500	-0.58089500
I	1.85283500	1.98100800	-0.31025900

H	-1.78628800	-2.48558600	1.10915200
H	0.89581300	-2.53247800	3.18061700
H	1.65475400	-3.20552200	1.74916000
H	-0.00913300	-3.65170800	2.15242900
H	-1.78232900	2.37081000	-0.92090500
H	-3.50389400	3.20671300	0.69129300
H	-3.85746900	1.55571200	0.24881200
H	-3.11053400	1.89941800	1.81124200
H	-1.65849400	4.14521200	0.95955400
H	-0.10804300	3.68399400	0.30124600
H	-0.63356200	3.02252700	1.85155500
C	3.23670400	-1.27741900	0.41389700
O	4.34387800	-1.42769400	0.85852100
C	2.81680600	-1.69967900	-0.98820100
H	1.85254000	-1.24641500	-1.22333300
C	2.67656400	-3.23221700	-1.06913400
H	1.87490700	-3.61130200	-0.43335100
H	2.44685600	-3.51538700	-2.09966800
H	3.60995700	-3.72344800	-0.78181700
C	3.86421700	-1.20142400	-1.99761300
H	3.99410500	-0.11857900	-1.94331800
H	4.83263200	-1.67335100	-1.81710700
H	3.54056800	-1.45563800	-3.01036100

10 (TS1)

33

XYZ -Stationary point- [Standard orientation]

C	1.09152600	-1.94005200	-0.11716000

C	1.74702500	-0.71771900	0.00801100
C	1.08688200	0.53325500	0.03291000
C	-0.27318800	0.42642700	-0.36656800
C	-0.94849200	-0.79603600	-0.46129300
C	-0.28246600	-2.01152300	-0.30004000
C	-1.00399000	-3.32580400	-0.41834700
C	1.75310000	1.86172100	0.49073900
C	0.77507500	2.87540200	1.13415300
C	2.86536400	1.66378800	1.55006000
O	-2.27423700	-0.84112300	-0.86909200
Br	3.67603200	-0.93010300	-0.09492300
Br	-1.28835900	1.91629500	-1.06708800
H	1.66598100	-2.85704400	-0.09593200
H	-1.54231700	-3.39552200	-1.36753100
H	-1.74276400	-3.45140600	0.37959500
H	-0.29865500	-4.15581500	-0.35790600
H	2.20281200	2.33513500	-0.39130200
H	1.36228400	3.65365200	1.62516400
H	0.11949600	3.37296700	0.42993100
H	0.15813000	2.39753300	1.90189200
H	3.12137800	2.64004200	1.96598000
H	3.78191600	1.23713600	1.16120300
H	2.51631700	1.03543500	2.37567100
C	-3.33315100	-0.78506500	0.01428600
O	-4.43595500	-0.87983900	-0.45275700
C	-3.01314800	-0.60926900	1.47974500
H	-2.31581100	-1.39961100	1.77560400
H	-2.46031900	0.32895600	1.59434000

C	-4.25622600	-0.61548500	2.36435000
H	-4.80048600	-1.55856800	2.27910100
H	-3.96569700	-0.48259700	3.40936400
H	-4.93915300	0.19282700	2.09493400

10 (TS2)

33

XYZ -Stationary point- [Standard orientation]

C	0.92386800	-1.84598000	-0.68873500
C	1.66126300	-0.78527000	-0.17498100
C	1.18398700	0.54512600	-0.09366700
C	-0.21890300	0.62043200	-0.29569200
C	-0.98309600	-0.45393300	-0.77505300
C	-0.40171700	-1.68618600	-1.07256400
C	-1.17993800	-2.79758200	-1.72275600
C	2.12359300	1.77326200	0.07625100
C	3.51121800	1.57532300	-0.57697900
C	1.58811200	3.08965100	-0.54279900
O	-2.32317600	-0.26929700	-1.08398900
Br	3.31239100	-1.38408100	0.65893700
Br	-1.28761100	2.13820800	0.24277100
H	1.38487300	-2.82096900	-0.78103000
H	-1.55719400	-2.48381300	-2.70050200
H	-2.04685300	-3.09800700	-1.12736000
H	-0.54534800	-3.67359500	-1.86426400
H	2.24783500	1.95748300	1.15098800
H	4.06311500	2.51447400	-0.50305900
H	4.12704900	0.81107500	-0.11986100

H	3.40231400	1.33851400	-1.64038700
H	2.42216900	3.78917100	-0.62722000
H	0.82655700	3.57933400	0.05359800
H	1.19204000	2.93136300	-1.55011400
C	-3.35027400	-0.61092200	-0.22543700
O	-4.46991200	-0.43988500	-0.62564600
C	-2.97985000	-1.17093500	1.12780300
H	-2.46054400	-2.12115500	0.96339500
H	-2.24483100	-0.50705200	1.59134700
C	-4.18829600	-1.36686100	2.03882900
H	-4.91021000	-2.05887400	1.60044900
H	-3.86305900	-1.77513900	2.99885300
H	-4.70029000	-0.42054500	2.22716500

11 (TS1)

33

XYZ -Stationary point- [Standard orientation]

C	-1.17457900	1.87433600	-0.20453600
C	-1.64098400	0.59090200	0.07905400
C	-0.78282100	-0.51566600	0.28606600
C	0.55097500	-0.26589800	-0.14378000
C	1.02469100	1.02690600	-0.39331900
C	0.17798500	2.13709400	-0.37782900
C	0.69083000	3.52563200	-0.64613700
C	-1.22637800	-1.83145400	0.98291400
C	-0.09659400	-2.53236100	1.77374100
C	-2.34832500	-1.62055700	2.02711000
O	2.33277300	1.23723900	-0.81773300

I	-3.78430300	0.46570300	-0.20944800
I	1.88673500	-1.81093800	-0.85054300
H	-1.87996700	2.68745800	-0.31542700
H	1.23385500	3.57027800	-1.59424000
H	1.38212200	3.85678700	0.13521900
H	-0.13603400	4.23636500	-0.68622500
H	-1.59383800	-2.52454700	0.21560200
H	-0.54141700	-3.31927700	2.38663600
H	0.65921100	-3.00464700	1.15758900
H	0.39996300	-1.82979300	2.45076700
H	-2.48359700	-2.55244000	2.58062200
H	-3.31407600	-1.36355600	1.60839100
H	-2.06876400	-0.84476800	2.74714500
C	3.38071200	1.45244000	0.05290600
O	4.45416800	1.68355800	-0.43641000
C	3.09342200	1.36740400	1.53238900
H	2.26376900	2.04271800	1.76376500
H	2.71967900	0.35884600	1.73817600
C	4.31337400	1.68101100	2.39333300
H	4.67934800	2.69413300	2.21223700
H	4.04996500	1.59788400	3.45064300
H	5.13152400	0.98690300	2.19033000

11 (TS2)

33

XYZ -Stationary point- [Standard orientation]

C	1.11132600	-1.67034400	-0.88923100
C	1.62806500	-0.48000500	-0.38119100

C	0.87309600	0.71619800	-0.30351400
C	-0.52618500	0.49043600	-0.42537500
C	-1.05247000	-0.72419300	-0.88772400
C	-0.22741500	-1.79403700	-1.23976800
C	-0.76182400	-3.03757900	-1.89911400
C	1.51543400	2.12768700	-0.19489900
C	2.91971000	2.21107100	-0.83747500
C	0.70462900	3.24234500	-0.90140500
O	-2.41686100	-0.84925700	-1.12622100
I	3.55395200	-0.80765600	0.55594100
I	-2.02927300	1.82520400	0.36222200
H	1.76222500	-2.52693400	-1.00791200
H	-1.31100100	-2.78825800	-2.81132300
H	-1.44988200	-3.58873300	-1.25088100
H	0.05755500	-3.70814800	-2.16274400
H	1.60690800	2.38577200	0.86779200
H	3.24239600	3.25446200	-0.82486300
H	3.68768300	1.64194600	-0.32764500
H	2.88719200	1.88590500	-1.88232400
H	1.35394700	4.10984700	-1.03606000
H	-0.16216600	3.58385600	-0.34789000
H	0.37404400	2.92202100	-1.89412900
C	-3.28140800	-1.48831200	-0.25863700
O	-4.42162200	-1.60540700	-0.62027900
C	-2.72930000	-1.96425600	1.06421100
H	-1.94547200	-2.70105600	0.86364700
H	-2.22819300	-1.11750000	1.54263000
C	-3.80148000	-2.55214200	1.97707100

H	-4.28628900	-3.41686500	1.51892400
H	-3.34761700	-2.87458400	2.91743400
H	-4.57453200	-1.81564900	2.20701700

12 (TS1)

25

XYZ -Stationary point- [Standard orientation]

C	1.13706600	1.63329500	0.01294800
C	-0.21492000	1.35677400	-0.10910300
C	-0.76945300	0.06971800	-0.15257000
C	0.21964100	-0.92256400	-0.11912900
C	1.59010600	-0.69911500	-0.00024400
C	2.07658600	0.60769600	0.07844200
C	3.55130000	0.87016000	0.21124900
C	-2.27636600	-0.22737500	-0.29067000
C	-2.71201600	-1.58245200	0.30800500
C	-3.18953700	0.86155100	0.31255100
O	2.47695300	-1.73723900	0.03277700
H	1.45054000	2.66999000	0.04706200
H	4.10623300	0.44546400	-0.63095000
H	3.95413000	0.41208400	1.11989200
H	3.75075500	1.94234500	0.24929100
H	-2.48172000	-0.26533700	-1.36859600
H	-3.80253100	-1.64427900	0.27100500
H	-2.31692400	-2.43960700	-0.23229000
H	-2.41139300	-1.66842900	1.35708100
H	-4.22381100	0.51108500	0.26801500
H	-3.13930000	1.80828300	-0.21967600

H	-2.94703200	1.04540900	1.36402400
H	2.00062700	-2.57586500	-0.04225100
F	-0.11160700	-2.24778400	-0.22591400
F	-1.02502000	2.45341200	-0.21338400

12 (TS2)

25

XYZ -Stationary point- [Standard orientation]

C	-1.13706600	1.63329500	0.01294800
C	0.21492000	1.35677400	-0.10910300
C	0.76945300	0.06971800	-0.15257000
C	-0.21964100	-0.92256400	-0.11912900
C	-1.59010600	-0.69911500	-0.00024400
C	-2.07658600	0.60769600	0.07844200
C	-3.55130000	0.87016000	0.21124900
C	2.27636600	-0.22737500	-0.29067000
C	3.18953700	0.86155100	0.31255100
C	2.71201600	-1.58245200	0.30800500
O	-2.47695300	-1.73723900	0.03277700
H	-1.45054000	2.66999000	0.04706200
H	-3.95413000	0.41208400	1.11989200
H	-4.10623300	0.44546400	-0.63095000
H	-3.75075500	1.94234500	0.24929100
H	2.48172000	-0.26533700	-1.36859600
H	4.22381100	0.51108500	0.26801500
H	3.13930000	1.80828300	-0.21967600
H	2.94703200	1.04540900	1.36402400
H	3.80253100	-1.64427900	0.27100500

H	2.31692400	-2.43960700	-0.23229000
H	2.41139300	-1.66842900	1.35708100
H	-2.00062700	-2.57586500	-0.04225100
F	0.11160700	-2.24778400	-0.22591400
F	1.02502000	2.45341200	-0.21338400

13 (TS1)

25

XYZ -Stationary point- [Standard orientation]

C	-1.09294800	-1.58972100	-0.02709200
C	0.28856600	-1.42135000	-0.12918300
C	0.85685000	-0.14195700	-0.18260000
C	-0.02215100	0.94886900	-0.13045300
C	-1.40137600	0.76943900	-0.02743600
C	-1.97352400	-0.50923200	0.02792800
C	-3.46421200	-0.68532100	0.14449500
C	2.36767000	0.06248200	-0.34700000
C	2.86849600	1.40548400	0.20996600
C	3.20121200	-1.08267100	0.25240900
O	-2.26086100	1.83951300	0.02692900
H	-1.50091300	-2.59522400	0.01442300
H	-3.98957000	-0.22288900	-0.69741600
H	-3.85330600	-0.21602900	1.05402000
H	-3.72670000	-1.74501600	0.16921300
H	2.56490100	0.07046000	-1.42831900
H	3.95117500	1.48149200	0.07501000
H	2.42242600	2.26463300	-0.29602200
H	2.65704400	1.49403700	1.28092300

H	4.26649200	-0.87879000	0.11080900
H	2.99257100	-2.04545100	-0.21862600
H	3.01830000	-1.18428400	1.32735200
H	-1.75741900	2.66301600	-0.01501500
H	0.35537900	1.96475900	-0.17083100
H	0.91500800	-2.30295100	-0.16714900

13 (TS2)

25

XYZ -Stationary point- [Standard orientation]

C	1.09294800	-1.58972100	-0.02709200
C	-0.28856600	-1.42135000	-0.12918300
C	-0.85685000	-0.14195700	-0.18260000
C	0.02215100	0.94886900	-0.13045300
C	1.40137600	0.76943900	-0.02743600
C	1.97352400	-0.50923200	0.02792800
C	3.46421200	-0.68532100	0.14449500
C	-2.36767000	0.06248200	-0.34700000
C	-3.20121200	-1.08267100	0.25240900
C	-2.86849600	1.40548400	0.20996600
O	2.26086100	1.83951300	0.02692900
H	1.50091300	-2.59522400	0.01442300
H	3.85330600	-0.21602900	1.05402000
H	3.98957000	-0.22288900	-0.69741600
H	3.72670000	-1.74501600	0.16921300
H	-2.56490100	0.07046000	-1.42831900
H	-4.26649200	-0.87879000	0.11080900
H	-2.99257100	-2.04545100	-0.21862600

H	-3.01830000	-1.18428400	1.32735200
H	-3.95117500	1.48149200	0.07501000
H	-2.42242600	2.26463300	-0.29602200
H	-2.65704400	1.49403700	1.28092300
H	1.75741900	2.66301600	-0.01501500
H	-0.35537900	1.96475900	-0.17083100
H	-0.91500800	-2.30295100	-0.16714900

Table S62. Activation parameters for the rotational barriers of studied compounds in $CDCl_3$

Compound	Barrier	ΔH≠(298K)	ΔS≠(298K)	ΔG≠(298K)	ΔG≠(298K)eff
	TS1 – *syn*-GS	14.3	-5.3	15.9	15.5
**1**	TS2 – *syn*-GS	14.3	-5.3	15.9	
	TS1 – *anti*-GS	14.4	-4.9	15.9	15.5
	TS2 - *anti*-GS	14.4	-4.9	15.9	
	TS1 – *syn*-GS	15.9	-4.4	17.2	16.8
**2**	TS2 – *syn*-GS	15.9	-4.4	17.2	
	TS1 – *anti*-GS	16.0	-4.8	17.4	17.0
	TS2 - *anti*-GS	16.0	-4.8	17.4	
	TS1 – *syn*-GS	17.1	-5.1	18.6	18.2
**3**	TS2 – *syn*-GS	17.1	-5.1	18.6	
	TS1 – *anti*-GS	17.1	-4.9	18.6	18.1
	TS2 - *anti*-GS	17.1	-4.9	18.6	
	TS1 – *syn*-GS	15.6	-5.6	17.3	17.1
**4**	TS2 – *syn*-GS	16.2	-5.8	17.9	
	TS1 – *anti*-GS	15.6	-4.3	16.9	16.7
	TS2 - *anti*-GS	16.2	-4.5	17.5	
	TS1 – *syn*-GS	16.4	-3.8	17.5	17.5
**5**	TS2 – *syn*-GS	17.3	-4.8	18.7	
	TS1 – *anti*-GS	16.3	-3.5	17.3	17.3
	TS2 - *anti*-GS	17.1	-4.5	18.4	
	TS1 – *syn*-GS	16.2	-3.9	17.4	16.2
**6**	TS2 – *syn*-GS	15.5	-2.4	16.3	
	TS1 – *anti*-GS	16.1	-3.8	17.2	16.0
	TS2 - *anti*-GS	15.5	-2.2	16.1	
	TS1 – *syn*-GS	17.3	-4.3	18.6	17.7
**7**	TS2 – *syn*-GS	16.6	-4.0	17.8	
	TS1 – *anti*-GS	17.2	-4.7	18.6	17.6
	TS2 - *anti*-GS	16.4	-4.4	17.7	
	TS1 – *syn*-GS	15.5	-3.3	16.5	16.4
**8**	TS2 – *syn*-GS	16.3	-4.8	17.7	
	TS1 – *anti*-GS	15.5	-4.2	16.7	16.7
	TS2 - *anti*-GS	16.3	-5.7	18.0	
	TS1 – *syn*-GS	16.2	-3.3	17.1	17.1
**9**	TS2 – *syn*-GS	17.6	-3.0	18.5	
	TS1 – *anti*-GS	16.0	-2.8	16.9	17.0
	TS2 - *anti*-GS	17.4	-2.5	18.2	

Table S62. Activation parameters for the rotational barriers of studied compounds in $CDCl_3$ (cont.)

Compound	Barrier	ΔH≠(298K)	ΔS≠(298K)	ΔG≠(298K)	ΔG≠(298K)eff
	TS1 – *syn*-GS	15.6	-3.8	16.8	16.6
**10**	TS2 – *syn*-GS	16.2	-3.9	17.3	
	TS1 – *anti*-GS	15.6	-4.2	16.9	16.7
	TS2 - *anti*-GS	16.2	-4.3	17.4	
	TS1 – *syn*-GS	16.6	-4.6	18.0	17.9
**11**	TS2 – *syn*-GS	17.5	-5.8	19.2	
	TS1 – *anti*-GS	16.5	-3.3	17.5	17.4
	TS2 - *anti*-GS	17.3	-4.5	18.7	
	TS1 – *syn*-GS	6.2	-5.6	7.9	7.5
**12**	TS2 – *syn*-GS	6.2	-5.6	7.9	
	TS1 – *anti*-GS	6.2	-5.8	8.0	7.6
	TS2 - *anti*-GS	6.2	-5.8	8.0	
	TS1 – *syn*-GS	1.7	-7.1	3.9	3.4
**13**	TS2 – *syn*-GS	1.7	-7.1	3.9	
	TS1 – *anti*-GS	1.9	-7.1	4.0	3.6
	TS2 - *anti*-GS	1.9	-7.1	4.0	

ZPE, thermal and entropy corrections are calculated at SMD($CDCl_3$)/B3LYP/6-311G(d,p) level of theory for 298K.

Theoretical predictions of coupling constants in carvacrol:

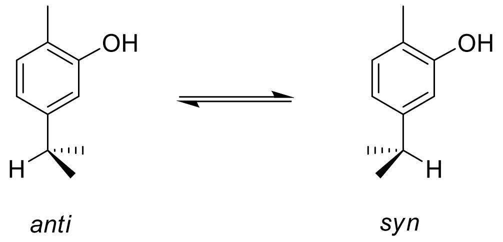

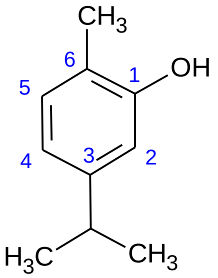

GIAO/B3LYP/aug-cc-pVTZ-J// B3LYP/6-311++G(d,p) calculations of coupling constants of two rotamers of carvacrol ($^{3}J_{anti} < {}^{3}J_{syn}$):

	$^{3}J_{(C\text{-}2,CH)}$	$^{3}J_{(C\text{-}4,CH)}$
Anti	5.8	6.9
Syn	7.2	5.5

For methyl group $^{3}J_{anti} \sim {}^{3}J_{syn}$: 6.9 and 6.8 Hz

GIAO/B3LYP/6-311++G**-J// B3LYP/6-311++G(d,p) calculations of coupling constants of two rotamers of carvacrol ($^{3}J_{anti} < {}^{3}J_{syn}$):

	$^{3}J_{(C\text{-}2,CH)}$	$^{3}J_{(C\text{-}4,CH)}$
Anti	5.9	7.1
Syn	7.5	5.4

For methyl group $^{3}J_{anti} \sim {}^{3}J_{syn}$: 6.9 and 6.9 Hz

Table S63. Comparison of GIAO/B3LYP/6-311++G(d,p) calculated and experimental chemical shifts of **1**

Syn	Calc.	Exp.
H-5	7.12	7.07
OH	5.42	5.92
CH	3.78	3.78
Ph-CH_3	2.24	2.21
CH-CH_3	1.50	1.36
C-1	156.50	148.96
C-3	147.03	138.84
C-4	140.59	124.46
C-5	137.78	129.89
C-2	134.03	120.06
C-6	131.72	123.83
CH	40.09	31.30
CH-CH_3	20.19	19.45
Ph-CH_3	19.64	16.38

Anti	Calc.	Exp.
H-5	7.23	7.01
OH	5.49	5.81
CH	3.93	3.69
Ph-CH_3	2.25	2.20
CH-CH_3	1.47	1.38
C-1	157.16	148.10
C-3	147.19	138.75
C-4	139.83	124.41
C-5	136.53	131.33
C-2	134.77	120.48
C-6	131.76	123.79
CH	39.53	31.77
CH-CH_3	20.23	19.45
Ph-CH_3	19.67	16.30

Table S64. Comparison of GIAO/B3LYP/6-311++G(d,p) calculated and experimental chemical shifts of **2**

Syn	Calc.	Exp.
H-5	7.28	7.29
OH	5.63	5.87
CH	3.82	3.75
Ph-CH_3	2.25	2.24
CH-CH_3	1.53	1.42
C-1	158.32	149.70
C-3	151.47	141.31
C-5	142.17	135.77
C-4	138.57	111.85
C-2	135.31	115.00
C-6	132.19	124.57
CH	44.06	36.31
CH-CH_3	20.00	19.54
Ph-CH_3	19.86	16.53

Anti	Calc.	Exp.
H-5	7.38	7.33
OH	5.84	6.04
CH	4.02	3.83
Ph-CH_3	2.26	2.25
CH-CH_3	1.49	1.40
C-1	158.98	150.50
C-3	151.59	141.43
C-5	140.65	134.08
C-4	139.42	115.49
C-2	134.43	111.22
C-6	132.29	124.57
CH	43.43	35.93
CH-CH_3	20.08	19.54
Ph-CH_3	19.89	16.67

Table S65. Comparison of GIAO/B3LYP/6-311++G(d,p) calculated and experimental chemical shifts of **3**

Syn	Calc.	Exp.
H-5	7.50	7.64
OH	5.60	5.72
CH	3.76	3.61
Ph-CH_3	2.24	2.23
CH-CH_3	1.55	1.40
C-1	161.03	152.88
C-3	157.74	145.83
C-5	148.84	144.30
C-6	132.68	124.49
C-2	131.45	98.34
C-4	129.05	80.60
CH	50.34	43.53
CH-CH_3	20.43	19.78
Ph-CH_3	19.49	16.80

Anti	Calc.	Exp.
H-5	7.61	7.65
OH	5.94	5.98
CH	3.95	3.65
Ph-CH_3	2.24	2.24
CH-CH_3	1.52	1.40
C-1	161.55	153.55
C-3	157.89	146.01
C-5	146.85	141.97
C-4	133.35	92.49
C-6	132.94	124.44
C-2	127.26	86.82
CH	49.54	43.38
CH-CH_3	20.61	19.72
Ph-CH_3	19.44	17.10

Table S66. Comparison of GIAO/B3LYP/6-311++G(d,p) calculated and experimental chemical shifts of **4**

Syn	Calc.	Exp.
H-5	7.35	7.34
OH	4.20	3.91
CH	3.73	3.75
Ph-CH_3	2.30	2.26
CH-CH_3	1.50	1.38
C-1	163.29	154.53
C-3	153.80	142.89
C-2	147.52	122.68
C-4	144.83	116.98
C-5	142.05	136.12
C-6	139.79	131.74
OCH_3	59.76	60.02
CH	42.85	35.59
CH-CH_3	19.86	19.37
Ph-CH_3	19.55	16.23

Anti	Calc.	Exp.
H-5	7.46	7.39
OH	4.01	3.80
CH	3.72	3.75
Ph-CH_3	2.30	2.26
CH-CH_3	1.54	1.40
C-1	164.11	155.41
C-3	153.85	142.88
C-2	147.08	119.19
C-4	145.70	120.76
C-5	140.34	134.25
C-6	139.82	131.71
OCH_3	59.94	60.16
CH	43.96	36.62
CH-CH_3	19.80	19.44
Ph-CH_3	19.35	16.34

Table S67. Comparison of GIAO/B3LYP/6-311++G(d,p) calculated and experimental chemical shifts of **5**

Syn	Calc.	Exp.
H-5	7.60	7.75
OH	4.27	3.62
CH	3.71	3.76
Ph-CH_3	2.33	2.29
CH-CH_3	1.53	1.43
C-1	165.87	158.71
C-3	160.13	147.63
C-5	149.06	142.49
C-2	144.97	98.87
C-6	139.13	131.00
C-4	136.56	93.37
OCH_3	59.50	60.24
CH	48.47	43.85
CH-CH_3	19.31	19.68
Ph-CH_3	19.12	16.71

Anti	Calc.	Exp.
H-5	7.72	7.75
OH	3.93	3.83
CH	3.69	3.76
Ph-CH_3	2.33	2.29
CH-CH_3	1.59	1.39
C-1	166.71	157.88
C-3	160.33	147.54
C-5	146.86	145.04
C-2	141.19	86.57
C-6	140.92	104.86
C-4	139.23	131.20
OCH_3	59.69	60.04
CH	50.21	42.74
CH-CH_3	19.45	19.77
Ph-CH_3	19.26	16.45

Table S68. Comparison of GIAO/B3LYP/6-311++G(d,p) calculated and experimental chemical shifts of **6**

Syn	Calc.	Exp.
H-5	7.36	7.36
=CH	6.72	6.16
=CH	5.56	5.50
=CH	5.48	5.34
CH_2	4.35	4.38
CH	4.20	3.94
Ph-CH_3	2.32	2.27
CH-CH_3	1.51	1.41
C-1	162.26	153.55
C-3	153.73	142.93
C-2	147.46	122.92
=CH	145.35	133.20
C-4	144.84	117.10
C-5	142.01	136.09
C-6	140.24	131.91
=CH_2	123.35	118.37
CH_2	77.52	73.23
CH	42.93	35.66
CH-CH_3	19.73	19.44
Ph-CH_3	19.45	16.48

Anti	Calc.	Exp.
H-5	7.46	7.41
=CH	6.73	6.16
=CH	5.55	5.50
=CH	5.48	5.34
CH_2	4.34	4.38
CH	4.04	3.82
Ph-CH_3	2.32	2.28
CH-CH_3	1.54	1.43
C-1	163.11	154.43
C-3	153.79	142.96
C-2	147.00	119.41
C-4	145.76	120.90
=CH	145.33	133.22
C-5	140.25	134.22
C-6	140.25	131.88
=CH_2	123.31	118.27
CH_2	77.71	73.31
CH	43.95	36.35
CH-CH_3	19.80	19.50
Ph-CH_3	19.50	16.58

Table S69. Comparison of GIAO/B3LYP/6-311++G(d,p) calculated and experimental chemical shifts of **7**

Syn	Calc.	Exp.
H-5	7.61	7.75
=CH	6.76	6.18
=CH	5.57	5.53
=CH	5.50	5.35
CH_2	4.34	4.35
CH	4.26	3.83
Ph-CH_3	2.33	2.27
CH-CH_3	1.53	1.39
C-1	164.98	156.79
C-3	160.06	147.50
C-5	149.04	145.00
=CH	145.18	133.06
C-2	145.02	105.23
C-6	139.68	131.45
C-4	136.63	86.71
=CH_2	123.44	118.57
CH_2	77.30	73.08
CH	48.55	42.83
CH-CH_3	19.38	19.69
Ph-CH_3	19.34	16.54

Anti	Calc.	Exp.
H-5	7.73	7.75
=CH	6.76	6.18
=CH	5.56	5.53
=CH	5.50	5.35
CH_2	4.32	4.34
CH	3.94	3.62
Ph-CH_3	2.34	2.27
CH-CH_3	1.59	1.43
C-1	165.83	157.60
C-3	160.28	147.61
C-5	146.83	142.44
=CH	145.05	133.02
C-2	141.24	93.67
C-4	141.05	99.06
C-6	139.76	131.24
=CH_2	123.44	118.48
CH_2	77.44	73.17
CH	50.21	43.89
CH-CH_3	19.47	19.78
Ph-CH_3	19.47	16.78

Table S70. Comparison of GIAO/B3LYP/6-311++G(d,p) calculated and experimental chemical shifts of **8**

Syn	Calc.	Exp.
H-5	7.43	7.41
CH	4.18	3.90
Ph-CH_3	2.28	2.13
COCH	2.09	2.93
CH-CH_3	1.52	1.39
COCH-CH_3	1.14	1.29
C=O	181.80	174.52
C-1	157.20	146.15
C-3	154.21	143.06
C-4	146.51	119.24
C-2	146.40	121.37
C-5	142.27	135.97
C-6	139.10	131.06
CH	43.26	35.76
COCH	36.37	34.35
COCH-CH_3	22.88	19.32
Ph-CH_3	20.82	16.61
CH-CH_3	19.67	19.16

Anti	Calc.	Exp.
H-5	7.53	7.46
CH	4.09	3.85
Ph-CH_3	2.27	2.13
COCH	2.07	2.94
CH-CH_3	1.53	1.42
COCH-CH_3	1.15	1.40
C=O	181.88	174.50
C-1	158.03	147.05
C-3	154.28	143.15
C-4	147.42	123.05
C-2	145.86	117.82
C-5	140.52	134.14
C-6	139.15	131.04
CH	44.02	36.32
COCH	36.38	34.34
COCH-CH_3	22.98	19.49
Ph-CH_3	20.88	16.72
CH-CH_3	19.65	19.38

Table S71. Comparison of GIAO/B3LYP/6-311++G(d,p) calculated and experimental chemical shifts of **9**

Syn	Calc.	Exp.
H-5	7.66	7.80
CH	4.28	3.78
Ph-CH_3	2.24	2.11
COCH	2.01	2.95
CH-CH_3	1.57	1.41
COCH-CH_3	1.18	1.40
C=O	181.96	174.42
C-3	160.70	149.82
C-1	160.19	147.66
C-5	149.04	144.73
C-2	144.69	88.94
C-6	139.00	103.33
C-4	138.55	130.84
Ph-CH	48.86	42.91
CO-CH	36.55	34.63
COCH-CH_3	23.34	19.66
Ph-CH_3	21.15	16.75
CH-CH_3	19.30	19.04

Anti	Calc.	Exp.
H-5	7.77	7.79
CH	4.02	3.65
Ph-CH_3	2.24	2.11
COCH	1.99	2.96
CH-CH_3	1.60	1.42
COCH-CH_3	1.17	1.40
C=O	182.02	174.46
C-1	161.11	150.74
C-3	160.86	147.81
C-5	146.80	142.18
C-4	143.01	101.32
C-2	141.05	91.65
C-6	139.18	130.70
Ph-CH	50.34	43.87
CO-CH	36.56	34.61
COCH-CH_3	23.35	19.70
Ph-CH_3	21.31	17.00
CH-CH_3	19.33	19.47

Table S72. Comparison of GIAO/B3LYP/6-311++G(d,p) calculated and experimental chemical shifts of **10**

Syn	Calc.	Exp.
H-5	7.40	7.42
CH	4.17	3.89
Ph-CH_3	2.17	2.15
CH_2	1.89	2.74
CH-CH_3	1.53	1.42
CH_2-CH_3	1.02	1.32
C=O	177.36	172.16
C-1	157.23	146.22
C-3	154.67	143.06
C-4	146.88	119.33
C-2	146.10	121.37
C-5	142.20	135.97
C-6	138.65	131.12
CH	43.20	35.81
CH_2	31.01	27.61
Ph-CH_3	20.23	16.66
CH-CH_3	19.65	19.44
CH_2-CH_3	10.71	9.38

Anti	Calc.	Exp.
H-5	7.50	7.47
CH	4.06	3.85
Ph-CH_3	2.16	2.15
CH_2	1.89	2.74
CH-CH_3	1.54	1.41
CH_2-CH_3	1.02	1.32
C=O	177.46	172.16
C-1	158.05	147.13
C-3	154.70	143.16
C-4	147.76	123.14
C-2	145.60	117.81
C-5	140.46	134.13
C-6	138.69	131.11
CH	44.05	36.31
CH_2	31.03	27.54
Ph-CH_3	20.30	16.77
CH-CH_3	19.68	19.44
CH_2-CH_3	10.67	9.40

Table S73. Comparison of GIAO/B3LYP/6-311++G(d,p) calculated and experimental chemical shifts of **11**

Syn	Calc.	Exp.
H-5	7.64	7.80
CH	4.25	3.77
Ph-CH_3	2.15	2.12
CH_2	1.90	2.73
CH-CH_3	1.55	1.39
CH_2-CH_3	1.04	1.33
C=O	177.34	172.07
C-3	161.09	147.66
C-1	160.28	149.87
C-5	149.03	144.73
C-2	144.26	103.36
C-4	138.77	89.04
C-6	138.26	130.82
CH	48.82	42.96
CH_2	31.43	28.12
Ph-CH_3	20.53	16.79
CH-CH_3	19.27	19.66
CH_2-CH_3	10.65	9.32

Anti	Calc.	Exp.
H-5	7.75	7.80
CH	4.00	3.65
Ph-CH_3	2.15	2.13
CH_2	1.88	2.73
CH-CH_3	1.60	1.41
CH_2-CH_3	1.01	1.33
C=O	177.47	172.12
C-3	161.27	147.82
C-1	161.17	150.81
C-5	146.82	142.17
C-4	143.18	101.43
C-2	140.53	91.66
C-6	138.42	130.82
CH	50.36	43.85
CH_2	31.43	28.04
Ph-CH_3	20.66	17.03
CH-CH_3	19.35	19.69
CH_2-CH_3	10.62	9.35

References:

1. Adams, R.P., 2007. Identification of Essential Oil Components by Gas Chromatography/ mass Spectrometry. Allured publishing corporation. Carol Stream, IL, USA.
2. Binsch, G. In *Dynamic NMR spectroscopy*, Jackman, L. M., Cotton, F.A., Eds.; Academic Press, New York, USA, 1975, pp. 45–82.
3. Heinzer, J.; Oth, J. Iterative Least-Squares Lineshape Fitting of ^{1}H-decoupled ^{13}C-DNMR.Spectra *Helv. Chim. Acta* **1981**, *64*, 258. DOI: 10.1002/hlca.19810640127.
4. Frisch, M.J.; Trucks, G.W.; Schlegel, H.B.; Scuseria, G.E.; Robb, M.A.; Cheeseman, J.R.; Scalmani, G.; Barone, V.; et al. *Gaussian*, Gaussian, Inc., Wallingford, CT, USA, 2013.
5. Becke, A. D. Density-functional thermochemistry. III. The role of exact exchange. *J. Chem. Phys.* **1993**, *98*, 5648–5652. DOI: 10.1063/1.464913
6. Hariharan, P.C.; Pople, J.A. The influence of polarization functions on molecular orbital hydrogenation energies. *Theoret. Chim. Acta* **1973**, *28*, 213–222. DOI: doi.org/10.1007/BF00533485.
7. Marenich, A.V.; Cramer, C.J; Truhlar, D.G. Universal solvation model based on solute electron density and on a continuum model of the solvent defined by the bulk dielectric constant and atomic surface tensions. *J. Phys. Chem. B*, **2009**, *113*, 6378–6396. DOI: 10.1021/jp810292n.
8. Wolinski, K.; Hinton, J.F.; Pulay, P. Efficient implementation of the gauge-independent atomic orbital method for NMR chemical shift calculations. *J. Am. Chem. Soc.* **1990**, *112*, 8251–8260. DOI: 10.1021/ja00179a005.
9. Cheeseman, J.R.; Trucks, G.W.; Keith, T.A.; Frisch, M.J. A comparison of models for calculating nuclear magnetic resonance shielding tensors. *J. Chem. Phys.* **1996**, *104*, 5497–5509. DOI: 10.1063/1.471789.
10. Helgaker, T.; Watson, M.; Handy, N.C. Analytical calculation of nuclear magnetic resonance indirect spin–spin coupling constants at the generalized gradient approximation and hybrid levels of density-functional theory. *J. Chem. Phys.* **2000**, *113*, 9402–9409. DOI: 10.1063/1.1321296.
11. Sychrovský, V.; Gräfenstein, J.; Cremer, D. Nuclear magnetic resonance spin–spin coupling constants from coupled perturbed density functional theory. *J. Chem. Phys.* 2000, *113*, 3530–3547. DOI: 10.1063/1.1286806.
12. Peralta, J.E.; Scuseria, G.E.; Cheeseman, J.R.; Frisch, M.J. Basis set dependence of NMR spin–spin couplings in density functional theory calculations: First row and hydrogen atoms. *Chem. Phys. Lett.* **2003**, *375*, 452–458. DOI: 10.1016/S0009-2614(03)00886-8.
13. Deng, W.; Cheeseman, J.R.; Frisch, M.J. Calculation of nuclear spin−spin coupling constants of molecules with first and second row atoms in study of basis set dependence. *J. Chem. Theory Comput.* **2006**, *2*, 1028–1037. DOI: 10.1021/ct600110u.
14. Enevoldsen, T.; Oddershede, J.; Sauer, S.P.A. Correlated calculations of indirect nuclear spin-spin coupling constants using second-order polarization propagator approximations: SOPPA and SOPPA (CCSD). Theor. Chem. Acc. 1998, 100, 275–284. DOI: 10.1007/s002140050388
15. Provasi, P.F.; Aucar, G.A.; Sauer, S.P.A. The effect of lone pairs and electronegativity on the indirect nuclear spin–spin coupling constants in CH2X (X= CH2, NH, O, S): Ab initio calculations using optimized contracted basis sets. J. Chem. Phys. 2001, 115, 1324–1334. DOI: 10.1063/1.1379331
16. Kjær, H.; Sauer, S.P.A. Pople Style Basis Sets for the Calculation of NMR Spin-Spin Coupling Constants: the 6-31G-J and 6-311G-J Basis Sets. J. Chem. Theory Comput. 2011, 7, 4070-4076. DOI: 10.1021/ct200546q
